# Annulative coupling of vinylboronic esters: aryne-triggered 1,2-metallate rearrangement†

**DOI:** 10.1039/d2sc02623f

**Published:** 2022-07-25

**Authors:** Haruki Mizoguchi, Hidetoshi Kamada, Kazuki Morimoto, Ryuji Yoshida, Akira Sakakura

**Affiliations:** Graduate School of Natural Science and Technology, Okayama University 3-1-1 Tsushima-naka Kita-ku Okayama 700-8530 Japan h_mizoguchi@okayama-u.ac.jp sakakura@okayama-u.ac.jp

## Abstract

A stereoselective annulative coupling of a vinylboronic ester ate-complex with arynes producing cyclic borinic esters has been developed. An annulation reaction that proceeded through the formation of two C–C bonds and a C–B bond was realized by exploiting a 1,2-metallate rearrangement of boronate triggered by the addition of a vinyl group to the strained triple bond of an aryne. The generated aryl anion would then cyclize to a boron atom to complete the annulation cascade. The annulated borinic ester could be converted to boronic acids and their derivatives by oxidation, halogenation, and cross-coupling. Particularly, halogenation and Suzuki–Miyaura coupling proceeded in a site-selective fashion and produced highly substituted alkylboronic acid derivatives.

## Abstract 2

A stereoselective annulative coupling of a vinylboronic ester ate-complex with arynes producing cyclic borinic esters has been developed.

## Graphical abstract

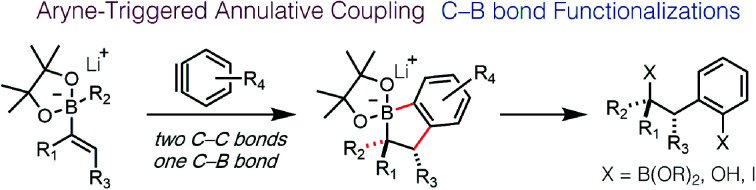

## Introduction

**Scheme 1:**
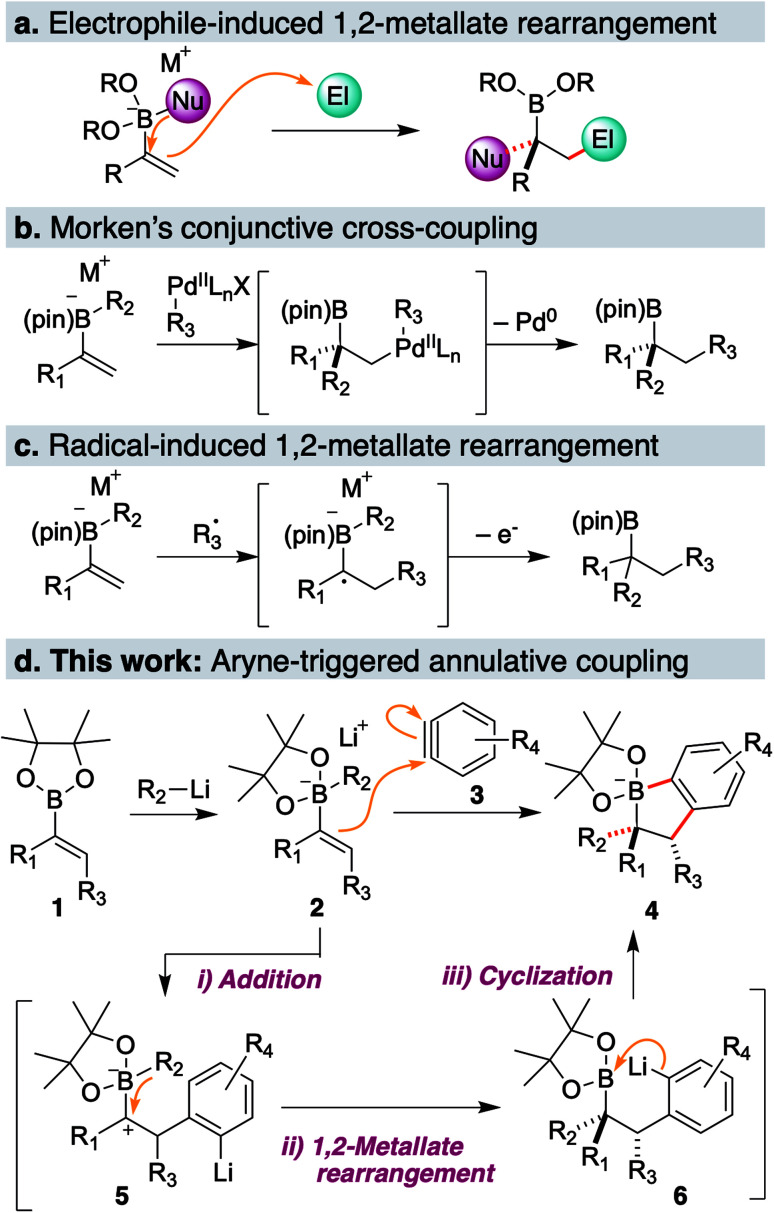
(a) 1,2-Metallate rearrangement of a vinylboronic ester ate-complex; (b) organopalladium induced conjunctive cross-coupling; (c) radical induced 1,2-metallate rearrangement; (d) this work: aryne-triggered annulative coupling of a vinylboronic ester ate-complex.

1,2-Metallate rearrangement of a boronic ester ate-complex is a powerful means to construct substituted alkylboron compounds. Alkylboronic acids and their derivatives are broadly appreciated as a key component in modern organic synthesis, since their carbon–boron bond can be used in a variety of stereospecific bond-forming events.^1^ For the rapid introduction of complexity and diversity into alkylboronic esters, the carbon electrophile-induced 1,2-metallate rearrangement of a vinylboronic ester ate-complex is especially attractive (Scheme 1a).^2,3^ This reaction can lead to two carbon–carbon bonds in the form of 1,2-difunctionalization of alkene and produce highly substituted alkylboronic esters. For example, Morken and coworkers discovered the palladium-catalyzed enantioselective conjunctive cross-coupling of a vinylboronic ester ate-complex (Scheme 1b).^4^ Coordination of the organometal species induces rearrangement, and subsequent reductive elimination provides a coupled product through C–C bond formation. Studer, Aggarwal and Renaud independently reported that an electrophilic radical could trigger metallate rearrangement (Scheme 1c).^5–7^ Addition of the radical species to a vinyl group followed by single-electron oxidation produces a carbocation adjacent to a boron ate-complex, which induces a 1,2-shift. Very recently, Ready and coworkers reported that an *in situ*-generated π-allyl iridium could promote rearrangement in an enantioselective fashion.^8^ Although these classes of electrophilic species and several others,^9–11^ enable a new entry point to various alkyl boronic esters, a carbon electrophile which could trigger the 1,2-metallate rearrangement of a simple, non-strained vinylboronic ester is still quite limited.

To discover a new class of carbon electrophiles that could be used in 1,2-metallate rearrangement, we were interested in the reactivity of arynes. Arynes are a highly reactive aromatic species possessing a strained triple bond.^12^ Because of this strained and weak triple bond, aryne is reactive toward a variety of nucleophiles. Thus, we envisioned that arynes could also react with a π-bond of a vinylboronic ester ate-complex to trigger 1,2-metallate rearrangement. By taking advantage of the nature of arynes, which generate the aryl anion after nucleophilic cleavage of a strained triple bond, we envisaged that the coupling of vinylboronic ester ate-complex 2 with aryne 3 would proceed in an annulative manner to give dihydrobenzoborole 4 (Scheme 1d). The annulation reaction would proceed through (1) a nucleophilic addition of a π-bond of the vinylboronic ester to the aryne (2 → 5), (2) 1,2-rearrangement of a substituent on the boron ate-complex to the adjacent carbocation (5 → 6), and (3) cyclization of the resulting aryl anion to a boron atom, to form borinic ester ate-complex 4. This borinic ester ate-complex 4 possesses a C(aromatic)–B bond and a C(alkyl)–B bond, and therefore, it could be a useful intermediate for synthesizing diverse boronic ester derivatives. Herein, we report an aryne-triggered strain-release 1,2-metallate rearrangement of simple vinyl boronic esters for the expeditious synthesis of structurally complex and diverse organoboronic ester derivatives.

## Results and discussion

**Scheme 2:**
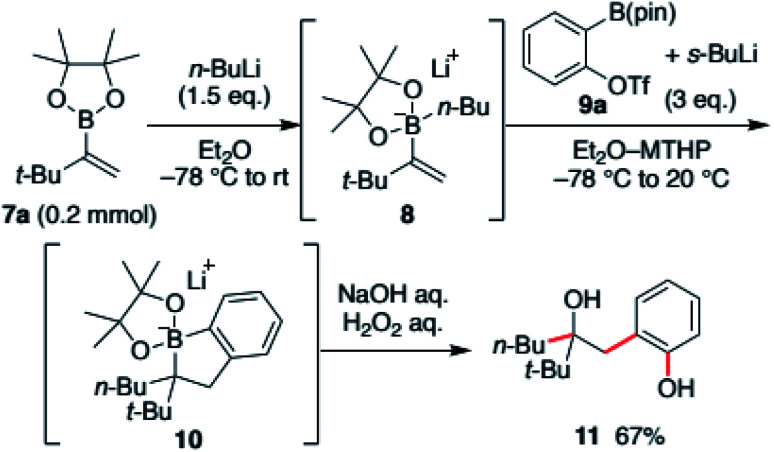
Optimized reaction conditions for aryne-triggered annulative coupling.

Our investigation commenced with an exploration of the reaction conditions that could promote the desired cascade reaction. The reaction was conducted using *tert*-butyl-substituted vinyl boronic ester 7a. The boronic ester was first converted to the ate-complex 8 with *n*-butyllithium and treated with benzyne generated *in situ* from benzyne precursors. The results were evaluated after the crude mixture was treated with H_2_O_2_ with the intent to convert the intermediate, borinic ester ate-complex 10, to hydroxyphenol 11 (Scheme 2).

**Scheme 3:**
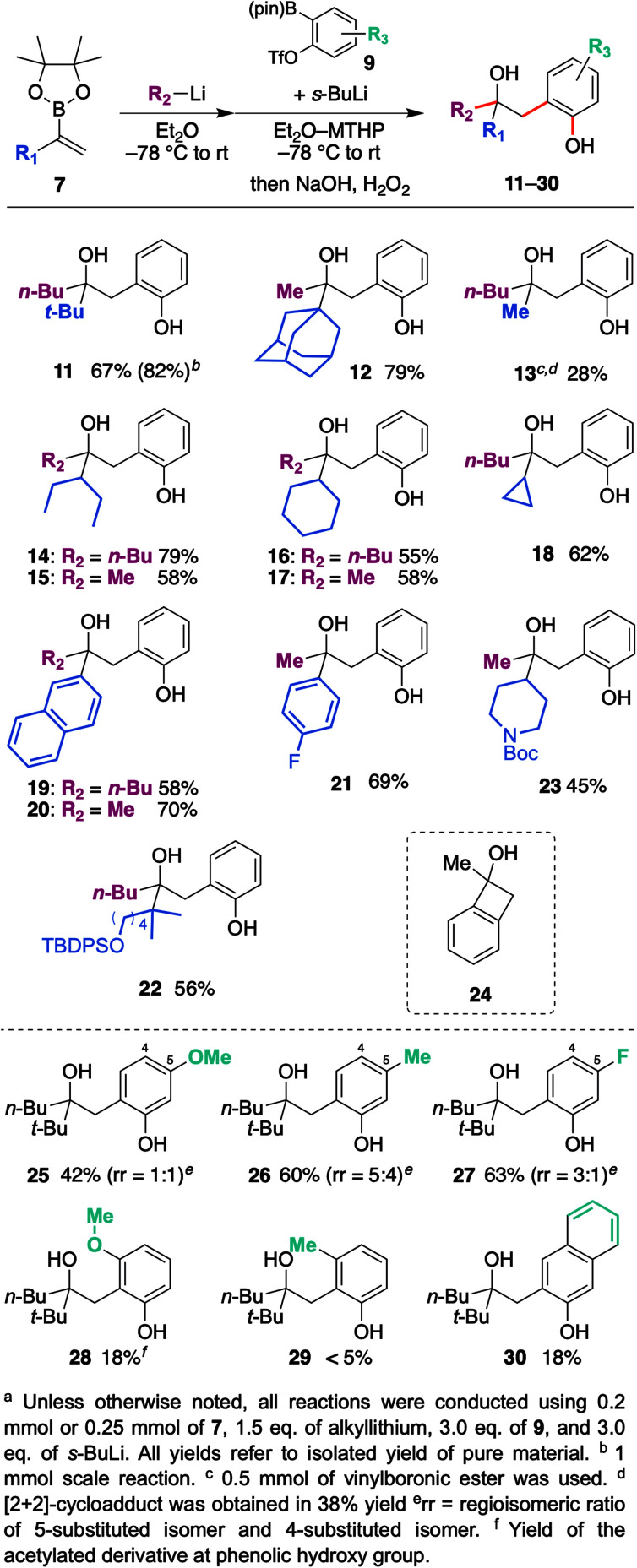
Scope and limitations of vinylboronic esters, organo-lithiums, and aryne precursors.

After screening various aryne-precursors, solvents and conditions, we found that the use of Hosoya's reagent 9 activated with *s*-BuLi was crucial for the reaction cascade (see the ESI† for the details).^13^ Treatment of the vinylboronic ester ate-complex dissolved in 4-methyltetrahydropyran (MTHP)^14^ with preactivated Hosoya's reagent at −78 °C to 20 °C afforded desired hydroxyphenol 11 in 67% yield. Noteworthily, the use of MTHP, a less nucleophilic cyclic ether solvent with similar solubilization properties to THF, was important for obtaining the coupled product in high yield. The use of THF resulted in the formation of significant amounts of the THF adduct of benzyne. The reaction could be easily scaled-up, and the product was obtained in 82% yield in a 1 mmol scale reaction (Scheme 3).

With the optimized reaction conditions in hand, we investigated the substrate scope of vinyl boronic esters (Scheme 3). In addition to 7a, a vinylboronic ester with a sterically demanding adamantyl ring afforded coupled product 12 in high yield. It is worth noting that a methyl group shifted efficiently in this reaction, whereas it is generally recognized as a poor migrating group.^15^ On the other hand, when a phenyl group was used (R_2_ = Ph) instead of a butyl or methyl group, the reactivity of the ate-complex became quite low, and the coupled product was not obtained. Since the aryl group is generally a good migrating group, we speculated that steric bulkiness around the boron atom hampers the reaction between a vinyl group and benzyne. Methyl-substituted vinylboronic ester afforded the desired product 13 in low yield, and a significant amount of [2 + 2] cycloadduct 24 was obtained. In addition, unfortunately, the non-substituted vinylboronic ester (R_1_ = H) only produced the [2 + 2] cycloadduct. Secondary alkyl groups such as 3-pentyl, cyclohexyl, and cyclopropyl worked well, and coupled products 14–18 were obtained in good yields. Not only an aliphatic group, aromatic substituents such as the naphthyl group and 4-fluorophenyl group also produced desired products 19–21 in good yields. Substrates bearing functional groups were also examined, and silyl ether as well as Boc-protected amine were found to be tolerated to give corresponding hydroxyphenol 22, 23.

The scope and limitations of the aryne precursors were next investigated (Scheme 3). 4-Methoxy, 4-methyl, and 4-fluoro-substituted benzyne afforded the desired products 25–27 in respective yields of 42, 60, and 63% as a mixture of regioisomers.^16,17^ These results suggest that this coupling is insensitive to the electronic nature of the aromatic ring. On the other hand, 3-methoxy- and 3-methyl-substituted benzyne afforded desired products 28 and 29 in low yield with high regioselectivity. In the case of these substrates, an aryl anion would be generated next to the substituent. Therefore, subsequent cyclization to a boron atom might be difficult due to steric repulsion between the substituent and pinacol moiety. 2,3-Naphthalyne also afforded the corresponding product 30, but the yield was unexpectedly low.

**Scheme 4:**
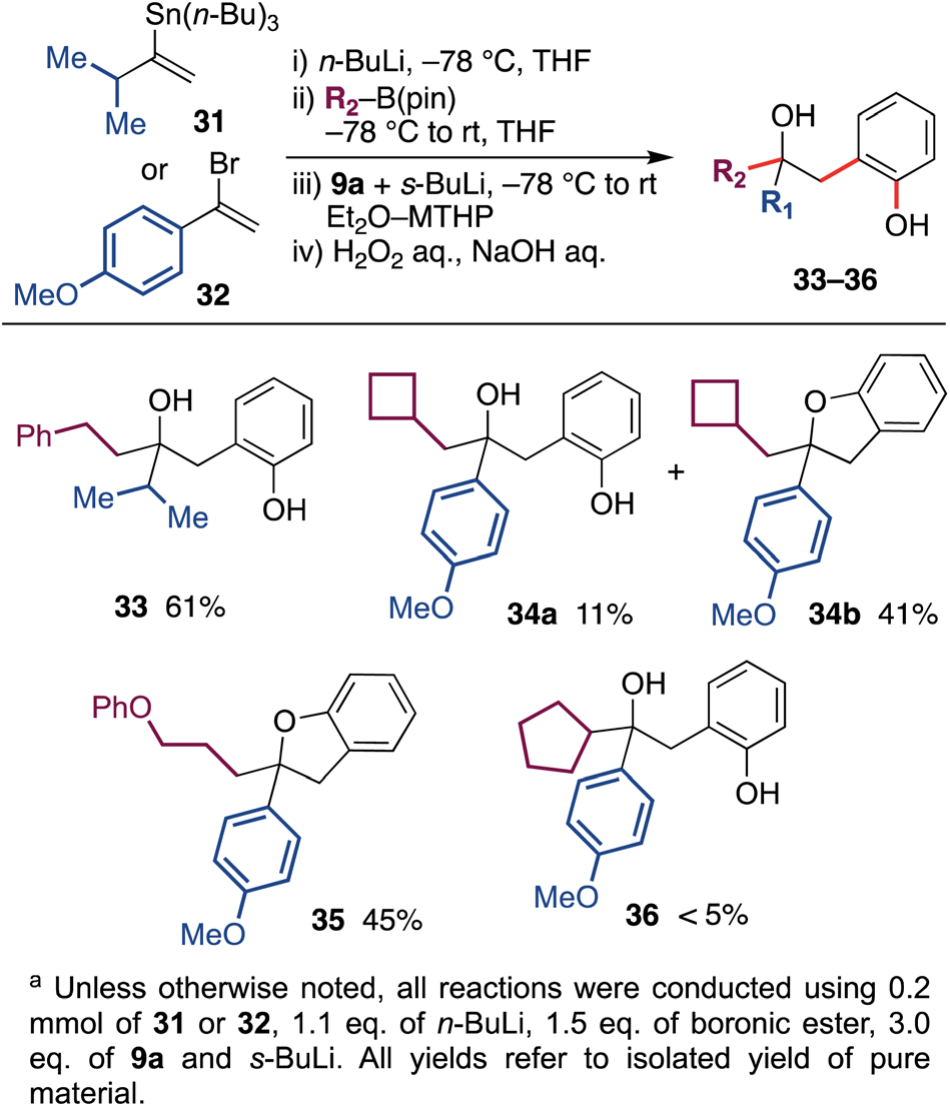
Scope and limitation of the migrating group.

In the protocol described above, the migrating group was limited to butyl and methyl groups because of the availability of corresponding alkyl lithium. Therefore, we next developed a protocol to generate the ate-complex from vinyllithium and organoboronic esters (Scheme 4). With the use of isopropyl-substituted vinyltin 31 and 4-methoxyphenyl-substituted vinylbromide 32 as a substrate, the reaction was conducted through a lithiation, borylation and aryne-triggered rearrangement sequence. As a result, phenethyl boronic ester and cyclobutylmethyl boronic ester afforded the desired hydroxyphenol 33 and 34 in good yield. In the latter example, some of the hydroxyphenol 34a was converted to dihydrobenzofuran 34b during purification.^18^ In addition, boronic ester containing an aryl ether moiety was also tolerated, and a coupled product 35 was obtained in the form of dihydrobenzofuran. On the other hand, the reaction using cyclopentyl boronic ester did not produce the desired product 36 in satisfactory yield, probably due to the steric bulkiness.

**Scheme 5:**
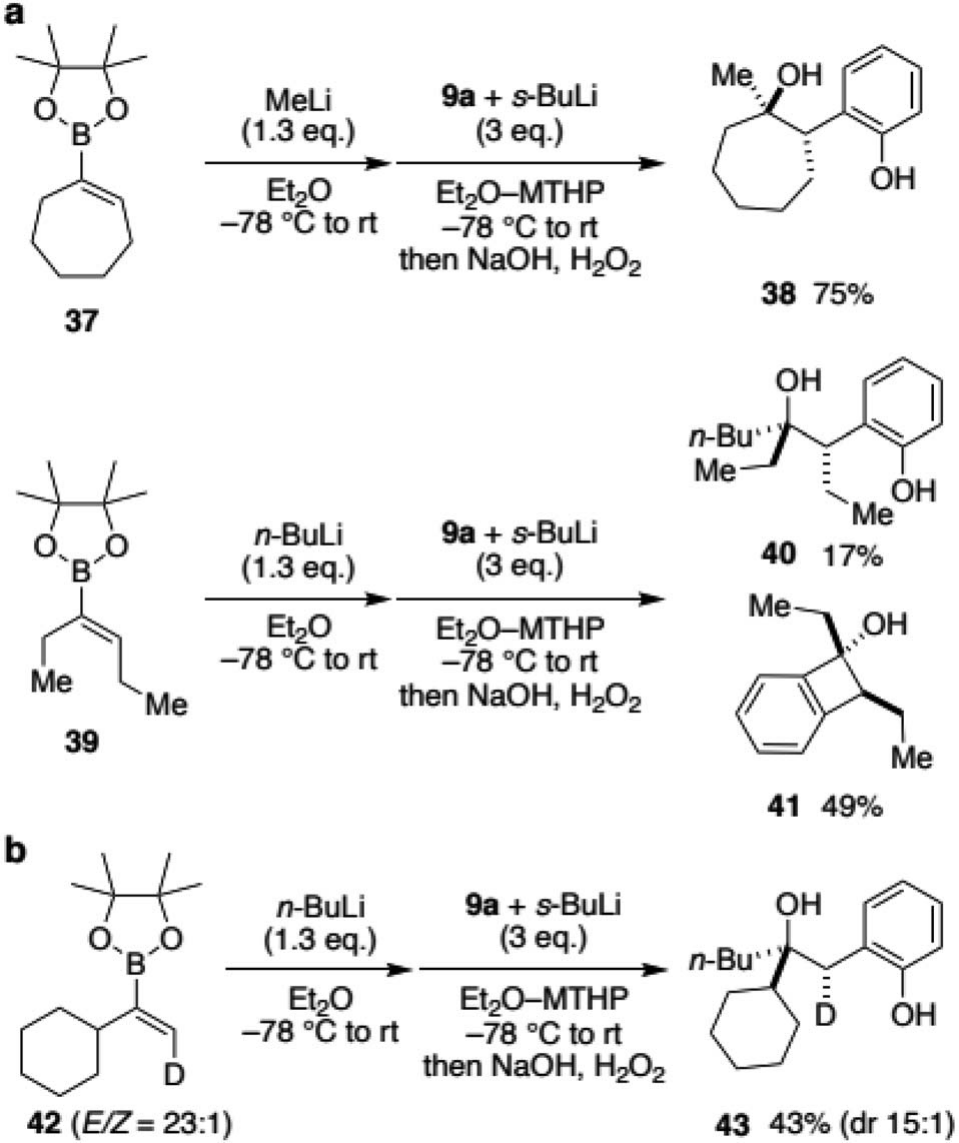
(a) Annulative coupling using boronic esters possessing internal alkenes; (b) annulative coupling of a deuterated vinylboronic ester.

To expand the scope of vinylboronic ester and to gain insight into the stereochemical outcome, we next reacted boronic esters with an internal alkene (Scheme 5a). Cycloheptenylboronic ester 37 was converted to the ate-complex and treated with benzyne. To our delight, desired hydroxyphenol 38 was obtained in 75% yield as a single isomer.^19^ Not only the cyclic vinylboronate, annulative coupling of acyclic 39 was also tested. Although the major product was the [2 + 2]-type adduct 41, desired hydroxyphenol 40 was obtained as a single diastereomer. These results were surprising because the 1,2-metallate rearrangement was initially expected to be a non-stereoselective process that proceeded through a carbocation intermediate. To gain further insight into the reaction mechanism for the stereoselectivity, deuterated vinylboronic ester 42 (*E*/*Z* = 23 : 1) was prepared and subjected to the developed conditions (Scheme 5b). When *n*-BuLi was used as a nucleophile, hydroxyphenol 43 was obtained in 43% yield with only a slight decrease in the stereoisomeric ratio (dr 15 : 1). Their stereochemistry was determined as shown using 2D NMR analysis of a carbonate derivative (see the ESI† for details). Interestingly, the results suggested that the reaction with benzyne and 1,2-rearrangement of the butyl group proceeded on the same face of the alkene.^20^ Accordingly, the reaction would not proceed through an anti-concerted pathway, nor a stepwise pathway including a ring cleavage of the [2 + 2] cycloadduct intermediate, both of which provide anti-adducts.

**Scheme 6:**
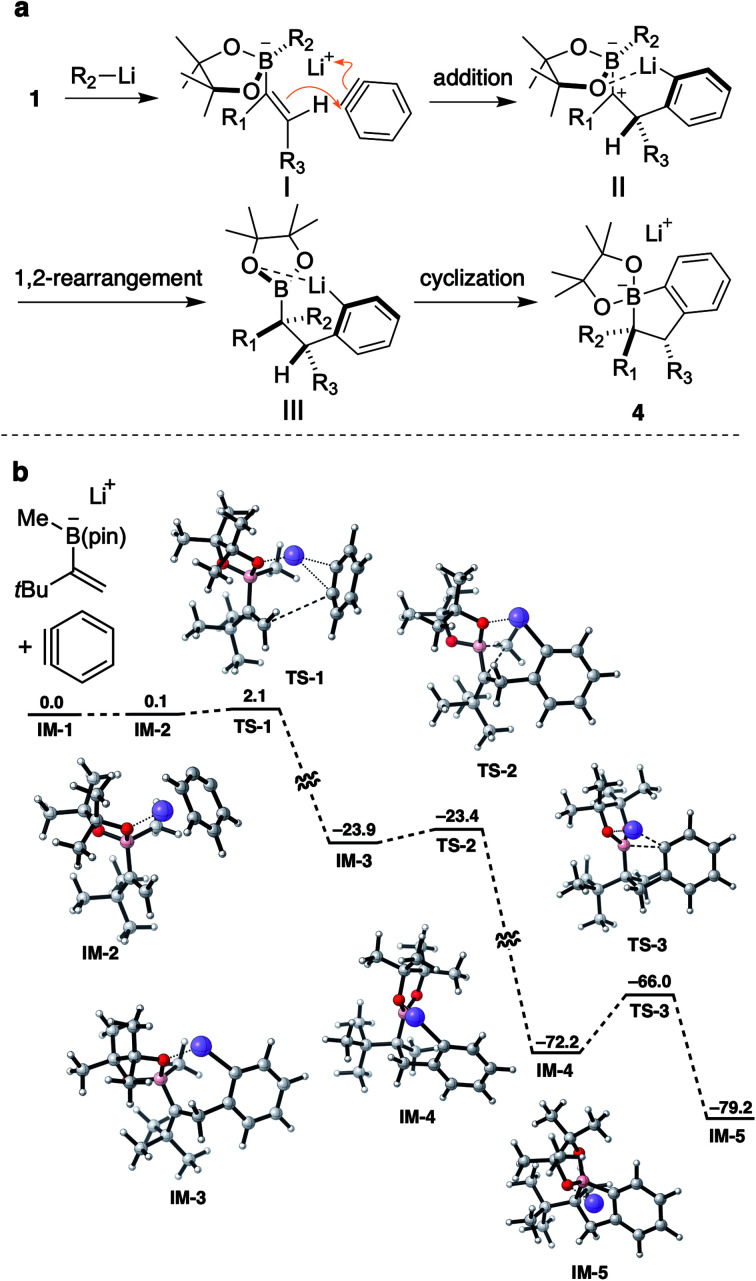
(a) Proposed reaction mechanism; (b) calculated reaction coordinates for aryne triggered annulative coupling. Optimized geometry was calculated using DFT (B3LYP-D3BJ/6-31G(d); the SMD solvation model with Et_2_O). Δ*G* values are in kcal mol^−1^, calculated using DFT (B3LYP-D3BJ/6-311+G(d,p); the SMD solvation model with Et_2_O). Calculated structures are shown with the following color code: grey: carbon, red: oxygen, pink: boron, and purple: lithium.

To support the experimental results, density functional theory (DFT) calculations were performed on a model structure (Scheme 6b).^21^ According to calculations, the annulation reaction would proceed in a stepwise process through three transition states TS-1–TS-3. The reaction would be initiated by an addition of an alkene to benzyne generating carbocation IM-3*via*TS-1 with an activation barrier of 2.0 kcal mol^−1^. During this step, a π-bond of benzyne is cleaved and the resulting carbanion coordinates to the lithium ion. Apparently, the lithium atom also interacts with an oxygen atom of the pinacol ester moiety to form a chelated structure. From IM-3, a methyl group on the boron atom migrates to the adjacent carbocation to give boronic ester IM-4*via*TS-2. This metallate rearrangement steps is highly exothermic, with a small activation barrier of 0.5 kcal mol^−1^, suggesting the fast and efficient 1,2-rearrangement even with the presence of a bulky *tert*-butyl group next to the carbocation. Finally, cyclization of aryllithium to a boron atom would proceed through TS-3 with an activation barrier of 6.2 kcal mol^−1^ to give IM-5.

Based on these experimental results and calculations, we proposed a mechanism for annulative coupling as shown in Scheme 6a. Initially, the attack of a vinyl group of ate-complex I to a benzyne would generate carbocation II. To avoid steric repulsion between the benzyne and a pinacol group, the benzyne would approach the same face of the vinyl group which the migrating group (R_2_) of boronate occupied. Because of this way of approach, the reaction of vinylboronates bearing a large migrating group such as phenyl and cyclopentyl with benzyne might be difficult, resulting in failure of the reaction.^22^

From carbocation II, stereoselective 1,2-rearrangement would occur to form a carbon–carbon bond. The conformation of carbocation II would be fixed by the chelated structure. Therefore, 1,2-rearrangement would proceed without rotation of a carbon–boron bond, maintaining the stereochemical information of alkene. After rearrangement, aryllithium III would be formed and cyclize to a boron atom generating borinic ester 4. From intermediate II, formation of a [2 + 2] adduct through cyclization would be a competitive pathway. Considering the experimental results, tertiary and secondary alkyl groups would be large enough to encumber the cyclization of II by the steric hindrance and 1,2-metallate rearrangement of R_2_ predominantly occurring to give the desired product 4. On the other hand, when small groups such as methyl and hydrogen were substituted, the cyclization would be fast enough to give a significant amount of the [2 + 2] adduct as a byproduct (Scheme 3). In addition, predominant formation of the [2 + 2] adduct of 39 also suggested that the rate of the cyclization is important: the Thorpe–Ingold effect would facilitate the cyclization to give cyclobutane.

**Scheme 7:**
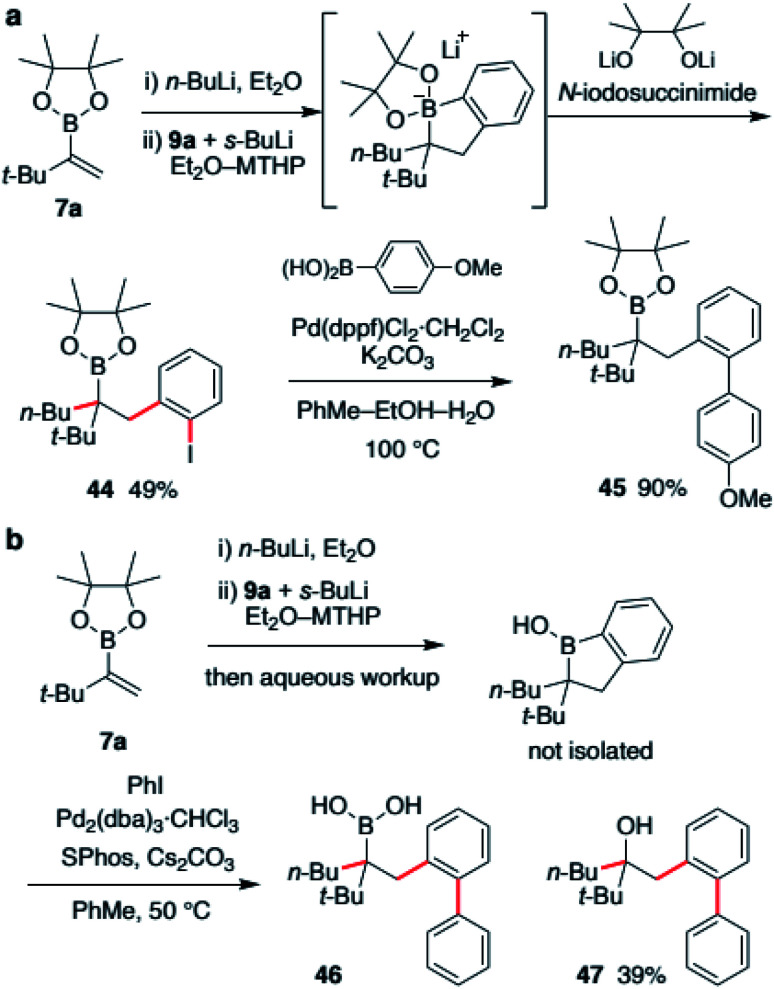
(a) Selective iodination of the cyclic borinic ester intermediate; (b) selective Suzuki–Miyaura coupling of the cyclic borinic ester intermediate for entry to highly substituted boronic acid derivatives.

Finally, derivatization of a borinic ester intermediate was investigated. The intermediate possesses both a C(sp^2^)–B bond and a C(sp^3^)–B bond. Therefore, we envisioned that selective functionalization of one of the bonds would deliver a highly substituted organoboronic ester. We first examined halogenation (Scheme 7a). The boronic ester ate-complex was generated according to a described process and treated with NIS (*N*-iodosuccinimide). As a result, the C(sp^2^)–B bond was found to be selectively iodinated. With the use of a dilithiated pinacol as an additive, desired alkylboronic ester 44 was obtained in 49% yield with high site-selectivity. This aryl iodide 44 was easily converted to biaryl derivative *via* Suzuki–Miyaura coupling to produce boronic ester 45 in 90% yield. Suzuki–Miyaura coupling of the borinic ester intermediate was also investigated (Scheme 7b). After substantial examination, we found that cross-coupling of the crude mixture of borinic ester after aqueous workup was effective. Treatment of the crude mixture with iodobenzene in the presence of Pd_2_(dba)_3_ and SPhos resulted in the formation of biaryl product 46 in 38% yield along with 39% of oxidized derivative 47. Although suppression of this overoxidation is under investigation at this stage, we were able to demonstrate a two-step, single purification process for the synthesis of highly functionalized alkyl boronic acid from a simple vinylboronic ester through the formation of three carbon–carbon bonds.

## Conclusions

In summary, we have developed annulative coupling of simple, non-activated vinylboronic esters which proceeds through an aryne-triggered 1,2-metallate rearrangement. By exploiting a highly strained aryne as an electrophile, coupling, 1,2-metallate rearrangement, and cyclization of the generated aryl anion proceeded to afford a cyclic borinic ester ate-complex through the formation of two C–C bonds and a C–B bond. A variety of vinylboronic ester, aryne, and migrating groups were applicable to this reaction cascade and afforded hydroxyphenols after oxidation. Although the yields are not always high, it is noteworthy that the cascade reaction forms two carbon–carbon bonds and two carbon–oxygen bonds in a one-pot process. Site-selective iodination and Suzuki–Miyaura coupling of the borinic ester intermediate were also achieved, and highly substituted alkylboronic acid derivatives were synthesized. Based on a mechanistic investigation using deuterated substrates, the 1,2-rearrangement was proposed to proceed through a stepwise process to give a product in a stereoselective manner. Compared to established electrophile-induced 1,2-metallate reactions of vinylboronic esters, the present aryne-triggered annulative coupling approach could provide an additional handle (carbon–boron bond) for the further diversification of boronic acid derivatives. Therefore, we believe that this method will be useful for rapidly preparing structurally complex and diverse boronic acids and their derivatives relevant to bioactive molecules.

## Author contributions

H. M. and A. S. conceived and directed the project. H. M., H. K., K. M., and R. Y. performed the experimental work. H. M., H. K., K. M., and R. Y. collected and analyzed the spectroscopic data. H. M. and A. S. wrote the manuscript. All the authors discussed the results and contributed to the preparation of the manuscript.

## Conflicts of interest

There are no conflicts to declare.

## Supplementary Material

SC-013-D2SC02623F-s001
