# Supplementary material for: Annulative coupling of vinylboronic esters: aryne-triggered 1,2-metallate rearrangement
Source: Chem Sci. 2022 Jul 25;13(33):9580–5. doi: 10.1039/d2sc02623f (PMC9400639; doi:10.1039/d2sc02623f)
Supplement: SC-013-D2SC02623F-s001 [file SC-013-D2SC02623F-s001.pdf]

Electronic Supplementary Material (ESI) for Chemical Science.
This journal is © The Royal Society of Chemistry 2022

Supporting Information

Annulative Coupling of Vinylboronic Esters: Aryne-Triggered 1,2-Metallate Rearrangement

Haruki Mizoguchi*, Hidetoshi Kamada, Kazuki Morimoto, Ryuji Yoshida, and Akira Sakakura*

Table of contents

General Methods S2

Preparation of Substrates S3–S11

Examination of benzyne precursors S12-S13

Optimization of Reaction Conditions S14

Procedures for the Aryne-triggered Annulative Coupling of Vinyl Boronic Ester Ate-complexes S15–S42

DFT Calculations for the Reaction Mechanism S43–S88

^{1}H and ^{13}C NMR Spectra of New Compounds S89–S128

General Methods

IR spectra were recorded on a SHIMADZU FTIR-8400 spectrometer. ^{1}H NMR spectra were measured on JEOL JNMECZ600R spectrometer (600 MHz), Varian NMR System 600 PS600 spectrometer (600 MHz), a Varian 400-MR ASW spectrometer (400 MHz), and a Varian Mercury-300 spectrometer (300 MHz) at ambient temperature. Data were recorded as follows: chemical shift in ppm from the solvent resonance employed as the internal standard ($CHCl_3$ at 7.26 ppm) on the δ scale, multiplicity (s = singlet; d = doublet; t = triplet; q = quartet; pent = pentet/quintet; m = multiplet), coupling constant (Hz), and integration. ^{13}C NMR spectra were measured on JEOL JNMECZ600R spectrometer (150* MHz), Varian NMR System 600 PS600 spectrometer (150 MHz) and a Varian 400-MR ASW spectrometer (100 MHz) at ambient temperature. Chemical shifts were recorded in ppm from the solvent resonance employed as the internal standard ($CDCl_3$ at 77.16 ppm). For TLC analysis, Merck precoated TLC plates (silica gel 60 F_{254} 0.25 mm) were used. For preparative column chromatography, Kanto Chemical Co., Inc. silica gel 60 N (spherical, neutral), Fuji Silysia Chemical PSQ100B, and Kanto Chemical Co., Inc. silica gel 60 (spherical) NH_2 were used. High- and low-resolution mass spectral analysis (HRMS) was measured on a JEOL JMS-700 Mstation (FAB) and a Bruker micrOTOF II (ESI) at Chemical Instrument Facility, Okayama University. Dry toluene, tetrahydrofuran (THF), dichloromethane (CH_2Cl_2), toluene, dimethyl sulfoxide (DMSO), methanol (MeOH), diethyl ether (Et_2O), ethyl acetate (EtOAc) and chloroform ($CHCl_3$) were purchased from Kanto Chemical Co., Inc. or Wako Pure Chemical Industries Ltd. as the "anhydrous" and stored under nitrogen. Cyclopentyl methyl ether (CPME), 1,2-dimethoxyethane (DME), and MTHP (4-methyltetrahydropyran) were purchased from Kanto Chemical Co., Inc., TCI Co., LTD or Wako Pure Chemical Industries Ltd. and distilled from sodium/benzophenone ketyl prior to use. Other materials were obtained from commercial supplies and used without further purification. All reactions were conducted in a flame dried glassware under nitrogen atmosphere, otherwise noted.

Preparation of substrates

2-(3,3-dimethylbut-1-en-2-yl)-4,4,5,5-tetramethyl-1,3,2-dioxaborolane (7a)

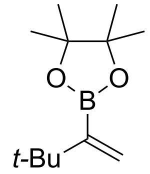

7a

7a was prepared according to a reported procedure.[1]

2-(1-(adamantan-1-yl)vinyl)-4,4,5,5-tetramethyl-1,3,2-dioxaborolane (7b)

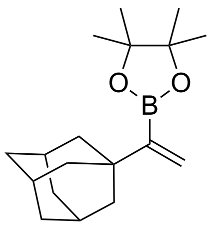

7b

7b was prepared according to a reported procedure.[2]

4,4,5,5-tetramethyl-2-(prop-1-en-2-yl)-1,3,2-dioxaborolane (7c)

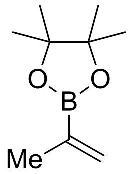

7c

Commercially available

2-(3-ethylpent-1-en-2-yl)-4,4,5,5-tetramethyl-1,3,2-dioxaborolane (7d)

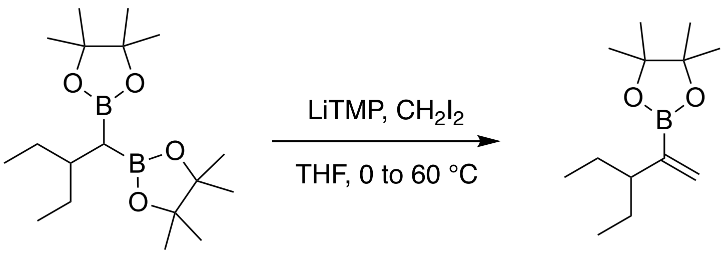

S16 **7d**

To a stirred solution of LiTMP in THF (4.5 mL) prepared from 2,2,6,6-tetramethylpiperidine (560 μL, 3.30 mmol) and *n*-BuLi (2.0 M in cyclohexane, 1.65 mL, 3.30 mmol) was added a solution of **S16**[3] (1.01 g, 3.00 mmol) in THF (9.0 mL) dropwise at 0 °C. After 5 min, a solution of CH_2I_2 (485 μL, 6.01 mmol) in THF (6.0 mL) was added. After being stirred at the same temperature for 10 min, the reaction mixture was warmed up to 60 °C and stirred for 2 h 40 min. The mixture was the cooled down to rt and the reaction was quenched with saturated aqueous NH_4Cl. The mixture was extracted with Et_2O. The organic layers were washed with brine, dried over Na_2SO_4 and concentrated under reduced pressure. The crude product was purified by column chromatography on silica gel to obtain **7d** (309 mg, 1.38 mmol, 46%).

7d: colorless oil; IR (film) 2961, 2932, 2872, 1609, 1370, 1304, 1146, 970, 943, 855, 756, 669 cm^{-1}; ^{1}H NMR (600 MHz, $CDCl_3$) δ 5.81 (d, *J* = 3.6 Hz, 1H), 5.53 (d, *J* = 3.6 Hz, 1H), 1.93 (pent, *J* = 7.2 Hz, 1H), 1.44 (app pent, *J* = 7.2 Hz, 4H), 1.25 (s, 12H), 0.79 (t, *J* = 7.2 Hz, 6H); ^{13}C NMR (150 MHz, $CDCl_3$) δ 129.1, 83.1, 49.6, 26.9, 24.8, 12.2; HRMS (ESI) *m/z* calcd for $C_{13}H_{25}BNaO_2$ [M+Na]$^+$ 247.1840, found 247.1840.

2-(1-cyclohexylvinyl)-4,4,5,5-tetramethyl-1,3,2-dioxaborolane (7e)

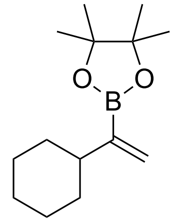

7e

7e was prepared according to a reported procedure.[4]

2-(1-cyclopropylvinyl)-4,4,5,5-tetramethyl-1,3,2-dioxaborolane (7f)

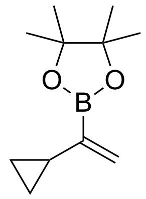

7f

7f was prepared according to a reported procedure.[5]

4,4,5,5-tetramethyl-2-(1-(naphthalen-2-yl)vinyl)-1,3,2-dioxaborolane (7g)

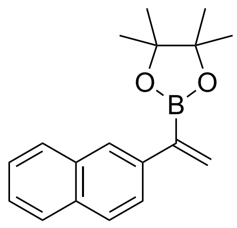

7g

7g was prepared according to a reported procedure.[1]

***tert*-butyl((5,5-dimethyl-6-(4,4,5,5-tetramethyl-1,3,2-dioxaborolan-2-yl)hept-6-en-1-yl)oxy)diphenylsilane (7h)**

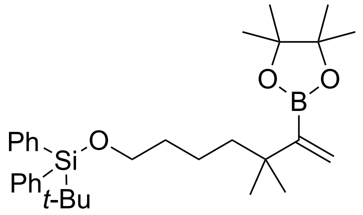

7h

7h was prepared according to a reported procedure.[6]

***tert*-butyl 4-(1-(4,4,5,5-tetramethyl-1,3,2-dioxaborolan-2-yl)vinyl)piperidine-1-carboxylate (7i)**

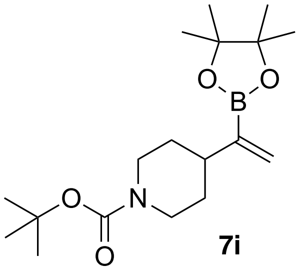

7i

7i was prepared according to a reported procedure.[7]

2-(1-(4-fluorophenyl)vinyl)-4,4,5,5-tetramethyl-1,3,2-dioxaborolane (7j)

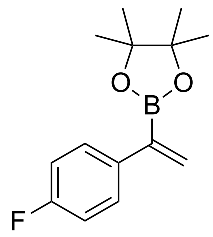

7j

7j was prepared according to a reported procedure. [8]

2-(4,4,5,5-tetramethyl-1,3,2-dioxaborolan-2-yl)phenyl trifluoromethanesulfonate (9a)

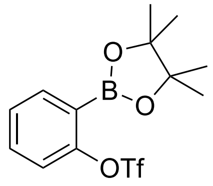

9a

9a was prepared according to a reported procedure.[9]

4-methoxy-2-(4,4,5,5-tetramethyl-1,3,2-dioxaborolan-2-yl)phenyl trifluoromethanesulfonate (9b)

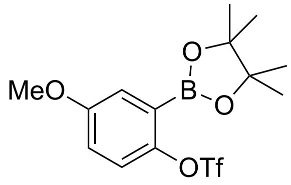

9b

9b was prepared according to a reported procedure.[10]

4-methyl-2-(4,4,5,5-tetramethyl-1,3,2-dioxaborolan-2-yl)phenyl trifluoromethanesulfonate (9c)

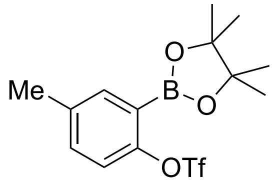

9c

9c was prepared according to a reported procedure.[10]

5-fluoro-2-(4,4,5,5-tetramethyl-1,3,2-dioxaborolan-2-yl)phenyl trifluoromethanesulfonate (9d)

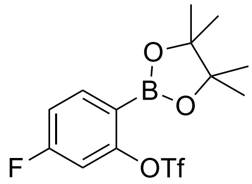

9d

9d was prepared from 4-fluoro-2-hydroxyphenylboronic acid following the reported procedure.[9]

9d: colorless oil; IR (film) 3525, 3433, 3358, 3327, 2980, 1622, 1485, 1423, 1203, 1053, 879, 705, 613 cm^{-1}; ^{1}H NMR (400 MHz, $CDCl_3$) δ 7.55–7.50 (m, 1H), 7.20–7.16 (m, 2H), 1.36 (s, 12H); ^{13}C NMR (100 MHz, $CDCl_3$) δ 161.3 (d, *J* = 249 Hz), 149.8 (d, *J* = 3.0 Hz), 123.4 (d, *J* = 23 Hz), 123.1 (d, *J* = 8.0 Hz), 119.7 (d, *J* = 24 Hz), 119.0 (q, *J* = 319 Hz), 85.1, 24.9; HRMS (ESI) *m/z* calcd for $C_{13}H_{15}BF_4NaO_5S$ $[M+Na]^+$ 393.0562, found 393.0558.

2-methoxy-6-(4,4,5,5-tetramethyl-1,3,2-dioxaborolan-2-yl)phenyl trifluoromethanesulfonate (9e)

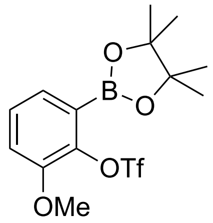

9e

9e was prepared according to a reported procedure.[9]

2-methyl-6-(4,4,5,5-tetramethyl-1,3,2-dioxaborolan-2-yl)phenyl trifluoromethanesulfonate (9f)

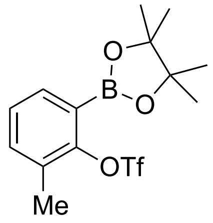

9f

9f was prepared from 2-hydroxy-3-methylphenylboronic acid following the reported procedure.[9]

9f: colorless oil; IR (film) 3502, 3464, 3365, 2982, 1614, 1458, 1359, 1070, 893, 611 cm^{-1}; ^{1}H NMR (400 MHz, $CDCl_3$) δ 7.85 (dd, *J* = 7.2, 1.7 Hz, 1H), 7.36 (dd, *J* = 7.6, 0.4 Hz, 1H), 7.26 (app t, *J* = 7.6 Hz, 1H), 2.37 (s, 3H), 1.37 (s, 12H); ^{13}C NMR (100 MHz, $CDCl_3$) δ 152.3, 135.2, 134.6, 130.3, 127.7, 118.9 (q, *J* = 318 Hz),

84.7, 25.0, 16.4 (q, J = 1.5 Hz); HRMS (ESI) m/z calcd for $C_{14}H_{18}BF_3NaO_5S$ $[M+Na]^+$ 389.0812, found 389.0777.

3-(4,4,5,5-tetramethyl-1,3,2-dioxaborolan-2-yl)naphthalen-2-yl trifluoromethanesulfonate (9g)

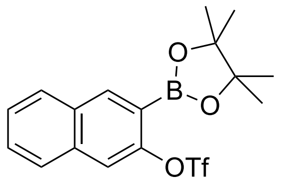

9g

9g was prepared according to a reported procedure.[9]

tributyl(3-methylbut-1-en-2-yl)stannane (31)

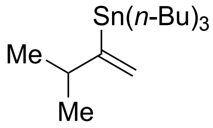

31

31 was prepared according to a reported procedure.[11]

1-(1-bromovinyl)-4-methoxybenzene (32)

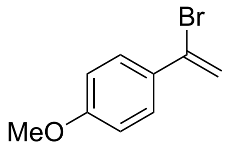

32

32 was prepared according to a reported procedure.[12]

2-(cyclohept-1-en-1-yl)-4,4,5,5-tetramethyl-1,3,2-dioxaborolane (37)

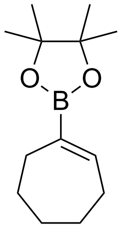

37

37 was prepared according to a reported procedure.[13]

(*Z*)-2-(hex-3-en-3-yl)-4,4,5,5-tetramethyl-1,3,2-dioxaborolane (39)

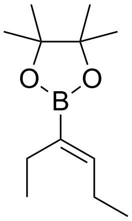

39

39 was prepared according to a reported procedure.[14]

2-(cyclohex-1-en-1-yl)-4,4,5,5-tetramethyl-1,3,2-dioxaborolane (S11)

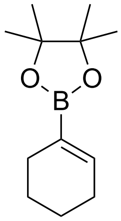

S11

S11 was prepared according to a reported procedure. [15]

(*Z*)-2-(cyclooct-1-en-1-yl)-4,4,5,5-tetramethyl-1,3,2-dioxaborolane (S12)

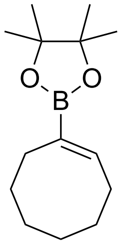

S12

S12 was prepared according to a reported procedure. [15]

2-(cyclobutylmethyl)-4,4,5,5-tetramethyl-1,3,2-dioxaborolane (S3)

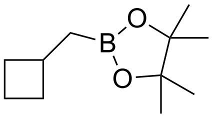

S3

S3 was prepared according to a reported procedure.[16]

4,4,5,5-tetramethyl-2-(3-phenoxypropyl)-1,3,2-dioxaborolane (S4)

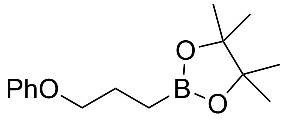

S4

S4 was prepared according to a reported procedure.[17,18]

(*Z*)-2-(1-cyclohexylvinyl-2-*d*)-4,4,5,5-tetramethyl-1,3,2-dioxaborolane (42)

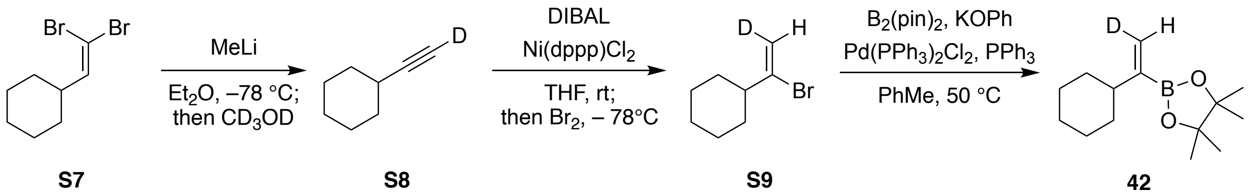

To a stirred solution of **S7** (2.55 g, 9.50 mmol) in Et_2O (30 mL) was added MeLi (1.5 M in Et_2O, 14.7 mL, 22.0 mmol) at –78 °C. After being stirred for 40 min, the mixture was warmed up to rt and further stirred for 30 min. The reaction was quenched with CD_3OD and diluted with saturated aqueous NH_4Cl. The mixture was extracted with Et_2O. The organic layers were washed with brine, dried over Na_2SO_4 and concentrated under reduced pressure. The crude mixture of **S8** was used for next reaction without purifications.

To a solution of Ni(dppp)Cl_2 (270 mg, 0.50 mmol) in THF (25 mL) was added DIBAL-H (1.0 M in hexane, 12 mL, 12 mmol) at room temperature. After 5 min, the mixture was cooled to 0 °C and a solution of **S8** (crude mixture, prepared above) was added. The mixture was warmed up to room temperature and stirred at room temperature for 4 h. After the period of time, the mixture was cooled to –78 °C and treated with bromine (0.665 mL, 12.9 mmol). After 30 min, the mixture was warmed up to rt and further stirred for 30 min. The reaction mixture was poured in to 0 °C solution of saturated aqueous Rochelle salt and resulting clouded mixture was stirred for 30 min. The mixture was extracted with Et_2O. The organic layers were washed with brine, dried over Na_2SO_4 and concentrated under reduced pressure. The crude mixture of **S9** was roughly purified by column chromatography on silica gel and used for next reaction as a mixture (904 mg, < 4.75 mmol).

Half amount of the above mixture (450 mg, < 2.38 mmol), $B_2(pin)_2$ (665 mg, 2.62 mmol), $Pd(PPh_3)_2Cl_2$ (50.1 mg, 0.0714 mmol), PPh_3 (37.5 mg, 0.142 mmol), and KOPh (472 mg, 3.57 mmol) was mixed in

toluene (15 mL) and the resulting suspension was stirred at 50 °C for 24 h. The mixture was cooled to room temperature and filtered through a pad of silica gel using hexane/Et_2O mixture (5:1) as an eluent. The eluate was concentrated under reduced pressure and purified by column chromatography on silica gel to obtain **42** (200 mg, 0.843 mmol). By comparing the benzylic methylene proton signals with that of **7e**, *E*/*Z* ratio was determined as 23:1.

42: ^{1}H NMR (600 MHz, $CDCl_3$) δ 5.69 (s, 1H), 2.12–2.05 (m, 1H), 1.78–1.64 (m, 5H), 1.35–1.20 (m, 2H), 1.26 (s, 12H), 1.20–1.08 (m, 3H)

Examination of benzyne precursors

As an initial study, we have tried to use Kobayashi's benzyne precursor (2-trimethylsilyl)phenyl trifluoromethanesulfonate) activating with cesium fluoride for our annulative coupling. We investigated various solvents, temperature, and additive (crown ether), however, desired hydroxyphenol was not obtained in satisfactory yield. With using aprotic solvents such as THF and DMF, no coupling product was observed. On the other hand, when solvents having relatively acidic proton such as AcOEt and acetonitrile, some coupling product was produced. However, the product was tertiary alcohol **S10**. This product would be produced by the protonation of arylanion intermediate before the cyclization to boron atom. To suppress the protonation, we conducted the rection using isobutyronitrile as a solvent (Scheme S1). To our delight, we were able to obtain desired hydroxyphenol **13**. However, the major product was still protonated product **S10**, and we concluded that the use of Kobayashi's precursor is not suitable.

Scheme S1

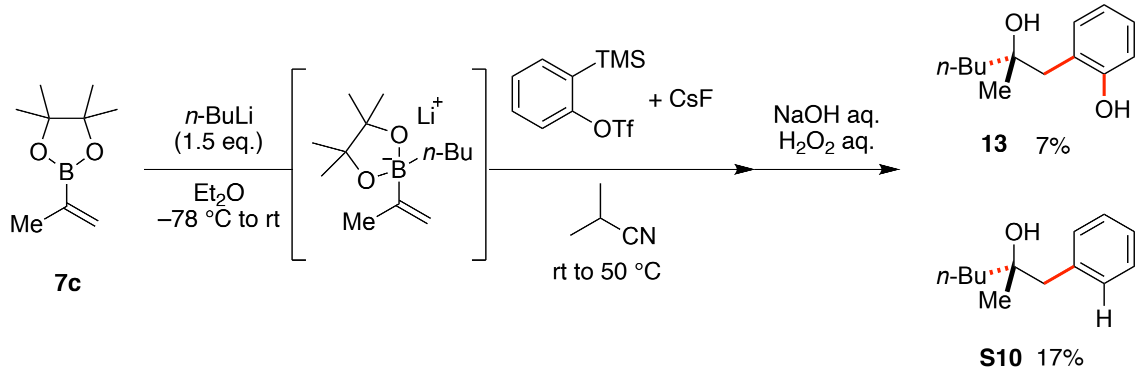

Other conditions for benzyne generation were also investigated. For example, the coupling reaction using a combination of fluorobenzene and butyl lithium was conducted, however, no coupling product was observed.

Scheme S2

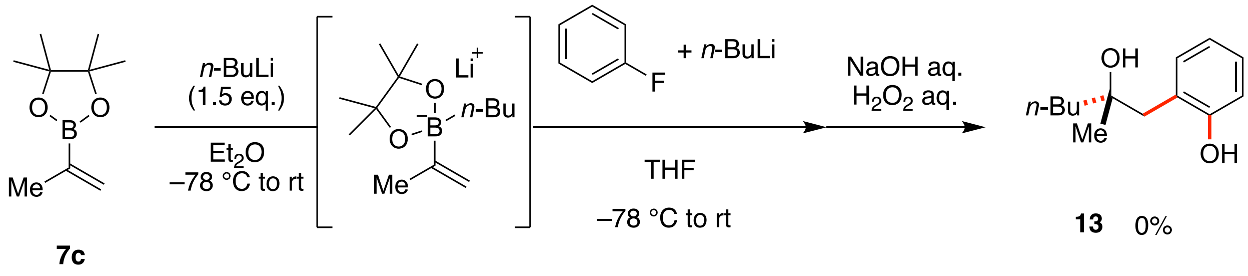

To realize the desired coupling reaction, we assumed that 1) the reaction must be conducted in aprotic and non-nucleophilic solvent, and 2) the temperature for benzyne generation must be relatively low

and controlled, considering the high reactivity of benzyne. With this in mind, we decided to focus on Hosoya's precursor. Because this precursor liberate benzyne through a boron ate-complex intermediate, ethereal solvents can be used. In addition, Hosoya and coworker reported that the activation of the precursor using alkyl lithium can be conducted at low temperature such as –78 °C, and the activated precursor start liberating benzyne above 0 °C.

Optimization of Reaction Conditions

Table S1

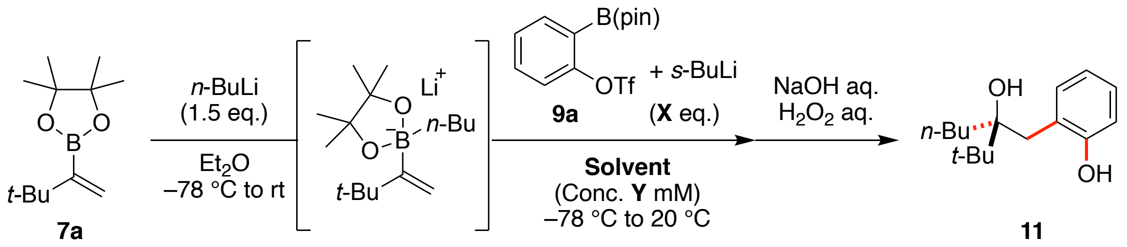

Entry	**Solvent**[1]	**X** equivalent	**Y** mM	Yield[2]
1	THF–Et_2O (1:1)	2.5	25	33
2	CPME–Et_2O (1:1)	2.5	25	7
3	DME–Et_2O (1:1)	2.5	25	10
4	MTHP–Et_2O (1:1)	2.5	25	55
5	MTHP	2.5	25	21
6	MTHP–Et_2O (1:1)	1.5	25	10
7	MTHP–Et_2O (1:1)	2.0	25	22
8	MTHP–Et_2O (1:1)	3.0	25	67 (isolated)
9	MTHP–Et_2O (1:1)	3.5	25	50
10	MTHP–Et_2O (1:1)	3.0	50	41
11	MTHP–Et_2O (1:1)	3.0	12	22

[1] THF = tetrahydrofuran, CPME = cyclopentyl methyl ether, DME = 1,2-dimethoxyethane, MTHP = 4-methyltetrahydropyran

[2] The yield was determined by NMR using 1,1,2-trichloroethylene as an internal standard

Aryne triggered annulative coupling of vinyl boronic ester ate-complexes

General procedure for the aryne triggered annulative coupling (oxidative workup)

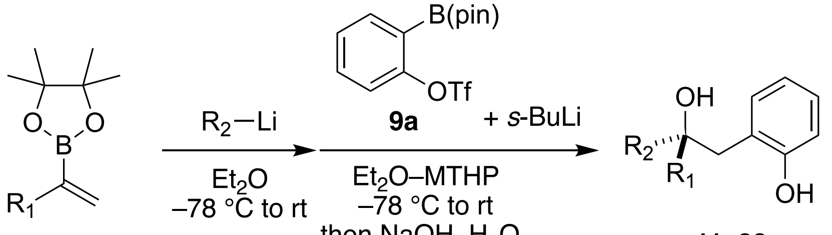

7 11–23

To a solution of vinylboronic ester **7** (0.20 mmol, 1.0 eq.) in Et_2O (2.0 mL) was added *n*-BuLi (2.0 M in hexanes, 0.15 mL, 0.30 mmol, 1.5 eq.) at –78 °C and stirred for 5 min. A cooling bath was removed, and the mixture was further stirred for 30 min. The solvent was removed in vacuo and the flask was back filled with nitrogen. The obtained mixture of vinylboronic ester ate-complex was then dissolved in 4-methyltetrahydropyran (4.0 mL). In the meantime, aryne precursor **9a** (0.6 mmol, 3.0 eq.) was added to a separate flask and dissolved in Et_2O (4.0 mL). The solution was treated with *s*-BuLi (1.2 M in hexanes, 0.5 mL, 0.6 mmol, 3.0 eq.) at –78 °C and stirred for 10 min at this temperature. To this activated aryne precursor solution was then added the solution of vinylboronic ester ate-complex prepared above dropwise at –78 °C. The reaction mixture was stirred at the same temperature for 10 min and a dry ice/acetone bath was exchanged to water bath which temperature was controlled to 20 °C with ice. The reaction mixture was stirred at the same temperature for 2 h. After this period of time, the reaction mixture was cooled to 0 °C and treated with aqueous solution of NaOH (3 M, 5 mL) and aqueous solution of H_2O_2 (30%, 5 mL). This biphasic solution was warmed to rt and stirred vigorously overnight. The flask was then cooled to 0 °C and treated with saturated aqueous solution of NH_4Cl until the pH of the mixture became neutral. The mixture was extracted with Et_2O. The organic layers were washed with brine, dried over Na_2SO_4 and concentrated under reduced pressure. The crude product was purified by column chromatography on silica gel to obtain hydroxyphenol **11**–**23**.

***rac*-2-(2-(*tert*-butyl)-2-hydroxyhexyl)phenol (11)**

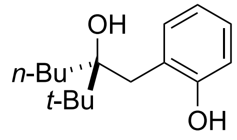

11

Following the general procedure, vinylboronic ester **7a** (42.0 mg, 0.20 mmol) was converted to the corresponding hydroxyphenol. Purification of the crude mixture by flash chromatography gave **11** (33.8 mg, 0.135 mmol, 67%).

For the 1 mmol scale reaction, vinylboronic ester **7a** (210 mg, 1.00 mmol) was converted to **11** (205 mg, 0.820 mmol, 82%) following the same procedure and purification method.

11: yellow solid; IR (film) 3237, 2956, 2868, 1585, 1489, 1244, 1112, 1045, 754 cm^{-1}; ^{1}H NMR (400 MHz, $CDCl_3$) δ 8.74 (s, 1H), 7.13 (app td, *J* = 7.6, 1.6 Hz, 1H), 7.01 (dd, *J* = 7.6, 1.6 Hz, 1H), 6.89 (dd, *J* = 8.0, 1.2 Hz, 1H), 6.81 (app td, *J* = 7.6, 1.2 Hz, 1H), 3.13 (d, *J* = 14.4 Hz, 1H), 2.66 (d, *J* = 14.4 Hz, 1H), 2.08 (br s, 1H), 1.59–1.52 (m, 2H), 1.27–0.95 (m, 4H), 1.05 (s, 9H), 0.74 (t, *J* = 7.2 Hz, 3H); ^{13}C NMR (100 MHz, $CDCl_3$) δ 156.3, 132.4, 128.3, 125.7, 120.1, 117.3, 81.7, 39.3, 38.1, 35.8, 27.3, 25.8, 23.6, 14.0; HRMS (ESI) *m/z* calcd for $C_{16}H_{26}NaO_2$ $[M+Na]^+$ 273.1830, found 273.1823.

***rac*-2-((*R*)-2-(adamantan-1-yl)-2-hydroxypropyl)phenol (12)**

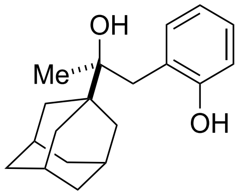

12

Following the general procedure, vinylboronic ester **7b** (58.2 mg, 0.202 mmol) was converted to the corresponding hydroxyphenol. Purification of the crude mixture by flash chromatography gave **12** (45.6 mg, 0.159 mmol, 79%).

12 yellow solid; IR (film) 3244, 2900, 2881, 1683, 1653, 1558, 1506, 1251, 1220, 771 cm^{-1}; ^{1}H NMR (400 MHz, $CDCl_3$) δ 8.70 (br s, 1H), 7.14 (app td, *J* = 7.6, 1.6 Hz, 1H), 6.96 (dd, *J* = 7.6, 1.6 Hz, 1H), 6.91 (dd, *J* = 8.0, 1.2 Hz, 1H), 6.82 (app td, *J* = 7.2, 1.2 Hz, 1H), 3.16 (d, *J* = 14.4 Hz, 1H), 2.47 (d, *J* = 14.4 Hz, 1H), 2.07 (s, 3H), 2.00 (br s, 1H), 1.83–1.55 (m, 12H), 0.99 (s, 3H); ^{13}C NMR (100 MHz, $CDCl_3$) δ 156.0, 132.9, 128.3, 125.5, 120.0, 117.4, 80.3, 39.6, 37.6, 37.1, 36.2, 28.6, 20.7; HRMS (ESI) *m/z* calcd for $C_{19}H_{26}NaO_2$ $[M+Na]^+$ 309.1830, found 309.1835.

rac-2-(2-hydroxy-2-methylhexyl)phenol (13)

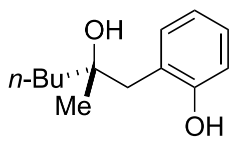

13

Following the general procedure, vinylboronic ester **7c** (0.094 mL, 0.500 mmol) was converted to the corresponding hydroxyphenol. Purification of the crude mixture by flash chromatography gave **13** (29.0 mg, 0.14 mmol, 28%), along with [2+2]-adduct[15] (25.6 mg, 0.191 mmol, 38%)

13: yellow oil; IR (film) 3223, 2931, 2858, 1585, 1489, 1456, 1247, 1126, 754 cm^{-1}; ^{1}H NMR (400 MHz, $CDCl_3$) δ 8.60 (br s, 1H), 7.16 (app td, *J* = 7.6, 1.6 Hz, 1H), 6.98 (dd, *J* = 7.6, 1.6 Hz, 1H), 6.92 (dd, *J* = 8.0, 1.2 Hz, 1H), 6.82 (app td, *J* = 7.4, 1.2 Hz, 1H), 2.91 (d, *J* = 14.8 Hz, 1H), 2.71 (d, *J* = 14.8 Hz, 1H), 2.12 (br s, 1H), 1.59–1.51 (m, 2H), 1.46–1.27 (m, 4H), 1.21 (s, 3H), 0.91 (t, *J* = 6.8 Hz, 3H); ^{13}C NMR (100 MHz, $CDCl_3$) δ 156.0, 132.6, 128.5, 124.7, 120.1, 117.5, 76.6, 44.0, 42.3, 26.9, 26.4, 23.2, 14.2; HRMS (ESI) *m/z* calcd for $C_{13}H_{20}NaO_2$ [M+Na]$^+$ 231.1361, found 231.1357.

rac-2-(2-hydroxy-2-(pentan-3-yl)hexyl)phenol (14)

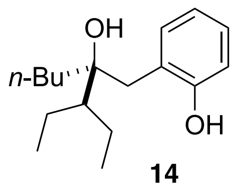

14

Following the general procedure (1.3 eq. of butyl lithium was used), vinylboronic ester **7d** (57.1 mg, 0.255 mmol) was converted to the corresponding hydroxyphenol. Purification of the crude mixture by flash chromatography gave **14** (53.3 mg, 0.202 mmol, 79%).

14: colorless oil; IR (film) 3250, 2959, 2934, 2874, 1586, 1489, 1466, 1379, 1248, 1044, 1003, 851, 754 cm^{-1}; ^{1}H NMR (600 MHz, $CDCl_3$) δ 8.83 (br s, 1H), 7.15 (app td, *J* = 7.8, 1.8 Hz, 1H), 6.97 (dd, *J* = 7.8, 1.8 Hz, 1H), 6.91 (dd, *J* = 7.8, 1.2 Hz, 1H), 6.81 (app td, *J* = 7.2, 1.2 Hz, 1H), 3.05 (d, *J* = 14.4 Hz, 1H), 2.64 (d, *J* = 14.4 Hz, 1H), 2.14 (br s, 1H), 1.76–1.68 (m, 1H), 1.64–1.56 (m, 1H), 1.53–1.47 (m, 1H), 1.43–1.37 (m, 1H), 1.37–1.28 (m, 3H), 1.28–1.19 (m, 4H), 1.00 (t, *J* = 7.2 Hz, 3H) , 0.97 (t, *J* = 7.2 Hz, 3H) , 0.87 (t, *J* = 7.8 Hz, 3H); ^{13}C NMR (150 MHz, $CDCl_3$) δ 156.1, 132.6, 128.3, 124.6, 120.0, 117.3, 81.4, 48.8, 39.8, 36.8, 25.5, 23.3, 22.9, 22.7, 14.2, 13.9; HRMS (ESI) *m/z* calcd for $C_{17}H_{28}NaO_2$ [M+Na]$^+$ 287.1982, found 287.1983.

rac-2-(3-ethyl-2-hydroxy-2-methylpentyl)phenol (15)

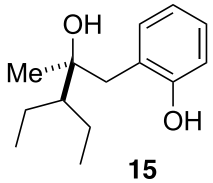

15

Following the general procedure (1.3 eq. of methyl lithium was used), vinylboronic ester **7d** (56.8 mg, 0.253 mmol) was converted to the corresponding hydroxyphenol. Purification of the crude mixture by flash chromatography gave **15** (33.0 mg, 0.148 mmol, 58%).

15: colorless oil; IR (film) 3279, 3013, 2959, 2874, 1586, 1489, 1456, 1379, 1252, 1217, 1117, 851, 764, 750 cm^{-1}; ^{1}H NMR (600 MHz, $CDCl_3$) δ 8.74 (br s, 1H), 7.16 (app td, *J* = 7.8, 1.2 Hz, 1H), 6.98 (dd, *J* = 7.2, 1.8 Hz, 1H), 6.92 (dd, *J* = 8.4, 1.2 Hz, 1H), 6.83 (app td, *J* = 7.2, 1.2 Hz, 1H), 3.04 (d, *J* = 14.4 Hz, 1H), 2.62 (d, *J* = 14.4 Hz, 1H), 2.22 (br s, 1H), 1.74–1.66 (m, 1H), 1.63–1.55 (m, 1H), 1.33 (app hept, *J* = 7.2 Hz, 1H), 1.30–1.22 (m, 2H), 1.12 (s, 3H), 1.01 (t, *J* = 7.8 Hz, 3H), 0.99 (t, *J* = 7.2 Hz, 3H); ^{13}C NMR (150 MHz, $CDCl_3$) δ 156.0, 132.7, 128.4, 124.7, 120.1, 117.5, 79.9, 52.3, 41.7, 24.5, 23.6, 23.0, 14.3, 13.9; HRMS (ESI) *m/z* calcd for $C_{14}H_{22}NaO_2$ [M+Na]$^+$ 245.1512, found 245.1512.

rac-2-(2-cyclohexyl-2-hydroxyhexyl)phenol (16)

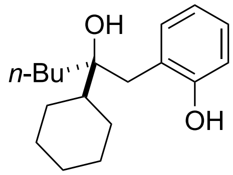

16

Following the general procedure (1.3 eq. of butyl lithium was used), vinylboronic ester **7e** (40.1 mg, 0.168 mmol) was converted to the corresponding hydroxyphenol. Purification of the crude mixture by flash chromatography gave **16** (25.7 mg, 0.0931 mmol, 55%).

16: yellow oil; IR (film) 3408, 3208, 2931, 2852, 1585, 1489, 1452, 1246, 1120, 1001, 752 cm^{-1}; ^{1}H NMR (400 MHz, $CDCl_3$) δ 7.15 (app td, *J* = 7.6, 1.6 Hz, 1H), 6.97 (dd, *J* = 7.6, 1.6 Hz, 1H), 6.92 (dd, *J* = 8.0, 0.8 Hz, 1H), 6.82 (app td, *J* = 7.6, 1.2 Hz, 1H), 3.02 (d, *J* = 14.4 Hz, 1H), 2.63 (d, *J* = 14.8 Hz, 1H), 1.93–1.54 (m, 5H), 1.52–1.03 (m, 12H), 0.86 (t, *J* = 7.2 Hz, 3H); ^{13}C NMR (100 MHz, $CDCl_3$) δ 156.0, 132.6, 128.3, 124.7, 120.0, 117.3, 80.0, 45.1, 39.3, 36.2, 27.0, 26.8, 26.5, 25.4, 23.3, 14.2; HRMS (ESI) *m/z* calcd for $C_{18}H_{28}NaO_2$ [M+Na]$^+$ 299.1987, found 299.1983.

***rac*-2-(2-cyclohexyl-2-hydroxypropyl)phenol (17)**

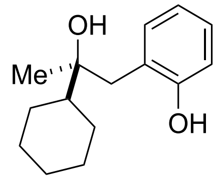

17

Following the general procedure (1.3 eq. of methyl lithium was used), vinylboronic ester **7e** (59.3 mg, 0.251 mmol) was converted to the corresponding hydroxyphenol. Purification of the crude mixture by flash chromatography gave **17** (34.2 mg, 0.146 mmol, 58%).

17: yellow solid; IR (film) 3395, 3200, 2972, 2852, 1585, 1489, 1452, 1379, 1247, 1118, 752 cm^{-1}; ^{1}H NMR (400 MHz, $CDCl_3$) δ 7.15 (app td, *J* = 7.6, 1.6 Hz, 1H), 6.97 (dd, *J* = 7.6, 1.6 Hz, 1H), 6.90 (dd, *J* = 8.0, 1.2 Hz, 1H), 6.80 (app td, *J* = 7.4, 1.2 Hz, 1H), 2.98 (d, *J* = 14.4 Hz, 1H), 2.65 (d, *J* = 14.8 Hz, 1H), 1.94 (br d, *J* = 12.8 Hz, 1H), 1.99–1.62 (m, 3H), 1.69 (br d, *J* = 10.4 Hz, 1H), 1.42 (tt, *J* = 11.6, 2.8 Hz, 1H), 1.32–0.99 (m, 5H), 1.11 (s, 3H); ^{13}C NMR (100 MHz, $CDCl_3$) δ 156.1, 132.6, 128.4, 124.7, 120.0, 117.5, 78.7, 47.9, 41.5, 28.1, 27.0, 26.63, 26.59, 26.5, 24.0; HRMS (ESI) *m/z* calcd for $C_{15}H_{22}NaO_2$ $[M+Na]^+$ 257.1517, found 257.1524.

***rac*-2-(2-cyclopropyl-2-hydroxyhexyl)phenol (18)**

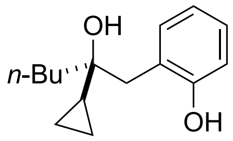

18

Following the general procedure (1.3 eq. of butyl lithium was used), vinylboronic ester **7f** (48.5 mg, 0.250 mmol) was converted to the corresponding hydroxyphenol. Purification of the crude mixture by flash chromatography gave **18** (36.2 mg, 0.155 mmol, 62%).

18: yellow solid; IR (film) 3235, 3082, 2958, 2870, 1585, 1489, 1456, 1246, 1116, 1022, 752 cm^{-1}; ^{1}H NMR (400 MHz, $CDCl_3$) δ 8.44 (br s, 1H), 7.14 (app td, *J* = 7.6, 1.6 Hz, 1H), 7.02 (dd, *J* = 7.6, 1.6 Hz, 1H), 6.90 (dd, *J* = 8.0, 1.2 Hz, 1H), 6.82 (app td, *J* = 7.2, 1.2 Hz, 1H), 2.90 (d, *J* = 14.8 Hz, 1H), 2.82 (d, *J* = 14.8 Hz, 1H), 1.81 (br s, 1H), 1.57–1.22 (m, 6H), 0.99–0.91 (m, 1H), 0.89 (t, *J* = 7.4 Hz, 3H), 0.46–0.37 (m, 2H), 0.37–0.24 (m, 2H); ^{13}C NMR (100 MHz, $CDCl_3$) δ 155.9, 132.6, 128.3, 124.7, 120.1, 117.3, 76.2, 42.7, 40.2, 26.0, 23.3, 19.5, 14.2, 0.48, 0.33; HRMS (ESI) *m/z* calcd for $C_{15}H_{22}NaO_2$ $[M+Na]^+$ 257.1517, found 257.1513.

***rac*-2-(2-hydroxy-2-(naphthalen-2-yl)hexyl)phenol (19)**

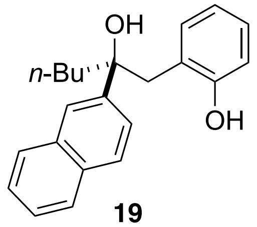

19

Following the general procedure (1.3 eq. of butyl lithium was used), vinylboronic ester **7g** (71.3 mg, 0.254 mmol) was converted to the corresponding hydroxyphenol. Purification of the crude mixture by flash chromatography gave **19** (47.1 mg, 0.147 mmol, 58%).

19: yellow solid; IR (film) 3295, 3013, 2957, 2934, 2866, 1586, 1489, 1456, 1379, 1244, 1215, 1126, 1044, 945, 854, 820, 752, 667 cm^{-1}; ^{1}H NMR (600 MHz, $CDCl_3$) δ 8.29 (br s, 1H), 7.86 (d, *J* = 1.8 Hz, 1H), 7.85–7.79 (m, 3H), 7.51–7.45 (m, 3H), 7.10 (app td, *J* = 7.8, 1.8 Hz, 1H), 6.92 (dd, *J* = 7.8, 1.2 Hz, 1H), 6.82 (dd, *J* = 7.2, 1.8 Hz, 1H), 6.70 (app td, *J* = 7.2, 1.2 Hz, 1H), 3.18 (ABq, *J* = 15.0 Hz, 2H), 2.91 (br s, 1H), 2.14 (ddd, *J* = 14.4, 12.6, 4.2 Hz, 1H), 1.92 (ddd, *J* = 13.8, 12.0, 4.2 Hz, 1H), 1.31–1.22 (m, 2H), 1.18–1.09 (m, 1H), 1.07–0.99 (m, 1H), 0.80 (t, *J* = 7.2 Hz, 3H); ^{13}C NMR (150 MHz, $CDCl_3$) δ 155.8, 142.6, 133.3, 132.8, 132.4, 128.5, 128.3, 128.3, 127.6, 126.4, 126.1, 124.0, 123.9, 123.5, 120.3, 117.4, 80.8, 46.4, 40.2, 25.7, 23.0, 14.1; HRMS (ESI) *m/z* calcd for $C_{22}H_{24}NaO_2$ $[M+Na]^+$ 343.1669, found 343.1665.

***rac*-2-(2-hydroxy-2-(naphthalen-2-yl)propyl)phenol (20)**

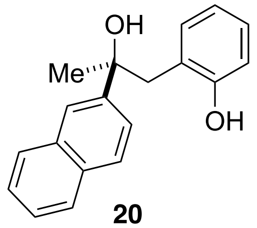

20

Following the general procedure, vinylboronic ester **7g** (70.6 mg, 0.252 mmol) was converted to the corresponding hydroxyphenol. Purification of the crude mixture by flash chromatography gave **20** (49.4 mg, 0.177 mmol, 70%).

20: yellow solid; IR (film) 3280, 3055, 2966, 1597, 1481, 1458, 1375, 1246, 1097, 1016, 856, 817, 750 cm^{-1}; ^{1}H NMR (400 MHz, $CDCl_3$) δ 7.94 (d, *J* = 5.0 Hz, 1H), 7.90–7.78 (m, 3H), 7.60 (dd, *J* = 8.8, 1.6 Hz, 1H), 7.53–7.44 (m, 2H), 7.14 (app td, *J* = 7.6, 1.6 Hz, 1H), 6.96 (dd, *J* = 8.0, 1.2 Hz, 1H), 6.88 (dd, *J* = 7.6, 1.6 Hz, 1H), 6.76 (app td, *J* = 7.2, 1.2 Hz, 1H), 3.29 (d, *J* = 14.8 Hz, 1H), 2.99 (d, *J* = 14.8 Hz, 1H), 1.72 (s, 3H); ^{13}C NMR (100 MHz, $CDCl_3$) δ 155.9, 144.6, 133.2, 132.8, 132.6, 128.7, 128.3, 127.7, 126.4, 126.2, 124.3, 123.4, 123.1, 120.3, 117.6, 78.2, 46.8, 28.5; HRMS (ESI) *m/z* calcd for $C_{19}H_{18}NaO_2$ $[M+Na]^+$ 301.1204, found 301.1198.

rac-2-(2-(4-fluorophenyl)-2-hydroxypropyl)phenol (21)

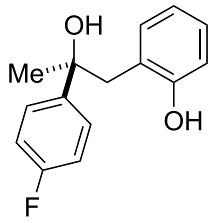

21

Following the general procedure, vinylboronic ester **7j** (62.4 mg, 0.252 mmol) was converted to the corresponding hydroxyphenol. Purification of the crude mixture by flash chromatography gave **21** (43.0 mg, 0.175 mmol, 69%).

21: colorless oil; IR (film) 3379, 3019, 1604, 1510, 1489, 1215, 1161, 930, 837, 768, 745, 669 cm^{-1}; ^{1}H NMR (600 MHz, $CDCl_3$) δ 8.30 (br s, 1H), 7.45–7.40 (m, 2H), 7.14 (ddd, *J* = 7.8, 7.2, 1.8 Hz, 1H), 7.04–7.00 (m, 2H), 6.92 (dd, *J* = 7.8, 1.2 Hz, 1H), 6.81 (dd, *J* = 7.2, 1.8 Hz, 1H), 6.77 (td, *J* = 7.2, 1.2 Hz, 1H), 3.12 (d, *J* = 14.4 Hz, 1H), 2.91 (d, *J* = 14.4 Hz, 1H), 2.81 (br s, 1H), 1.72 (s, 3H); ^{13}C NMR (150 MHz, $CDCl_3$) δ 162.0 (d, *J* = 244.2 Hz), 155.7, 142.9 (d, *J* = 2.4 Hz), 132.8, 128.7, 126.5 (d, *J* = 8.3 Hz), 124.2, 120.4, 117.5, 115.2 (d, *J* = 21.2 Hz), 77.7, 47.1, 28.6; HRMS (ESI) *m/z* calcd for $C_{15}H_{15}FNaO_2$ [M+Na]$^+$ 269.0954, found 269.0921.

rac-2-(7-((_tert_-butyldiphenylsilyl)oxy)-2-hydroxy-2,3,3-trimethylheptyl)phenol (22)

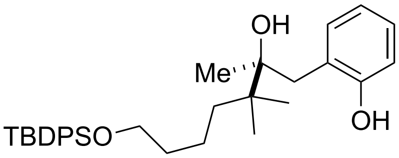

22

Following the general procedure (1.3 eq. of methyl lithium was used), vinylboronic ester **7h** (116 mg, 0.229 mmol) was converted to the corresponding hydroxyphenol. Purification of the crude mixture by flash chromatography gave **22** (64.6 mg, 0.128 mmol, 56%).

22: White solid; IR (film) 3360, 3015, 2945, 2861, 1586, 1489, 1474, 1427, 1379, 1248, 1215, 1103, 754, 702, 669 cm^{-1}; ^{1}H NMR (600 MHz, $CDCl_3$) δ 8.66 (br s, 1H), 7.70–7.67 (m, 4H), 7.45–7.37 (m, 6H), 7.16 (td, *J* = 7.8, 1.2 Hz, 1H), 6.97 (d, *J* = 7.2 Hz, 1H), 6.95–6.91 (m, 1H), 6.85–6.82 (m, 1H), 3.71 (t, *J* = 6.0 Hz, 2H), 3.18 (d, *J* = 14.4 Hz, 1H), 2.50 (d, *J* = 14.4 Hz, 1H), 2.01 (br s, 1H), 1.62–1.55 (m, 2H), 1.49–1.37 (m, 3H), 1.36–1.30 (m, 1H), 1.07 (s, 9H), 1.03 (s, 3H), 0.99 (s, 3H), 0.98 (s, 3H); ^{13}C NMR (150 MHz, $CDCl_3$) δ 156.0, 135.7, 134.2, 132.8, 129.8, 129.7, 128.3, 127.8, 125.4, 120.1, 117.5, 115.4, 81.2, 63.9, 40.9, 38.5, 36.5, 33.7, 27.0, 21.8, 21.5, 21.3, 21.1, 19.4; HRMS (ESI) *m/z* calcd for $C_{32}H_{44}NaO_3Si$ [M+Na]$^+$ 527.2952, found 527.2953.

tert-butyl _rac_-4-(2-hydroxy-1-(2-hydroxyphenyl)propan-2-yl)piperidine-1-carboxylate (23)

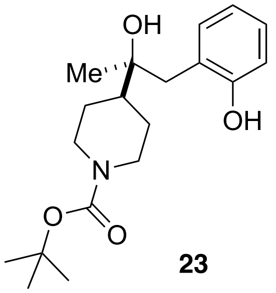

23

Following the general procedure (1.3 eq. of methyl lithium was used), vinylboronic ester **7i** (84.3 mg, 0.250 mmol) was converted to the corresponding hydroxyphenol. Purification of the crude mixture by flash chromatography gave **23** (44.2 mg, 0.113 mmol, 45%).

23: White solid; IR (film) 3354, 3019, 2976, 1665, 1489, 1431, 1368, 1244, 1215, 1163, 1034, 928, 758, 669 cm^{-1}; ^{1}H NMR (600 MHz, $CDCl_3$) δ 8.87 (br s, 1H), 7.14 (app td, *J* = 7.8, 1.2 Hz, 1H), 6.96 (dd, *J* = 7.2, 1.2 Hz, 1H), 6.90 (dd, *J* = 7.8, 1.2 Hz, 1H), 6.82 (app td, *J* = 7.2, 1.2 Hz, 1H), 4.18 (br s, 2H), 3.11 (br s, 1H), 2.99 (br d, *J* = 14.4 Hz, 1H), 2.71–2.51 (m, 2H), 2.60 (br d, *J* = 14.4 Hz, 1H), 1.84 (br dt, *J* = 13.2, 2.4 Hz, 1H), 1.77 (br dt, *J* = 13.2, 1.8 Hz, 1H), 1.56 (tt, *J* = 12.0, 3.0 Hz, 1H), 1.45 (s, 9H), 1.36–1.25 (m, 2H), 1.11 (s, 3H); ^{13}C NMR (150 MHz, $CDCl_3$) δ 155.9, 155.0, 132.6, 128.5, 124.4, 120.1, 117.4, 79.8, 77.4, 46.5. 44.6 (br), 43.8 (br), 41.6, 28.6, 27.1 (br), 26.3, 23.7; HRMS (ESI) *m/z* calcd for $C_{19}H_{29}NNaO_4$ $[M+Na]^+$ 358.1989, found 358.1988.

General procedure for the aryne triggered annulative coupling (oxidative workup) using substituted aryne precursors

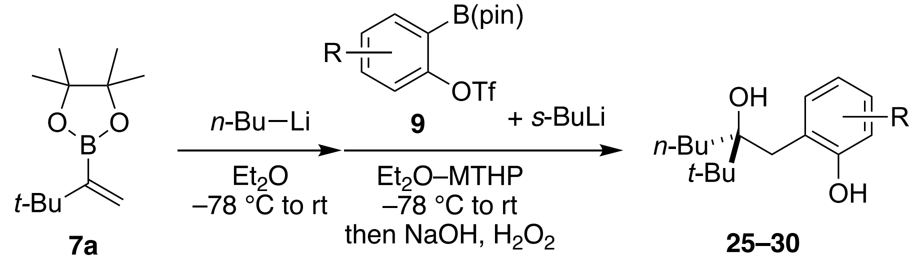

To a solution of vinylboronic ester **7a** (0.20 mmol, 1.0 eq.) in Et_2O (2.0 mL) was added *n*-BuLi (2.0 M in hexanes, 0.15 mL, 0.30 mmol, 1.5 eq.) at –78 °C and stirred for 5 min. A cooling bath was removed, and the mixture was further stirred for 30 min. The solvent was removed in vacuo and the flask was back filled with nitrogen. The obtained mixture of vinylboronic ester ate-complex was then dissolved in 4-methyltetrahydropyran (4.0 mL). In the meantime, aryne precursor **9** (0.6 mmol, 3.0 eq.) was added to a separate flask and dissolved in Et_2O (4.0 mL). The solution was treated with *s*-BuLi (1.2 M in hexanes, 0.5 mL, 0.6 mmol, 3.0 eq.) at –78 °C and stirred for 10 min at this temperature. To this activated aryne precursor solution was then added the solution of vinylboronic ester ate-complex prepared above dropwise at –78 °C. The reaction mixture was stirred at the same temperature for 10 min and a dry ice/acetone bath was exchanged to water bath which temperature was controlled to 20 °C with ice. The reaction mixture was stirred at the same temperature for 2 h. After this period of time, the reaction mixture was cooled to 0 °C and treated with aqueous solution of NaOH (3 M, 5 mL) and aqueous solution of H_2O_2 (30%, 5 mL). This biphasic solution was warmed to rt and stirred vigorously overnight. The flask was then cooled to 0 °C and treated with saturated aqueous solution of NH_4Cl until the pH of the mixture became neutral. The mixture was extracted with Et_2O. The organic layers were washed with brine, dried over Na_2SO_4 and concentrated under reduced pressure. The crude product was purified by column chromatography on silica gel to obtain hydroxyphenol **25**-**30**.

rac-2-(2-(_tert_-butyl)-2-hydroxyhexyl)-5-methoxyphenol (25a) and _rac_-2-(2-(_tert_-butyl)-2-hydroxyhexyl)-4-methoxyphenol (25b)

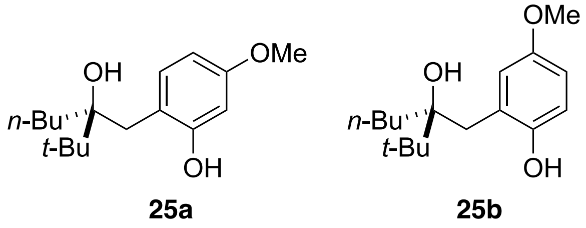

Following the general procedure, vinylboronic ester **7a** (42.0 mg, 0.200 mmol) was coupled with aryne precursor **9b** to give corresponding hydroxyphenol. Purification of the crude mixture by flash chromatography gave **25** (23.6 mg, 0.0842 mmol, 42%, 1:1 mixture of inseparable regioisomers **25a** and **25b**).

25a + **25b**: colorless oil; IR (film) 3383, 2957, 2893, 1989, 1624, 1499, 1223 cm^{-1}; ^{1}H NMR (600 MHz, $CDCl_3$) δ 6.88 (d, *J* = 8.4, 1H), 6.82 (d, *J* = 8.7 Hz, 1H), 6.70 (dd, *J* = 9.0, 3.0 Hz, 1H), 6.59 (d, *J* = 3.0 Hz, 1H), 6.48 (d, *J* = 2.6 Hz, 1H), 6.39 (dd, *J* = 8.4, 2.6 Hz, 1H), 3.77 (s, 3H), 3.75 (s, 3H), 3.13 (d, *J* = 14.4 Hz, 1H), 3.05 (d, *J* = 14.4 Hz, 1H), 2.61 (d, *J* = 14.4 Hz, 1H), 2.60 (d, *J* = 14.4 Hz, 1H), 1.58–1.50 (m, 4H), 1.23–1.16 (m, 2H), 1.17–0.99 (m, 6H), 1.05 (s, 9H), 1.04 (s, 9H), 0.77 (t, *J* = 7.2 Hz, 3H), 0.76 (t, *J* = 7.2 Hz, 3H); ^{13}C NMR (150 MHz, $CDCl_3$) δ 160.0, 157.2, 153.2, 150.3, 132.8, 126.6, 118.0, 117.72, 117.71, 113.2, 106.1, 102.7, 81.7(2C), 56.0, 55.4, 39.3, 39.2, 38.1, 37.4, 35.83, 35.80, 27.38, 27.35, 25.9, 25.8, 23.7(2C), 14.0(2C); HRMS (ESI) *m/z* calcd for $C_{17}H_{28}NaO_3$ $[M+Na]^+$ 303.1931, found 303.1922.

rac-2-(2-(_tert_-butyl)-2-hydroxyhexyl)-5-methylphenol (26a) and _rac_-2-(2-(_tert_-butyl)-2-hydroxyhexyl)-4-methylphenol (26b)

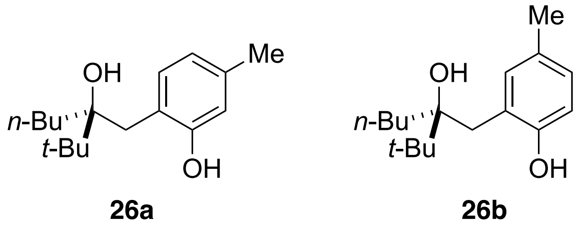

Following the general procedure, vinylboronic ester **7a** (42.4 mg, 0.202 mmol) was coupled with aryne precursor **9c** to give corresponding hydroxyphenol. Purification of the crude mixture by flash chromatography gave **26** (31.9 mg, 0.121 mmol, 60%, 5:4 mixture of inseparable regioisomers **26a** and **26b**).

26a + **26b**: colorless oil; IR (film) 3391, 3250, 2957, 2872, 1626, 1577, 1504, 1251 cm^{-1}; ^{1}H NMR (400 MHz, $CDCl_3$) δ 8.61 (br s, 1H), 8.47 (br s, 1H), 6.93 (dd, *J* = 8.0, 2.0 Hz, 1H), 6.88 (d, *J* = 7.6 Hz, 1H), 6.82 (d, *J* = 1.8 Hz, 1H), 6.78 (d, *J* = 8.1 Hz, 1H), 6.72 (br s, 1H), 6.62 (dd, *J* = 7.5, 1.2 Hz, 1H), 3.11 (d, *J* = 14.4 Hz, 1H), 3.08 (d, *J* = 14.4 Hz, 1H), 2.63 (d, *J* = 14.4 Hz, 1H), 2.60 (d, *J* = 14.4 Hz, 1H), 2.28 (s, 3H), 2.24 (s, 3H),

2.01 (br s, 2H), 1.57–1.50 (m, 4H), 1.26–0.99 (m, 8H), 1.05 (s, 9H), 1.05 (s, 9H), 0.77 (t, *J* = 7.0 Hz, 3H), 0.75 (t, *J* = 7.0 Hz, 3H); ^{13}C NMR (150 MHz, $CDCl_3$) δ 156.1, 154.0, 138.2, 133.0, 132.2, 129.1, 128.7, 125.4, 122.5, 120.9, 117.9, 117.0, 81.69, 81.66, 39.31, 39.27, 38.0, 37.7, 35.9, 35.7, 27.4, 27.3, 25.9, 25.8, 23.7, 23.6, 21.2, 20.6, 14.0, 13.9; HRMS (ESI) *m/z* calcd for $C_{17}H_{28}NaO_2$ $[M+Na]^+$ 287.1982, found 287.1977.

rac-2-(2-(_tert_-butyl)-2-hydroxyhexyl)-5-fluorophenol (27a) and _rac_-2-(2-(_tert_-butyl)-2-hydroxyhexyl)-4-fluorophenol (27b)

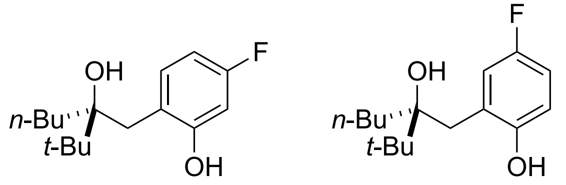

27a **27b**

Following the general procedure, vinylboronic ester **7a** (53.5 mg, 0.255 mmol) was coupled with aryne precursor **9d** to give corresponding hydroxyphenol. Purification of the crude mixture by flash chromatography gave **27** (42.8 mg, 0.159 mmol, 63%, 3:1 mixture of regioisomers **27a** and **27b**). These regioisomers were able to separate to obtain pure **27a** and **27b** after multiple column chromatography.

27a: pale yellow oil; IR (film) 3187, 2957, 2926, 2355, 1493, 1458, 1242 cm^{-1}; ^{1}H NMR (400 MHz, $CDCl_3$) δ 9.02 (br, 1H), 6.93 (dd, *J* = 8.4, 6.8 Hz, 1H), 6.61 (dd, *J* = 10.4, 2.4 Hz, 1H), 6.52 (app td, *J* = 8.4, 2.4 Hz, 1H), 3.07 (d, *J* = 14.8 Hz, 1H), 2.64 (d, *J* = 14.8 Hz, 1H), 2.06 (br s, 1H), 1.58–1.51 (m, 2H), 1.22–0.98 (m, 4H), 1.04 (s, 9H), 0.76 (t, *J* = 7.2 Hz, 3H); ^{13}C NMR (100 MHz, $CDCl_3$) δ 162.9 (d, *J* = 243 Hz), 157.7 (d, *J* = 12 Hz), 132.8 (d, *J* = 9.0 Hz), 121.4 (d, *J* = 3.0 Hz), 106.7 (d, *J* = 21 Hz), 104.7 (d, *J* = 24 Hz), 81.8 (d, *J* = 1.0 Hz), 39.3, 37.7, 35.7, 27.3, 25.8, 23.6, 14.0; HRMS (ESI) *m/z* calcd for $C_{16}H_{25}FNaO_2$ $[M+Na]^+$ 291.1731, found 291.1729.

27b: pale yellow oil; IR (film) 3129, 2961, 2903, 1603, 1506, 1468, 1370 cm^{-1}; ^{1}H NMR (400 MHz, $CDCl_3$) δ 8.54 (br, 1H), 6.84–6.80 (m, 2H), 6.76–6.71 (m, 1H), 3.12 (d, *J* = 14.4 Hz, 1H), 2.60 (d, *J* = 14.4 Hz, 1H), 2.02 (br, 1H), 1.62–1.48 (m, 2H), 1.23–0.98 (m, 4H), 1.05 (s, 9H), 0.77 (t, *J* = 7.2 Hz, 3H). ^{13}C NMR (100 MHz, $CDCl_3$) δ 156.6 (d, *J* = 236 Hz), 152.4 (d, *J* = 2.0 Hz), 127.1 (d, *J* = 7.0 Hz), 118.3 (d, *J* = 22 Hz), 118.0 (d, *J* = 9.0 Hz), 114.4 (d, *J* = 22 Hz), 81.9, 39.4, 38.2, 35.7, 27.3, 25.8, 23.6, 13.9; HRMS (ESI) *m/z* calcd for $C_{16}H_{25}FNaO_2$ $[M+Na]^+$ 291.1731, found 291.1723.

***rac*-2-(2-(*tert*-butyl)-2-hydroxyhexyl)-6-methoxyphenyl acetate (S1)**

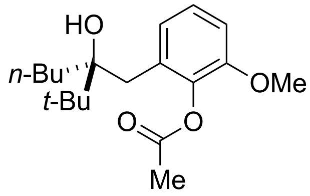

S1 (acetylated **28**)

Following the general procedure, vinylboronic ester **7a** (42.0 mg, 0.192 mmol) was coupled with aryne precursor **9e** to give corresponding hydroxyphenol. Purification of the crude mixture by flash chromatography gave **28**. Because **28** was not able to separate from unknown byproducts, it was converted to acetate derivative (Ac_2O, DMAP, pyridine). Purification of the crude mixture by flash chromatography gave **S1** (11.3 mg, 0.035 mmol, 18% in 2 steps).

S1: white solid; IR (film) 3568, 2957, 2872, 1773, 1734, 1478, 1437 cm^{-1}; ^{1}H NMR (600 MHz, $CDCl_3$) δ 7.13 (app t, *J* = 8.4 Hz, 1H), 6.87–6.84 (m, 2H), 3.81 (s, 3H), 2.91 (d, *J* = 13.2 Hz, 1H), 2.67 (d, *J* = 13.2 Hz, 1H), 2.32 (s, 3H), 1.53–1.48 (m, 2H), 1.29–1.12 (m, 3H), 1.04–0.96 (m, 1H), 1.02 (s, 9H), 0.81 (t, *J* = 7.2 Hz, 3H); ^{13}C NMR (150 MHz, $CDCl_3$) δ 169.0, 151.4, 139.4, 132.3, 126.1, 124.3, 110.5, 77.7, 56.1, 39.1, 29.9, 27.8, 25.9, 23.8, 20.7, 14.2; HRMS (ESI) *m/z* calcd for $C_{19}H_{30}NaO_4$ [M+Na]$^+$ 345.2036, found 345.1970.

***rac*-3-(2-(*tert*-butyl)-2-hydroxyhexyl)naphthalen-2-ol (30)**

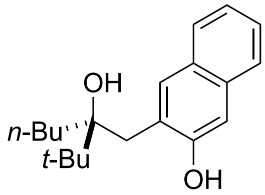

30

Following the general procedure, vinylboronic ester **7a** (45.2 mg, 0.215 mmol) was coupled with aryne precursor **9g** to give corresponding hydroxyphenol. Purification of the crude mixture by flash chromatography gave **30** (12.8 mg, 0.039 mmol, 18%).

30: colorless oil; IR (film) 3471, 2959, 2872, 2490, 1956, 1458, 1260 cm^{-1}; ^{1}H NMR (600 MHz, $CDCl_3$) δ 8.93 (br s, 1H), 7.68 (app d, *J* = 9.0 Hz, 2H), 7.51 (s, 1H), 7.37 (ddd, *J* = 8.4, 6.6, 1.2 Hz, 1H), 7.29–7.26 (m, 2H), 3.29 (d, *J* = 14.4 Hz, 1H), 2.89 (d, *J* = 14.4 Hz, 1H), 2.14 (br s, 1H), 1.64–1.54 (m, 2H), 1.37–1.20 (m, 2H), 1.09 (s, 9H), 1.07–1.00 (m, 2H), 0.68 (t, *J* = 7.3 Hz, 3H); ^{13}C NMR (150 MHz, $CDCl_3$) δ 154.7, 134.4, 131.3, 128.8, 128.6, 127.1, 126.3, 125.8, 123.3, 111.8, 81.5, 39.5, 38.6, 35.8, 27.2, 25.9, 23.5, 13.9; HRMS (ESI) *m/z* calcd for $C_{20}H_{28}NaO_2$ [M+Na]$^+$ 323.1982, found 323.1939.

Procedure for the aryne triggered annulative coupling using vinyltin as a substrate (oxidative workup)

***rac*-2-(2-hydroxy-2-isopropyl-4-phenylbutyl)phenol (33)**

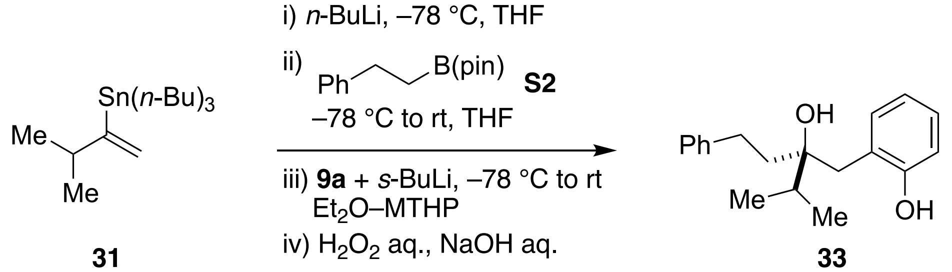

31 **33**

To a solution of vinyltin **31** (0.208 mmol, 1.0 eq.) in THF (1.0 mL) was added *n*-BuLi (2.5 M in hexanes, 0.10 mL, 0.24 mmol, 1.2 eq.) at –78 °C and stirred for 30 min. A cooling bath was then removed, and the mixture was further stirred at 0 °C for 10 min. Resulting vinyllithium solution was cooled again to –78 °C and a THF solution (1.5 mL) of boronic ester **S2** (60.4 mg, 0.26 mmol, 1.3 eq.) was added dropwise. After being stirred at the same temperature for 5 min, the cooling bath was removed, and the mixture was stirred at rt for 45 min. Then, the solvent was removed in vacuo and the flask was back filled with nitrogen. The obtained mixture of vinylboronic ester ate-complex was then dissolved in 4-methyltetrahydropyran (2.0 mL). In the meantime, aryne precursor **9a** (0.60 mmol, 3.0 eq.) was added to a separate flask and dissolved in Et_2O (4.0 mL). The solution was treated with *s*-BuLi (1.06 M in hexanes, 0.57 mL, 0.60 mmol, 3.0 eq.) at –78 °C and being stirred for 10 min at this temperature. To this activated aryne precursor solution was then added the solution of vinylboronic ester ate-complex prepared above dropwise at –78 °C. The reaction mixture was stirred at the same temperature for 10 min and a dry ice/acetone bath was exchanged to water bath which temperature was controlled to 20 °C with ice. The reaction mixture was stirred at the same temperature for 2 h. After this period of time, the reaction mixture was cooled to 0 °C and treated with aqueous solution of NaOH (3 M, 10.0 mL) and aqueous solution of H_2O_2 (30%, 5.0 mL). This biphasic solution was warmed to rt and stirred vigorously overnight. The flask was then cooled to 0 °C and treated with aqueous solution of HCl (1 M) until the pH of the mixture became slightly acidic. The mixture was extracted with Et_2O. The organic layers were washed with brine, dried over Na_2SO_4 and concentrated under reduced pressure. The crude product was purified by column chromatography on silica gel to obtain hydroxyphenol **33** (37.2 mg, 0.126 mmol, 61%).

33: Yellow solid; IR (film) 3414, 2922, 1585, 1489, 1456, 1250, 1217, 758 cm^{-1}; ^{1}H NMR (600 MHz, $CDCl_3$) δ 8.71 (br s, 1H), 7.25 (app t, *J* = 7.8 Hz, 2H), 7.17 (app tt, *J* = 7.8, 1.8 Hz, 2H), 7.09–7.07 (m, 2H), 7.06 (dd, *J* = 7.8, 1.8 Hz, 1H), 6.93 (dd, *J* = 8.4, 1.2 Hz, 1H), 6.84 (app td, *J* = 7.2, 1.2 Hz, 1H), 3.07 (d, *J* = 15.0 Hz, 1H), 2.78 (d, *J* = 15.0 Hz, 1H), 2.73 (ddd, *J* = 13.8, 12.6, 5.4 Hz, 1H), 2.60 (ddd, *J* = 12.6, 12.0, 6.0 Hz, 1H), 2.06 (br s, 1H), 1.98 (septet, *J* = 7.2 Hz, 1H), 1.77 (ddd, *J* = 13.8, 12.0, 4.8 Hz, 1H), 1.75 (ddd, *J* = 13.8, 12.0, 6.0 Hz, 1H), 1.04 (d, *J* = 7.2 Hz, 6H); ^{13}C NMR (150 MHz, $CDCl_3$) δ156.0, 141.7, 132.5, 128.5, 128.4, 128.3, 126.1, 124.0, 119.9, 117.3, 79.9, 39.1, 38.2, 34.7, 29.7, 16.9, 16.7; HRMS (ESI) *m/z* calcd for $C_{22}H_{30}NaO_2$ $[M+Na]^+$ 307.1669, found 307.1663.

General procedure for the aryne triggered annulative coupling using vinylbromide as a substrate (oxidative workup)

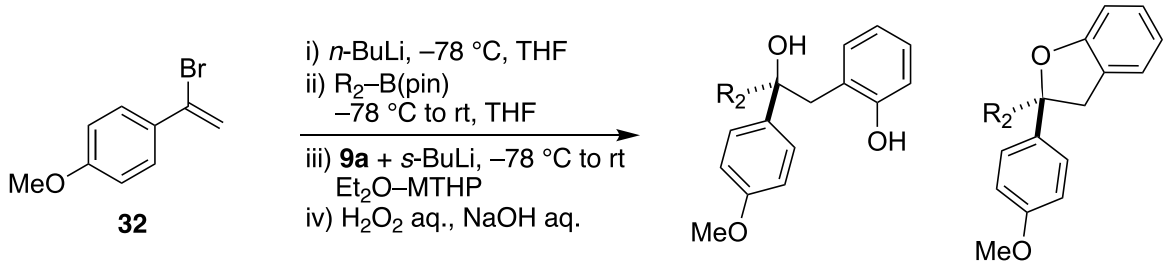

34-36

A solution of vinylbromide **32** (0.30 mmol, 1.0 eq.) in THF–Et_2O (1:1, 2.0 mL) was cooled to –78 °C and treated with *n*-BuLi (1.5 M in hexanes, 0.22 mL, 0.33 mmol, 1.1 eq.). After being stirred for 30 min at same temperature, resulting vinyllithium solution was added a THF solution (1.5 mL) of boronic ester (0.39 mmol, 1.3 eq.) dropwise. After being stirred at the same temperature for 5 min, the cooling bath was removed, and the mixture was stirred at rt for 1 h. Then, the solvent was removed in vacuo and the flask was back filled with nitrogen. The obtained mixture of vinylboronic ester ate-complex was then dissolved in 4-methyltetrahydropyran (3.0 mL). In the meantime, aryne precursor **9a** (0.90 mmol, 3.0 eq.) was added to a separate flask and dissolved in Et_2O (6.0 mL). The solution was treated with *s*-BuLi (1.23 M in hexanes, 0.73 mL, 0.90 mmol, 3.0 eq.) at –78 °C and being stirred for 10 min at this temperature. To this activated aryne precursor solution was then added the solution of vinylboronic ester ate-complex prepared above dropwise at –78 °C. The reaction mixture was stirred at the same temperature for 10 min and a dry ice/acetone bath was exchanged to water bath which temperature was controlled to 20 °C with ice. The reaction mixture was stirred at the same temperature for 2 h. After this period of time, the reaction mixture was cooled to 0 °C and treated with aqueous solution of NaOH (3 M, 10.0 mL) and aqueous solution of H_2O_2 (30%, 5.0 mL). This biphasic solution was warmed to rt and stirred vigorously overnight. The flask was then cooled to 0 °C and treated with saturate aqueous solution of NH_4Cl until the pH of the mixture became slightly acidic. The mixture was extracted with Et_2O. The organic layers were washed with brine, dried over Na_2SO_4 and concentrated under reduced pressure. The crude product was purified by column chromatography on silica gel to obtain hydroxyphenol **34**-**36**.

***rac*-2-(3-cyclobutyl-2-hydroxy-2-(4-methoxyphenyl)propyl)phenol (34a) and *rac*-2-(cyclobutylmethyl)-2-(4-methoxyphenyl)-2,3-dihydrobenzofuran (34b)**

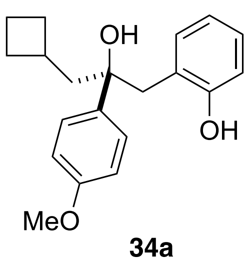

34a

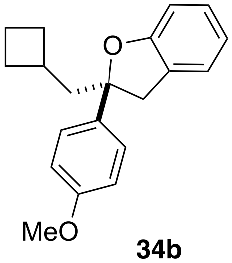

34b

Following the general procedure, vinylbromide **32** (63.9 mg, 0.300 mmol) and boronic ester **S3** (76.5 mg, 0.39 mmol) was converted to corresponding hydroxyphenol. Purification of the crude mixture by flash chromatography gave **34a** (8.4 mg, 0.0342 mmol, 11%) and dihiydrobenzofuran **34b** (36.7 mg, 0.124 mmol, 41%).

34a: Yellow solid; IR (film) 3292, 2959, 2930, 2859, 1584, 1489, 1248, 1035, 760 cm^{-1}; ^1H NMR (600 MHz, $CDCl_3$) δ 8.29 (br s, 1H), 7.27–7.24 (m, 2H), 7.11 (app td, *J* = 8.4, 1.2 Hz, 1H), 6.91 (dd, *J* = 8.4, 1.2 Hz, 1H), 6.86–6.83 (m, 2H), 6.77 (dd, *J* = 7.8, 1.8 Hz, 1H), 6.72 (app td, *J* = 7.2, 1.2 Hz, 1H), 3.80 (s, 3H), 3.01 (ABq, *J* = 14.4 Hz, 2H), 2.54 (br s, 1H), 2.26–2.18 (m, 1H), 2.10 (dd, *J* = 14.4, 5.4 Hz, 1H), 1.95 (dd, *J* = 14.4, 8.4 Hz, 1H), 1.96–1.88 (m, 1H), 1.79–1.57 (m, 4H), 1.60–1.50 (m, 1H); ^{13}C NMR (150 MHz, $CDCl_3$) δ 158.5, 155.9, 137.2, 132.9, 128.4, 126.2, 124.1, 120.1, 117.4, 113.6, 81.2, 55.4, 47.4, 46.9, 31.7, 29.9, 29.7, 19.9; HRMS (ESI) *m/z* calcd for $C_{20}H_{24}NaO_3$ $[M+Na]^+$ 335.1618, found 335.1618.

34b: Yellow solid; IR (film) 2955, 2930, 2857, 1584, 1481, 1248, 1034, 752 cm^{-1}; ^1H NMR (600 MHz, $CDCl_3$) δ 7.35–7.31 (m, 2H), 7.13–7.08 (m, 2H), 6.87–6.84 (m, 2H), 6.81 (app td, *J* = 7.8, 1.2 Hz, 1H), 3.79 (s, 3H), 3.35 (ABq, *J* = 15.6 Hz, 2H), 2.40–2.32 (m, 1H), 2.13 (dd, *J* = 14.4, 6.6 Hz, 1H), 2.06 (dd, *J* = 14.4, 6.6 Hz, 1H), 1.99–1.90 (m, 1H), 1.76–1.60 (m, 4H), 1.54–1.46 (m, 1H); ^{13}C NMR (150 MHz, $CDCl_3$) δ 159.1, 158.5, 138.2, 128.1, 126.8, 126.3, 125.0, 120.3, 113.6, 109.5, 91.7, 55.4, 49.6, 43.5, 32.4, 30.0, 29.7, 19.4; HRMS (ESI) *m/z* calcd for $C_{20}H_{22}NaO_2$ $[M+Na]^+$ 317.1512, found 317.1512.

***rac*-2-(4-methoxyphenyl)-2-(3-phenoxypropyl)-2,3-dihydrobenzofuran (35)**

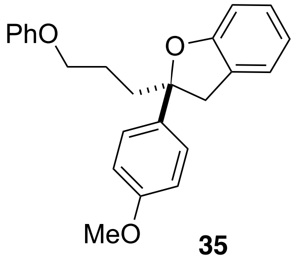

35

Following the general procedure, vinylbromide **32** (63.9 mg, 0.30 mmol) and boronic ester **S4** (102 mg, 0.33 mmol) was converted to corresponding hydroxyphenol. Purification of the crude mixture by flash chromatography gave dihydrobenzofuran **35** (48.4 mg, 0.134 mmol, 45%).

35: Yellow solid; IR (film) 2924, 2899, 1586, 1479, 1246, 1034, 750 cm^{-1}; ^{1}H NMR (600 MHz, $CDCl_3$) δ 7.38–7.35 (m, 2H), 7.26–7.22 (m, 2H), 7.15–7.10 (m, 2H), 6.91 (tt, *J* = 7.8, 1.2 Hz, 1H), 6.89–6.86 (m, 3H), 6.84–6.82 (m, 3H), 3.89 (app td, *J* = 6.6, 1.2 Hz, 2H), 3.79 (s, 3H), 3.41 (ABq, *J* = 15.6 Hz, 2H), 2.26–2.14 (m, 2H), 1.94–1.86 (m, 1H), 1.73–1.65 (m, 1H); ^{13}C NMR (150 MHz, $CDCl_3$) δ 159.1, 158.6, 137.7, 129.5, 128.2, 126.6, 126.3, 125.1, 120.7, 120.5, 114.6, 113.8, 109.6, 91.2, 67.8, 55.4, 43.9, 39.0, 24.3; HRMS (ESI) *m/z* calcd for $C_{24}H_{24}NaO_3$ $[M+Na]^+$ 383.1618, found 383.1616.

Aryne triggered annulative coupling of a vinylboronic ester with internal alkene

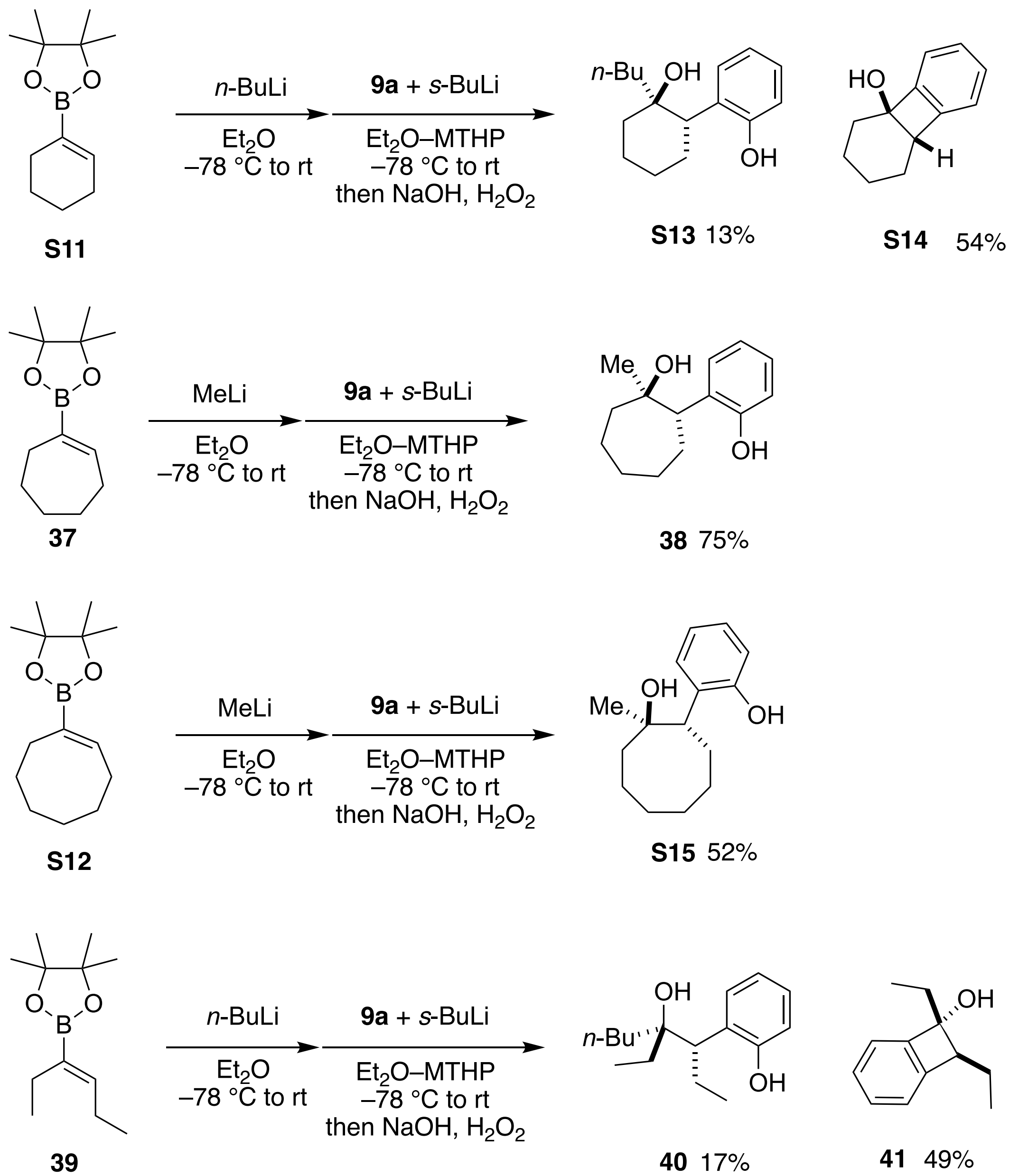

Figure S1. Annulative coupling of boronic esters possessing internal alkene. Significant amount of [2+2]-type products were obtained when **S11** and **39** were used as substrates (54% and 49% respectively).

Procedure for the aryne triggered annulative coupling of a vinylboronic ester with internal alkene (oxidative workup)

***rac*-(1*R*,2*S*)-1-butyl-2-(2-hydroxyphenyl)cycloheptan-1-ol (38)**

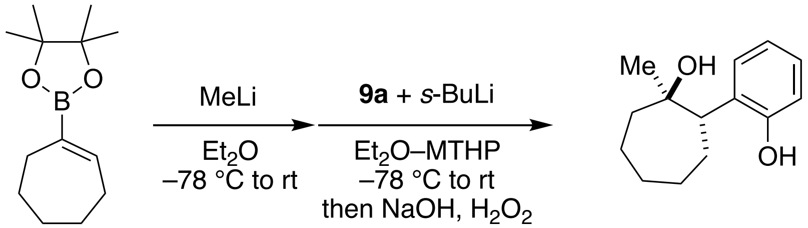

37 **38**

To a solution of vinylboronic ester **37** (56.1 mg, 0.253 mmol, 1.0 eq.) in Et_2O (2.0 mL) was added MeLi (3.1 M in hexanes, 0.115 mL, 0.357 mmol, 1.4 eq.) at –78 °C and stirred for 5 min. A cooling bath was removed, and the mixture was further stirred for 30 min. The solvent was removed in vacuo and the flask was back filled with nitrogen. The obtained mixture of vinylboronic ester ate-complex was then dissolved in 4-methyltetrahdyropyran (4.0 mL). In the meantime, aryne precursor **9a** (264 mg, 0.75 mmol, 3.0 eq.) was added to a separate flask and dissolved in Et_2O (4.0 mL). The solution was treated with *s*-BuLi (1.2 M in hexanes, 0.625 mL, 0.75 mmol, 3.0 eq.) at –78 °C and stirred for 10 min at this temperature. To this activated aryne precursor solution was then added the solution of vinylboronic ester ate-complex prepared above dropwise at –78 °C. The reaction mixture was stirred at the same temperature for 10 min and a dry ice/acetone bath was exchanged to water bath which temperature was controlled to 20 °C with ice. The reaction mixture was stirred at the same temperature for 2 h. After this period of time, the reaction mixture was cooled to 0 °C and treated with aqueous solution of NaOH (3 M, 5 mL) and aqueous solution of H_2O_2 (30%, 5 mL). This biphasic solution was warmed to rt and stirred vigorously overnight. The flask was then cooled to 0 °C and treated with saturated aqueous solution of NH_4Cl until the pH of the mixture became neutral. The mixture was extracted with Et_2O. The organic layers were washed with brine, dried over Na_2SO_4 and concentrated under reduced pressure. The crude product was purified by column chromatography on silica gel to obtain hydroxyphenol **38** (41.7 mg, 0.189 mmol, 75%).

38: colorless solid; IR (film) 3603, 3277, 3019, 2932, 2859, 2400, 1582, 1487, 1456, 1215, 763, 746, 669 cm^{-1}; ^1H NMR (600 MHz, $CDCl_3$) δ 8.37 (br s, 1H), 7.11 (ddd, *J* = 7.8, 7.2, 1.8 Hz, 1H), 7.04 (dd, *J* = 7.8, 1.8 Hz, 1H), 6.92 (dd, *J* = 8.4, 1.2 Hz, 1H), 6.89 (app td, *J* = 7.2, 1.2 Hz, 1H), 3.26 (d, *J* = 10.2 Hz, 1H), 1.99–1.93 (m, 1H), 1.93–1.87 (m, 3H), 1.87–1.79 (m, 2H), 1.76 (dd, *J* = 14.4, 10.2 Hz, 1H), 1.73–1.63 (m, 2H), 1.51 (dddd, *J* = 26.4, 12.6, 4.8, 2.4 Hz, 1H), 1.38–1.29 (m, 1H), 0.97 (s, 3H); ^{13}C NMR (150 MHz, $CDCl_3$) δ 154.7, 132.0, 129.1, 127.4, 120.4, 117.5, 80.1, 48.1, 45.0, 30.9, 30.8, 30.3, 26.7, 21.8; HRMS (ESI) *m/z* calcd for $C_{14}H_{20}NaO_2$ $[M+Na]^+$ 243.1356, found 243.1350.

Stereochemistry of **38** was assigned as shown based on a proposed mechanism.

rac-2-((1_S_,2_R_)-2-butyl-2-hydroxycyclohexyl)phenol (S13)

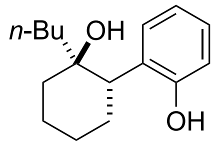

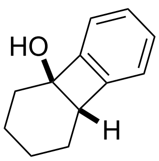

S13 **S14**

Following the procedure described above (1.3 eq. of 2 M solution of *n*-butyllithium was used), vinylboronic ester **S11** (54.0 mg, 0.259 mmol) was converted to the corresponding hydroxyphenol. Purification of the crude mixture by flash chromatography gave **S13** (8.2 mg, 0.0330 mmol, 13%) and [2+2] adduct **S14**[19] (24.2 mg, 0.139 mmol, 54%).

S13: colorless oil; IR (film) 3300, 3019, 2936, 2862, 1582, 1487, 1456, 1217, 1044, 773, 760, 743, 669 cm^{-1}; ^{1}H NMR (600 MHz, $CDCl_3$) δ 8.31 (br s, 1H), 7.42 (app td, *J* = 7.8, 1.2 Hz, 1H), 7.09 (dd, *J* = 7.8, 1.2 Hz, 1H), 6.95 (dd, *J* = 7.8, 1.2 Hz, 1H), 6.91 (td, *J* = 7.2, 1.2 Hz, 1H), 3.10 (dd, *J* = 12.6, 3.0 Hz, 1H), 2.24 (br s, 1H), 2.07–2.01 (m, 1H), 1.95–1.84 (m, 2H), 1.78–1.69 (m, 3H), 1.58–1.52 (m, 1H), 1.39 (app qt, *J* = 13.2, 3.62 Hz, 1H), 1.31 (tt, *J* = 13.8, 3.6 Hz, 1H), 1.28–1.05 (m, 5H), 0.85 (t, *J* = 7.2 Hz, 3H); ^{13}C NMR (150 MHz, $CDCl_3$) δ 155.4, 129.4, 128.7, 127.7, 120.4, 117.7, 78.8, 47.2, 37.7, 30.6, 29.0, 26.4, 24.6, 23.8, 23.2, 14.3; HRMS (ESI) *m/z* calcd for $C_{16}H_{24}NaO_2$ $[M+Na]^+$ 271.1674, found 271.1659.

rac-(_1R,2S_)-2-(2-hydroxyphenyl)-1-methylcyclooctan-1-ol (S15)

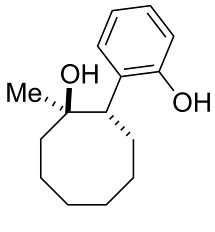

S15

Following the procedure described above (1.3 eq. of 3.1 M solution of methyllithium was used), vinylboronic ester **S12** (59.4 mg, 0.251 mmol) was converted to the corresponding hydroxyphenol. Purification of the crude mixture by flash chromatography gave **S15** (30.6 mg, 0.131 mmol, 52%).

S15: colorless oil; IR (film) 3449, 3019, 2924, 1487, 1379, 1215, 1044, 932, 775, 669 cm^{-1}; ^{1}H NMR (600 MHz, $CDCl_3$) δ 7.88 (br s, 1H), 7.15 (dd, *J* = 7.2, 1.2 Hz, 1H), 7.12 (ddd, *J* = 7.8, 7.2, 1.8 Hz, 1H), 6.95–6.90 (m, 2H), 3.57 (t, *J* = 6.0 Hz, 1H), 2.00 (ddd, *J* = 15.0, 10.2, 1.8 Hz, 1H), 1.97–1.89 (m, 1H), 1.86 (app br q, *J* = 6.0 Hz, 2H), 1.84–1.77 (m, 2H), 1.74–1.64 (m, 4H), 1.54–1.45 (m, 1H), 1.08 (s, 3H); ^{13}C NMR (150 MHz, $CDCl_3$) δ 155.4, 132.0, 129.6, 127.4, 120.6, 117.7, 79.3, 41.1, 40.8, 30.5, 28.8, 27.9, 25.5, 24.9, 21.9; HRMS (ESI) *m/z* calcd for $C_{15}H_{22}NaO_2$ $[M+Na]^+$ 257.1517, found 257.1510.

***rac*-2-((3*S*,4*S*)-4-ethyl-4-hydroxyoctan-3-yl)phenol (40)**

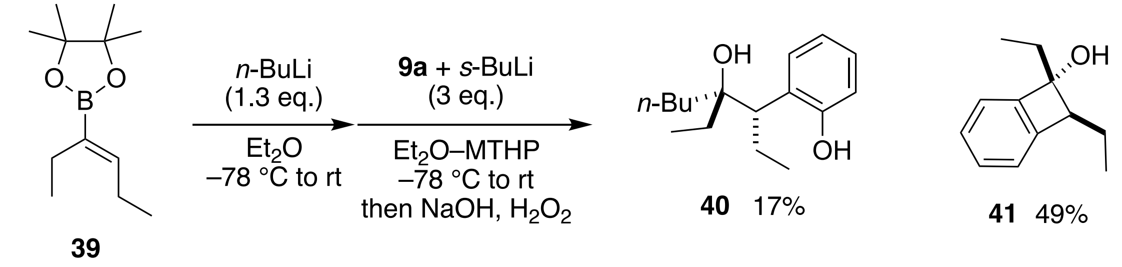

To a solution of vinylboronic ester **39** (63.0 mg, 0.250 mmol, 1.0 eq.) in Et_2O (3.0 mL) was added *n*-BuLi (1.28 M in hexanes, 0.260 mL, 0.330 mmol, 1.3 eq.) at –78 °C and stirred for 5 min. A cooling bath was removed, and the mixture was further stirred for 30 min. The solvent was removed in vacuo and the flask was back filled with nitrogen. The obtained mixture of vinylboronic ester ate-complex was then dissolved in 4-methyltetrahydropyran (5.0 mL). In the meantime, aryne precursor **9a** (264 mg, 0.75 mmol, 3.0 eq.) was added to a separate flask and dissolved in Et_2O (5.0 mL). The solution was treated with *s*-BuLi (1.23 M in hexanes, 0.610 mL, 0.75 mmol, 3.0 eq.) at –78 °C and stirred for 10 min at this temperature. To this activated aryne precursor solution was then added the solution of vinylboronic ester ate-complex prepared above dropwise at –78 °C. The reaction mixture was stirred at the same temperature for 10 min and a dry ice/acetone bath was exchanged to water bath which temperature was controlled to 20 °C with ice. The reaction mixture was stirred at the same temperature for 2 h. After this period of time, the reaction mixture was cooled to 0 °C and treated with aqueous solution of NaOH (3 M, 5 mL) and aqueous solution of H_2O_2 (30%, 5 mL). This biphasic solution was warmed to rt and stirred vigorously overnight. The flask was then cooled to 0 °C and treated with saturated aqueous solution of NH_4Cl until the pH of the mixture became neutral. The mixture was extracted with Et_2O. The organic layers were washed with brine, dried over Na_2SO_4 and concentrated under reduced pressure. The crude product was purified by column chromatography on silica gel to obtain hydroxyphenol **40** (10.4 mg, 0.0415 mmol, 17%) and benzocyclobutene **41** (21.4 mg, 0.121 mmol, 49%).

40: colorless solid; IR (film) 3395, 3021, 2955, 2874, 1489, 1217, 776, 754, 669 cm^{-1}; ^{1}H NMR (600 MHz, $CDCl_3$) δ 9.25 (br s, 1H), 7.14 (td, *J* = 7.8, 7.2 Hz, 1H), 6.91 (br d, *J* = 7.2 Hz, 1H), 6.88 (dd, *J* = 7.8, 1.2 Hz, 1H), 6.77 (t, *J* = 7.2 Hz, 1H), 2.49 (br s, 1H), 2.22 (br s, 1H), 1.91–1.82 (m, 1H), 1.82–1.74 (m, 1H), 1.72–1.63 (m, 2H), 1.45–1.32 (m, 4H), 1.32–1.18 (m, 2H), 0.96 (t, *J* = 7.2 Hz, 3H), 0.79 (br t, *J* = 7.2 Hz, 3H), 0.69 (t, *J* = 7.2 Hz, 3H); ^{13}C NMR (150 MHz, $CDCl_3$) δ 155.6, 133.5, 128.2, 126.7, 119.4, 117.8, 79.4, 55.5, 35.8, 30.4, 26.4, 23.3, 19.5, 14.2, 13.4, 7.9; HRMS (ESI) *m/z* calcd for $C_{16}H_{26}NaO_2$ $[M+Na]^+$ 273.1830, found 273.1811.

Stereochemistry of **40** was assigned as shown based on a proposed mechanism.

41: colorless solid; IR (film) 3385, 3019, 2961, 2920, 2874, 1458, 1375, 1217, 1132, 995, 970, 754 cm^{-1}; ^{1}H NMR (600 MHz, $CDCl_3$) δ 7.33–7.29 (m, 1H), 7.27–7.23 (m, 3H), 3.32 (dd, *J* = 10.2, 6.0 Hz, 1H), 2.26 (br s, 1H), 1.93 (dq, *J* = 14.4, 7.8 Hz, 1H), 1.91–1.84 (m, 1H), 1.77 (dq, *J* = 14.4, 7.8 Hz, 1H), 1.66–1.58 (m, 1H), 1.21 (t, *J* = 7.8 Hz, 3H), 1.18 (t, *J* = 7.2 Hz, 3H); ^{13}C NMR (150 MHz, $CDCl_3$) δ 149.1, 146.5, 129.3, 127.5,

123.6, 121.8, 82.9, 61.3, 27.9, 22.8, 13.2, 8.8; HRMS (ESI) *m/z* calcd for $C_{12}H_{16}NaO$ $[M+Na]^+$ 199.1099, found 199.1117.

Procedure for the aryne triggered annulative coupling of deuterated substrate and their derivatization

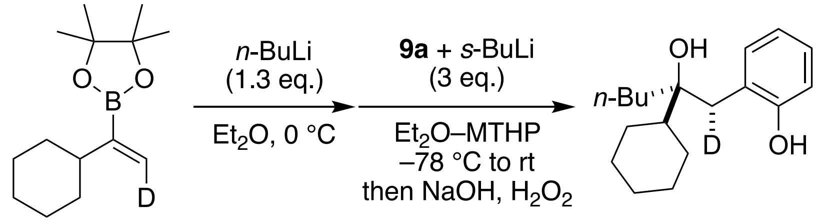

42 (E/Z = 23:1) **43** 43% (dr 15:1)

To a solution of vinylboronic ester **42** (45.9 mg, 0.194 mmol, E/Z = 23:1, 1.0 eq.) in Et_2O (2.0 mL) was added n-BuLi (1.79 M in cyclohexane, 0.145 mL, 0.26 mmol, 1.3 eq.) at –78 °C and stirred for 5 min. A cooling bath was removed, and the mixture was further stirred for 30 min. The solvent was removed in vacuo and the flask was back filled with nitrogen. The obtained mixture of vinylboronic ester ate-complex was then dissolved in 4-methyltetrahydropyran (4.0 mL). In the meantime, aryne precursor **9a** (211 mg, 0.60 mmol, 3.0 eq.) was added to a separate flask and dissolved in Et_2O (4.0 mL). The solution was treated with s-BuLi (1.23 M in hexanes, 0.490 mL, 0.75 mmol, 3.0 eq.) at –78 °C and stirred for 10 min at this temperature. To this activated aryne precursor solution was then added the solution of vinylboronic ester ate-complex prepared above dropwise at –78 °C. The reaction mixture was stirred at the same temperature for 10 min and a dry ice/acetone bath was exchanged to water bath which temperature was controlled to 20 °C with ice. The reaction mixture was stirred at the same temperature for 2 h. After this period of time, the reaction mixture was cooled to 0 °C and treated with aqueous solution of NaOH (3 M, 5 mL) and aqueous solution of H_2O_2 (30%, 5 mL). This biphasic solution was warmed to rt and stirred vigorously overnight. The flask was then cooled to 0 °C and treated with saturated aqueous solution of NH_4Cl until the pH of the mixture became neutral. The mixture was extracted with Et_2O. The organic layers were washed with brine, dried over Na_2SO_4 and concentrated under reduced pressure. The crude product was purified by column chromatography on silica gel to obtain hydroxyphenol **43** (23.1 mg, 0.0833 mmol, 43%, dr 15:1).

43: ^{1}H NMR (600 MHz, $CDCl_3$) δ 7.16 (app td, J = 7.6, 1.6 Hz, 1H), 6.97 (dd, J = 7.6, 1.6 Hz, 1H), 6.91 (dd, J = 8.0, 1.2 Hz, 1H), 6.81 (app td, J = 7.4, 1.2 Hz, 1H), 3.01 (s, 1H), 1.91–1.80 (m, 2H), 1.75 (br d, J = 12.0 Hz, 1H), 1.69 (br d, J = 10.8 Hz, 1H), 1.50–1.41 (m, 2H), 1.40–1.29 (m, 2H), 1.27–1.07 (m, 8H), 0.86 (t, J = 7.2 Hz, 3H);

***rac*-(4*S*,5*S*)-4-butyl-4-cyclohexyl-4,5-dihydrobenzo[*d*][1,3]dioxepin-2-one (S5)**

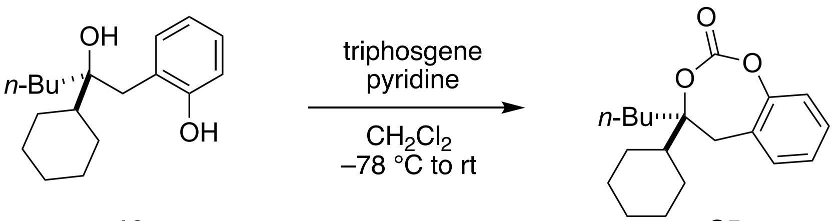

16 **S5**

To a stirred solution of **16** (9.5 mg, 0.0344 mmol) and pyridine (30 μL, 0.372 mmol) in CH_2Cl_2 (1.0 mL) was added triphosgene (12.5 mg, 0.0421 mmol) at –78 °C. After being stirred at same temperature for 10 min, the reaction mixture was warmed to rt. After 1 h, the reaction was quenched with saturated aqueous $NaHCO_3$ at 0 °C. The mixture was extracted with AcOEt. The organic layers were washed with brine, dried over Na_2SO_4 and concentrated under reduced pressure. The crude product was purified by column chromatography on silica gel to obtain carbonate **S5** (10.5 mg, 0.0347 mmol, quant.).

S5: white powder; IR (film) 2928, 2855, 1759, 1489, 1456, 1233, 1211, 1173, 1146, 1001, 764 cm^{-1}; ^{1}H NMR (600 MHz, $CDCl_3$) δ 7.31–7.26 (m, 1H), 7.19–7.16 (m, 2H), 7.11 (d, J = 7.8 Hz, 1H), 3.26 (d, J = 15.0, 1H), 2.89 (d, J = 15.0, 1H), 1.89 (br dd, J = 10.8, 1.8 Hz, 1H), 1.84–1.78 (m, 3H), 1.74 (app tt, J = 11.4, 2.4 Hz, 1H), 1.68 (br d, J = 10.8 Hz, 1H), 1.61 (dd, J = 9.0, 7.2 Hz, 2H), 1.42–1.28 (m, 2H), 1.28–1.11 (m, 8H), 0.87 (t, J = 7.2 Hz, 3H); ^{13}C NMR (150 MHz, $CDCl_3$) δ 150.7, 150.1, 130.5, 128.7, 128.0, 125.9, 119.7, 98.6, 46.0, 36.7, 35.8, 27.6, 27.4, 26.8, 26.6, 26.4, 25.7, 23.2, 14.1; HRMS (ESI) *m/z* calcd for $C_{19}H_{26}NaO_3$ $[M+Na]^+$ 325.1774, found 325.1771.

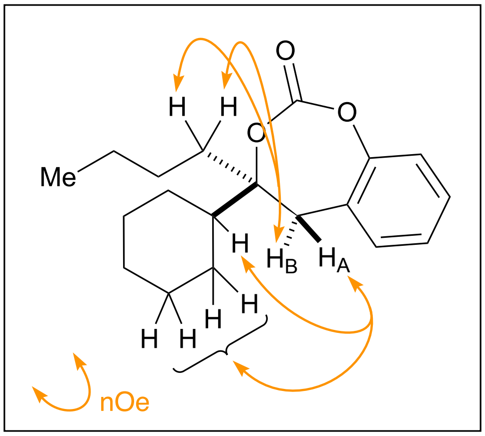

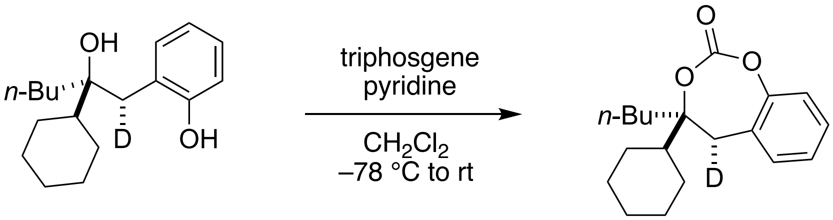

43 **S6**

Following the same procedure, deuterated hydroxyphenol **43** (9.9 mg, 0.0357 mmol) was converted to corresponding carbonate **S6** (9.7 mg, 0.0320 mmol, 90%).

By comparing the ^{1}H-NMR of **S5** and **S6**, deuterated proton was assigned as H_B.

Procedure for the aryne triggered annulative coupling (iodination of the borinic ester), and Suzuki-Miyaura coupling of aryl iodide

***rac*-2-(3-(2-iodobenzyl)-2,2-dimethylheptan-3-yl)-4,4,5,5-tetramethyl-1,3,2-dioxaborolane (44)**

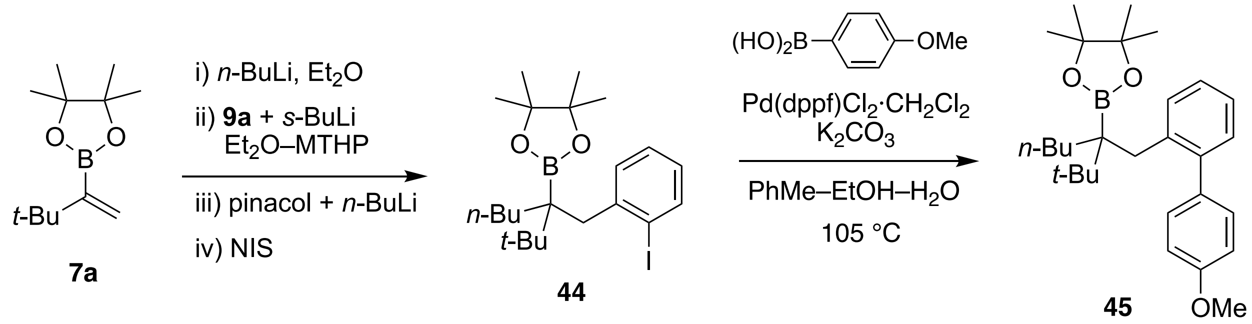

To a solution of vinylboronic ester **7a** (42.3 mg, 0.201 mmol, 1.0 eq.) in Et_2O (2.0 mL) was added *n*-BuLi (2.0 M in cyclohexanes, 1.5 mL, 0.3 mmol, 1.5 eq.) at –78 °C and stirred for 5 min. A cooling bath was removed, and the mixture was further stirred for 45 min. The solvent was removed in vacuo and the flask was back filled with nitrogen. The obtained mixture of vinylboronic ester ate-complex was then dissolved in 4-methyltetrahydropyran (4.0 mL). In the meantime, aryne precursor **9a** (0.600 mmol, 3.0 eq.) was added to a separate flask and dissolved in Et_2O (4.0 mL). The solution was treated with *s*-BuLi (1.23 M in hexanes, 0.49 mL, 0.6 mmol, 3.0 eq.) at –78 °C and stirred for 10 min at this temperature. To this activated aryne precursor solution was then added the solution of vinylboronic ester ate-complex prepared above dropwise at –78 °C. The reaction mixture was stirred at the same temperature for 10 min and a dry ice/acetone bath was exchanged to water bath which temperature was controlled to 20 °C with ice. The reaction mixture was stirred at the same temperature for 2 h before being cooled to 0 °C. In a mean time, pinacol (47.6 mg, 0.4 mmol, 2.0 eq.) placed in a separate flask was dissolved in THF (2.0 mL). This solution was treated with *n*-BuLi (2.0 M in cyclohexanes, 0.41 mL, 0.82 mmol, 4.1 eq.) at 0 °C and stirred at the same temperature for 10 min. Then, 1.2 mL (0.2 mmol) of this alkoxide solution was added dropwise to an above reaction mixture via syringe. The resulting mixture was warmed to rt and being stirred for 30 min before it was placed into a –78 °C cooling bath. To the reaction mixture was then added a THF solution (4.0 mL) of *N*-iodosuccinimide (181 mg, 0.8 mmol, 4.0 eq.) and being stirred at same temperature for 1 h. After this period of time, the reaction was quenched by the addition of saturated aqueous solution of $NaHCO_3$ and extracted with Et_2O. The organic layers were washed with brine, dried over Na_2SO_4 and concentrated under reduced pressure. The crude product was purified by column chromatography on silica gel to obtain boronic ester **44** (46.1 mg, 0.098 mmol, 49%).

44: colorless oil; IR (film) 2959, 2870, 1466, 1371, 1294, 1144, 1009, 856 cm^{-1}; ^1H NMR (600 MHz, $CDCl_3$) δ 7.78 (dd, *J* = 7.8, 1.2 Hz, 1H), 7.72 (dd, *J* = 7.8, 1.8 Hz, 1H), 7.16 (app td, *J* = 7.2, 1.2 Hz, 1H), 6.81 (app dt, *J* = 7.8, 1.8 Hz, 1H), 3.26 (d, *J* = 13.8 Hz, 1H), 2.88 (d, *J* = 13.8 Hz, 1H), 1.56–1.47 (m, 2H), 1.31 (s, 6H), 1.26 (s, 6H), 1.06 (s, 9H), 1.06–0.98 (m, 2H), 0.97–0.92 (m, 1H), 0.60 (t, *J* = 7.2 Hz, 3H), 0.14–0.06 (m, 1H); ^{13}C

NMR (150 MHz, $CDCl_3$) δ 146.0, 139.8, 131.0, 127.5, 127.4, 103.8, 83.5, 42.2, 37.1, 31.5, 29.9, 27.8, 25.9, 24.9, 24.4, 14.1; HRMS (ESI) *m/z* calcd for $C_{22}H_{36}BINaO_2$ $[M+Na]^+$ 493.1745, found 493.1746.

rac-2-(3-((4'-methoxy-[1,1'-biphenyl]-2-yl)methyl)-2,2-dimethylheptan-3-yl)-4,4,5,5-tetramethyl-1,3,2-dioxaborolane (45)

Aryliodide **44** (37.4 mg, 0.0795 mmol, 1.0 eq.), 4-methoxyphenylboronic acid (18.1 mg, 0.119 mmol, 1.5 eq.), Pd(dppf)Cl_2 (3.2 mg, 3.98 μmol, 5 mol%), and K_2CO_3 (55 mg, 0.400 mmol, 5.0 eq.) were dissolved in toluene–EtOH (5:2, 1.4 mL) and stirred at 105 °C for 2 h. After being cooled to rt, the mixture was diluted with AcOEt and water. The mixture was extracted with AcOEt, washed with brine, dried over Na_2SO_4 and concentrated under reduced pressure. The crude product was purified by column chromatography on silica gel to obtain boronic ester **45** (32.1 mg, 0.0713 mmol, 90%).

45: white powder; IR (film) 3061, 2953, 2870, 2837, 1611, 1514, 1371, 1296, 1144 cm^{-1}; ^1H NMR (400 MHz, $CDCl_3$) δ 7.73–7.68 (m, 1H), 7.25–7.21 (m, 2H), 7.19–7.11 (m, 3H), 6.96–6.91 (m, 2H), 3.85 (s, 3H), 2.98 (ABq, *J* = 13.6 Hz, 2H), 1.30 (s, 6H), 1.26 (s, 6H), 1.24–1.16 (m, 1H), 1.00–0.87 (m, 4H), 0.75 (s, 9H), 0.64 (t, *J* = 7.2 Hz, 3H), 0.35–0.23 (m, 1H); ^{13}C NMR (100 MHz, $CDCl_3$) δ 158.3, 142.9, 140.6, 135.5, 131.3, 130.8, 130.4, 129.2, 128.4, 126.4, 125.5, 125.3, 113.4, 83.2, 55.4, 36.7, 33.5, 31.8, 29.7, 27.5, 25.9, 25.0, 24.6, 14.2; HRMS (ESI) *m/z* calcd for $C_{29}H_{43}BNaO_3$ $[M+Na]^+$ 473.3197, found 473.3194.

Procedure for the aryne triggered annulative coupling/Suzuki-Miyaura coupling sequence

rac-(3-([1,1'-biphenyl]-2-ylmethyl)-2,2-dimethylheptan-3-yl)boronic acid (46)

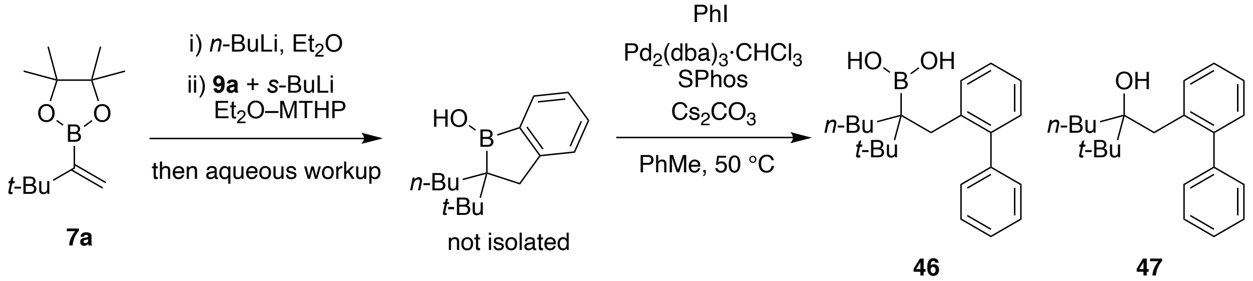

To a solution of vinylboronic ester **7a** (42 mg, 0.20 mmol, 1.0 eq.) in Et_2O (2.0 mL) was added *n*-BuLi (1.28 M in hexanes, 0.23 mL, 0.294 mmol, 1.5 eq.) at –78 °C and stirred for 5 min. A cooling bath was removed, and the mixture was further stirred for 30 min. The solvent was removed in vacuo and the flask was back filled with nitrogen. The obtained mixture of vinylboronic ester ate-complex was then dissolved in 4-methyltetrahyrdopyran (4.0 mL). In the meantime, aryne precursor **9a** (211 mg, 0.60 mmol, 3.0 eq.) was added to a separate flask and dissolved in Et_2O (4.0 mL). The solution was treated with *s*-BuLi (1.0 M in hexanes, 0.6 mL, 0.60 mmol, 3.0 eq.) at –78 °C and being stirred for 10 min at this temperature. To this activated aryne precursor solution was then added the solution of vinylboronic ester ate-complex prepared above dropwise at –78 °C. The reaction mixture was stirred at the same temperature for 10 min and a dry ice/acetone bath was exchanged to water bath which temperature was controlled to 20 °C with ice. After being stirred at same temperature for 2 h, the reaction was quenched by the addition of saturated aqueous solution of NH_4Cl. The mixture was quickly extracted with Et_2O, washed with brine, dried over Na_2SO_4 and concentrated under reduced pressure. The crude mixture was dissolved in degassed toluene (1.0 mL) and added to a separate flask containing a degassed toluene solution (4.0 mL) of $Pd_2(dba)_3·CHCl_3$ (10.3 mg, 0.010 mmol, 5 mol%), SPhos (9.9 mg, 0.024 mmol, 12 mol%) and Cs_2CO_3 (215 mg, 0.66 mmol, 3.3 eq.). The mixture was stirred at 50 °C for 6 h. The reaction was quenched by the addition of EtOAc, filtered through a pad of celite and extracted with EtOAc. The organic layers were washed with brine, dried over Na_2SO_4 and concentrated under reduced pressure. The crude product was purified by column chromatography on silica gel to obtain boronic ester **46** (25.8 mg, 0.0763 mmol, 38%), along with overoxidized alcohol **47** (24.2 mg, 0.0779 mmol, 39%).

46: pale yellow oil; IR (film) 3464, 2951, 2872, 1558, 1473, 1375, 1338, 1219, 1010, 767, 704 cm^{-1}; ^1H NMR (400 MHz, $CDCl_3$) δ 7.49–7.10 (m, 9H), 2.98 (d, *J* = 14.0 Hz, 1H), 2.86 (d, *J* = 14.0 Hz, 1H), 1.27–1.07 (m, 2H), 1.05–0.90 (m, 4H), 0.79 (s, 9H), 0.71 (t, *J* = 7.2 Hz, 3H); ^{13}C NMR (100 MHz, $CDCl_3$) δ 143.2, 142.5, 139.2, 130.4, 130.1, 129.9, 128.1, 127.3, 126.8, 125.7, 35.9, 34.2, 32.3, 30.0, 27.8, 24.7, 14.1; HRMS (ESI) *m/z* calcd for $C_{22}H_{31}BNaO_2$ [M+Na]$^+$ 361.2315, found 361.2308.

47: colorless oil; IR (film) 3676, 2960, 1598, 1396, 1261, 1093, 1020, 866, 775, 702 cm^{-1}; ^1H NMR (600 MHz, $CDCl_3$) δ 7.43–7.38 (m, 3H), 7.34–7.31 (m, 3H), 7.30–7.27 (m, 2H), 7.24–7.22 (m, 1H), 3.03 (d, *J* = 13.8 Hz, 1H), 2.95 (d, *J* = 13.8 Hz, 1H), 1.30 (ddd, *J* = 13.8, 12.6, 4.2 Hz, 1H), 1.25 (br s, 1H), 1.24–1.17 (m,

1H), 1.11–1.03 (m, 2H), 0.99–0.92 (m, 1H), 0.82–0.75 (m, 1H), 0.77 (s, 9H), 0.76 (t, J = 7.2 Hz, 3H); ^{13}C NMR (100 MHz, $CDCl_3$) δ 143.5, 142.5, 136.3, 131.8, 130.5, 130.0, 128.4, 127.1, 126.9, 126.4, 78.2, 39.0, 36.5, 35.7, 27.5, 25.6, 23.9, 14.2; HRMS (ESI) *m/z* calcd for $C_{22}H_{30}NaO$ $[M+Na]^+$ 333.2194, found 333.2200.

References

(1) Nemec, V.; Hylsova, M.; Maier, L.; Flegel, J.; Sievers, S.; Ziegler, S.; Schroder, M.; Berger, B.; Chaikuad, A.; Valcikova, B.; Uldrijan, S.; Drapela, S.; Soucek, K.; Waldmann, H.; Knapp, S.; Paruch, K. *Angew. Chem. Int. Ed.* **2019,** *58*, 1062–1066.

(2) Fordham, J.; Grayson, M.; Aggarwal, V. *Angew. Chem. Int. Ed.* **2019,** *58*, 15268–15272.

(3) Wu, C.; Bao, Z.; Dou, B.; Wang, J. *Chem. Eur. J.* **2021**, *27*, 2294–2298.

(4) Coombs, J.; Zhang, L.; Morken, J. *Org. Lett.* **2015,** *17*, 1708–1711.

(5) Hryshchuk, O.; Varenyk, A.; Yurov, Y.; Kuchkovska, Y.; Tymtsunik, A.; Grygorenko, O. *Eur. J. Org. Chem.* **2020,** 2217–2224.

(6) Aparance, M. D.; Gao, C.; Lovinger, G. J.; Morken, J. P. *Angew. Chem. Int. Ed.* **2019**, *58*, 592–595.

(7) Daniels, M. H.; Malojcic, G.; Clugston, S. L.; Williams, B.; Gal, M. C.-L.; Pan-Zhou, X.-R.; Venkatachalan, S.; Harmange, J.-C.; Ladeboer, M. *J. Med. Chem.* **2022**, *65*, 3575–3596.

(8) Sun, X.; Gu, P.; Qin, J.; Su, Y. *Chem. Commun.* **2020**, *56*, 12379–12382.

(9) Sumida, Y.; Kato, T.; Hosoya, T. *Org. Lett.* **2013,** *15*, 2806–2809.

(10) Sumida, Y.; Sumida, T.; Hashizume, D.; Hosoya, T. *Org. Lett.* **2016,** *18*, 5600–5603.

(11) Darwish, A.; Chong, J. *J. Org. Chem.* **2007,** *72*, 1507–1509.

(12) Rosiak, A.; Frey, W.; Christoffers, J. *Eur. J. Org. Chem.* **2006,** *2006*, 4044–4054.

(13) Takagi, J.; Takahashi, K.; Ishiyama, T.; Miyaura, N. *J. Am. Chem. Soc.* **2002,** *124*, 8001–8006.

(14) Wahl, J. M.; Conner, M. L.; Brown, K. M. *J. Am. Chem. Soc.* **2018**, *140*, 15943–15949.

(15) Takagi, J.; Takahashi, K.; Ishiyama, T.; Miyaura, N. *J. Am. Chem. Soc.* **2002**, *124*, 8001–8006.

(16) Kubota, K.; Yamamoto, E.; Ito, H. *J. Am. Chem. Soc.* **2015,** *137*, 12729–12729.

(17) Bull, J.; Charette, A. *J. Am. Chem. Soc.* **2010,** *132*, 1895–1902.

(18) Wang, Z.; Wang, M.; Gao, J.; Shi, S.; Xu, Y. *Org. Chem. Front.* **2019,** *6*, 2949–2953.

(19) Allen, B. D. W.; Hareram, M. D.; Seastram, A. C.; McBride, T.; Wirth, T.; Browne, D. L.; Morrill, L. C. *Org. Lett.* **2019**, *21*, 9241–9246.

DFT calculations for the reaction mechanism

Density functional theory (DFT) calculations were performed using Gaussian 09 program.[20] Geometries were optimized using B3LYP-D3BJ[21] with 6-31G(d) basis set combined with SMD[22] solvation model in diethyl ether (Et_2O). Tight convergence criteria and an ultrafine integration grid were applied. Thermochemical corrections were obtained from frequency calculations at the same level of theory. The single point energies were computed at the B3LYP-D3(BJ)/6-311+G(d,p) basis set with SMD solvation model in diethyl ether (Et_2O). Enthalpy and free energies in solution were computed by adding the gas phase thermochemical corrections to the solution phase single point energy. The data were summarized in Figure S1. Calculated structures are illustrated using ChemDraw and CYLView.[23]

In addition to the presented pathway, we also tried to find a transition state for a syn-concerted mechanism. However, we could not find a reasonable transition state structure for this pathway.

---

(20) Gaussian 09, Revision E.01, Frisch, M. J.; Trucks, G. W.; Schlegel, H. B.; Scuseria, G. E.; Robb, M. A.; Cheeseman, J. R.; Scalmani, G.; Barone, V.; Mennucci, B.; Petersson, G. A.; Nakatsuji, H.; Caricato, M.; Li, X.; Hratchian, H. P.; Izmaylov, A. F.; Bloino, J.; Zheng, G.; Sonnenberg, J. L.; Hada, M.; Ehara, M.; Toyota, K.; Fukuda, R.; Hasegawa, J.; Ishida, M.; Nakajima, T.; Honda, Y.; Kitao, O.; Nakai, H.; Vreven, T.; Montgomery, J. A., Jr.; Peralta, J. E.; Ogliaro, F.; Bearpark, M.; Heyd, J. J.; Brothers, E.; Kudin, K. N.; Staroverov, V. N.; Kobayashi, R.; Normand, J.; Raghavachari, K.; Rendell, A.; Burant, J. C.; Iyengar, S. S.; Tomasi, J.; Cossi, M.; Rega, N.; Millam, N. J.; Klene, M.; Knox, J. E.; Cross, J. B.; Bakken, V.; Adamo, C.; Jaramillo, J.; Gomperts, R.; Stratmann, R. E.; Yazyev, O.; Austin, A. J.; Cammi, R.; Pomelli, C.; Ochterski, J. W.; Martin, R. L.; Morokuma, K.; Zakrzewski, V. G.; Voth, G. A.; Salvador, P.; Dannenberg, J. J.; Dapprich, S.; Daniels, A. D.; Farkas, Ö.; Foresman, J. B.; Ortiz, J. V.; Cioslowski, J.; Fox, D. J. Gaussian, Inc., Wallingford CT, 2009.

(21)(a) Grimme, S.; Antony, J.; Ehrlich, S.; Krieg, H. *J. Chem. Phys.* **2010**, *132*, 154104–15419. (b) Grimme, S.; Ehrlich, S.; Goerigk, L. *J. Comp. Chem.* **2011**, *32*, 1456–1465. (c) Phys, J. C.; Becke, A. D.; Johnson, E. R. A Density-Functional Model of the Dispersion Interaction. *J. Chem. Phys.* **2005**, *123*, 154101–154109.

(22) Marenich, A. V.; Cramer, C. J.; Truhlar, D. G. *J. Phys. Chem. B* **2009**, *113*, 6378–6396.

(23) Legault, C. Y. CYLView, 1.0b; Université de Sherbrooke, Canada, 2009; http://www.cylview.org.

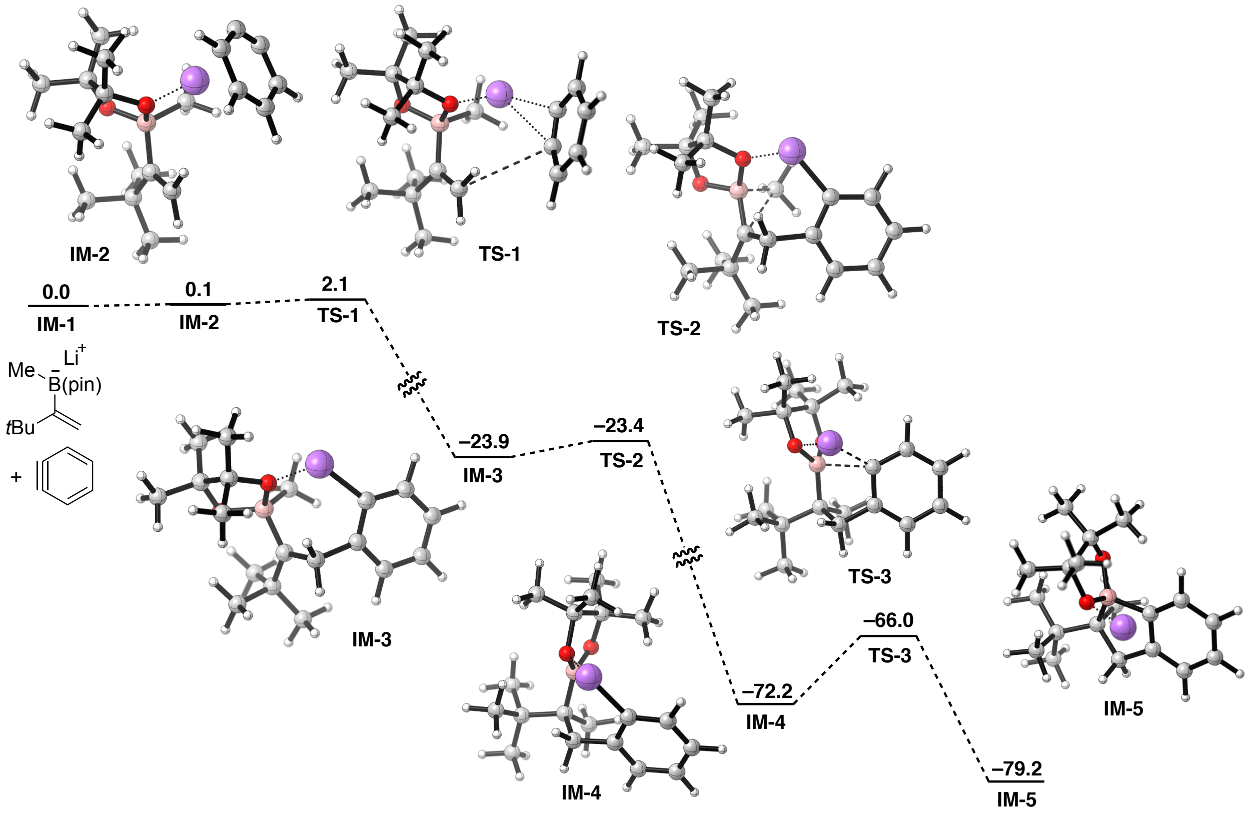

Figure S2. Calculated reaction coordinate for the benzyne induced 1,2-metallate rearrangement. Optimized geometry calculated using DFT (B3LYP-D3BJ/6-31G(d)). ΔG values are in kcal/mol, calculated using DFT (B3LYP-D3BJ/6-311+G(d,p); SMD solvation model with Et_2O). Calculated structures were shown with following color code: grey: carbon, red: oxygen, pink: boron, purple: lithium.

The Cartesian coordinates for the optimized geometries are listed, below, together with the following energies (all in Hartree):

B3LYP-D3BJ/6-31G(d)/SMD(Et_2O) electronic energy ($E_{6\text{-}31G}$)

B3LYP-D3BJ/ 6-31G(d)/SMD(Et_2O) Gibbs free energy ($G_{6\text{-}31G}$),

along with the solution-phase single-point energies at the B3LYP-D3BJ/6-311+G(d,p) of theory in Et_2O:

B3LYP-D3BJ/6-311+G(d,p)/SMD(Et_2O) electronic energy ($E_{6\text{-}311+G}$)

B3LYP-D3BJ/ 6-311+G(d,p)/SMD(Et_2O) Gibbs free energy ($G_{6\text{-}311+G}$)

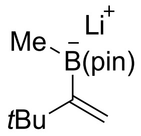

C	0.419647	1.810906	-0.795589
C	-0.710382	1.504973	-0.138244
C	-1.896520	2.485796	-0.119924
B	-0.791684	0.076309	0.652794
C	-1.517031	3.914093	-0.547713
C	-2.970547	1.956928	-1.097898
C	-2.523998	2.561502	1.287399
O	0.090979	-0.997872	-0.023210
C	-0.769946	-1.914076	-0.716995
C	-2.050373	-1.896591	0.191076
O	-2.125254	-0.548687	0.633213
C	-1.911237	-2.824486	1.412034
C	-3.342096	-2.245329	-0.549098
C	-1.032034	-1.366732	-2.124756
C	-0.070936	-3.267493	-0.818232
C	-0.201331	0.272865	2.207512
H	1.236022	1.091936	-0.861675
H	0.593357	2.750820	-1.317228
H	-2.389850	4.574537	-0.470092

H	-0.724619	4.327670	0.087937
H	-1.171202	3.953724	-1.586728
H	-3.849245	2.616592	-1.099282
H	-3.286325	0.952627	-0.807284
H	-2.576890	1.917659	-2.120878
H	-2.855680	1.576033	1.624766
H	-1.810370	2.959031	2.019635
H	-3.395287	3.229395	1.278593
H	-1.982652	-3.884113	1.141244
H	-0.961115	-2.669309	1.934781
H	-2.716487	-2.595101	2.117515
H	-3.302427	-3.257465	-0.969354
H	-4.187548	-2.202105	0.146723
H	-3.538631	-1.538807	-1.358892
H	-1.566708	-0.416093	-2.087040
H	-0.072891	-1.192814	-2.623817
H	-1.610237	-2.074134	-2.728953
H	-0.734838	-4.020155	-1.258225
H	0.813147	-3.185887	-1.461500
H	0.252586	-3.634339	0.161406
H	-0.845638	0.968549	2.751419
H	-0.207634	-0.645562	2.829455
H	0.803221	0.737354	2.279203
Li	1.205389	-1.087478	1.347213

0 imaginary frequencies

$E_{6\text{-}31G}$ = -694.090168

$G_{6\text{-}31G}$ = -693.757568

$E_{6\text{-}311+G}$ = -694.285231

$G_{6\text{-}311+G}$ = -693.952631

Benzyne

C	4.779203	0.174703	-1.097821
C	5.084206	-0.706728	-0.044436
C	4.451170	-0.631555	1.216295
C	3.816844	1.201579	-0.974463
H	5.302400	0.064276	-2.044902
H	5.835664	-1.476793	-0.203186
H	4.686046	-1.310972	2.028771
H	3.581368	1.881415	-1.786415
C	3.259216	1.187490	0.293898
C	3.530416	0.403770	1.230527

0 imaginary frequencies

$E_{6\text{-}31G}$ = -230.933774

$G_{6\text{-}31G}$ = -230.88588

$E_{6\text{-}311+G}$ = -230.996913

$G_{6\text{-}311+G}$ = -230.949019

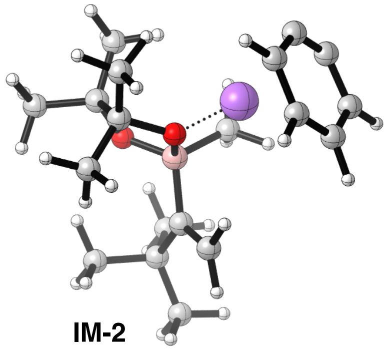

IM-2

C	-0.709311	1.983320	-1.331924
C	-1.424705	1.336844	-0.397288
C	-2.897963	1.703317	-0.146405
B	-0.687620	0.164926	0.476100
C	-3.302563	3.063044	-0.741812
C	-3.779155	0.609958	-0.792607
C	-3.204162	1.735511	1.365143
O	0.429016	-0.519968	-0.336669
C	-0.033985	-1.816291	-0.736319
C	-0.972169	-2.203042	0.461001
O	-1.566897	-0.966773	0.827778
C	-0.182321	-2.763982	1.658033
C	-2.078110	-3.191921	0.090717
C	-0.790721	-1.676043	-2.062453
C	1.168846	-2.736293	-0.931661
C	0.038470	0.849425	1.814475
H	0.315142	1.683421	-1.551753
H	-1.087424	2.805732	-1.937600
H	-4.347380	3.287392	-0.492508
H	-2.680766	3.876127	-0.347614
H	-3.219750	3.074380	-1.834428
H	-4.843559	0.816264	-0.614077
H	-3.536049	-0.368629	-0.373033
H	-3.619989	0.575142	-1.877278

H	-2.980007	0.773270	1.832702
H	-2.620241	2.513519	1.872172
H	-4.266528	1.956711	1.532376
H	0.186634	-3.779770	1.474579
H	0.673819	-2.130224	1.912020
H	-0.844063	-2.792080	2.529968
H	-1.663235	-4.141404	-0.268707
H	-2.691678	-3.405019	0.973368
H	-2.733808	-2.783175	-0.681767
H	-1.680684	-1.055090	-1.946537
H	-0.136844	-1.191657	-2.795536
H	-1.089438	-2.651773	-2.460994
H	0.845843	-3.762604	-1.140351
H	1.766877	-2.395096	-1.784595
H	1.814332	-2.756505	-0.048375
H	-0.734701	1.249064	2.476797
H	0.606988	0.141107	2.448578
H	0.689209	1.718660	1.603512
L	1.740266	0.135914	0.653507
C	3.625810	1.953793	-0.491616
C	3.885424	0.746694	-1.173462
C	4.168936	-0.464785	-0.500940
C	3.629800	2.049444	0.920767
H	3.404571	2.847454	-1.068818
H	3.855436	0.740245	-2.259632
H	4.355217	-1.396202	-1.021477
H	3.419966	2.971925	1.448789
C	3.921572	0.809847	1.481439
C	4.147499	-0.264730	0.876337

0 imaginary frequencies

$E_{6\text{-}31G}$ = -925.051355

$G_{6\text{-}31G}$ = -924.653319

$E_{6\text{-}311+G}$ = -925.299458

$G_{6\text{-}311+G}$ = -924.901422

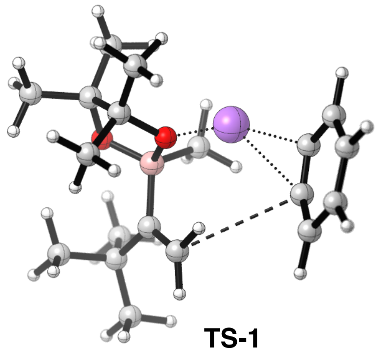

TS-1

C	-0.378071	1.931463	-0.747985
C	-1.263982	1.275838	0.020677
C	-2.695436	1.812568	0.194037
B	-0.788549	-0.105005	0.764499
C	-2.845642	3.295865	-0.186334
C	-3.621375	0.978193	-0.720889
C	-3.179399	1.642053	1.648335
O	0.357495	-0.783814	-0.009467
C	-0.164344	-1.937515	-0.677680
C	-1.308099	-2.384369	0.300880
O	-1.823124	-1.154655	0.791981
C	-0.771732	-3.217905	1.478662
C	-2.441061	-3.155685	-0.377030
C	-0.694255	-1.508250	-2.051439
C	0.962623	-2.951376	-0.859184
C	-0.193846	0.250113	2.284613
H	0.604971	1.506362	-0.944152
H	-0.577275	2.879292	-1.246427
H	-3.873703	3.630619	0.000931
H	-2.172438	3.931633	0.401769
H	-2.635394	3.472178	-1.247129
H	-4.665970	1.300810	-0.610937
H	-3.551464	-0.081213	-0.463805
H	-3.338721	1.101339	-1.773484

H	-3.146948	0.592917	1.953527
H	-2.564605	2.226264	2.343850
H	-4.215056	1.993061	1.745906
H	-0.485164	-4.231470	1.174833
H	0.095381	-2.741620	1.946637
H	-1.558193	-3.296953	2.236497
H	-2.076549	-4.082363	-0.836628
H	-3.199397	-3.424226	0.367140
H	-2.927147	-2.552014	-1.147167
H	-1.524750	-0.806270	-1.953782
H	0.108287	-1.004844	-2.601071
H	-1.026339	-2.368943	-2.642248
H	0.585604	-3.887495	-1.286772
H	1.718302	-2.550871	-1.545490
H	1.454614	-3.183807	0.090340
H	-1.017564	0.557504	2.934951
H	0.263805	-0.614794	2.803252
H	0.517939	1.095859	2.335662
Li	1.598344	-0.264519	1.143231
C	4.666040	1.942882	-1.359497
C	5.277019	0.869313	-0.684929
C	4.721908	0.285952	0.466555
C	3.439847	2.511308	-0.937937
H	5.149377	2.374106	-2.232583
H	6.211561	0.473523	-1.073539
H	5.187048	-0.559116	0.963150
H	2.945999	3.335955	-1.434284
C	3.077754	1.845114	0.199010
C	3.498540	0.866428	0.873283

1 imaginary frequencies

$E_{6\text{-}31G}$ = -925.046278

$G_{6\text{-}31G}$ = -924.648239

$E_{6\text{-}311+G}$ = -925.296343

$G_{6\text{-}311+G}$ = -924.898304

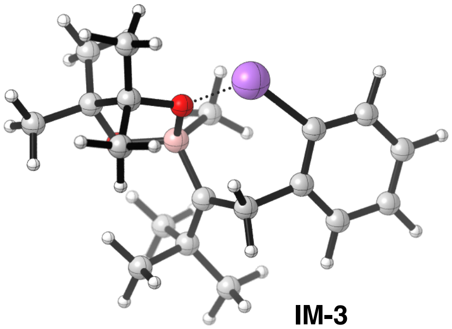

IM-3

C	0.389809	1.134305	-1.344093
C	-0.593304	0.838311	-0.278729
C	-1.665953	1.826498	0.034472
B	-0.504497	-0.574385	0.518527
C	-1.371754	3.307160	-0.255093
C	-2.754565	1.316506	-0.987406
C	-2.251124	1.711464	1.454769
O	0.277473	-1.617494	-0.238439
C	-0.623862	-2.639323	-0.699349
C	-1.738618	-2.587600	0.399391
O	-1.826005	-1.196147	0.696763
C	-1.333244	-3.345971	1.673383
C	-3.107268	-3.073282	-0.072088
C	-1.140360	-2.242296	-2.087145
C	0.136761	-3.958717	-0.787061
C	0.284918	-0.169627	1.925966
H	0.584776	0.219256	-1.908115
H	0.038140	1.913580	-2.023843
H	-2.283607	3.886952	-0.075855
H	-0.593564	3.686900	0.412659
H	-1.064654	3.493371	-1.287470
H	-3.624753	1.971004	-0.861780
H	-3.044079	0.288709	-0.764282
H	-2.415888	1.392598	-2.025001
H	-2.541848	0.687603	1.689914

H	-1.525160	2.055143	2.198016
H	-3.135663	2.354032	1.527984
H	-1.357795	-4.432380	1.532772
H	-0.330173	-3.060354	2.003333
H	-2.036600	-3.088383	2.471964
H	-3.068854	-4.121720	-0.389981
H	-3.827766	-2.996631	0.749636
H	-3.481239	-2.470378	-0.903509
H	-1.713577	-1.311747	-2.048349
H	-0.288314	-2.092129	-2.759254
H	-1.777817	-3.020887	-2.518851
H	-0.539936	-4.785524	-1.030102
H	0.892325	-3.907163	-1.580938
H	0.641762	-4.192929	0.154026
H	-0.440096	-0.202380	2.747460
H	1.062480	-0.917221	2.137468
H	0.776982	0.808748	1.959717
Li	2.059268	-1.300079	-0.243565
C	3.183582	3.459562	-0.129048
C	4.160834	2.550669	0.277869
C	3.913433	1.177131	0.170064
C	1.972392	2.978202	-0.628016
H	3.360507	4.531036	-0.071287
H	5.109608	2.911837	0.672133
H	4.710180	0.501851	0.491385
H	1.223498	3.682793	-0.976973
C	1.751738	1.594745	-0.715035
C	2.704676	0.630881	-0.317806

0 imaginary frequencies

$E_{6\text{-}31G}$ = -925.099638

$G_{6\text{-}31G}$ = -924.693374

$E_{6\text{-}311+G}$ = -925.345901

$G_{6\text{-}311+G}$ = -924.939637

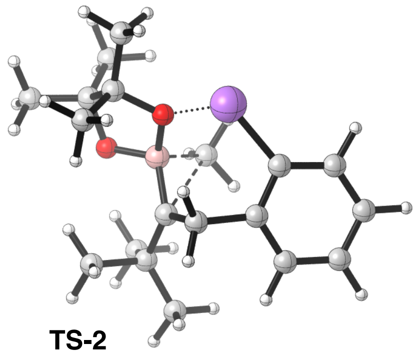

TS-2

C	1.323530	0.322572	-1.256349
C	0.419054	0.956758	-0.258802
C	0.543311	2.419618	0.023923
B	-0.670668	0.055927	0.488491
C	1.924520	3.060149	-0.189375
C	-0.448514	2.953909	-1.075924
C	0.010714	2.859923	1.400983
O	-0.999504	-1.226153	-0.219779
C	-2.369759	-1.169685	-0.660930
C	-3.007334	-0.252094	0.435181
O	-1.971668	0.695067	0.692712
C	-3.314022	-1.021480	1.729502
C	-4.253416	0.500864	-0.023616
C	-2.400803	-0.535845	-2.056176
C	-2.923043	-2.589604	-0.719979
C	0.204636	-0.259209	1.892049
H	0.726532	-0.370780	-1.855950
H	1.761876	1.074795	-1.916442
H	1.840584	4.140279	-0.027629
H	2.650992	2.667582	0.527789
H	2.318957	2.912179	-1.198140
H	-0.450709	4.046921	-0.993901
H	-1.459553	2.582153	-0.898992
H	-0.128499	2.688888	-2.088387

H	-0.986872	2.465962	1.594839
H	0.684719	2.528911	2.196961
H	-0.030199	3.954471	1.431377
H	-4.176634	-1.688031	1.619131
H	-2.453885	-1.616318	2.051001
H	-3.539149	-0.299050	2.520809
H	-5.050422	-0.192245	-0.316917
H	-4.631240	1.121198	0.796501
H	-4.034123	1.158747	-0.868373
H	-2.030022	0.492876	-2.033246
H	-1.761175	-1.116244	-2.730267
H	-3.412763	-0.527950	-2.474132
H	-3.994803	-2.582020	-0.947307
H	-2.422870	-3.158249	-1.514068
H	-2.776272	-3.116907	0.226611
H	-0.318331	0.249001	2.709262
H	0.183531	-1.340619	2.075329
H	1.257284	0.040290	1.934742
Li	0.323634	-2.466167	-0.292314
C	4.798350	-0.543226	0.099092
C	4.639935	-1.879997	0.465051
C	3.401470	-2.504686	0.275260
C	3.712458	0.146956	-0.442743
H	5.755584	-0.041633	0.221838
H	5.477678	-2.431248	0.889912
H	3.325346	-3.555138	0.567006
H	3.840438	1.179364	-0.755499
C	2.480843	-0.503579	-0.609708
C	2.265530	-1.856351	-0.260416

1 imaginary frequencies

$E_{6\text{-}31G}$ = -925.099579

$G_{6\text{-}31G}$ = -924.692425

$E_{6\text{-}311+G}$ = -925.346035

$G_{6\text{-}311+G}$ = -924.938882

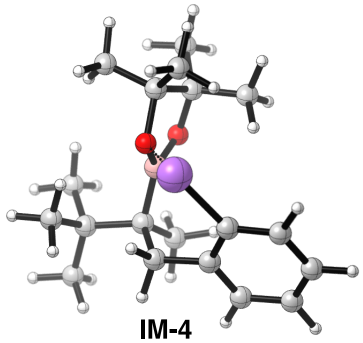

IM-4

C	1.821568	0.683640	-0.639960
C	0.639903	1.362086	0.132958
C	0.284871	2.775281	-0.518428
B	-0.651964	0.446622	0.093828
C	1.393110	3.813302	-0.255894
C	0.089318	2.663066	-2.043924
C	-1.025624	3.334619	0.074125
O	-1.106901	-0.251970	-1.035883
C	-2.449239	-0.788085	-0.732417
C	-2.435293	-0.804511	0.836209
O	-1.486320	0.256255	1.154598
C	-1.858421	-2.091382	1.428953
C	-3.771688	-0.468007	1.482415
C	-3.453581	0.187378	-1.340209
C	-2.575693	-2.155303	-1.393630
C	1.049855	1.532197	1.612736
H	1.536445	0.551418	-1.690213
H	2.664078	1.386809	-0.645483
H	1.169348	4.737120	-0.803492
H	1.469406	4.072893	0.804628
H	2.375851	3.465556	-0.590866
H	-0.296054	3.611044	-2.438682
H	-0.628206	1.878273	-2.305735
H	1.028238	2.453268	-2.565729
H	-1.894125	2.724543	-0.198778

H	-0.994727	3.398023	1.165908
H	-1.208063	4.345285	-0.310705
H	-2.534347	-2.939431	1.280153
H	-0.882621	-2.332827	0.995056
H	-1.718744	-1.949243	2.504769
H	-4.529559	-1.210722	1.210844
H	-3.663026	-0.478295	2.571392
H	-4.127643	0.521155	1.185715
H	-3.371572	1.182441	-0.895226
H	-3.264130	0.277503	-2.414500
H	-4.478403	-0.170148	-1.202435
H	-3.549749	-2.599429	-1.165404
H	-2.511809	-2.051972	-2.483735
H	-1.808506	-2.858527	-1.054178
H	0.296716	2.077458	2.188447
H	1.180197	0.558744	2.092220
H	2.000643	2.068406	1.704055
Li	0.119324	-1.479317	-1.784837
C	3.782556	-1.885039	1.389197
C	3.225628	-3.083841	0.942249
C	2.219903	-3.053715	-0.032350
C	3.317834	-0.679761	0.859120
H	4.574268	-1.885559	2.135603
H	3.579662	-4.032784	1.343587
H	1.820235	-4.016316	-0.363333
H	3.759131	0.256797	1.198649
C	2.297289	-0.663486	-0.105664
C	1.714036	-1.863795	-0.601794

0 imaginary frequencies

$E_{6\text{-}31G}$ = -925.181114

$G_{6\text{-}31G}$ = -924.77142

$E_{6\text{-}311+G}$ = -925.426387

$G_{6\text{-}311+G}$ = -925.016693

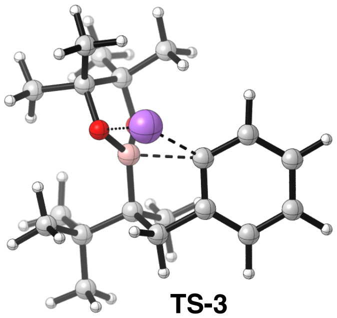

TS-3

C	1.506528	1.641306	-0.649517
C	0.229951	1.485041	0.223519
C	-0.844707	2.601722	-0.129706
B	-0.368680	-0.005536	0.058035
C	-0.252761	4.022609	-0.008550
C	-1.379324	2.464642	-1.570468
C	-2.047121	2.522362	0.834754
O	-0.978335	-0.536307	-1.134360
C	-2.004792	-1.508358	-0.749189
C	-1.602282	-1.850622	0.733661
O	-0.887577	-0.669770	1.155258
C	-0.644302	-3.040018	0.849176
C	-2.786378	-2.052173	1.673544
C	-3.360554	-0.815888	-0.885168
C	-1.941676	-2.683921	-1.721265
C	0.673259	1.613296	1.701707
H	1.238180	1.624674	-1.715619
H	1.979299	2.613408	-0.465226
H	-1.047621	4.765243	-0.153744
H	0.189687	4.209117	0.974953
H	0.513199	4.216087	-0.766863
H	-2.058859	3.297312	-1.792084
H	-1.926120	1.532999	-1.720096
H	-0.573421	2.500276	-2.311789
H	-2.424072	1.500855	0.937479

H	-1.786678	2.874910	1.837939
H	-2.868352	3.152200	0.470793
H	-1.138132	-3.983100	0.593790
H	0.235064	-2.912373	0.213243
H	-0.289920	-3.104551	1.882343
H	-3.397999	-2.903791	1.355741
H	-2.417296	-2.258430	2.683284
H	-3.420202	-1.163766	1.721038
H	-3.445635	0.047132	-0.221829
H	-3.484191	-0.465884	-1.914843
H	-4.178440	-1.507705	-0.660556
H	-2.678801	-3.447071	-1.451248
H	-2.173180	-2.340969	-2.735846
H	-0.961559	-3.174047	-1.732831
H	-0.163598	1.502642	2.394663
H	1.398453	0.833493	1.949237
H	1.157298	2.577542	1.897249
Li	0.572806	-1.236715	-1.827684
C	4.639862	-0.284618	0.389912
C	4.151433	-1.590770	0.368874
C	2.823586	-1.825395	-0.018940
C	3.794227	0.771923	0.033681
H	5.665373	-0.084843	0.693122
H	4.797379	-2.419260	0.655704
H	2.479298	-2.863484	-0.017388
H	4.163660	1.796381	0.072850
C	2.475000	0.515557	-0.352069
C	1.940926	-0.793438	-0.396360

1 imaginary frequencies

$E_{6\text{-}31G}$ = -925.172589

$G_{6\text{-}31G}$ = -924.761151

$E_{6\text{-}311+G}$ = -925.418208

$G_{6\text{-}311+G}$ = -925.006770

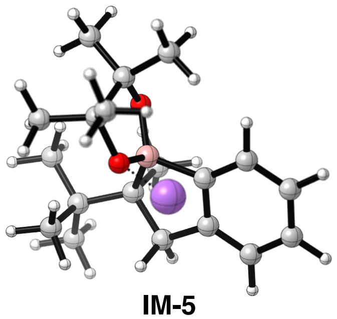

IM-5

C	1.888753	1.556403	-0.351265
C	0.508369	1.517263	0.365052
C	-0.429059	2.669818	-0.146223
B	0.026325	-0.088843	0.232821
C	0.229112	4.062527	-0.014567
C	-0.779348	2.498291	-1.639428
C	-1.731993	2.698816	0.680120
O	-0.649459	-0.568611	-1.086582
C	-1.927754	-1.178037	-0.823929
C	-1.891245	-1.436973	0.738120
O	-0.942878	-0.506254	1.234101
C	-1.450864	-2.867503	1.094907
C	-3.225838	-1.171232	1.442478
C	-3.045796	-0.226624	-1.266601
C	-2.035927	-2.448118	-1.675927
C	0.792213	1.692066	1.878497
H	1.776256	1.787267	-1.419430
H	2.561313	2.327392	0.051280

H	-0.462558	4.839315	-0.367559
H	0.488218	4.307854	1.020597
H	1.141212	4.141916	-0.617527
H	-1.515249	3.252942	-1.946918
H	-1.182111	1.510023	-1.850302
H	0.102302	2.639065	-2.276944
H	-2.138470	1.695674	0.819044
H	-1.559799	3.115485	1.678763
H	-2.490663	3.325763	0.193771
H	-2.234801	-3.603002	0.880452
H	-0.552310	-3.170864	0.554697
H	-1.227461	-2.906058	2.165842
H	-4.017332	-1.832930	1.071829
H	-3.107764	-1.355222	2.515987
H	-3.548893	-0.135523	1.316022
H	-3.036046	0.710265	-0.710557
H	-2.919927	0.009600	-2.328760
H	-4.029370	-0.691805	-1.140935
H	-2.975952	-2.977443	-1.485252
H	-2.019007	-2.181886	-2.740103
H	-1.218779	-3.151911	-1.483085
H	-0.109527	1.545133	2.478065
H	1.520474	0.948360	2.222871
H	1.210264	2.678713	2.118009

Li	0.786508	-1.406100	-1.773974
C	4.277068	-1.455564	-0.180799
C	3.366758	-2.434757	0.225868
C	2.018454	-2.101804	0.417947
C	3.842453	-0.142387	-0.383508
H	5.324322	-1.710375	-0.322979
H	3.708559	-3.450486	0.411061
H	1.339307	-2.876923	0.763759
H	4.554704	0.626977	-0.676727
C	2.495945	0.181031	-0.192651
C	1.549645	-0.790577	0.205997

0 imaginary frequencies

$E_{6\text{-}31G}$ = -925.194881

$G_{6\text{-}31G}$ = -924.783425

$E_{6\text{-}311+G}$ = -925.439199

$G_{6\text{-}311+G}$ = -925.027743

Effect of substituents in 1,2-metallate rearrangement

To further understand the 1,2-metallate rearrangement step of this cascade reaction, we calculated the energy profile of the rearrangement of the ate-complex bearing different substituents. The calculated reaction coordinate of the ate-complex having methyl group instead of *tert*-butyl group is shown in Figure S3 (The results of *tert*-butyl substituted vinylboronate is also shown as a reference). According to the calculated results, activation barrier of the rearrangement is very small in both example (0.5 kcal/mol and 1.2 kcal/mol respectively). These results suggest that low yield observed in the annulative coupling using viniylboronic ester **7c** is not due to the inefficiency of the 1,2-metallate rearrangement. We speculate that competitive cyclization of IM-3-type intermediate producing [2+2]-type cycloadduct is quite fast and efficient when **7c** was used as a substrate.

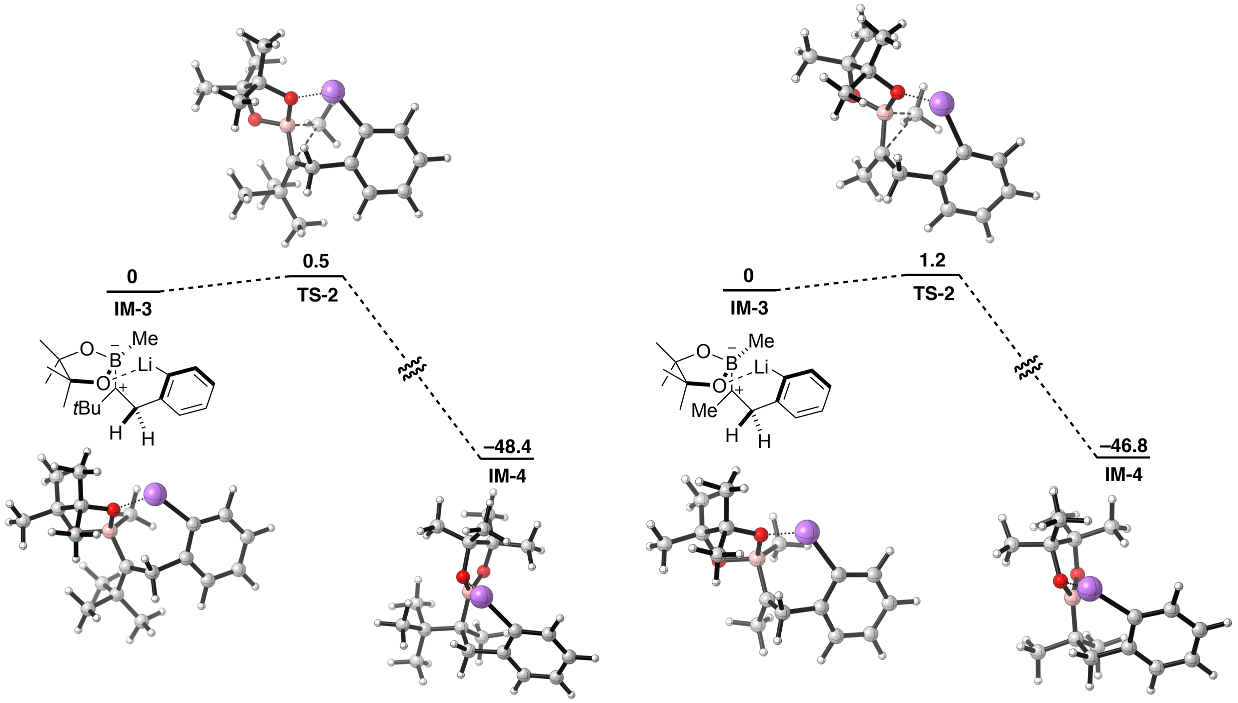

Figure S3 Calculated reaction coordinate for the 1,2-metallate rearrangement. Optimized geometry calculated using DFT (B3LYP-D3BJ/6-31G(d); SMD solvation model with Et_2O). ΔG values are in kcal/mol, calculated using DFT (B3LYP-D3BJ/6-311+G(d,p); SMD solvation model with Et_2O). Calculated structures were shown with following color code: grey: carbon, red: oxygen, pink: boron, purple: lithium.

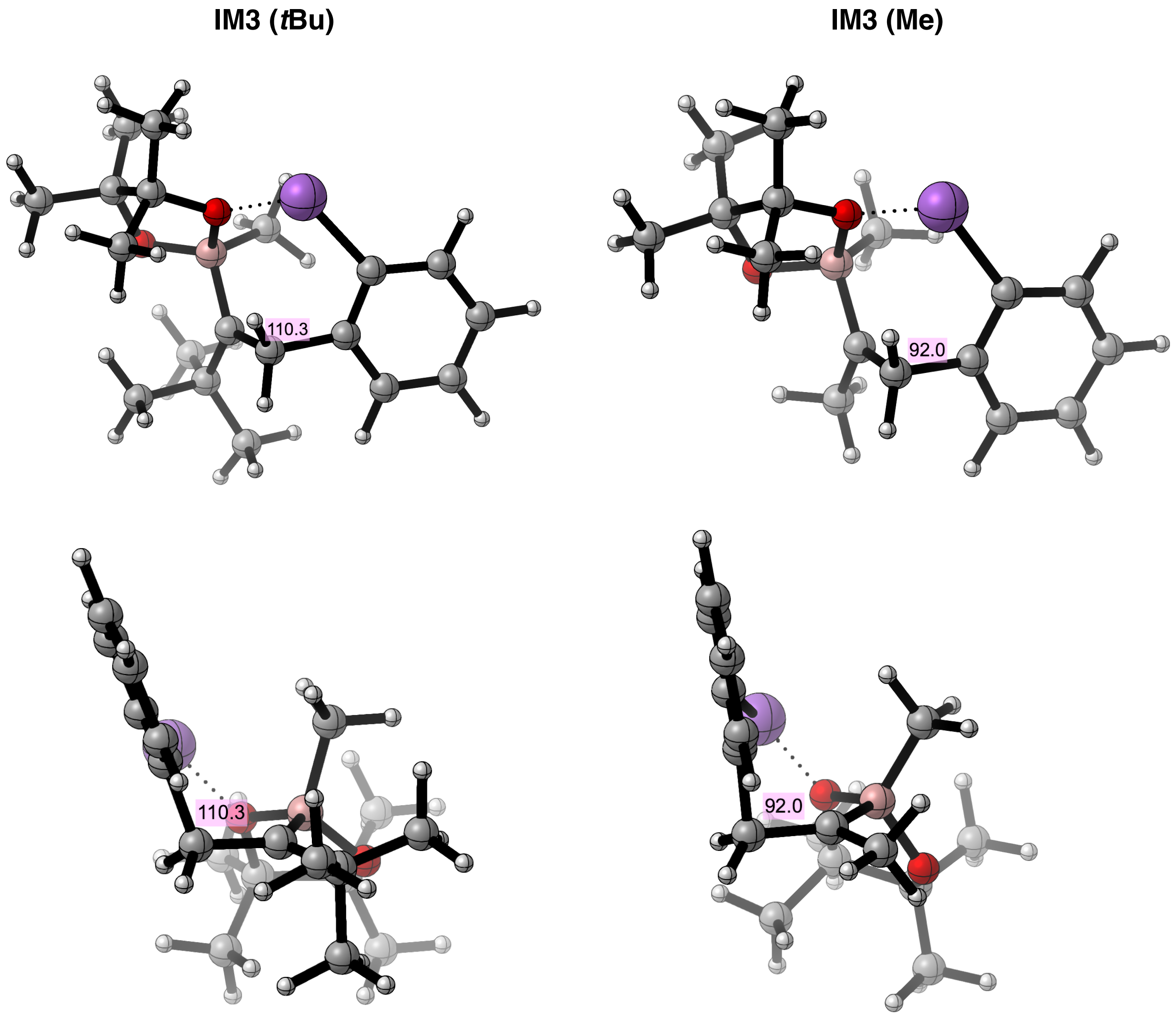

Figure S4 Calculated geometry optimized structures of IM3 (IM3 (*t*Bu) = IM3 structure of the *tert*-butyl substituted vinylboronate derivative, IM3 (Me) = IM3 structure of the methyl substituted vinylboronate derivative) from different view. The numbers highlighted in pink are the calculated bond angles C(aromatic)–C(methylene)–C(carbocation): 110.3 ° for IM3 (*t*Bu), 92.0 ° for IM3 (Me). Structures were shown with following color code: grey: carbon, red: oxygen, pink: boron, purple: lithium.

The calculated structure of carbocation intermediate IM3 (*t*Bu) and IM3 (Me) were also compared. Although both compounds have similar structural properties, there is a significant different in a bond angle C(aromatic)–C(methylene)–C(carbocation). IM3 (*t*Bu) shows a bond angle of 110.3°, a typical angle for sp^3 hybridized carbon. Whereas, IM3 (Me) shows a much smaller bond angle, 92°. Apparently, when large substituent was attached next to the carbocation, it shields the carbocation center sterically, and possibly stabilize the carbocation by hyperconjugation. On the other hand, when substituent of the carbocation was small, C(methylene)–C(phenyl) σ-bond, or π-electron of phenyl group would strongly interact with carbocation, and consequently, phenyl group get closer to the carbocation. This structure seems to be favorable for the cyclization of aryl anion leading to [2+2] adduct.

According to our investigation, the annulative coupling is ineffective when sterically demanding migrating group was used. For example, when phenyl lithium was used as a nucleophile, no coupling product was observed. Therefore, we calculated the energy profile of the rearrangement of the phenyl substituted ate-complex.

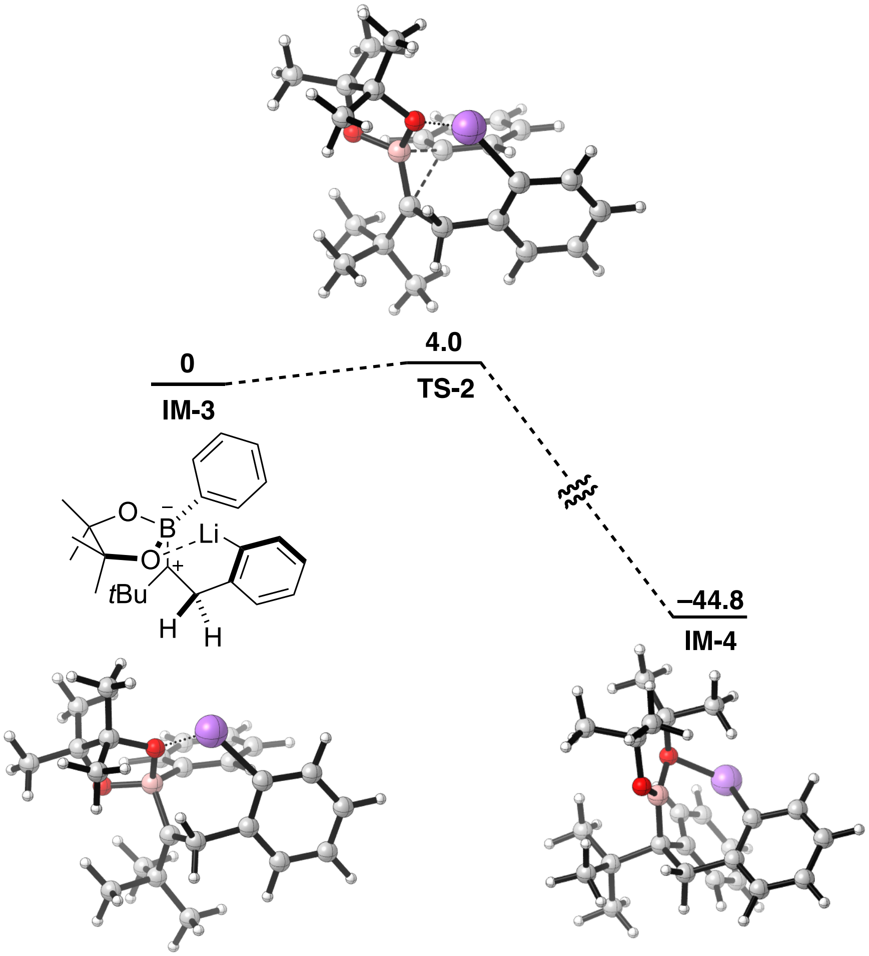

Figure S5 Calculated reaction coordinate for the 1,2-metallate rearrangement. Optimized geometry calculated using DFT (B3LYP-D3BJ/6-31G(d); SMD solvation model with Et_2O). ΔG values are in kcal/mol, calculated using DFT (B3LYP-D3BJ/6-311+G(d,p); SMD solvation model with Et_2O). Calculated structures were shown with following color code: grey: carbon, red: oxygen, pink: boron, purple: lithium.

The calculated energy barrier of the rearrangement was 4.0 kcal/mol. Although the barrier is higher than that of methyl group (0.5 kcal/mol), it seems enough small for the reaction progress under our reaction conditions. Therefore, we hypothesize that the initial step (IM2 to IM3, addition of vinyl group to benzyne) is difficult because of the steric repulsion between phenyl group and benzyne. To verify this,

we tried to find a transition state structure for addition step and calculate an activation barrier. However, we could not find the transition state structure.

The Cartesian coordinates for the optimized geometries are listed, below, together with the following energies (all in Hartree):

B3LYP-D3BJ/6-31G(d)/SMD(Et_2O) electronic energy ($E_{6\text{-}31G}$)

B3LYP-D3BJ/ 6-31G(d)/SMD(Et_2O) Gibbs free energy ($G_{6\text{-}31G}$),

along with the solution-phase single-point energies at the B3LYP-D3BJ/6-311+G(d,p) of theory in Et_2O:

B3LYP-D3BJ/6-311+G(d,p)/SMD(Et_2O) electronic energy ($E_{6\text{-}311+G}$)

B3LYP-D3BJ/ 6-311+G(d,p)/SMD(Et_2O) Gibbs free energy ($G_{6\text{-}311+G}$)

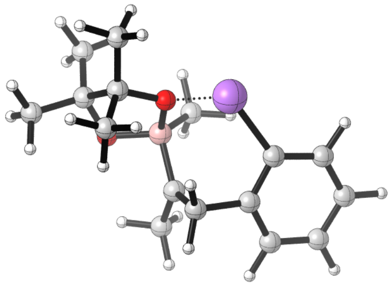

IM-3 (Me)

C	1.177854	0.637260	-1.491952
C	0.568285	1.148232	-0.279213
B	-0.507124	0.330869	0.663365
O	-0.949975	-0.970941	0.027980
C	-2.258618	-0.807446	-0.549920
C	-2.851030	0.363610	0.311006
O	-1.715325	1.182411	0.576381
C	-3.436263	-0.134028	1.643301
C	-3.899569	1.206255	-0.414235
C	-2.114604	-0.426339	-2.028386
C	-3.007361	-2.132673	-0.436527
C	0.081373	0.079955	2.156390
H	0.641190	-0.176962	-1.972281
H	1.507334	1.390241	-2.212298
H	-4.390821	-0.653377	1.501230
H	-2.748032	-0.809654	2.156955
H	-3.609758	0.729949	2.293068
H	-4.754541	0.596121	-0.728182
H	-4.272194	1.986125	0.259115
H	-3.477893	1.698026	-1.294423
H	-1.639825	0.550903	-2.146992
H	-1.497159	-1.173846	-2.539579
H	-3.088041	-0.397194	-2.529149
H	-4.048602	-2.024681	-0.760587
H	-2.537902	-2.886342	-1.081262

H	-3.000309	-2.509225	0.589371
H	0.264592	1.028616	2.680820
H	-0.628988	-0.488681	2.772009
H	1.033366	-0.469053	2.158039
Li	0.457118	-2.105535	-0.071306
C	4.673841	0.384074	0.060310
C	4.680166	-0.941830	0.510878
C	3.542085	-1.740163	0.362247
C	3.525103	0.886364	-0.538331
H	5.555046	1.010902	0.168227
H	5.573582	-1.343539	0.986081
H	3.597543	-2.762467	0.741190
H	3.512966	1.906779	-0.913503
C	2.401677	0.049459	-0.693870
C	2.336093	-1.284808	-0.218303
C	0.861588	2.546111	0.128882
H	-0.118160	3.047378	0.135134
H	1.223306	2.598094	1.161664
H	1.538758	3.089252	-0.535484

0 imaginary frequencies

$E_{6\text{-}31G}$ = -807.146707

$G_{6\text{-}31G}$ = -806.821938

$E_{6\text{-}311+G}$ = -807.362863

$G_{6\text{-}311+G}$ = -807.038094

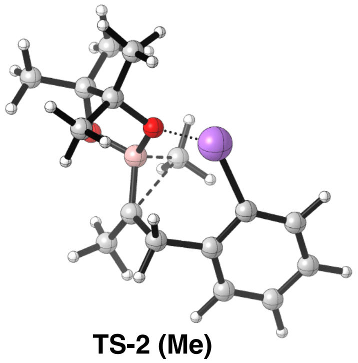

TS-2 (Me)

C	1.282646	0.300773	-1.333150
C	0.380211	0.857890	-0.301803
B	-0.687563	-0.005143	0.488767
O	-1.028283	-1.315074	-0.145163
C	-2.394511	-1.229154	-0.604776
C	-3.020043	-0.264662	0.461493
O	-1.961679	0.657409	0.726754
C	-3.384440	-0.998421	1.761655
C	-4.232211	0.517206	-0.040757
C	-2.387883	-0.623395	-2.012893
C	-2.988038	-2.632512	-0.640076
C	0.219532	-0.243580	1.886317
H	0.729829	-0.399413	-1.965430
H	1.695612	1.106389	-1.950659
H	-4.277672	-1.622711	1.645928
H	-2.562084	-1.631888	2.106242
H	-3.585754	-0.253569	2.538393
H	-5.039423	-0.156219	-0.351794
H	-4.616700	1.154863	0.762866
H	-3.969106	1.162181	-0.882880
H	-1.991592	0.396606	-2.005018
H	-1.755243	-1.233453	-2.667153
H	-3.393612	-0.596351	-2.444471
H	-4.056943	-2.597897	-0.877910

H	-2.497005	-3.231695	-1.417175
H	-2.864225	-3.144009	0.318120
H	0.232275	0.668451	2.492314
H	-0.345074	-1.011050	2.432989
H	1.246381	-0.597212	1.766602
Li	0.361237	-2.480867	-0.340480
C	4.727457	-0.379538	0.172572
C	4.645043	-1.741597	0.466131
C	3.463341	-2.441738	0.191787
C	3.625830	0.258555	-0.400147
H	5.637874	0.179338	0.375858
H	5.497144	-2.252766	0.911701
H	3.443725	-3.503786	0.446990
H	3.687225	1.315752	-0.654637
C	2.462521	-0.474883	-0.670741
C	2.311588	-1.846489	-0.370664
C	0.611509	2.261439	0.095129
H	0.245499	2.863637	-0.754922
H	0.058621	2.562698	0.985668
H	1.677951	2.502312	0.195044

1 imaginary frequencies

$E_{6\text{-}31G}$ = -807.142461

$G_{6\text{-}31G}$ = -806.819112

$E_{6\text{-}311+G}$ = -807.359468

$G_{6\text{-}311+G}$ = -807.036119

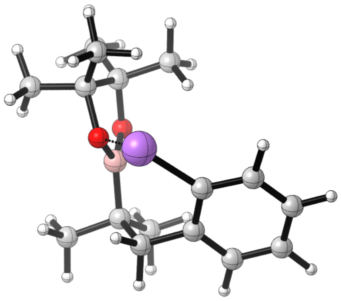

IM-4 (Me)

C	1.569335	1.207445	-0.707627
C	0.373689	1.341744	0.281507
B	-0.600956	0.105001	0.237863
O	-0.998246	-0.534470	-0.947726
C	-2.187490	-1.351182	-0.630901
C	-1.998319	-1.591792	0.906096
O	-1.261164	-0.399593	1.317732
C	-1.103566	-2.789368	1.229565
C	-3.294709	-1.658396	1.700532
C	-3.399510	-0.489907	-0.976505
C	-2.151970	-2.604025	-1.494737
C	0.879646	1.576239	1.714705
H	1.167728	1.133333	-1.729178
H	2.123404	2.157070	-0.671369
H	-1.604179	-3.736035	1.003237
H	-0.157807	-2.746673	0.678853
H	-0.867366	-2.773393	2.297676
H	-3.907978	-2.501946	1.365520
H	-3.066823	-1.805506	2.760798
H	-3.878016	-0.740097	1.602154
H	-3.425229	0.422233	-0.372616
H	-3.347408	-0.199994	-2.030646
H	-4.332055	-1.039494	-0.817662
H	-3.001013	-3.253636	-1.258974
H	-2.230299	-2.331480	-2.553808

H	-1.239249	-3.189669	-1.343348
H	0.045094	1.707004	2.412514
H	1.484298	0.736768	2.071028
H	1.501311	2.480814	1.760379
Li	0.419722	-1.324346	-1.912059
C	4.548830	-0.764353	0.617142
C	4.303053	-2.034479	0.095138
C	3.168653	-2.244779	-0.700036
C	3.654054	0.270597	0.336168
H	5.427711	-0.577148	1.230671
H	4.993405	-2.851654	0.301309
H	3.019936	-3.253711	-1.094688
H	3.847029	1.266406	0.734695
C	2.517849	0.043162	-0.456815
C	2.232532	-1.234884	-1.015057
C	-0.476464	2.561181	-0.157698
H	-1.334157	2.718491	0.508413
H	0.131979	3.475428	-0.134265
H	-0.859629	2.442082	-1.178570

0 imaginary frequencies

$E_{6\text{-}31G}$ = -807.227497

$G_{6\text{-}31G}$ = -806.898272

$E_{6\text{-}311+G}$ = -807.441922

$G_{6\text{-}311+G}$ = -807.112697

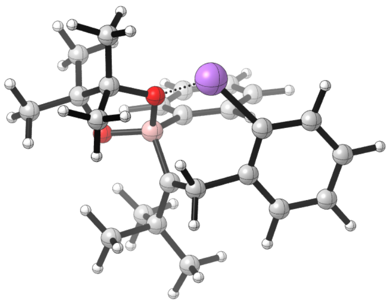

IM-3 (Ph)

C	0.909708	-0.151481	-1.962139
C	0.163463	0.753641	-1.058660
C	0.169186	2.218510	-1.344878
B	-0.783199	0.133321	0.146126
C	1.455676	2.805320	-1.952549
C	-0.963909	2.263606	-2.445492
C	-0.270176	3.115390	-0.175225
O	-1.003994	-1.345886	-0.024963
C	-2.346253	-1.580618	-0.499849
C	-3.110407	-0.345147	0.083132
O	-2.136869	0.698649	0.008827
C	-3.524547	-0.562074	1.546645
C	-4.333078	0.078314	-0.728624
C	-2.342248	-1.601845	-2.033249
C	-2.808532	-2.936701	0.026742
H	0.286481	-1.022571	-2.167765
H	1.176688	0.332696	-2.903894
H	1.267445	3.851032	-2.217992
H	2.271085	2.784191	-1.224097

H	1.781972	2.291373	-2.859771
H	-1.071320	3.317410	-2.726423
H	-1.910775	1.901932	-2.043479
H	-0.694442	1.694461	-3.339448
H	-1.235375	2.807506	0.225173
H	0.468266	3.092083	0.630740
H	-0.353547	4.148323	-0.531322
H	-4.330555	-1.299209	1.631254
H	-2.681381	-0.893376	2.157810
H	-3.888895	0.384873	1.957252
H	-5.082174	-0.721174	-0.763008
H	-4.796169	0.953880	-0.260897
H	-4.064026	0.347571	-1.752936
H	-2.060214	-0.632132	-2.451708
H	-1.623370	-2.350098	-2.385945
H	-3.326906	-1.871030	-2.429039
H	-3.862608	-3.113099	-0.214218
H	-2.227568	-3.741085	-0.443226
H	-2.683107	-3.009563	1.109923
Li	0.369282	-2.530770	0.065887
C	4.595922	-0.405222	-0.939630
C	4.629937	-1.636257	-0.283061
C	3.449880	-2.374284	-0.141866
C	3.384203	0.064545	-1.446293
H	5.502495	0.180820	-1.070541
H	5.569905	-2.014463	0.116000

H	3.519068	-3.329574	0.383732
H	3.368625	1.002226	-1.990950
C	2.216290	-0.698701	-1.289394
C	2.189904	-1.941840	-0.616450
C	-0.070202	0.457461	1.587781
C	-0.786222	1.211888	2.535181
C	1.208429	0.014839	1.967859
C	-0.274629	1.481026	3.804770
H	-1.766687	1.589690	2.264645
C	1.732147	0.278071	3.237533
H	1.816072	-0.545936	1.266494
C	0.991605	1.011626	4.164279
H	-0.862413	2.059548	4.514214
H	2.725057	-0.084308	3.494342
H	1.396753	1.222743	5.150878

0 imaginary frequencies

$E_{6\text{-}31G}$ = -1116.865304

$G_{6\text{-}31G}$ = -1116.408292

$E_{6\text{-}311+G}$ = -1117.154663

$G_{6\text{-}311+G}$ = -1116.697651

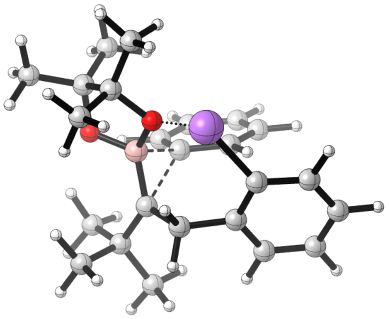

TS-2 (Ph)

C	-1.505444	1.461508	-2.364394
C	-1.743027	1.354034	-1.047353
C	-2.814779	2.221783	-0.364887
B	-0.844659	0.321519	-0.158313
C	-3.243431	3.435969	-1.206748
C	-4.058918	1.338832	-0.116107
C	-2.312075	2.740670	0.998394
O	-0.244546	-0.812715	-1.012875
C	-1.072627	-1.976327	-0.830568
C	-1.494584	-1.830545	0.674417
O	-1.610570	-0.425983	0.859681
C	-0.422114	-2.383559	1.630523
C	-2.835308	-2.485663	1.010673
C	-2.264513	-1.906145	-1.792344
C	-0.253898	-3.224531	-1.147631
H	-0.780960	0.809610	-2.849549
H	-2.014354	2.162479	-3.024660
H	-3.973614	4.039877	-0.653303
H	-2.388035	4.078692	-1.447828

H	-3.716676	3.136762	-2.148719
H	-4.843560	1.912566	0.396341
H	-3.795130	0.476813	0.500658
H	-4.472817	0.975500	-1.064897
H	-2.045541	1.914806	1.663017
H	-1.430477	3.381262	0.882241
H	-3.095982	3.332715	1.489016
H	-0.371566	-3.478178	1.607543
H	0.573479	-1.989769	1.398583
H	-0.669851	-2.076839	2.651993
H	-2.811964	-3.566572	0.826490
H	-3.063735	-2.328956	2.070917
H	-3.648040	-2.049540	0.425562
H	-2.907507	-1.052485	-1.575686
H	-1.892740	-1.794028	-2.816076
H	-2.863264	-2.822034	-1.743648
H	-0.842191	-4.130785	-0.964774
H	0.038918	-3.221729	-2.204101
H	0.653646	-3.283612	-0.542881
Li	1.498974	-0.542975	-0.661794
C	3.959969	-0.943001	0.774168
C	3.555937	-2.254254	0.446317
C	3.296935	-2.662755	-0.883579
C	4.142934	0.065214	-0.201306
H	4.132961	-0.688518	1.815834
H	3.436038	-2.982171	1.244303

H	2.989450	-3.668944	-1.139805
H	4.440466	1.074429	0.048192
C	3.851517	-0.437250	-1.464387
C	3.489641	-1.600161	-1.760708
C	0.435573	1.149643	0.497394
C	1.149283	2.134639	-0.218636
C	0.837101	0.928360	1.829966
C	2.199325	2.856673	0.358835
H	0.844881	2.363779	-1.237882
C	1.879518	1.647262	2.420153
H	0.285691	0.202494	2.419469
C	2.565959	2.617245	1.684799
H	2.718452	3.616806	-0.220732
H	2.147265	1.464006	3.458624
H	3.370069	3.187344	2.143469

1 imaginary frequencies

$E_{6\text{-}31G}$ = -1116.859735

$G_{6\text{-}31G}$ = -1116.401827

$E_{6\text{-}311+G}$ = -1117.149150

$G_{6\text{-}311+G}$ = -1116.691242

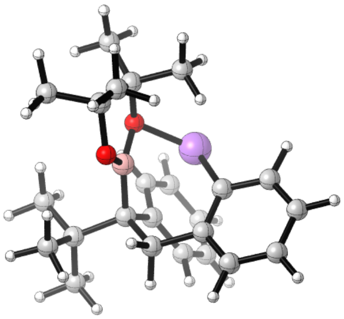

IM-4 (Ph)

C	1.176618	0.920795	-1.340843
C	0.267982	1.213282	-0.112678
C	-0.302504	2.716133	-0.199891
B	-0.930658	0.167282	-0.249956
C	0.794169	3.743989	0.137127
C	-0.835331	3.044441	-1.609661
C	-1.460892	2.923509	0.793841
O	-1.432391	-0.632654	0.782404
C	-2.449370	-1.535810	0.219068
C	-2.814263	-0.822844	-1.138875
O	-1.652254	0.013899	-1.398159
C	-4.017468	0.116496	-1.035750
C	-2.988598	-1.766980	-2.322153
C	-1.790720	-2.903439	0.041515
C	-3.587704	-1.628536	1.225906
H	0.556974	1.027621	-2.236303
H	1.948798	1.694730	-1.436480
H	0.403678	4.758236	-0.011508

H	1.126353	3.664735	1.176391
H	1.672722	3.633732	-0.507347
H	-1.327794	4.024232	-1.588355
H	-1.562915	2.306132	-1.954664
H	-0.031377	3.103296	-2.349383
H	-2.296960	2.241075	0.599835
H	-1.143570	2.787175	1.830872
H	-1.848769	3.945242	0.703989
H	-4.947358	-0.441973	-0.889863
H	-3.907363	0.828469	-0.212852
H	-4.102963	0.685924	-1.966268
H	-3.823829	-2.454573	-2.151052
H	-3.208805	-1.183632	-3.221798
H	-2.084072	-2.349545	-2.508912
H	-0.923691	-2.851423	-0.620843
H	-1.457579	-3.274747	1.019225
H	-2.503224	-3.631644	-0.357072
H	-4.401091	-2.245109	0.828875
H	-3.226557	-2.100477	2.145888
H	-3.987199	-0.644874	1.481591
Li	0.365135	-1.290523	1.223604
C	3.384997	-1.907302	-2.578202
C	3.025896	-2.962216	-1.737592
C	2.069457	-2.743438	-0.738345
C	2.776076	-0.663940	-2.403130
H	4.127130	-2.049131	-3.361311

H	3.489898	-3.940598	-1.858377
H	1.815487	-3.598257	-0.102393
H	3.045965	0.159824	-3.065790
C	1.820480	-0.463200	-1.393795
C	1.434910	-1.499992	-0.506977
C	1.048048	1.043763	1.202877
C	0.401796	0.815783	2.434938
C	2.452299	1.137498	1.235659
C	1.120941	0.650400	3.626004
H	-0.678719	0.760526	2.475089
C	3.170001	0.986914	2.418807
H	3.000134	1.310922	0.318573
C	2.510951	0.730412	3.624235
H	0.581273	0.472033	4.552567
H	4.254060	1.059764	2.396662
H	3.073543	0.603845	4.544884

0 imaginary frequencies

$E_{6\text{-}31G}$ = -1116.941129

$G_{6\text{-}31G}$ = -1116.480897

$E_{6\text{-}311+G}$ = -1117.229304

$G_{6\text{-}311+G}$ = -1116.769072

^{1}H and ^{13}C NMR Spectra of New Compounds

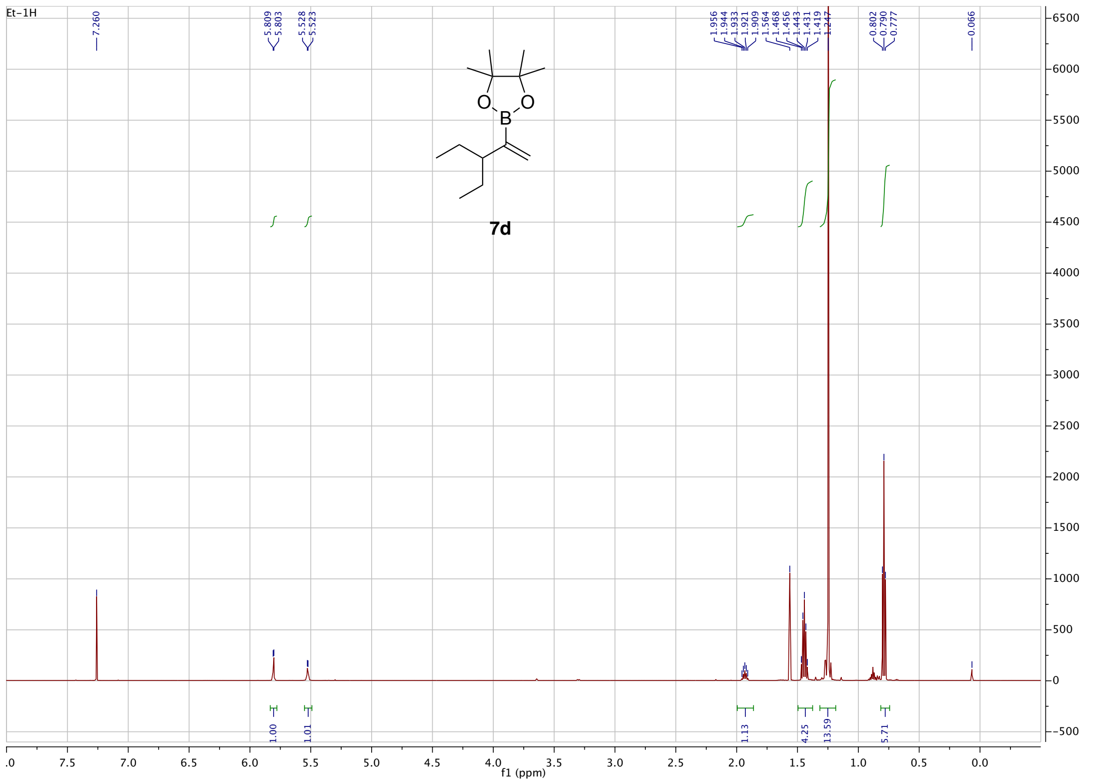

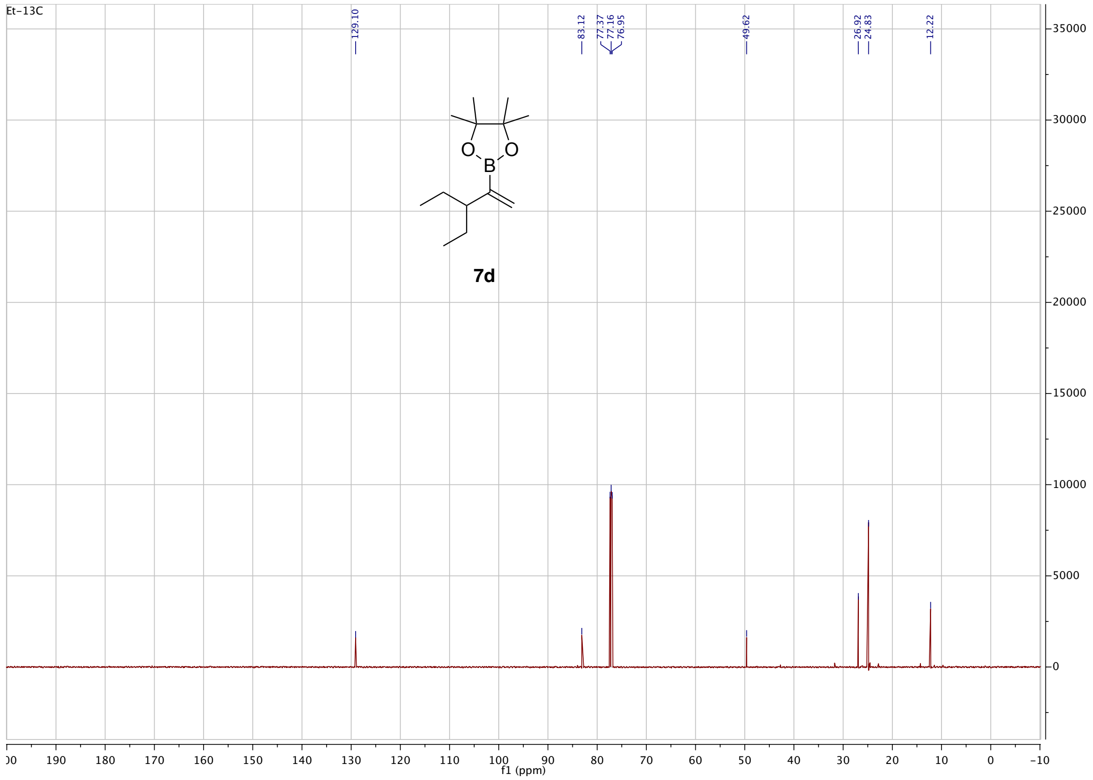

Figure S6. ^{1}H and ^{13}C NMR Spectra of **7d**

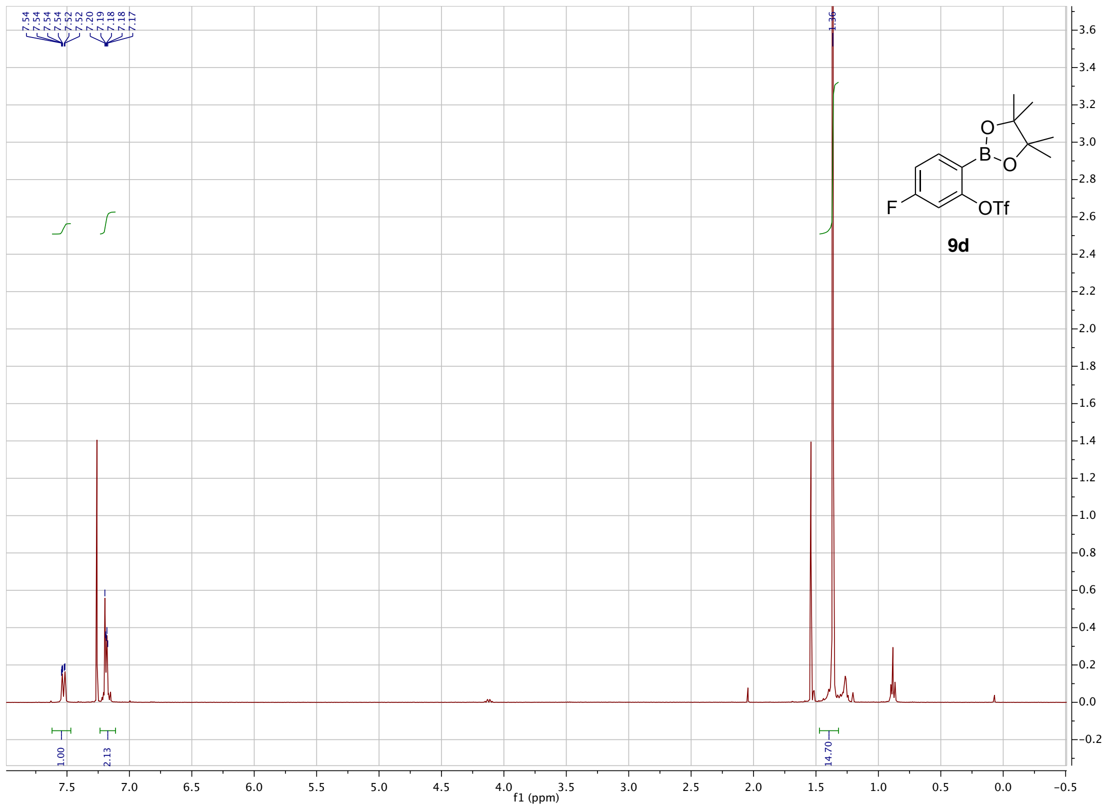

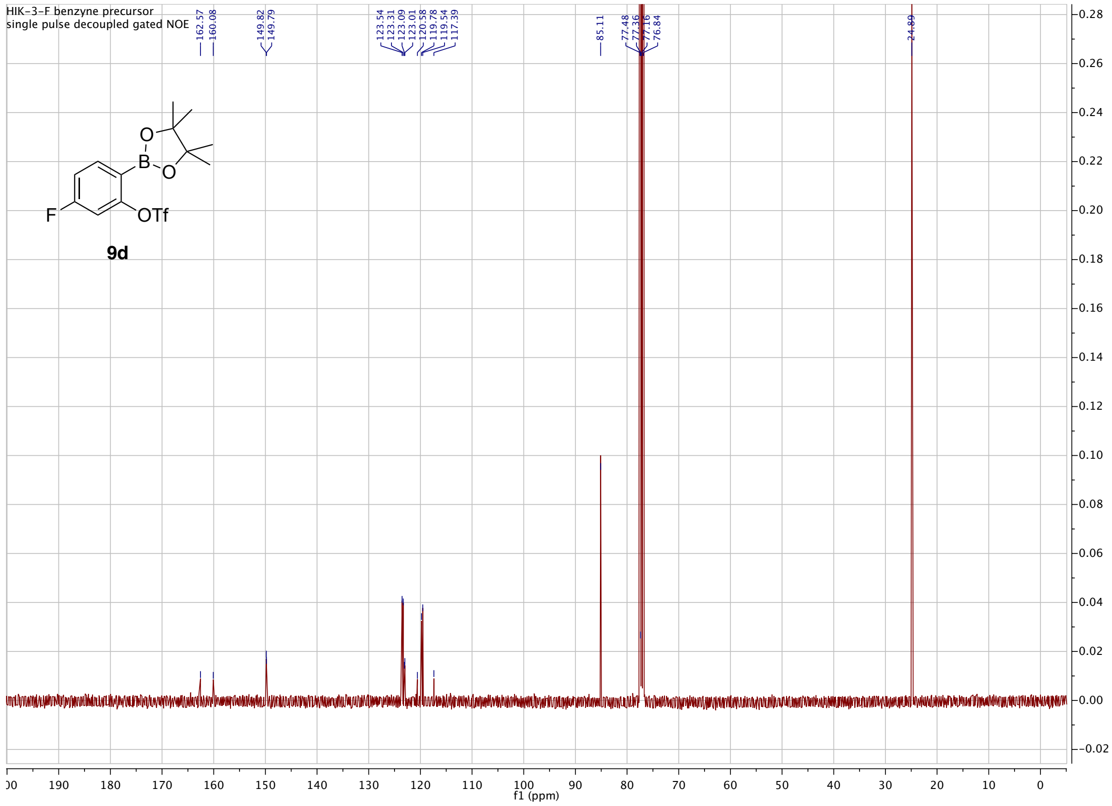

Figure S7. ^{1}H and ^{13}C NMR Spectra of **9d**

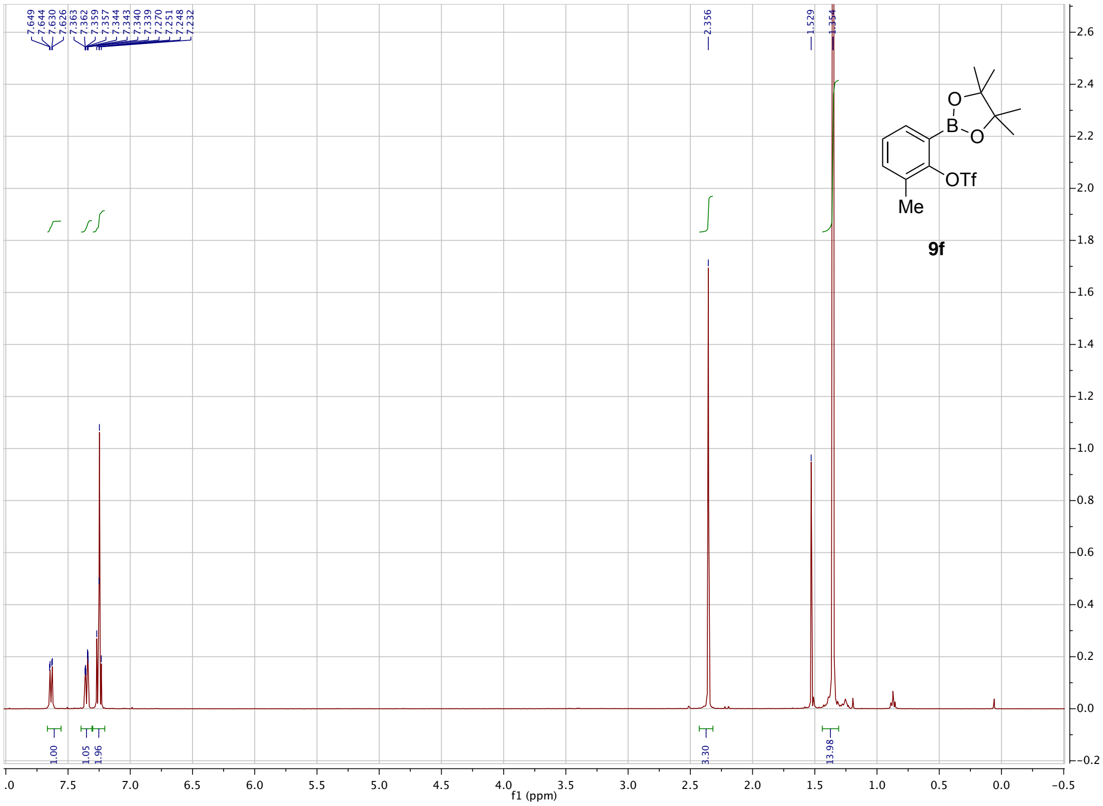

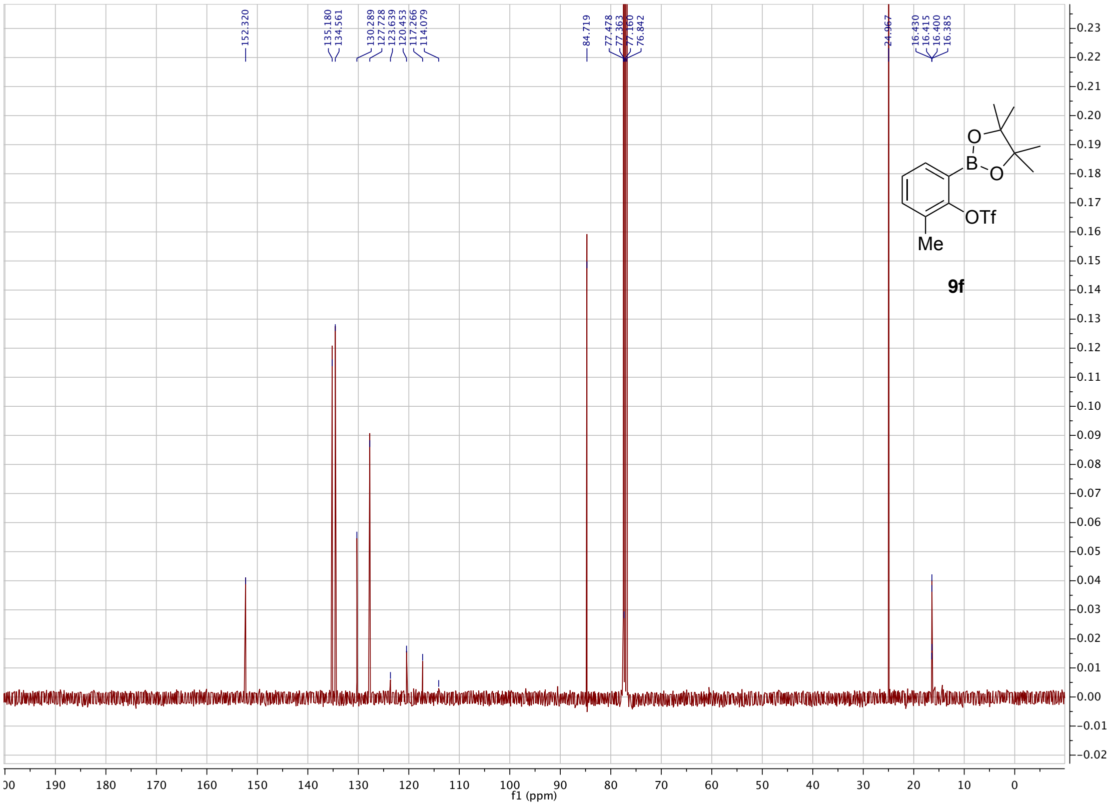

Figure S8. ^{1}H and ^{13}C NMR Spectra of **9f**

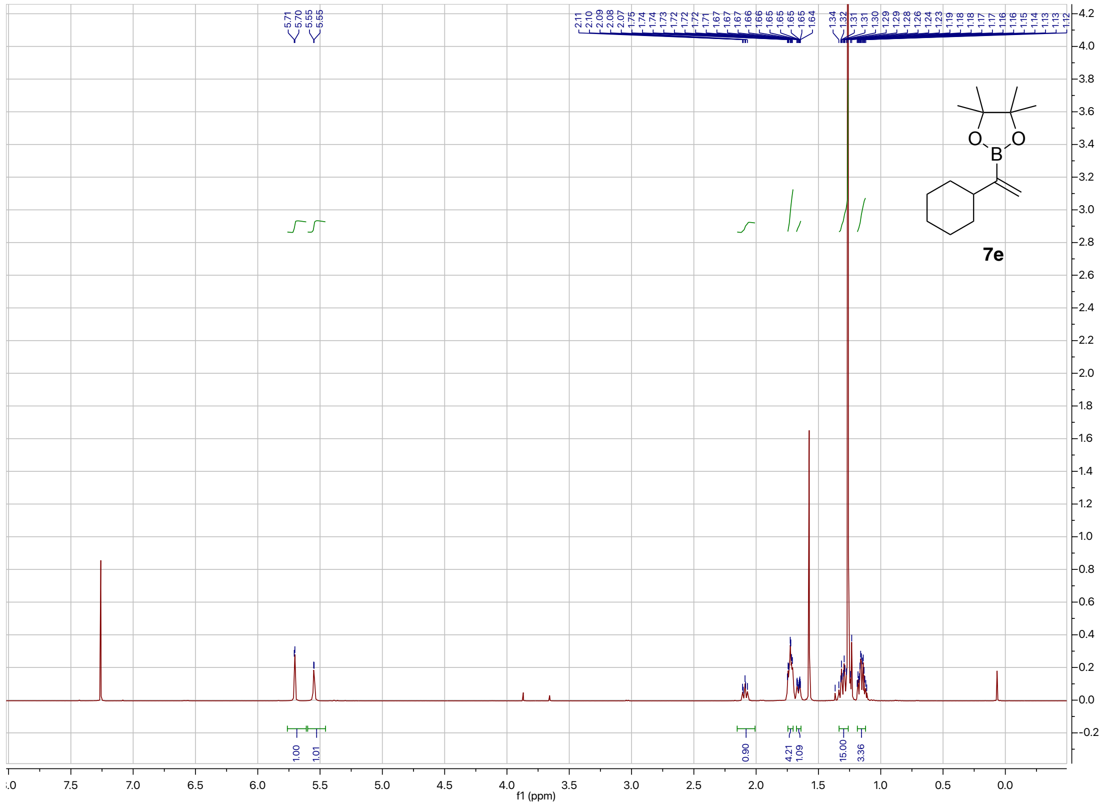

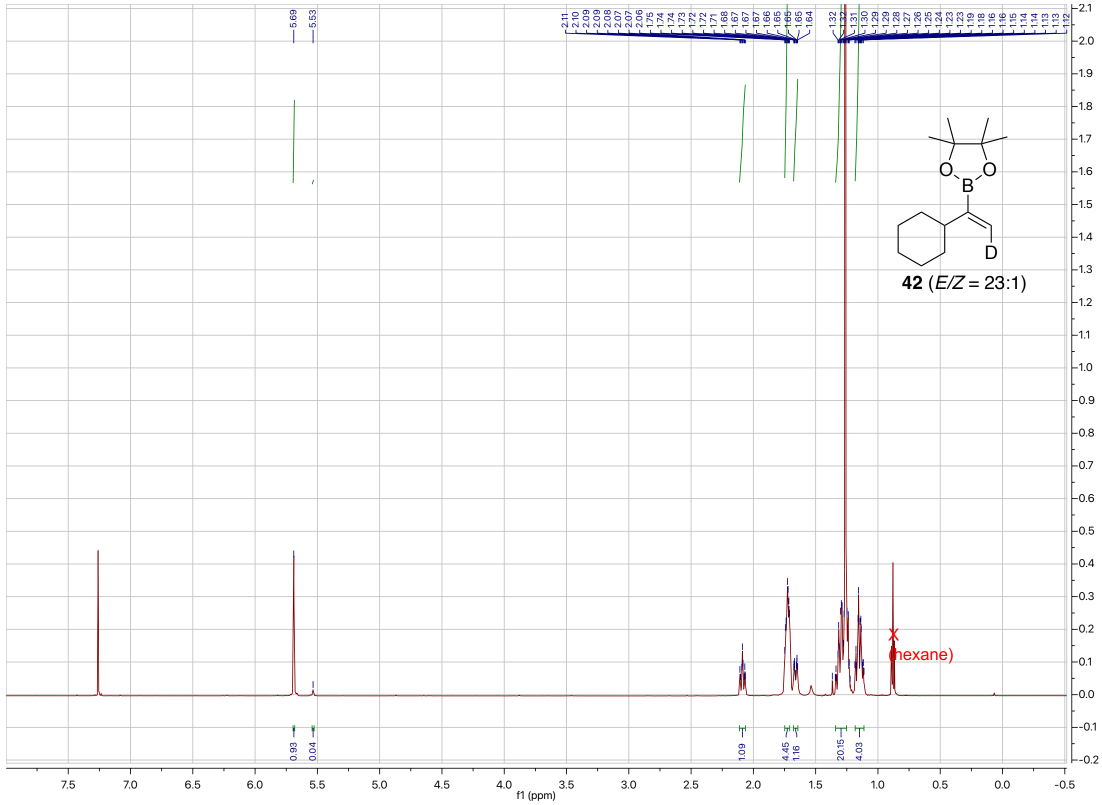

Figure S9. ^{1}H NMR Spectra of **7e** and **42** (*E*/*Z* = 23:1)

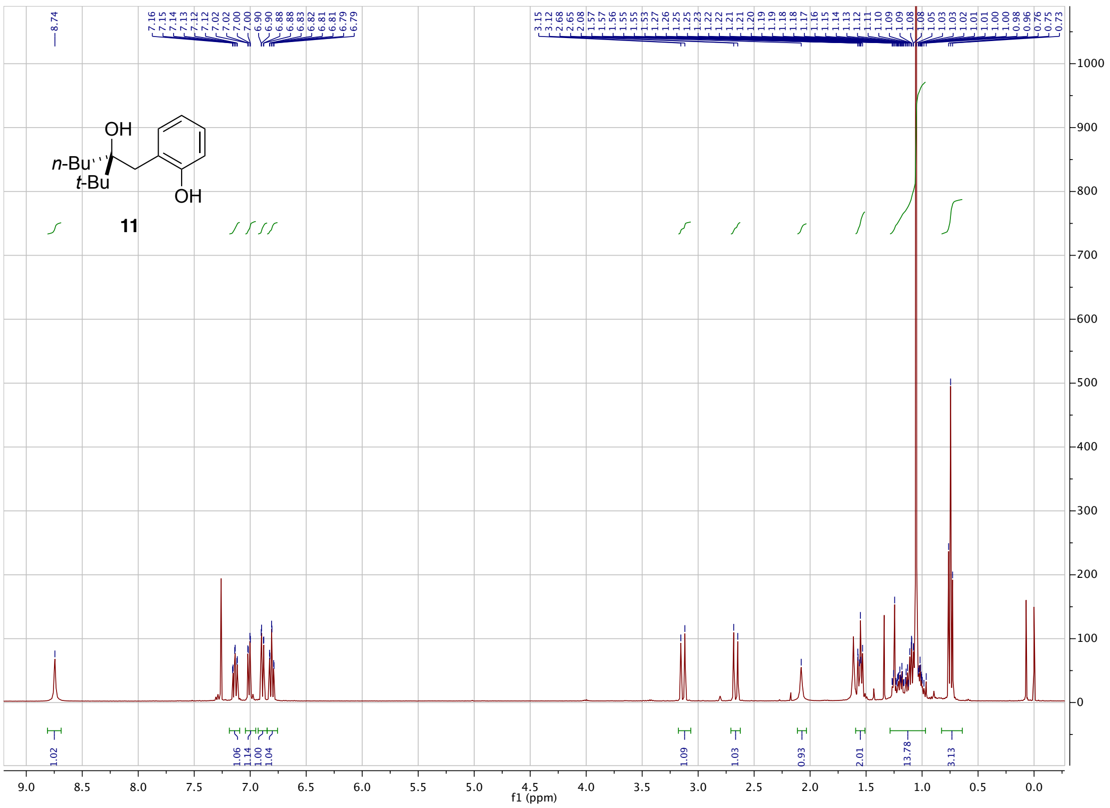

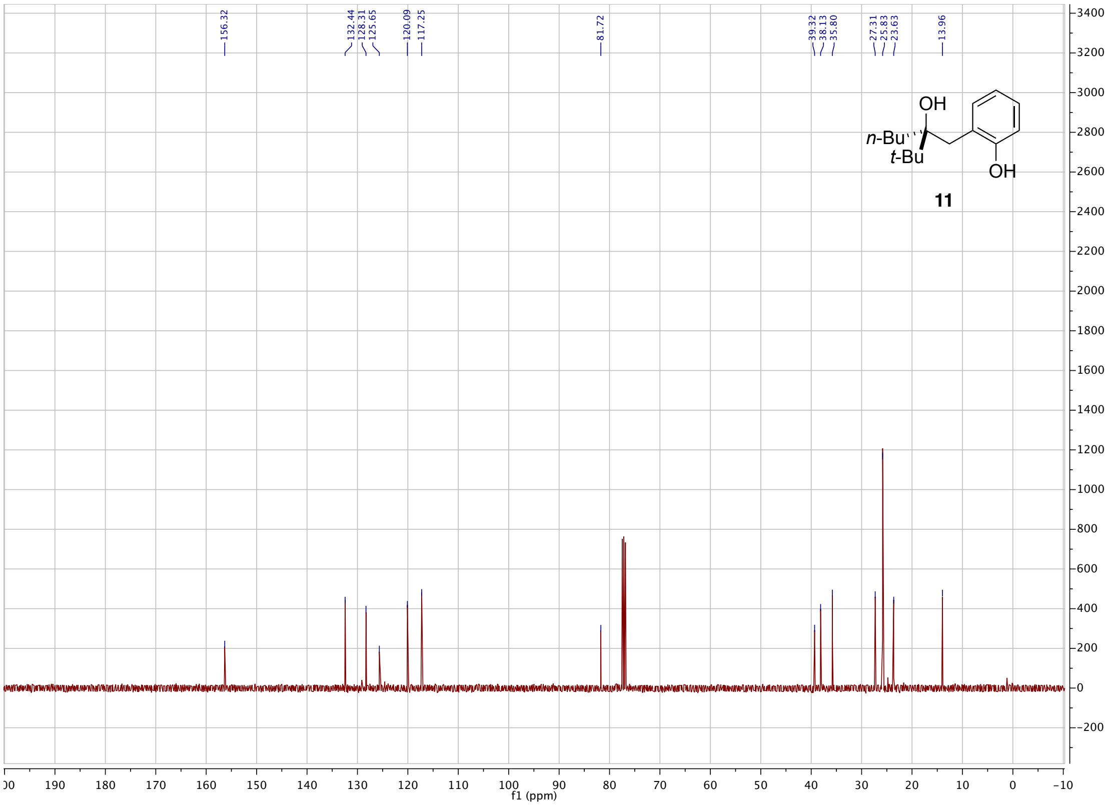

Figure S10. ^{1}H and ^{13}C NMR Spectra of **11**

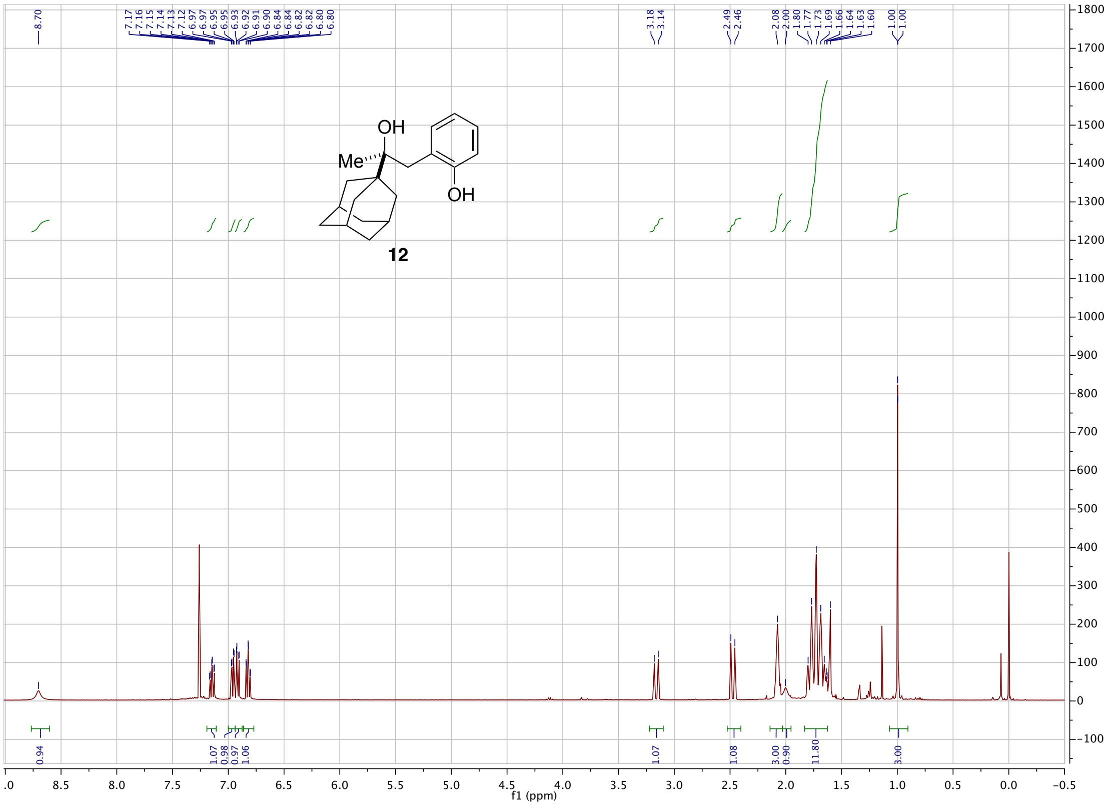

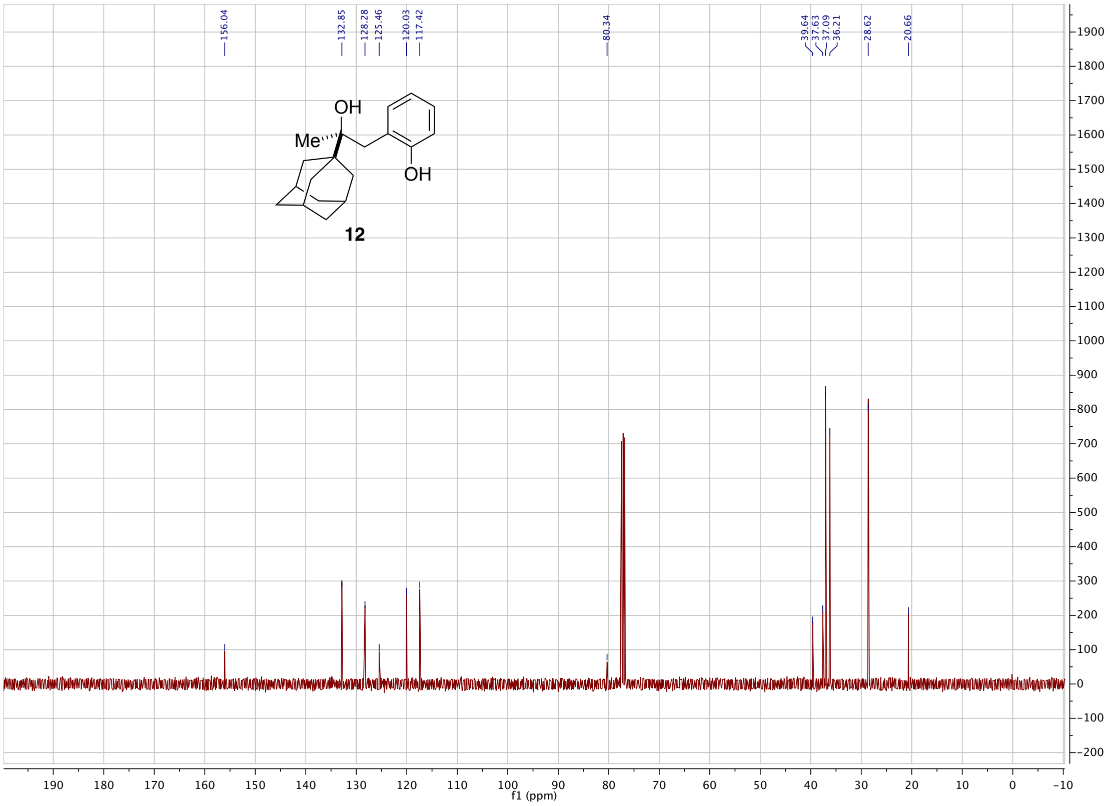

Figure S11. ^{1}H and ^{13}C NMR Spectra of **12**

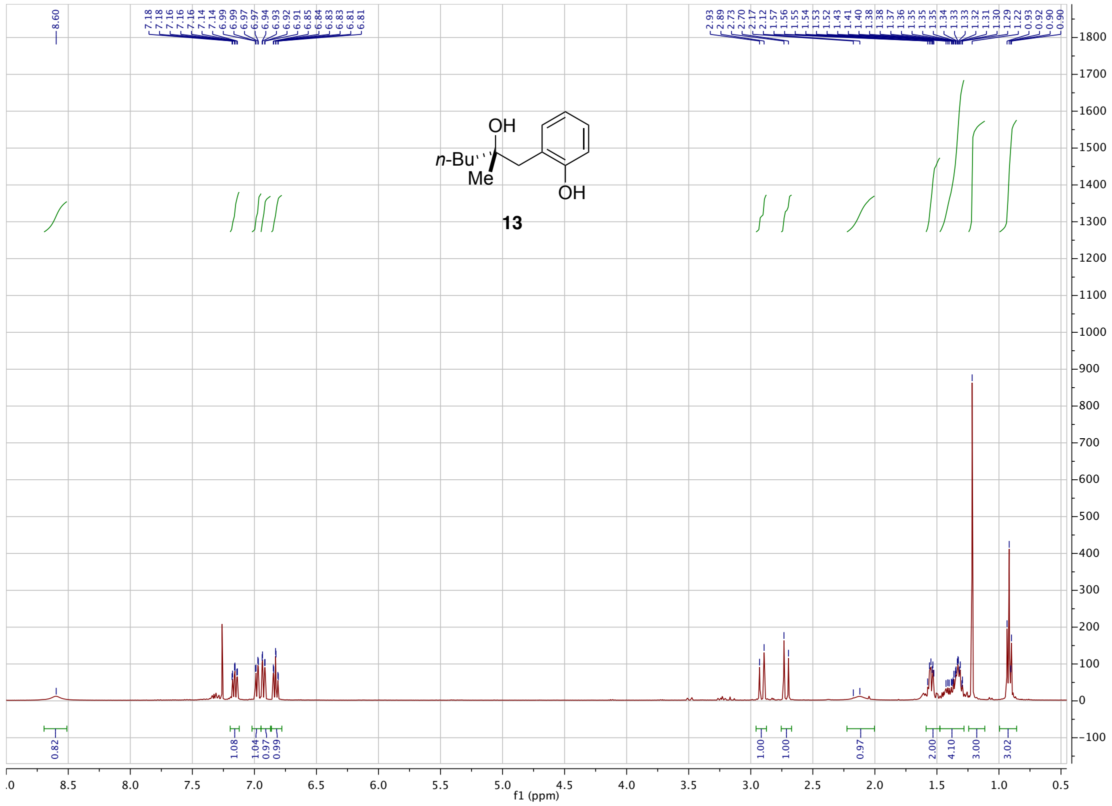

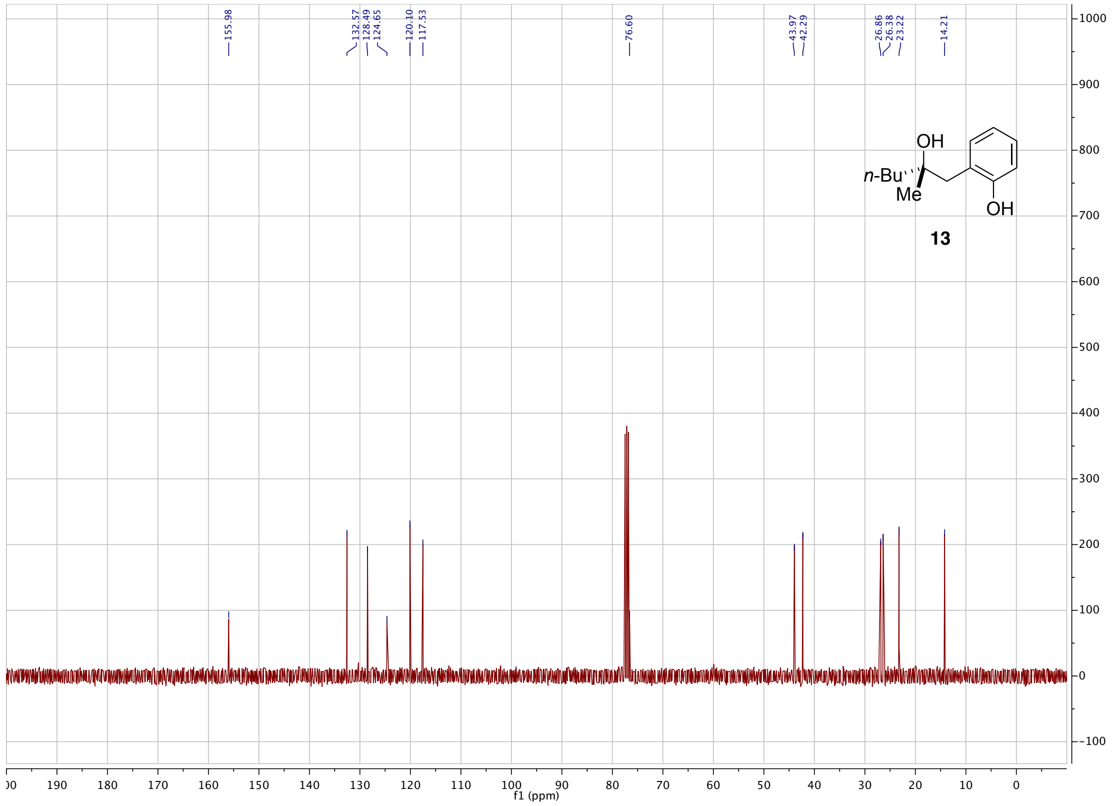

Figure S12. ^{1}H and ^{13}C NMR Spectra of **13**

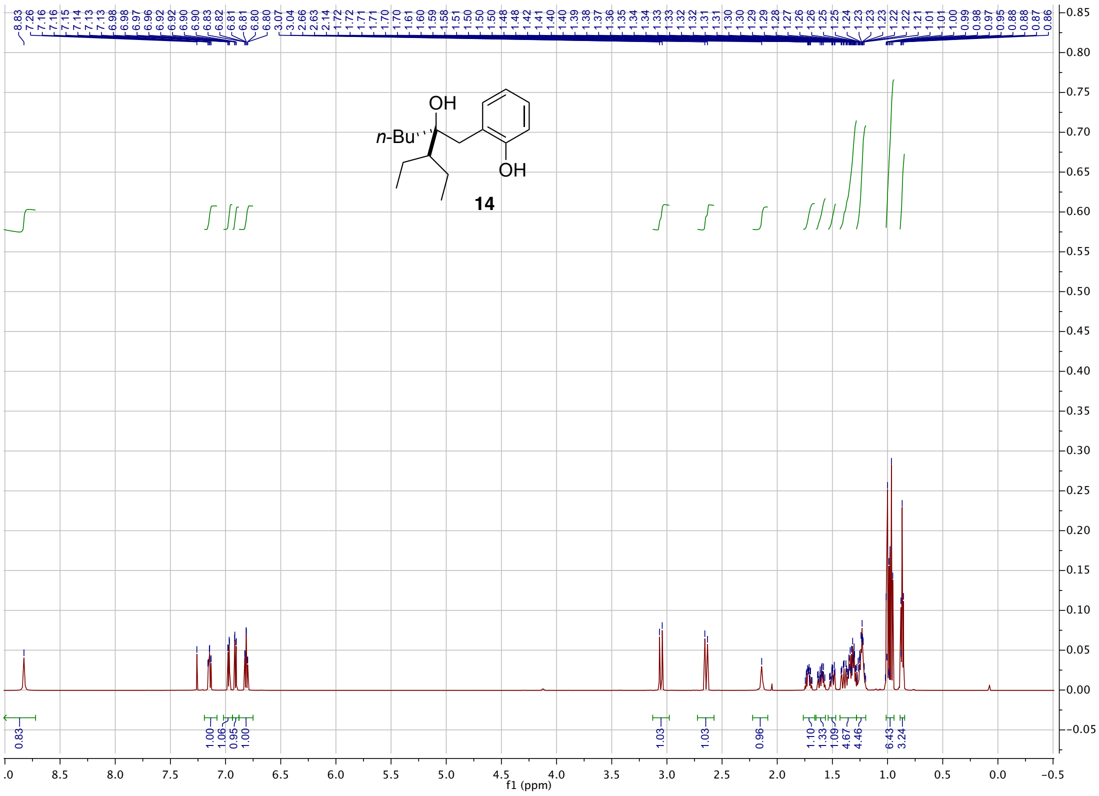

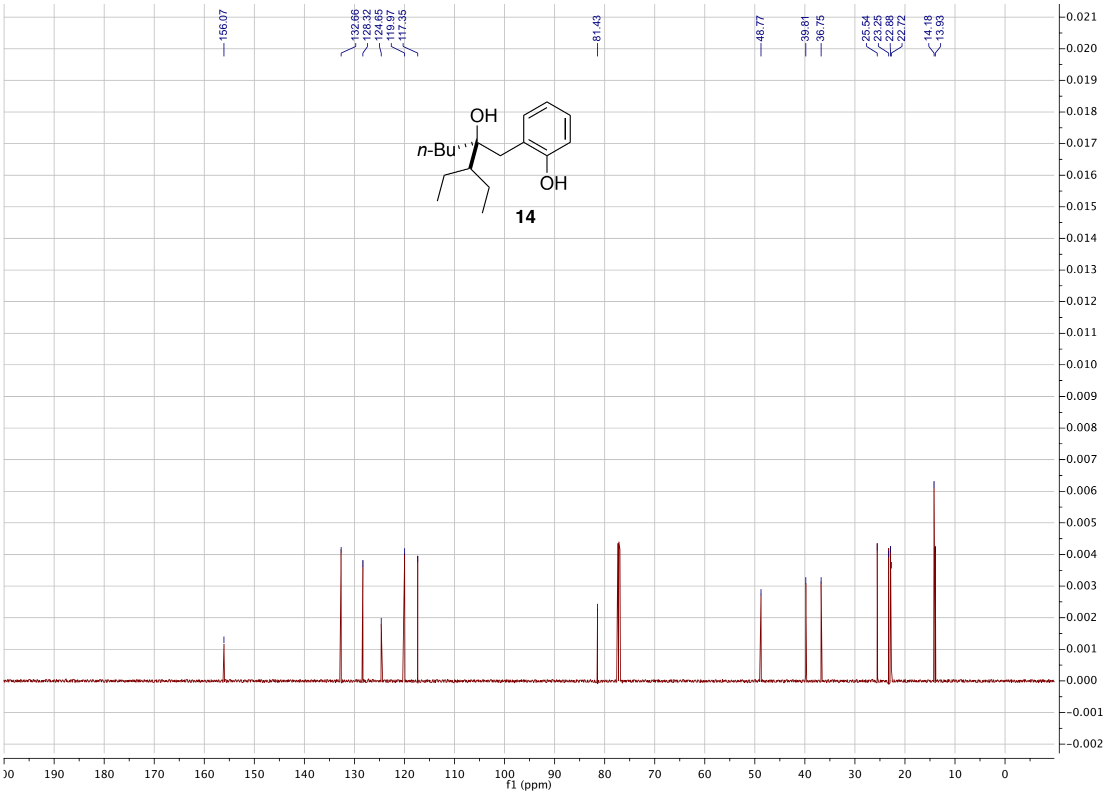

Figure S13. ^{1}H and ^{13}C NMR Spectra of **14**

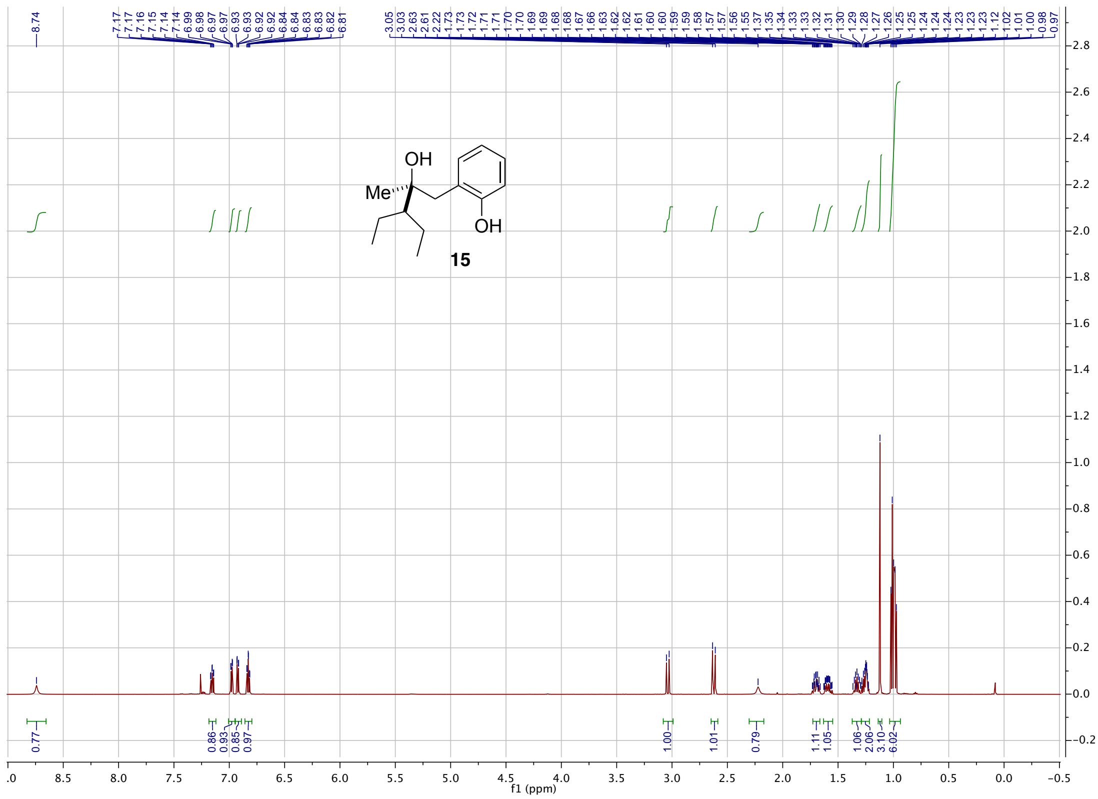

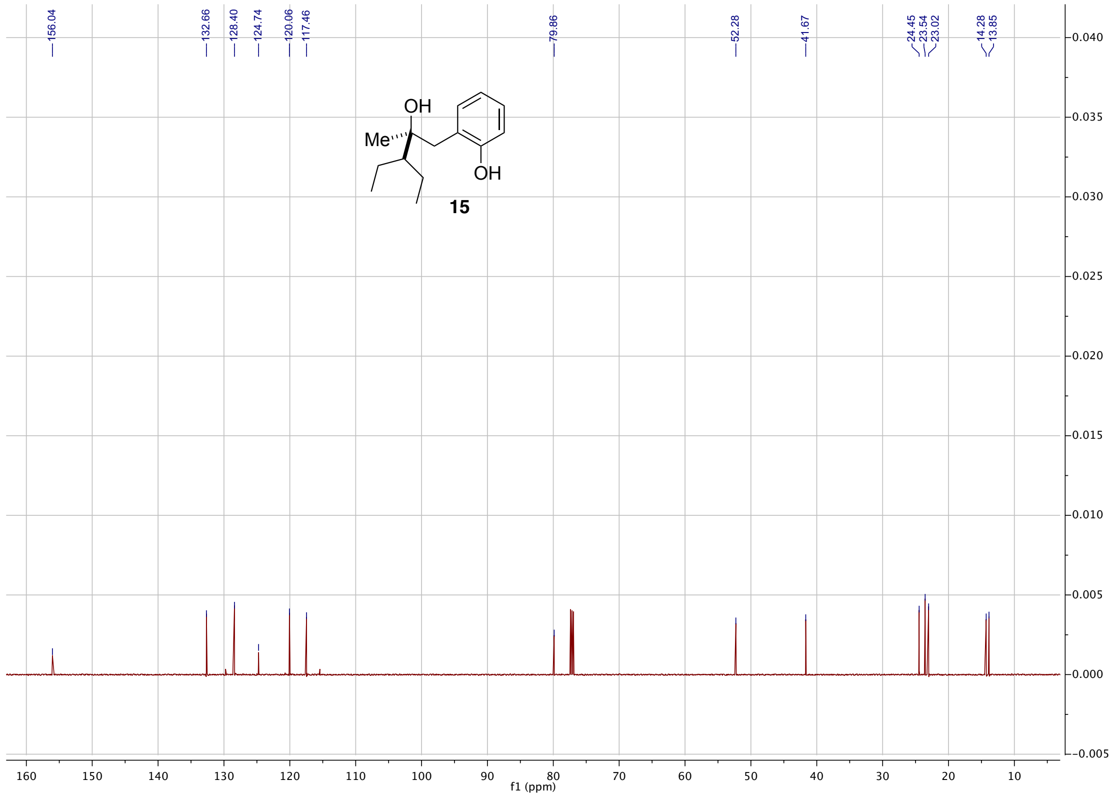

Figure S14. ^{1}H and ^{13}C NMR Spectra of **15**

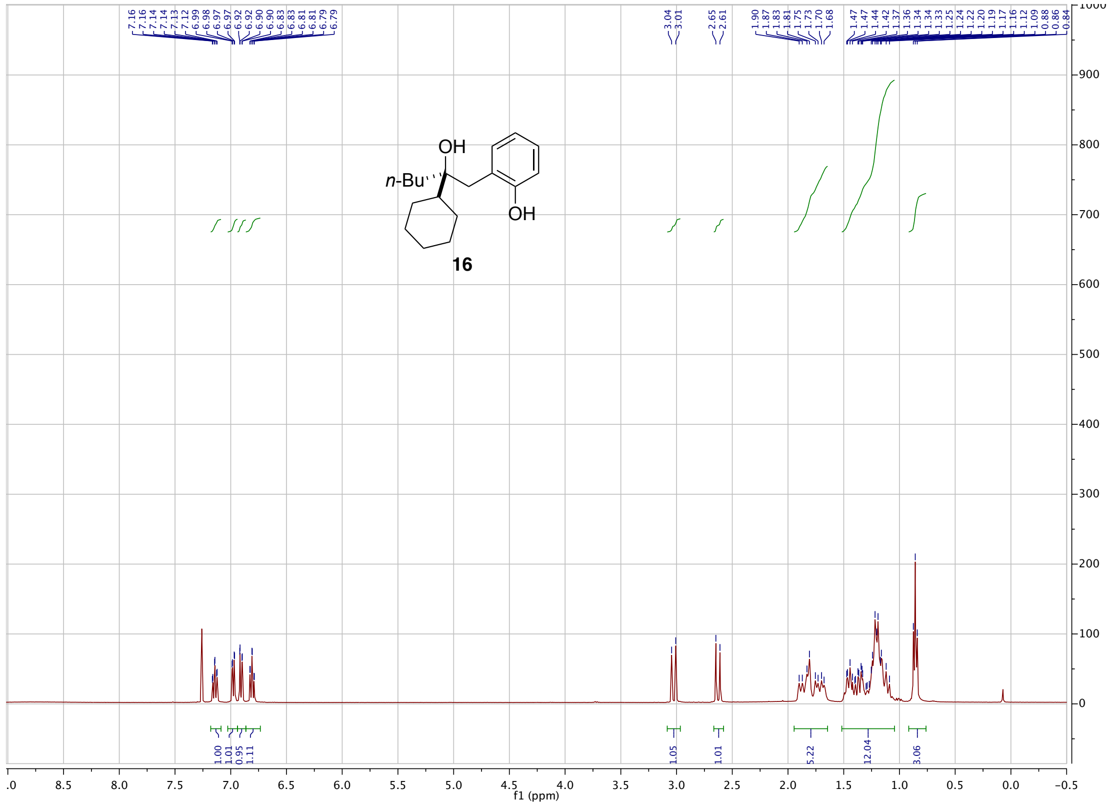

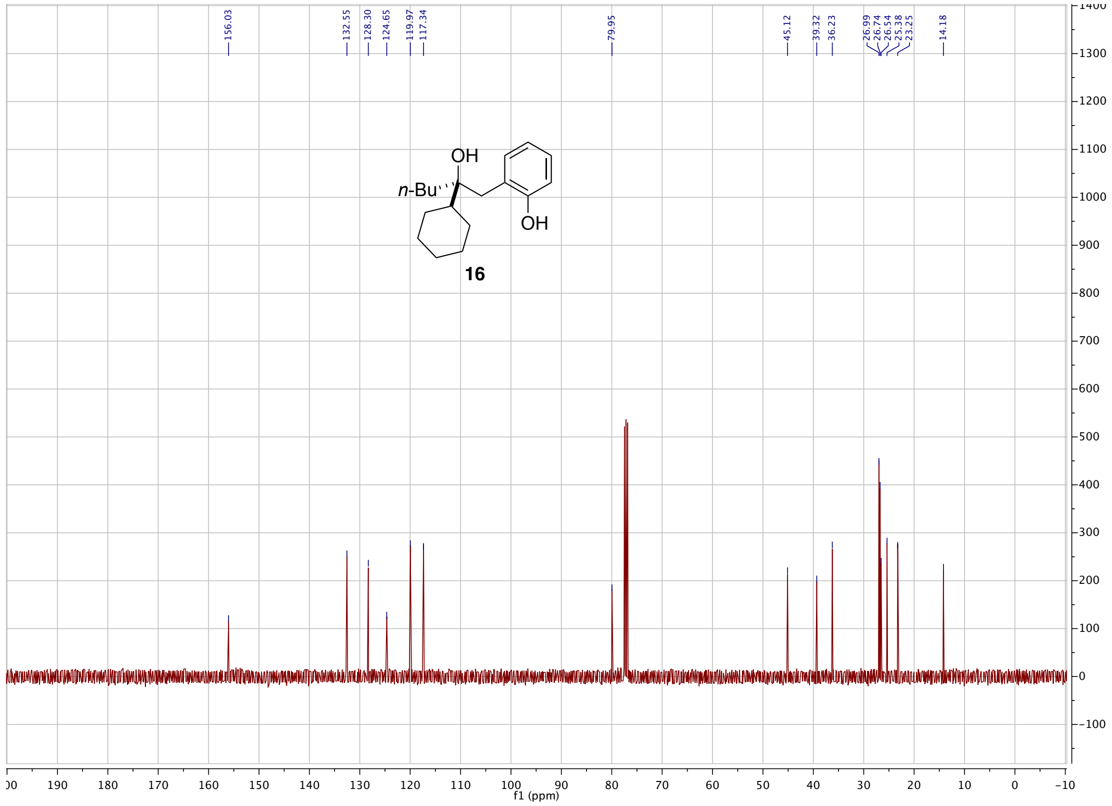

Figure S15. ^{1}H and ^{13}C NMR Spectra of **16**

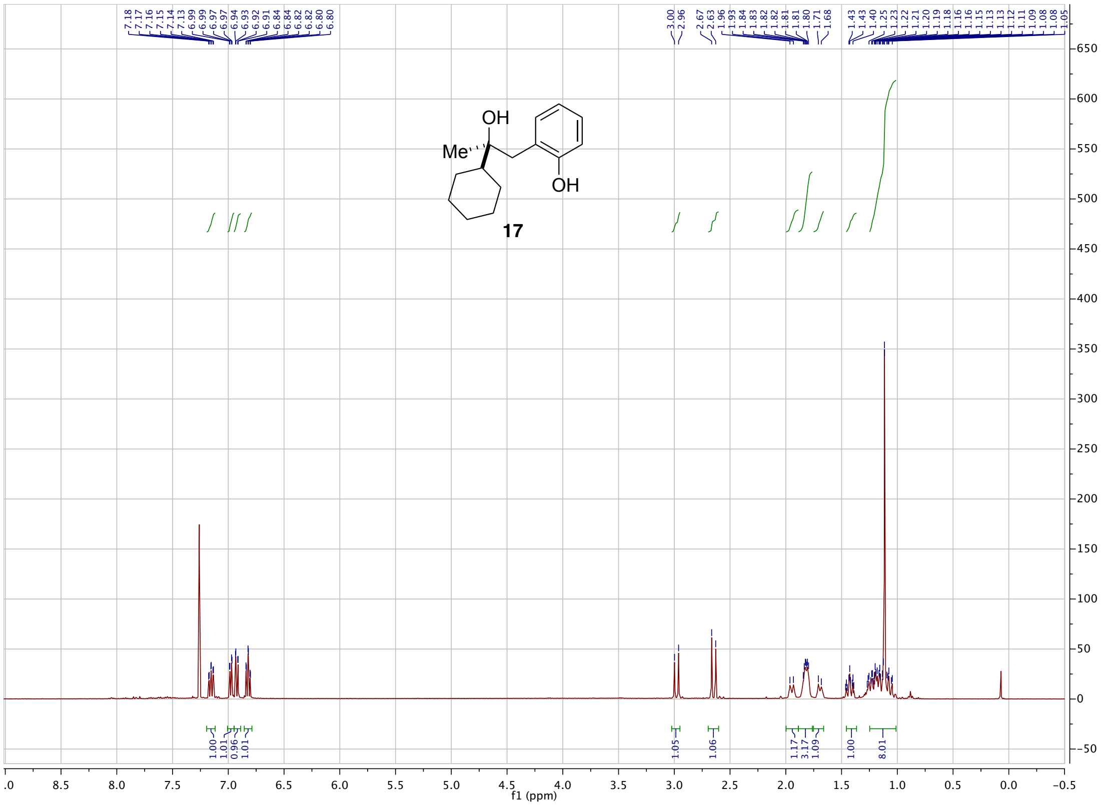

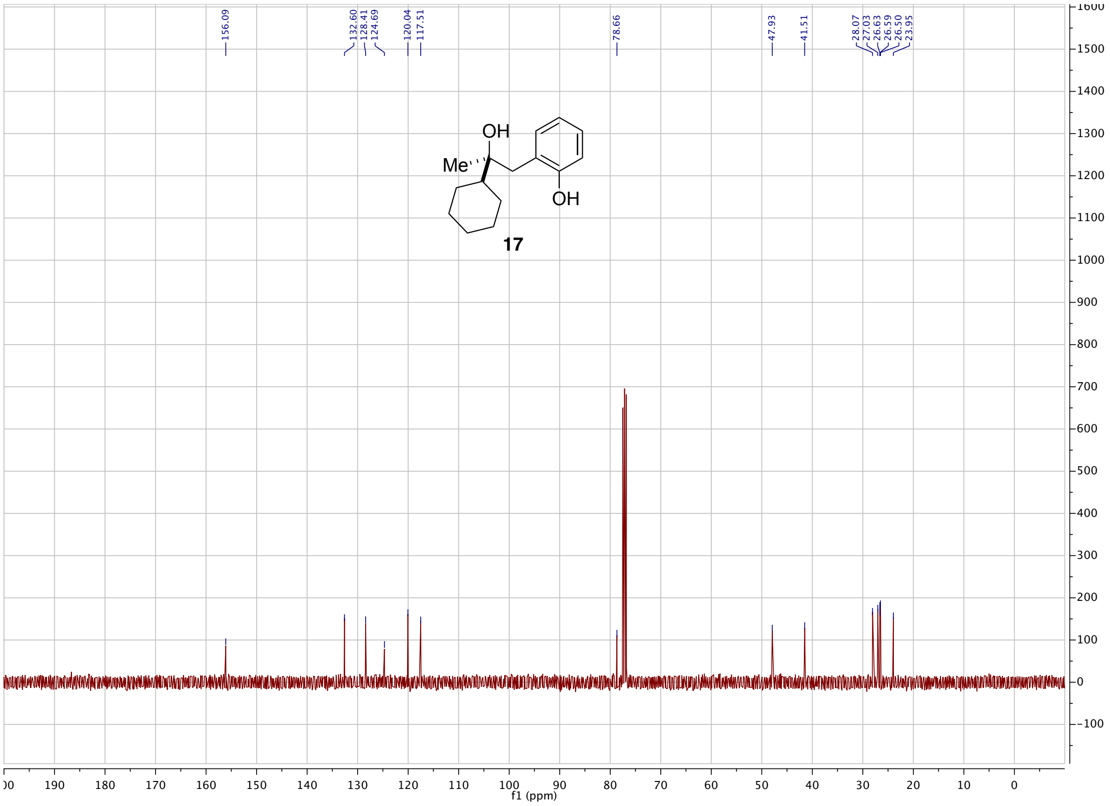

Figure S16. ^{1}H and ^{13}C NMR Spectra of **17**

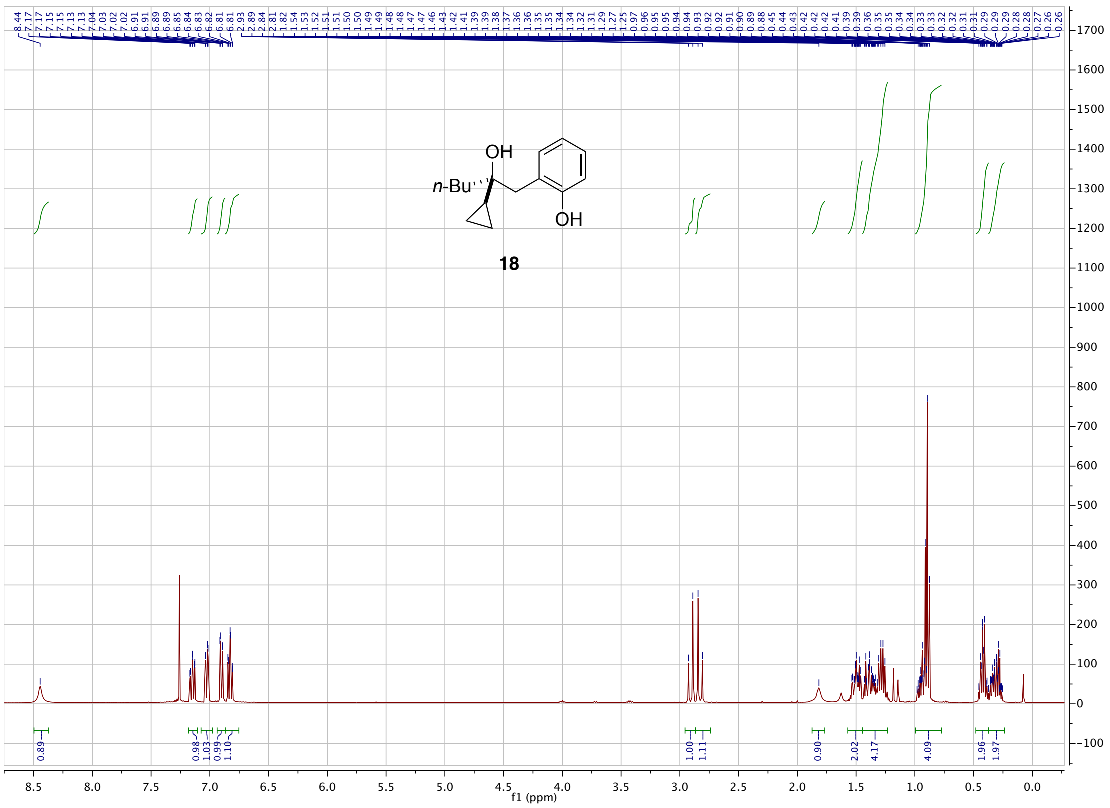

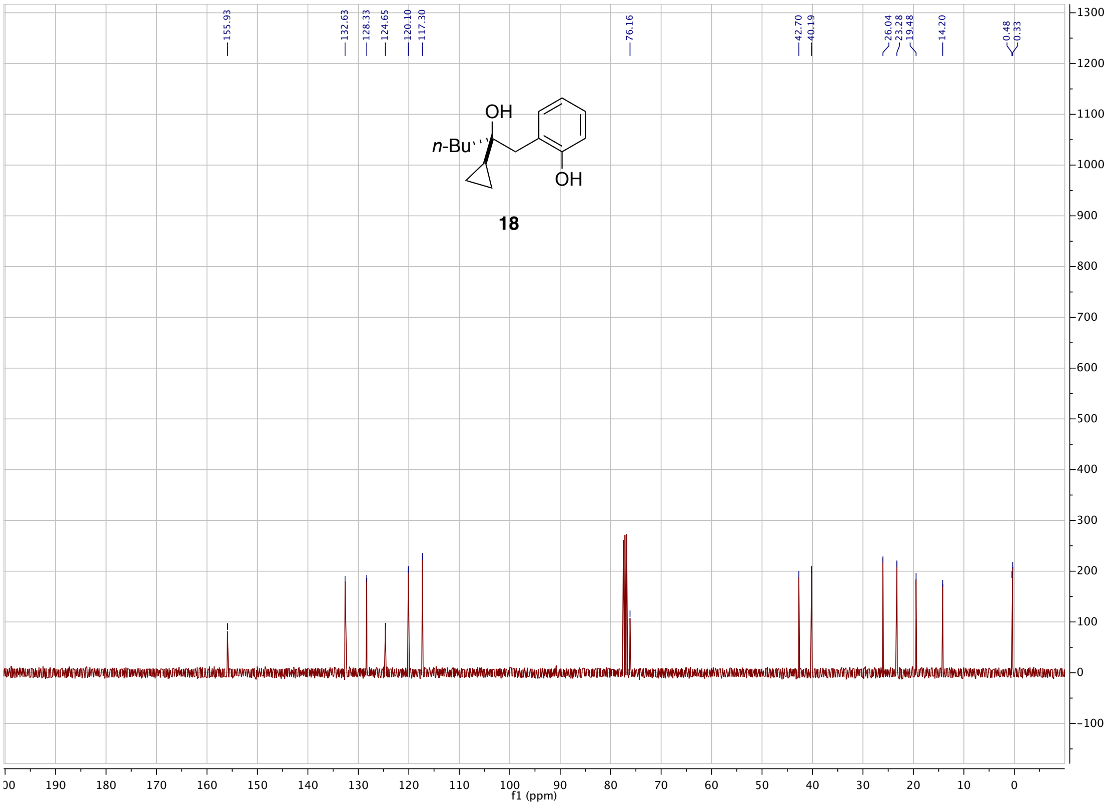

Figure S17. ^{1}H and ^{13}C NMR Spectra of **18**

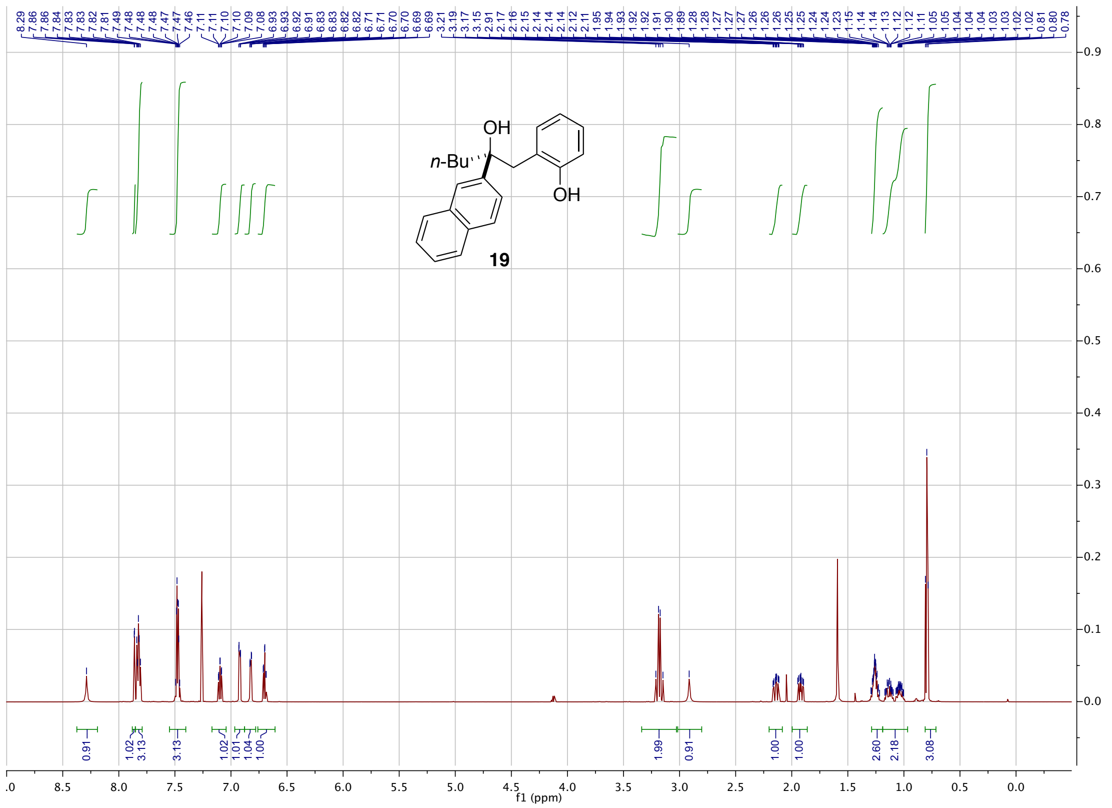

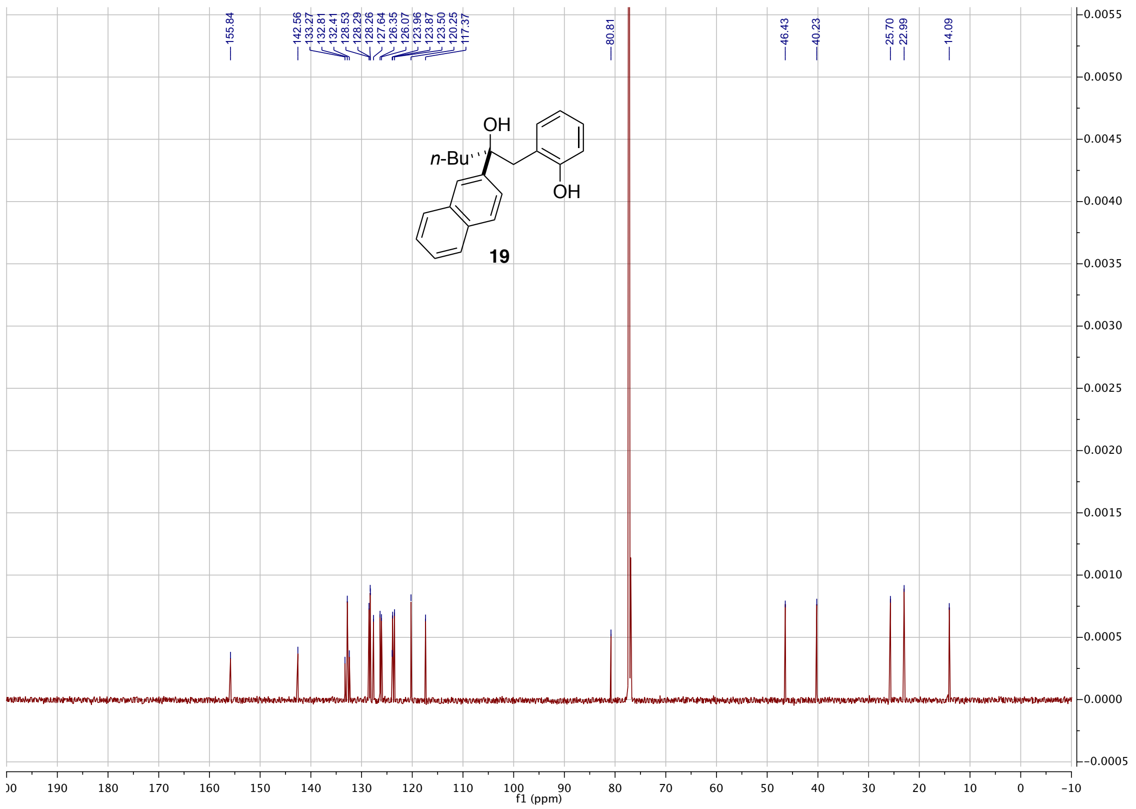

Figure S18. ^{1}H and ^{13}C NMR Spectra of **19**

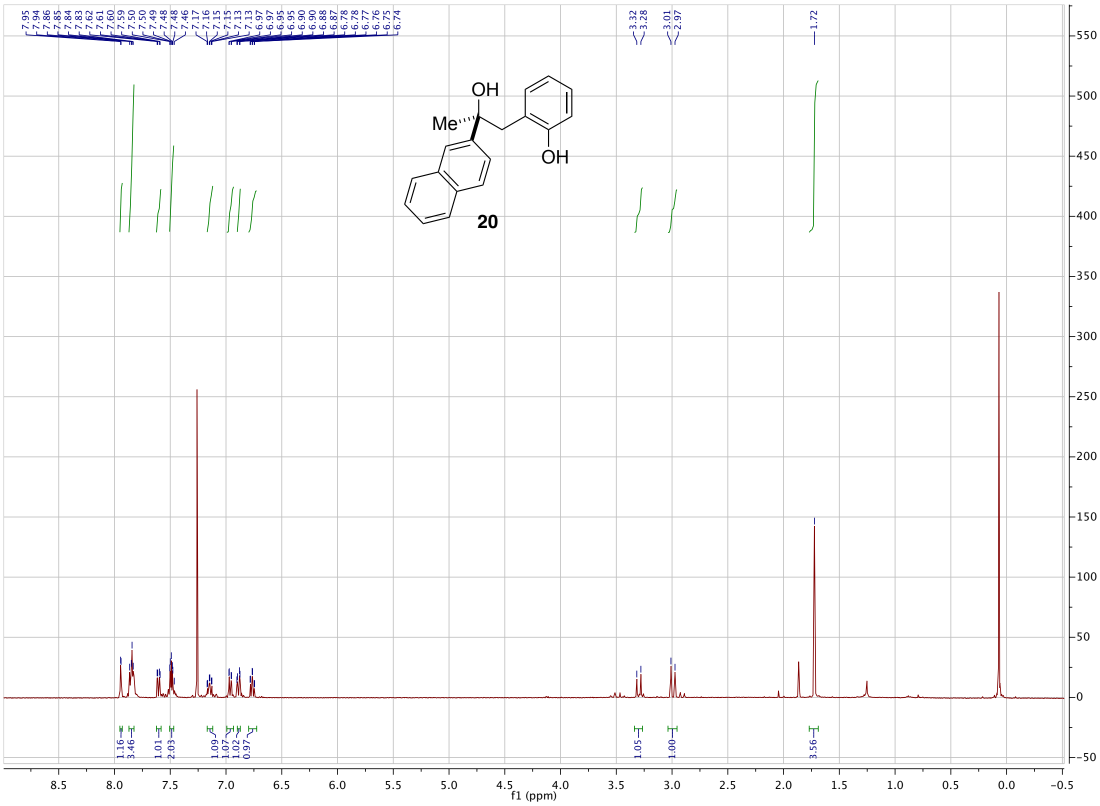

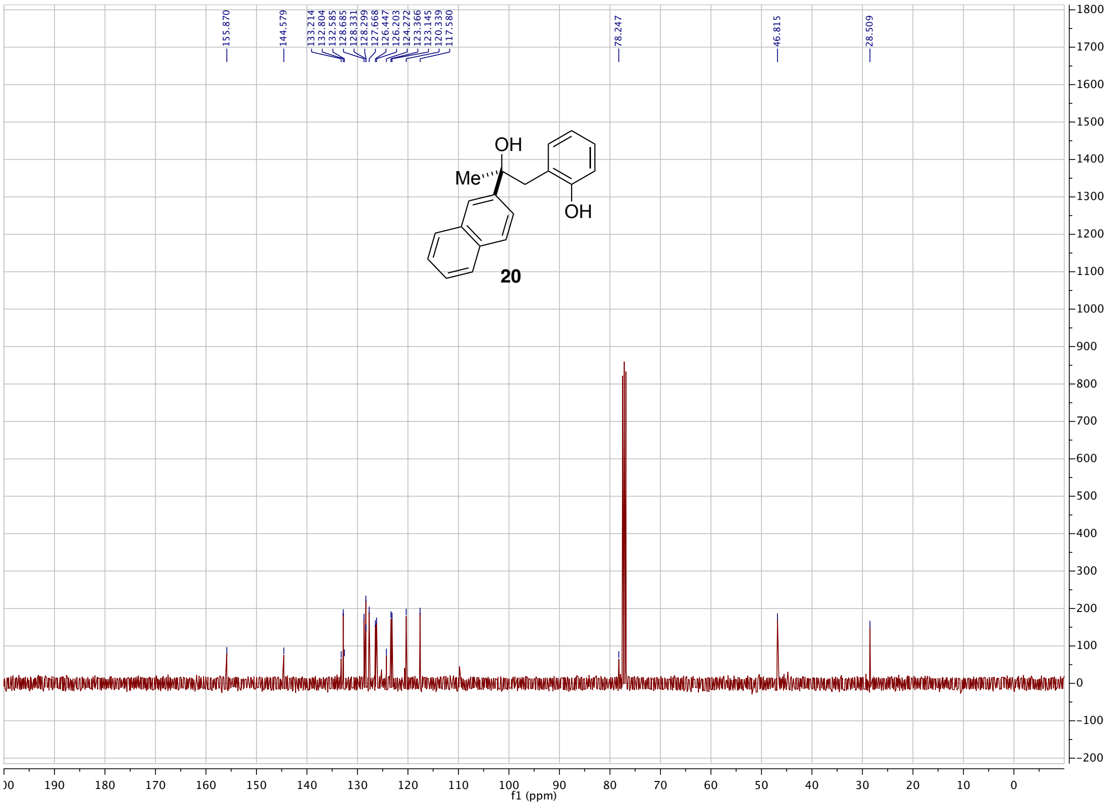

Figure S19. ^{1}H and ^{13}C NMR Spectra of **20**

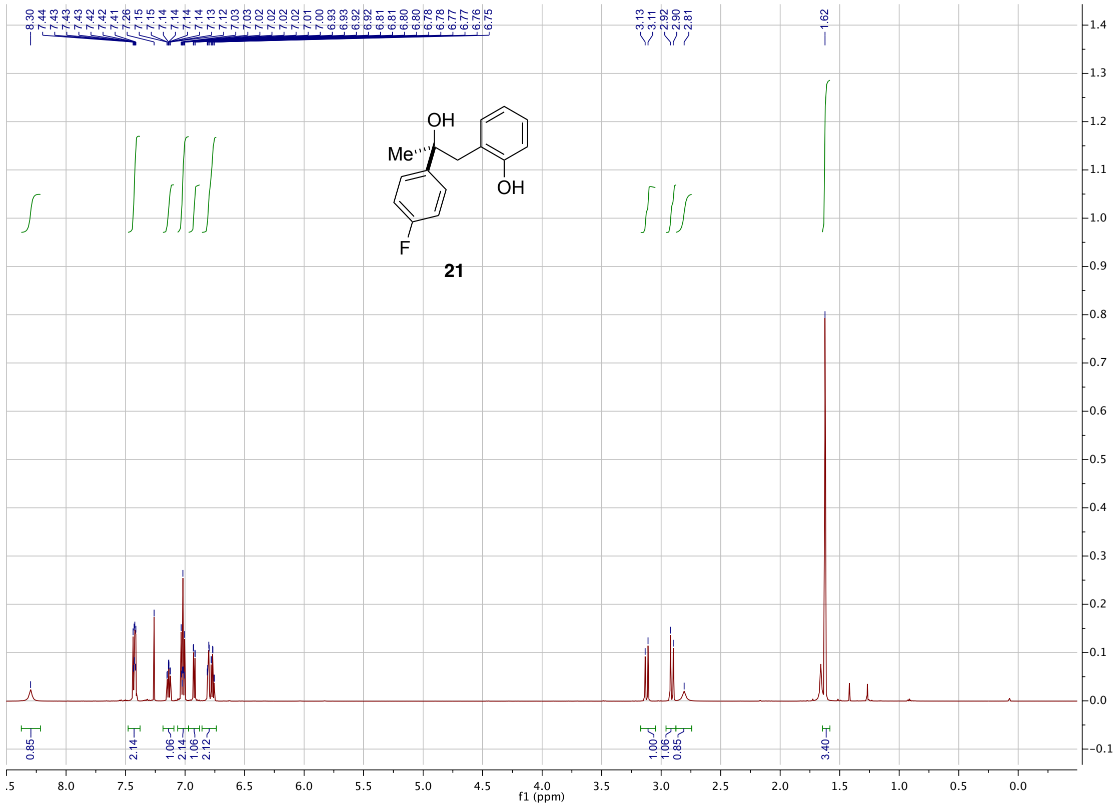

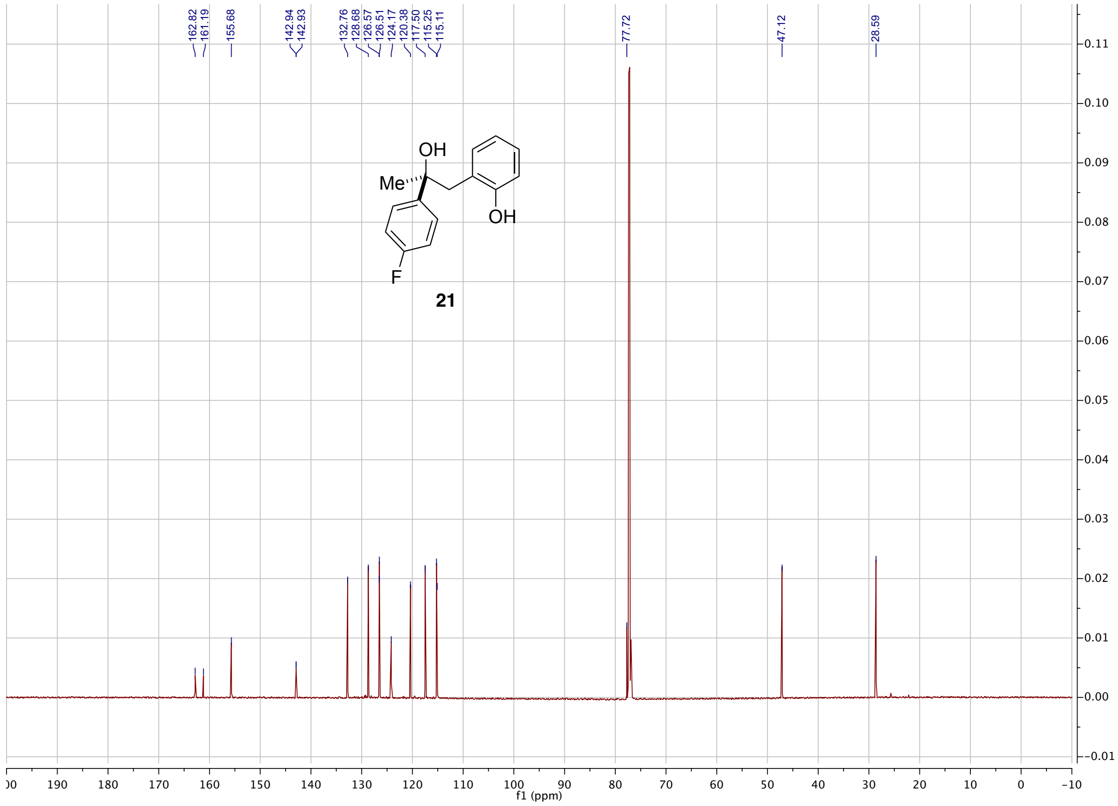

Figure S20. ^{1}H and ^{13}C NMR Spectra of **21**

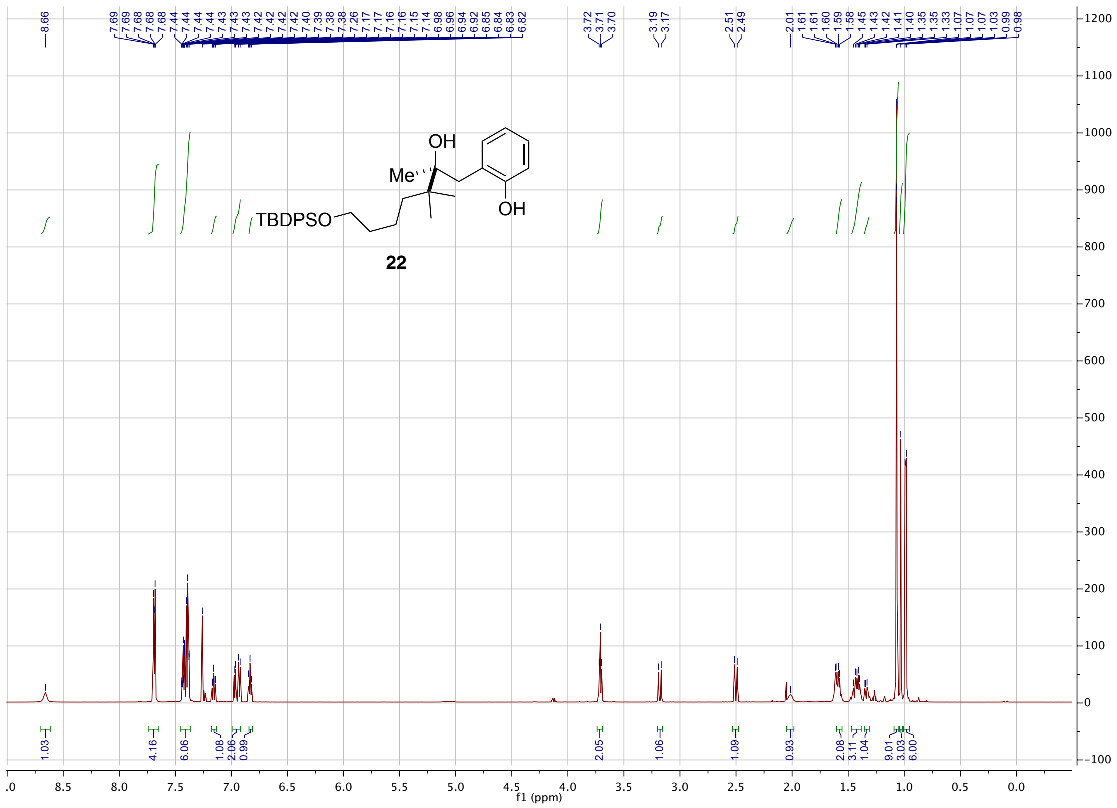

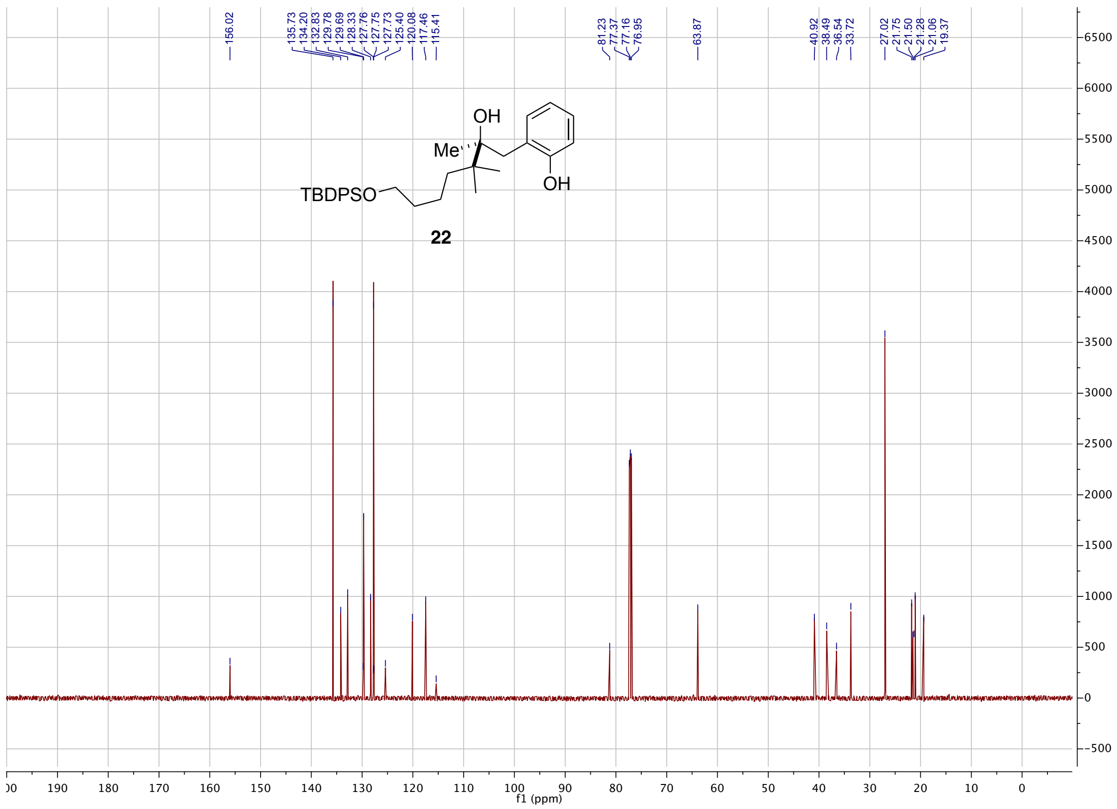

Figure S21. ^{1}H and ^{13}C NMR Spectra of **22**

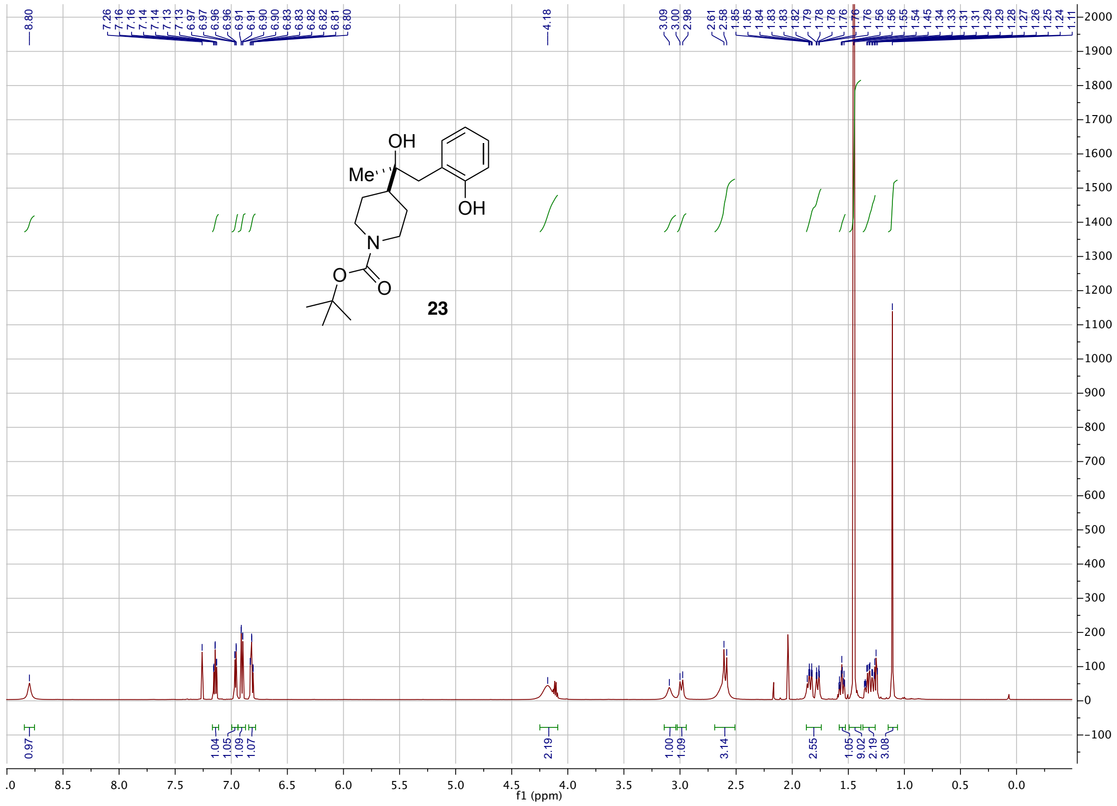

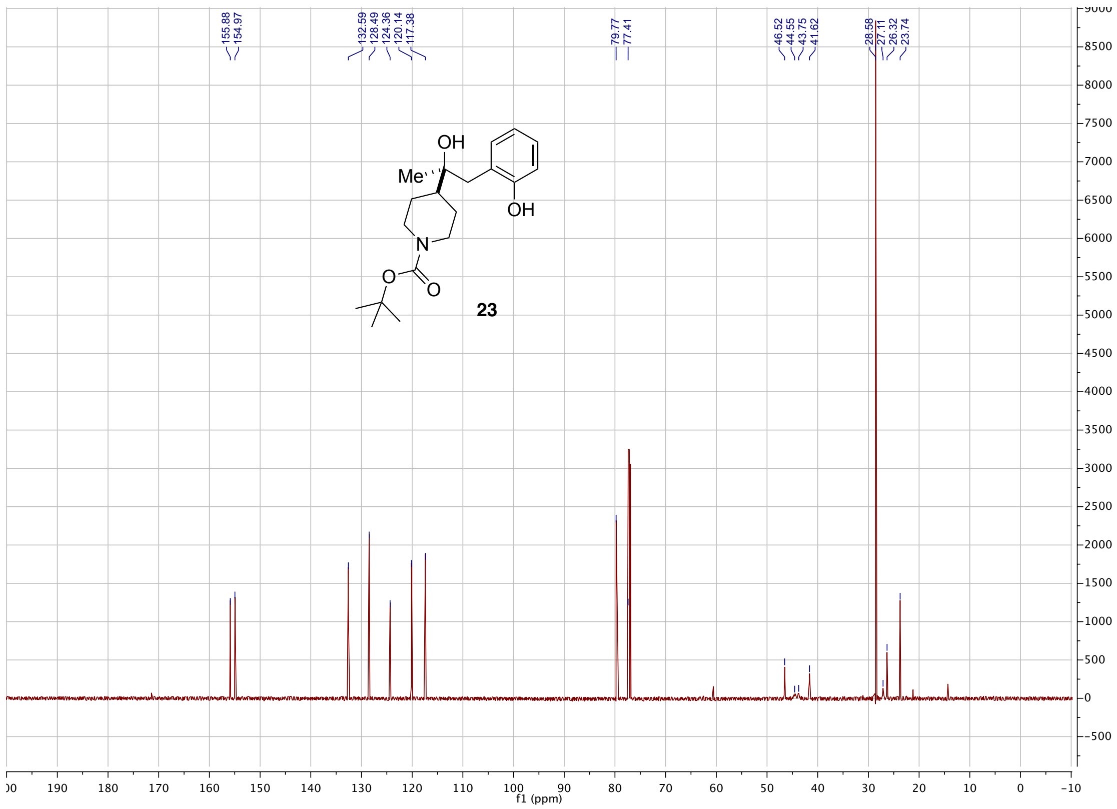

Figure S22. ^{1}H and ^{13}C NMR Spectra of **23**

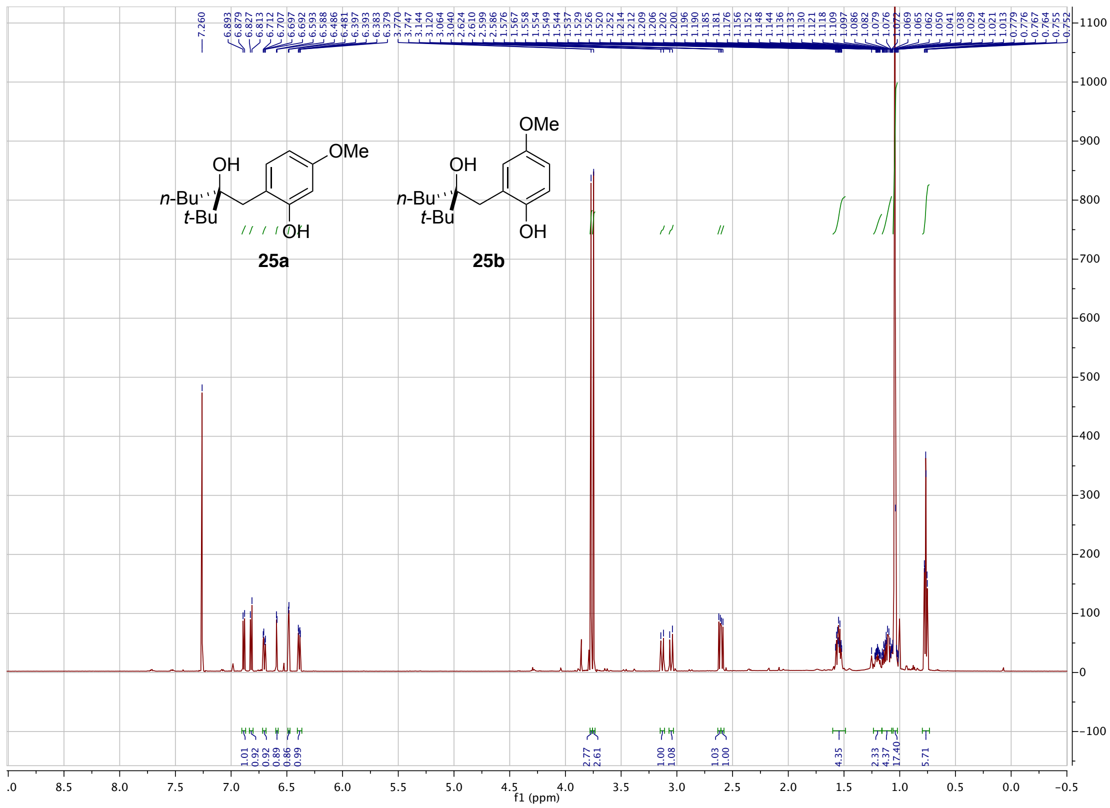

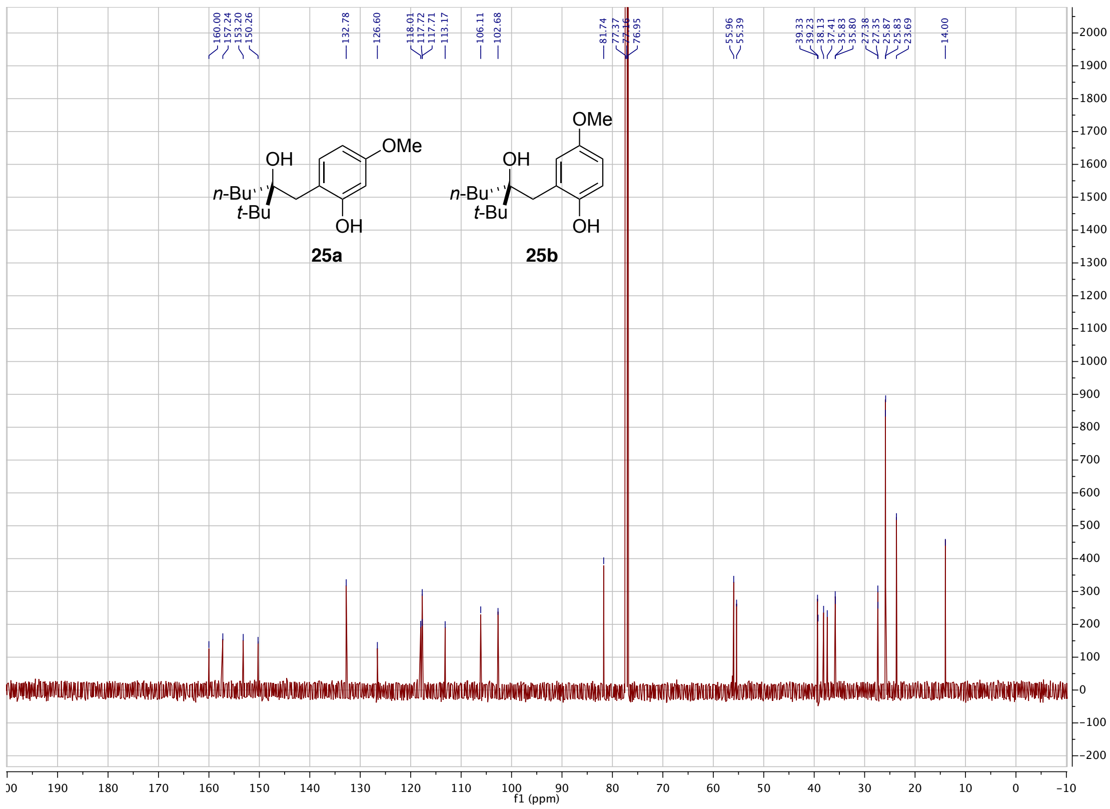

Figure S23. ^{1}H and ^{13}C NMR Spectra of **25**

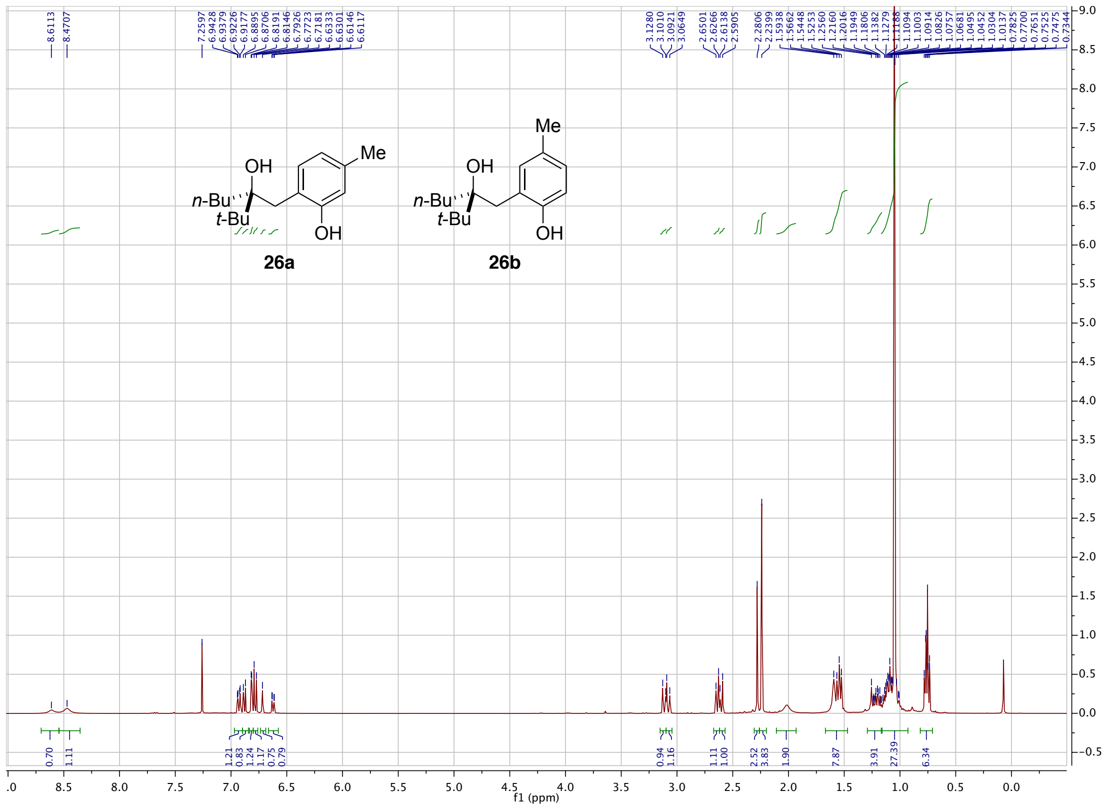

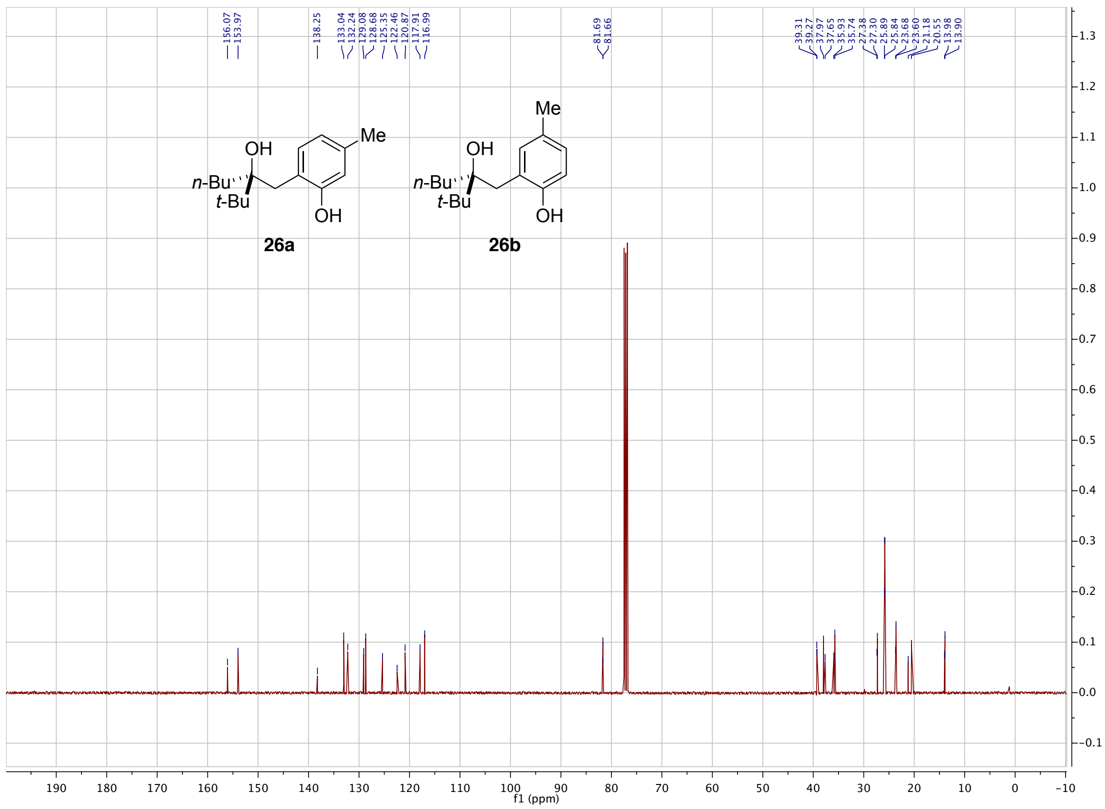

Figure S24. ^{1}H and ^{13}C NMR Spectra of **26**

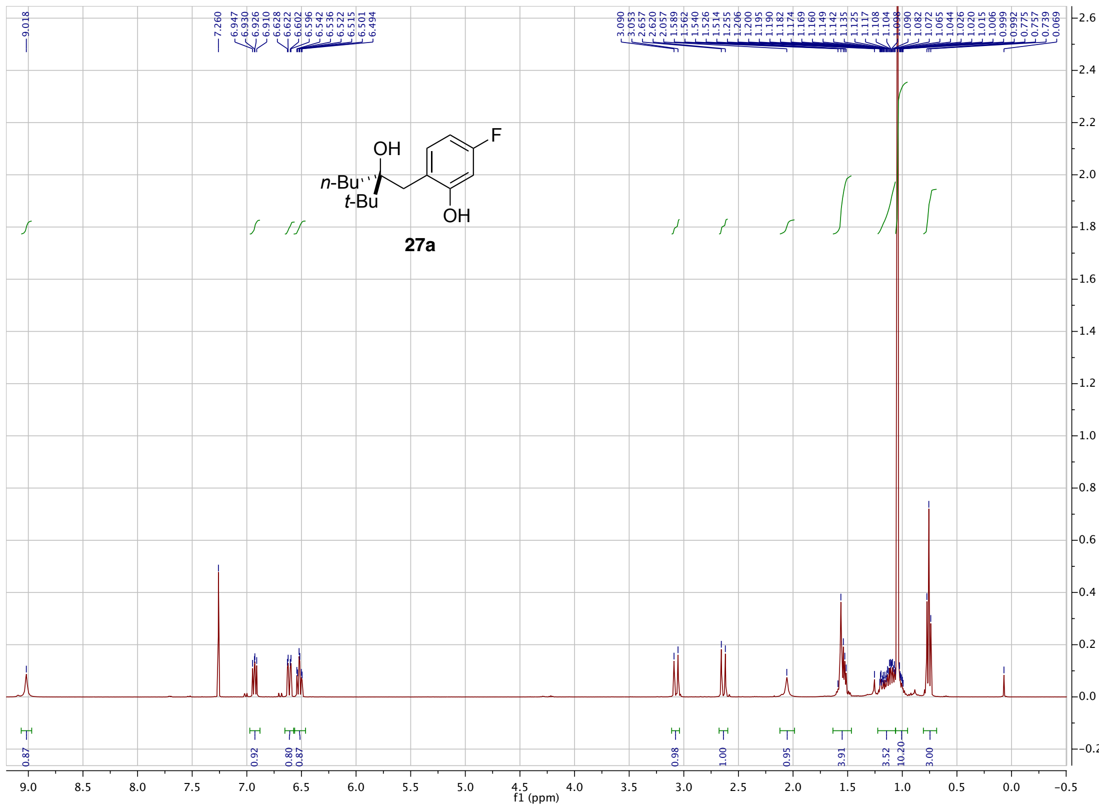

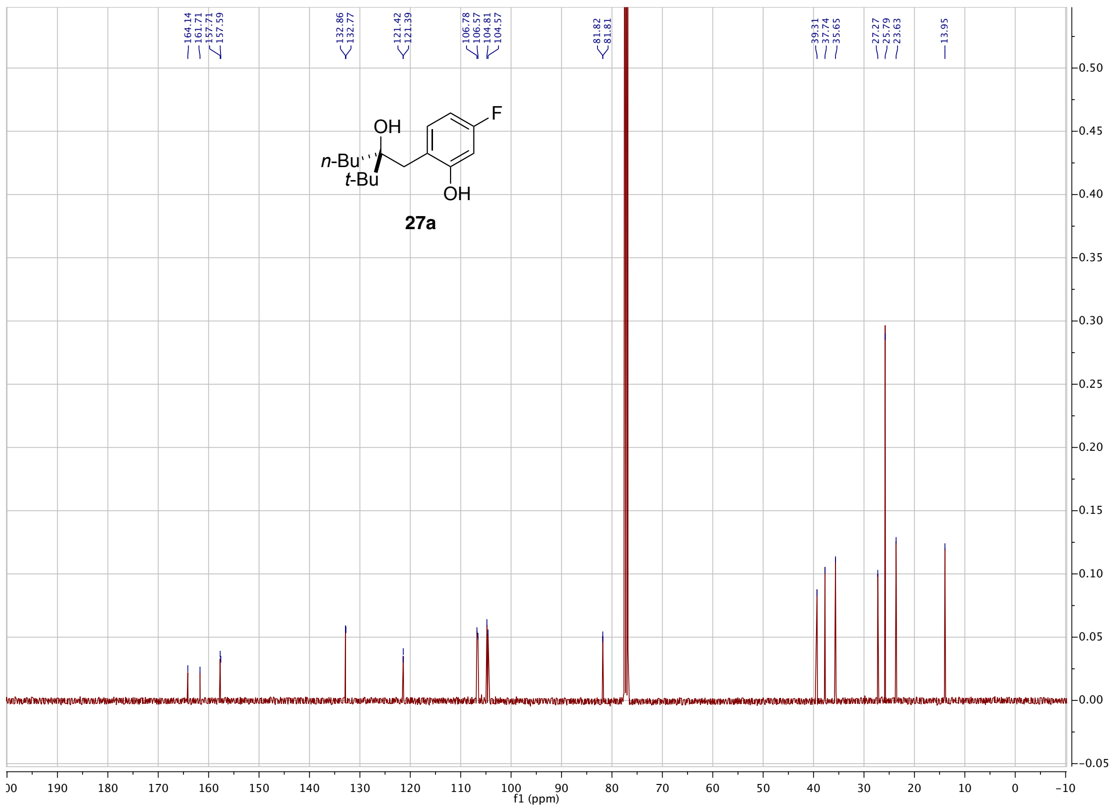

Figure S25. ^{1}H and ^{13}C NMR Spectra of **27a**

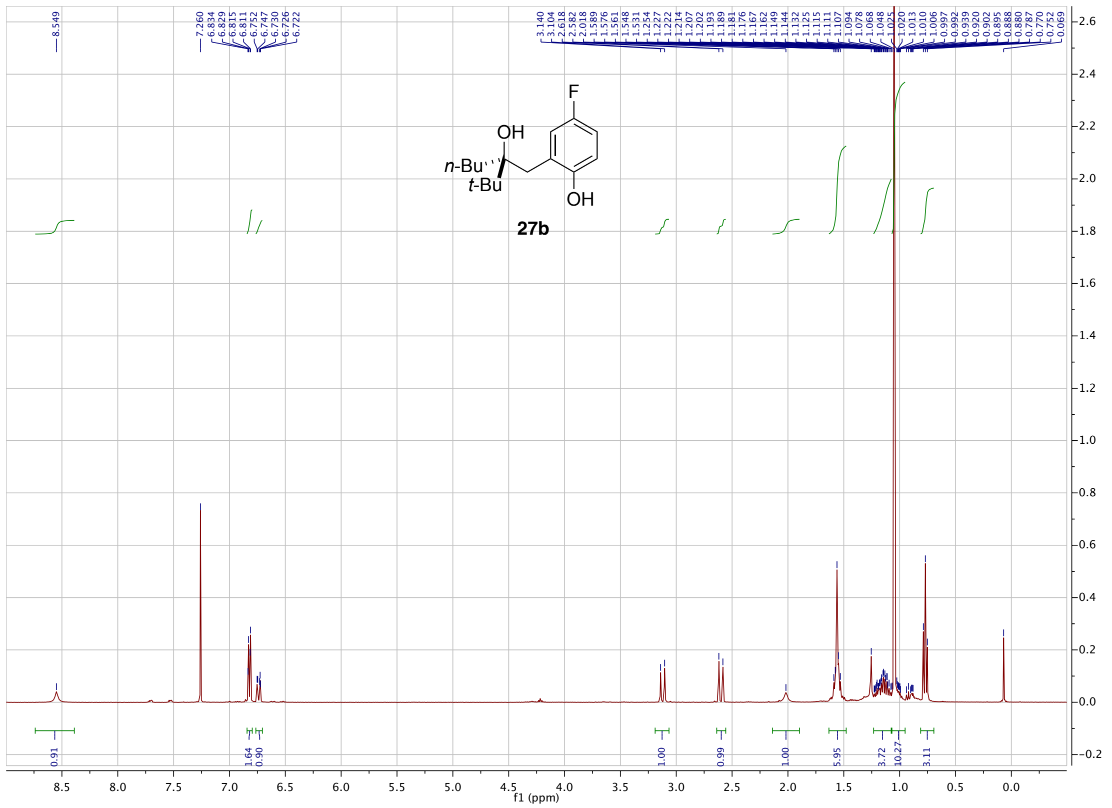

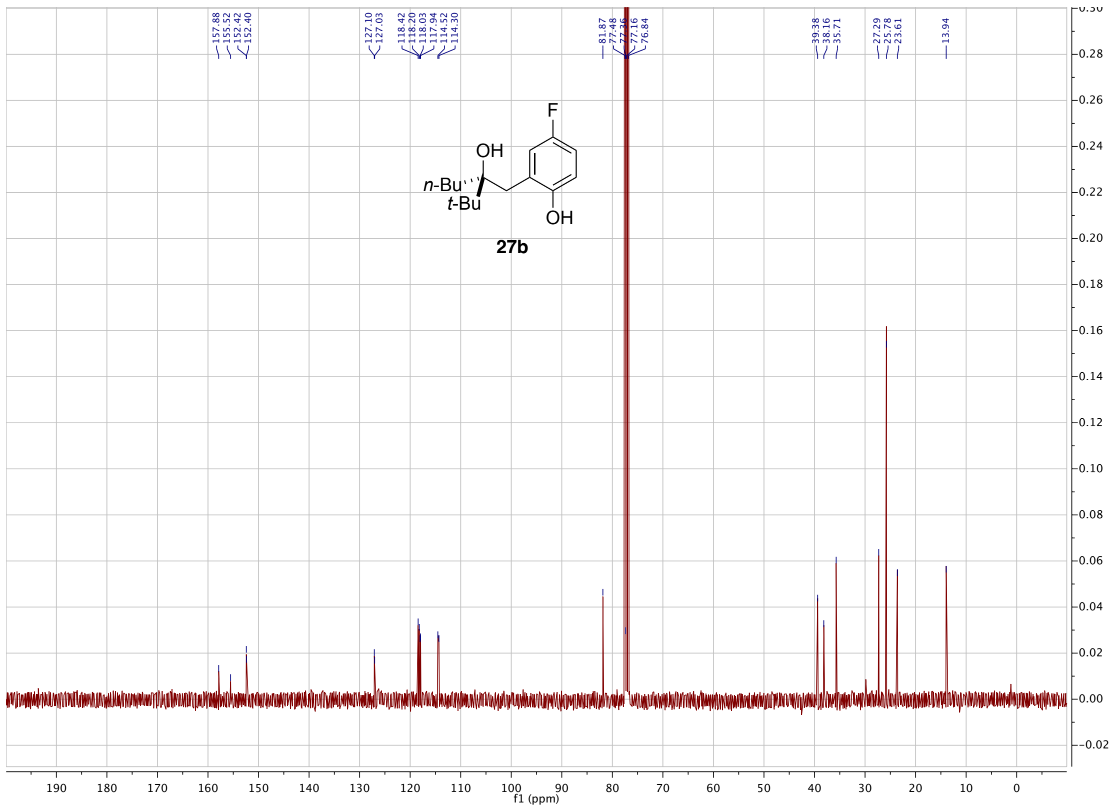

Figure S26. ^{1}H and ^{13}C NMR Spectra of **27b**

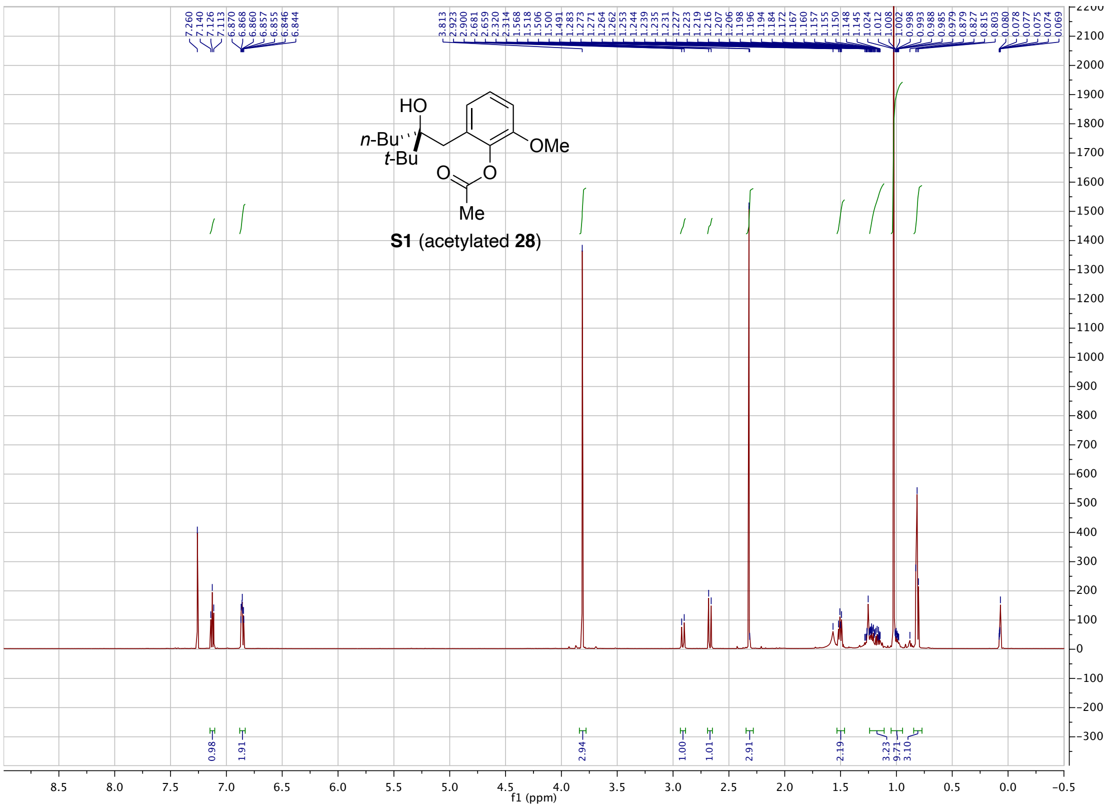

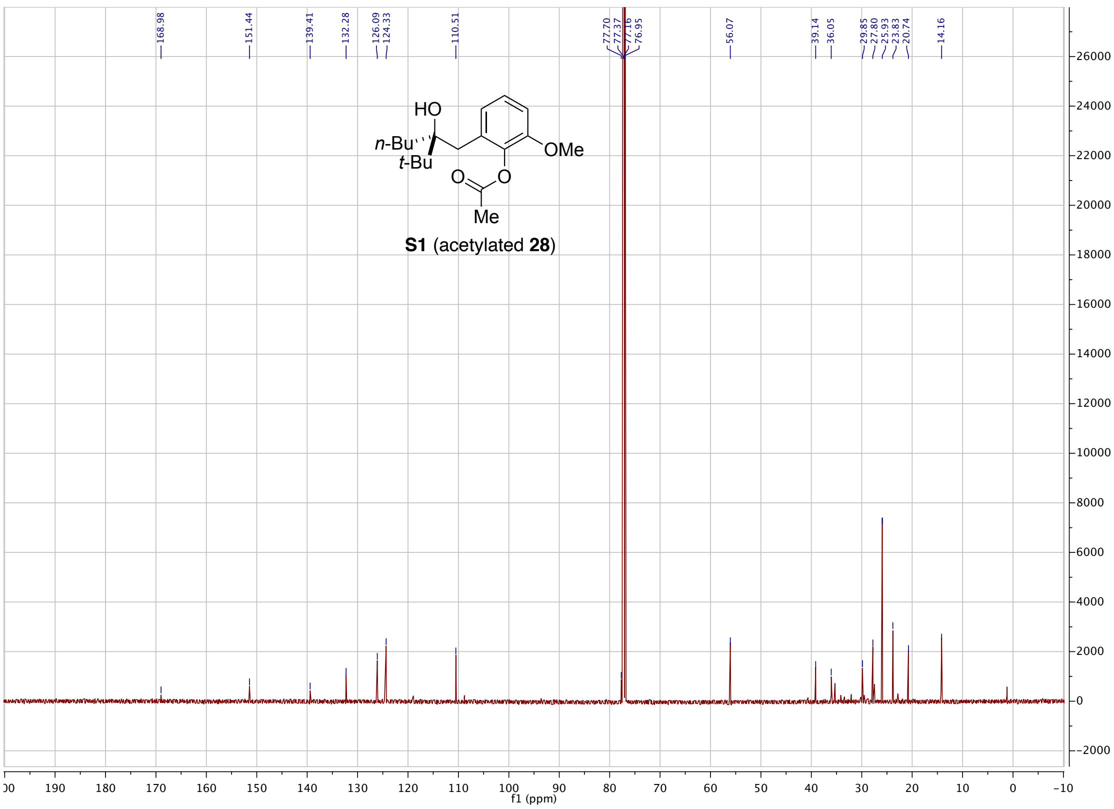

Figure S27. ^{1}H and ^{13}C NMR Spectra of **S1** (acetylated **28**)

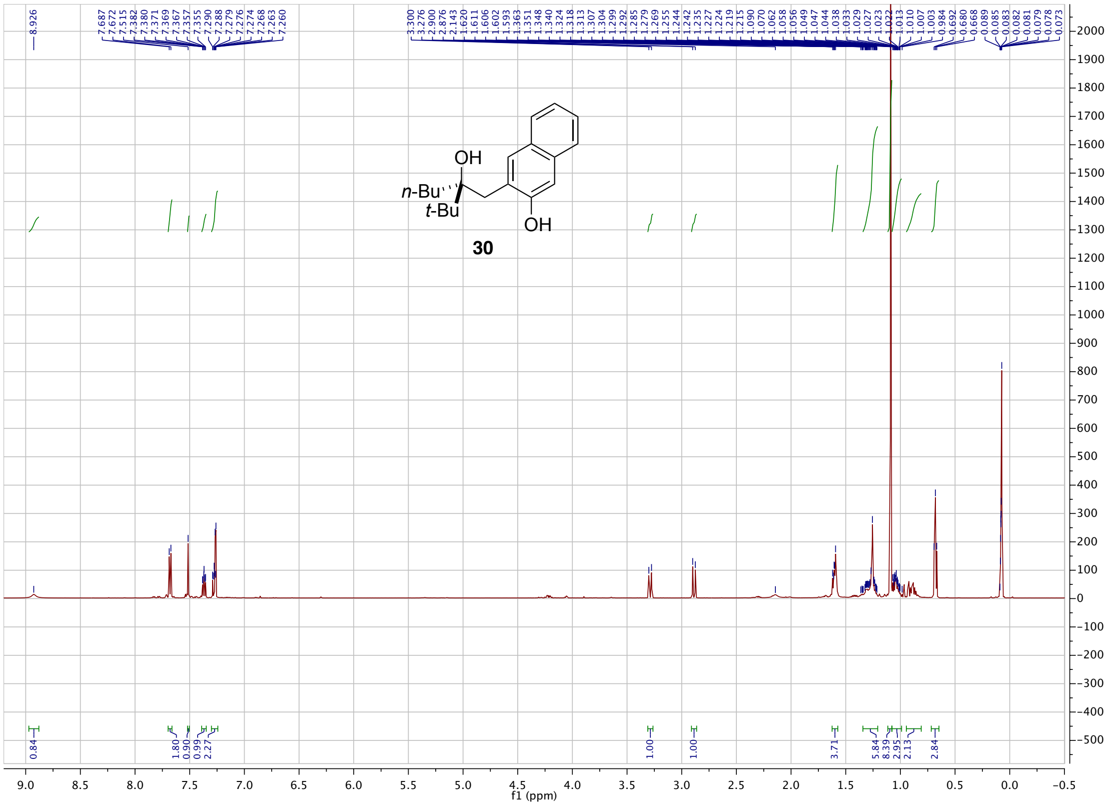

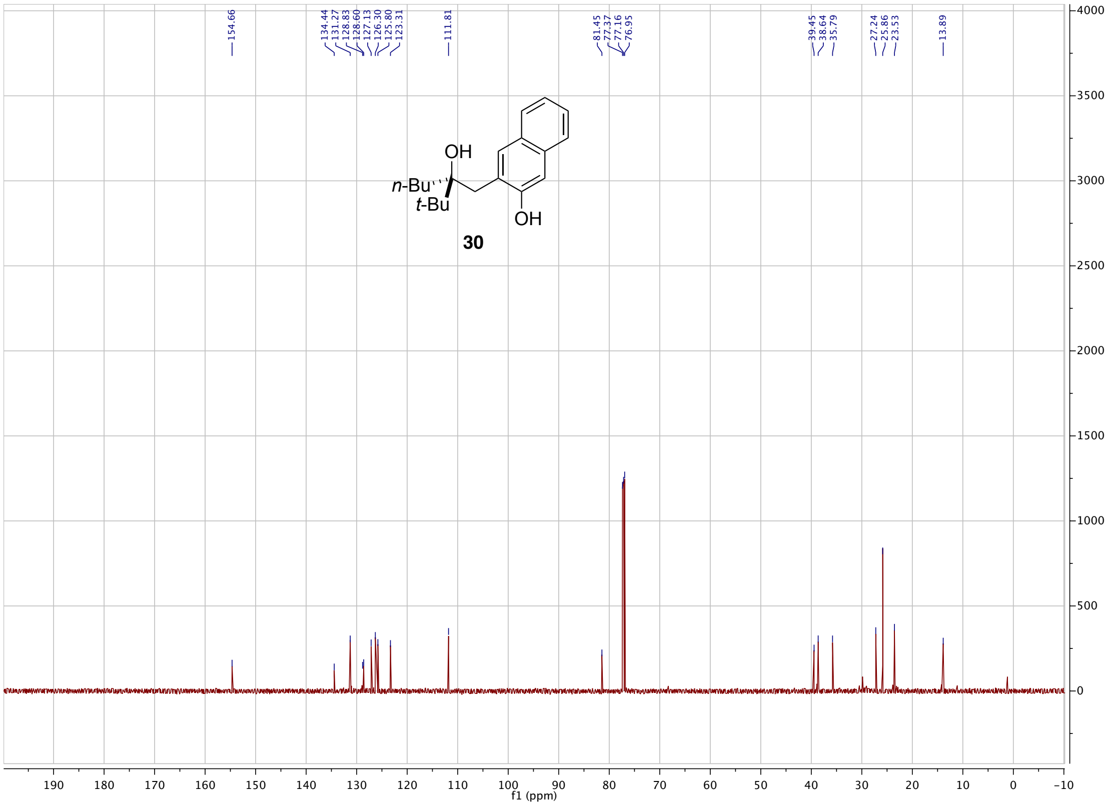

Figure S28. ^{1}H and ^{13}C NMR Spectra of **30**

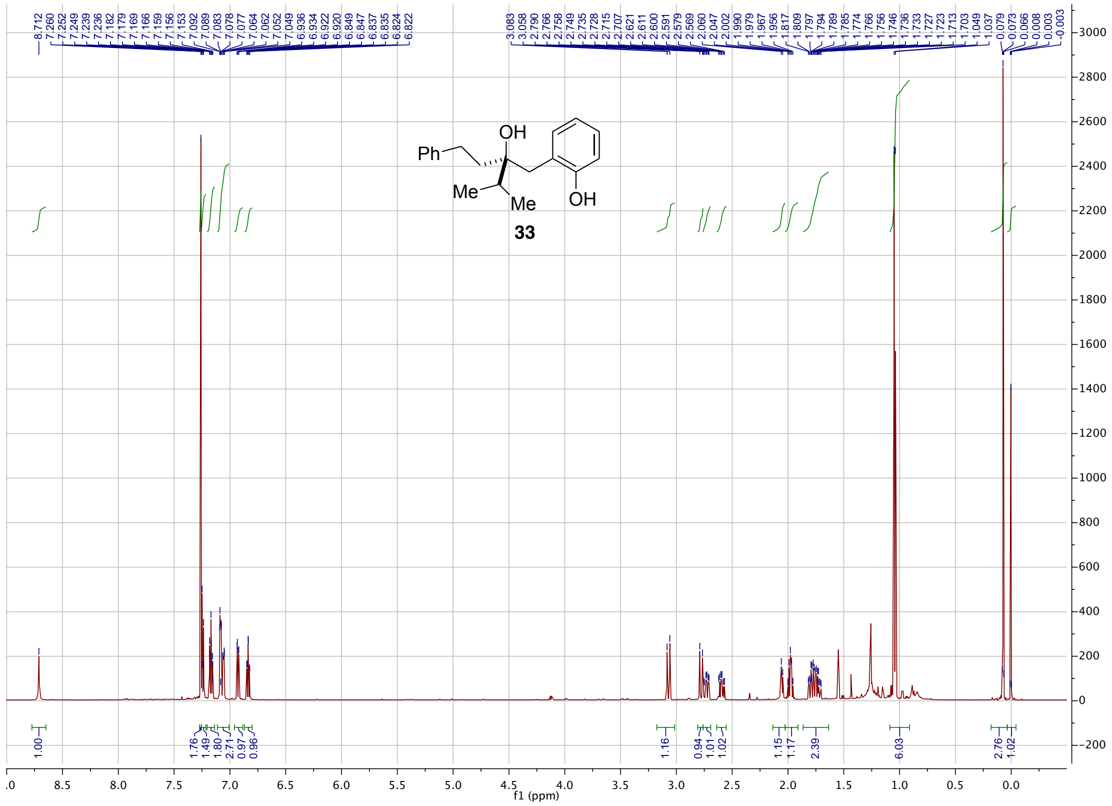

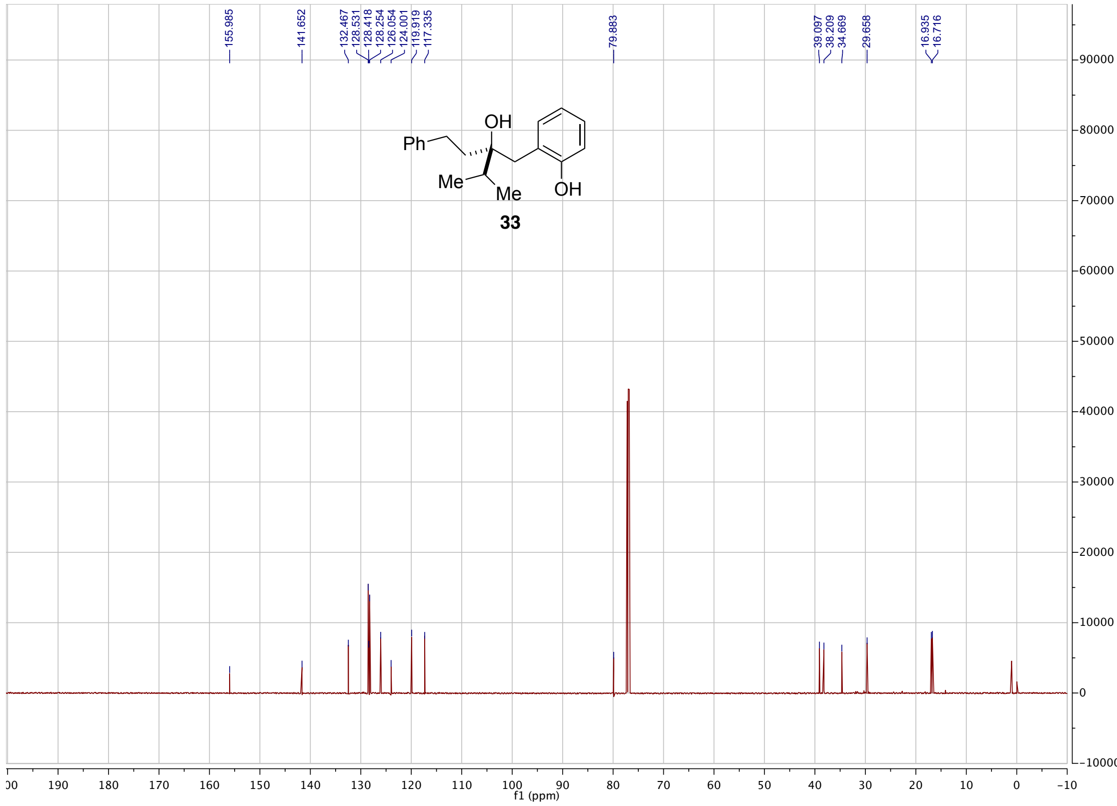

Figure S29. ^{1}H and ^{13}C NMR Spectra of **33**

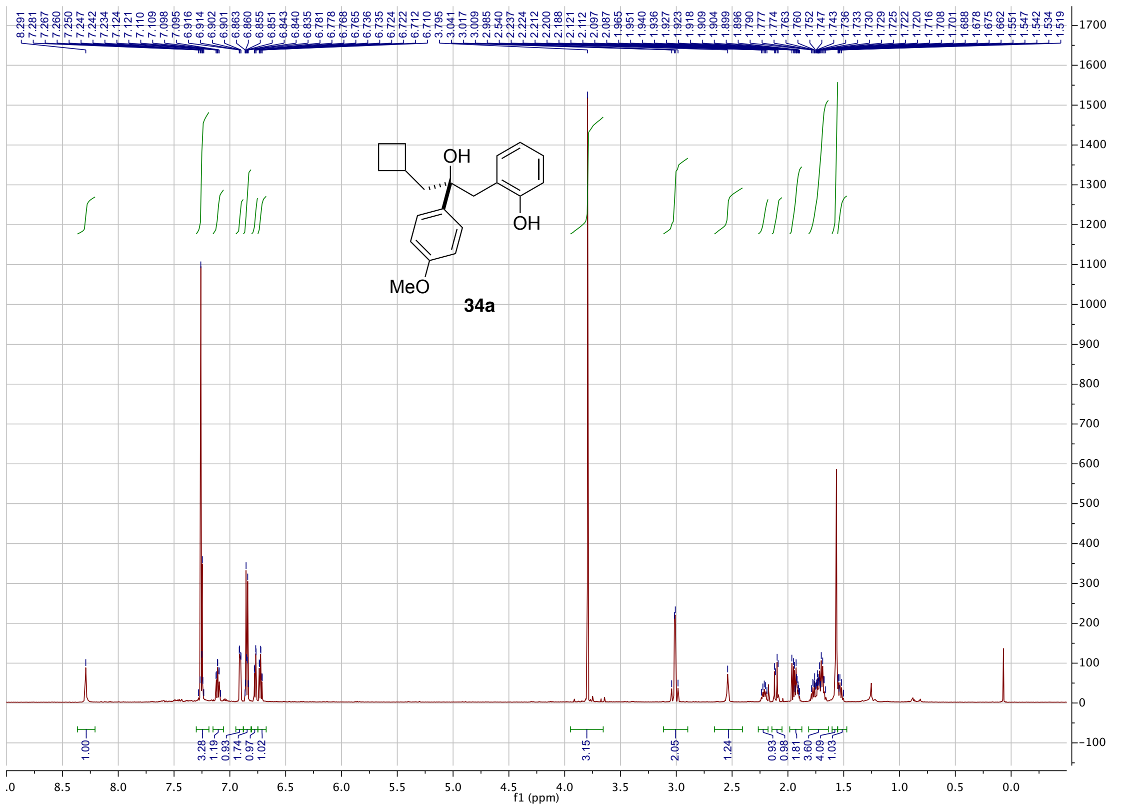

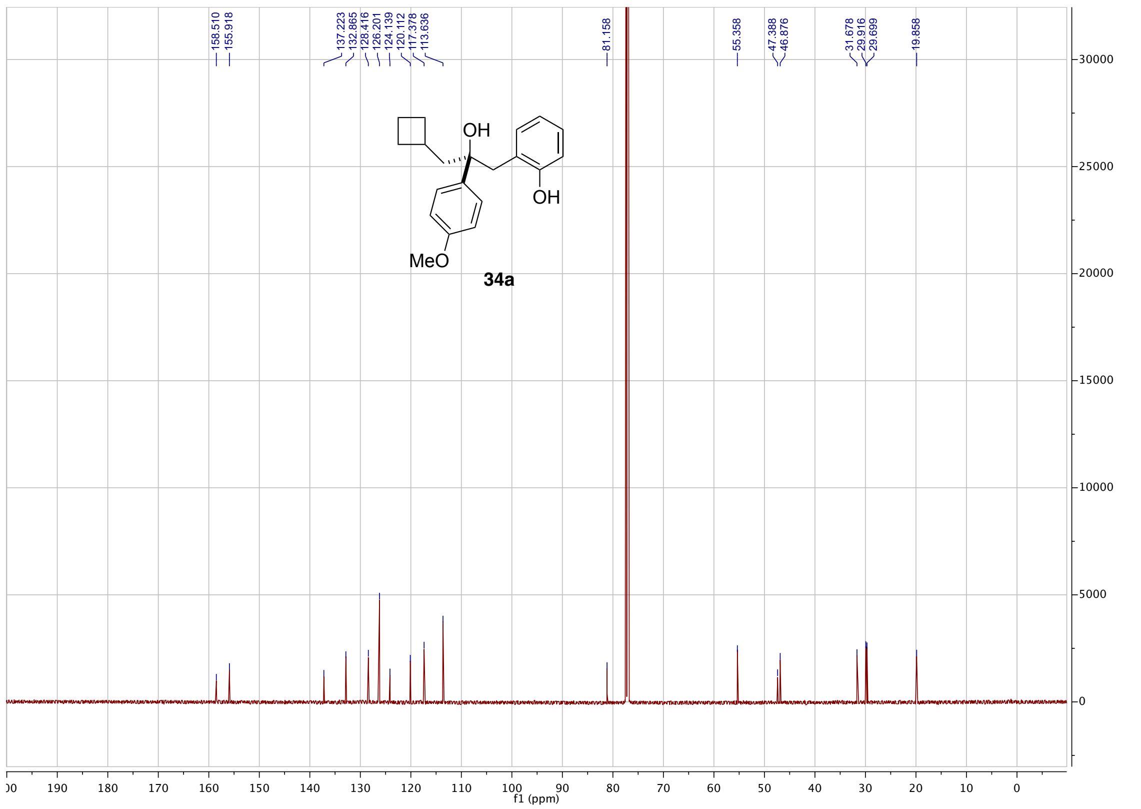

Figure S30. ^{1}H and ^{13}C NMR Spectra of **34a**

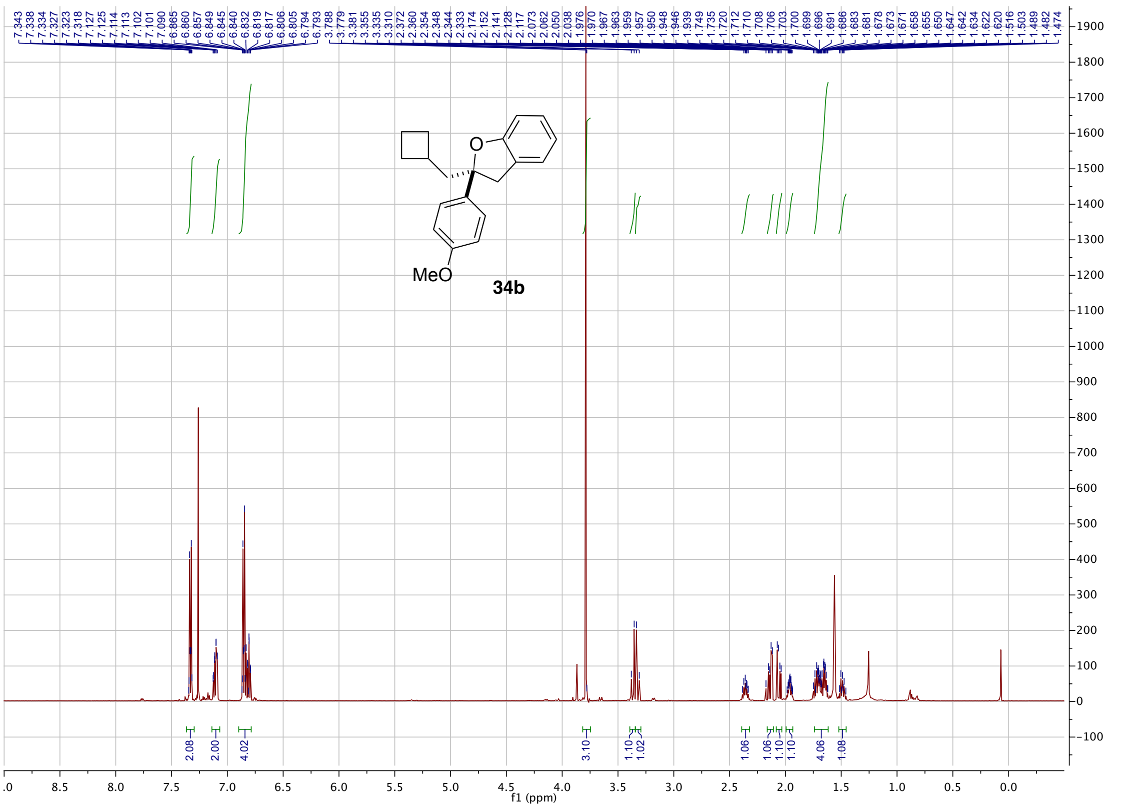

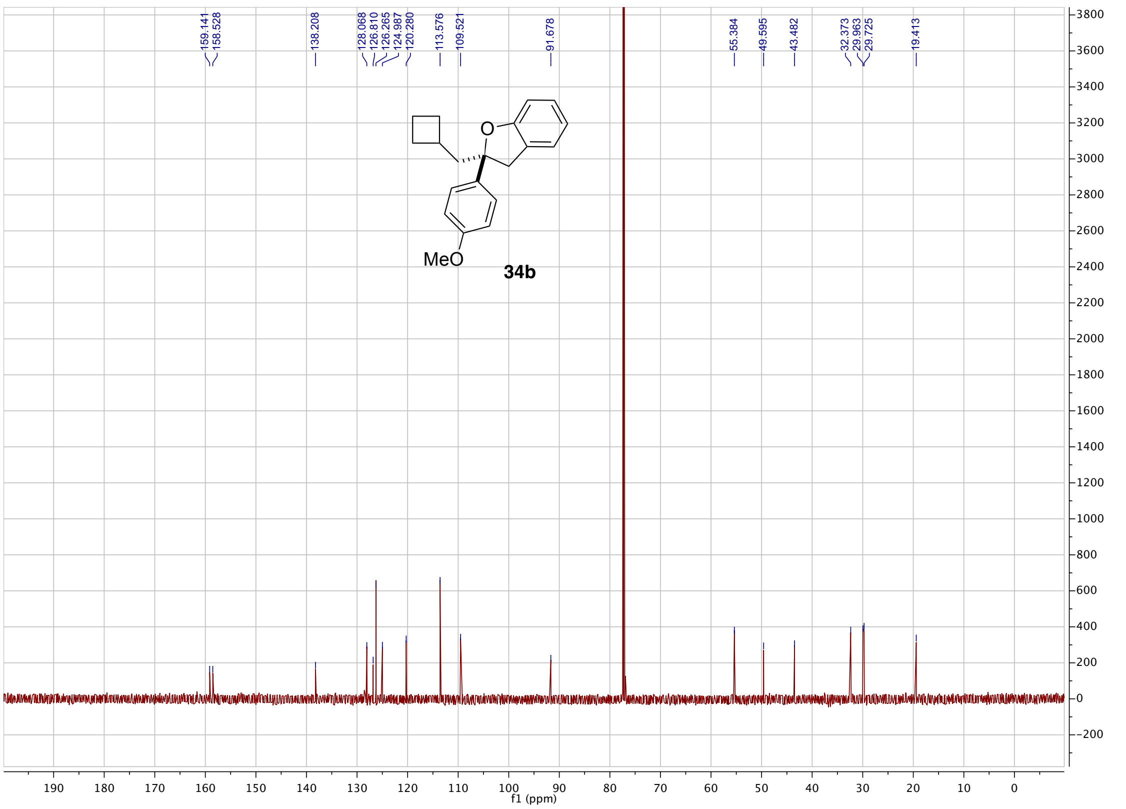

Figure S31. ^{1}H and ^{13}C NMR Spectra of **34b**

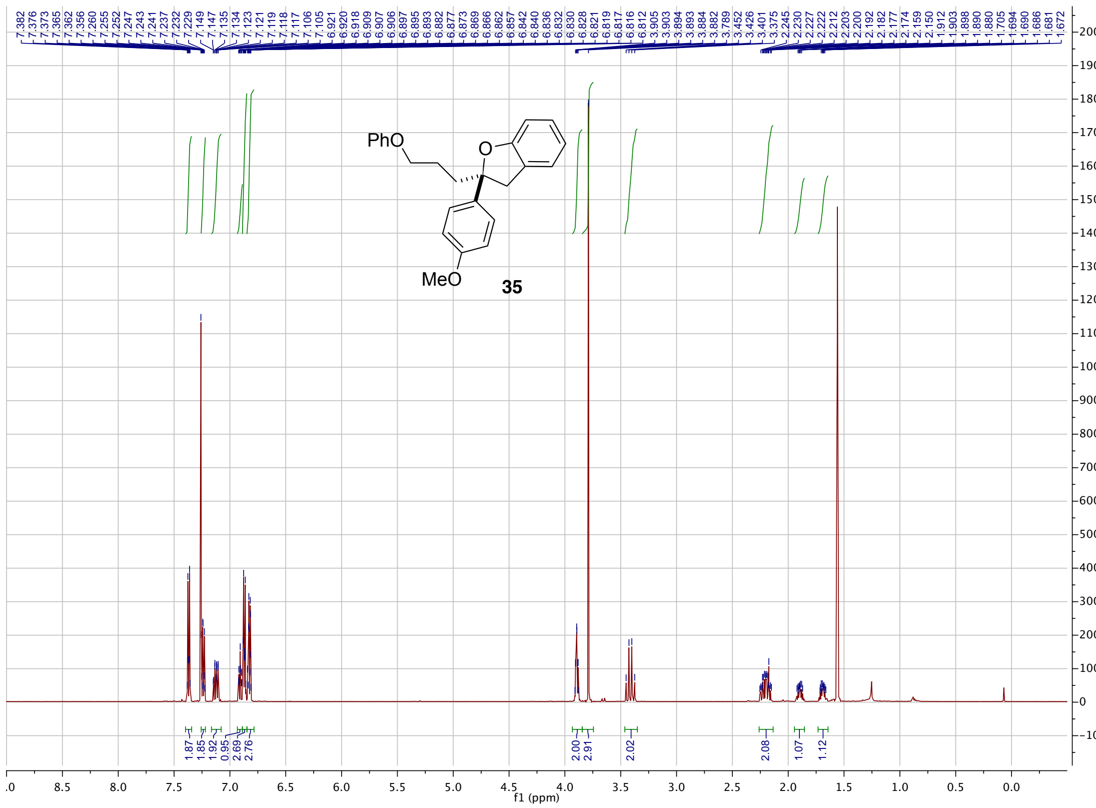

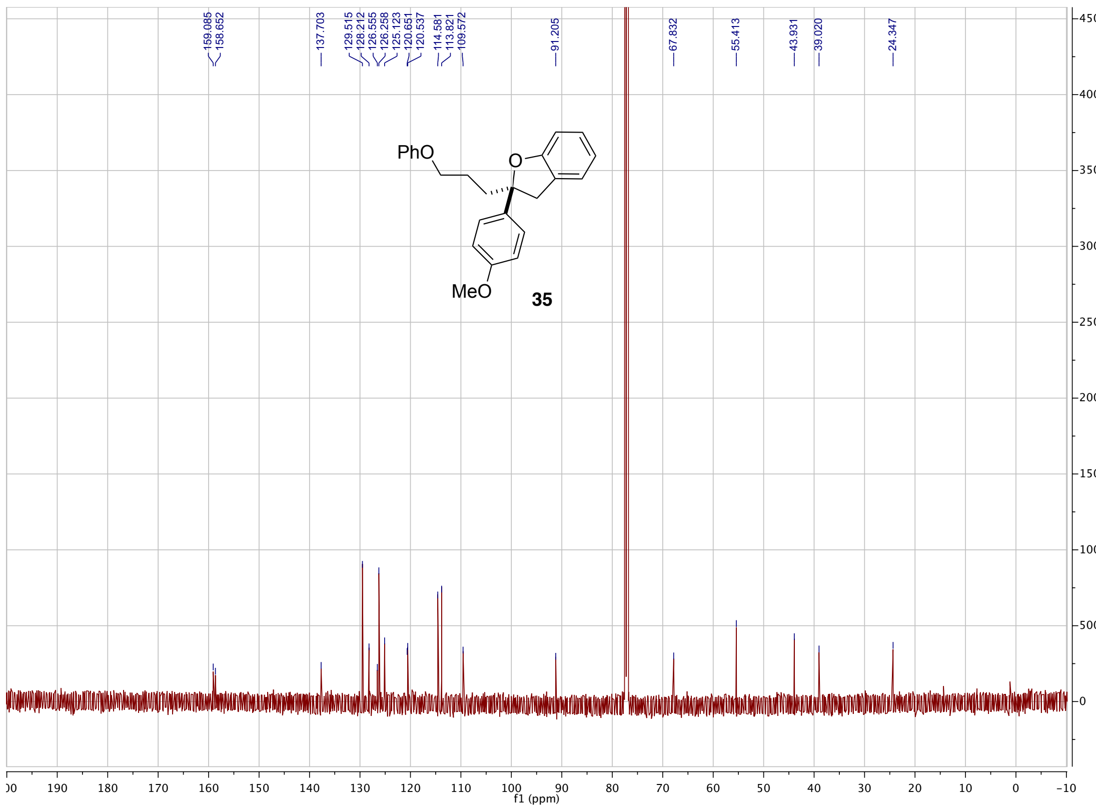

Figure S32. ^{1}H and ^{13}C NMR Spectra of **35**

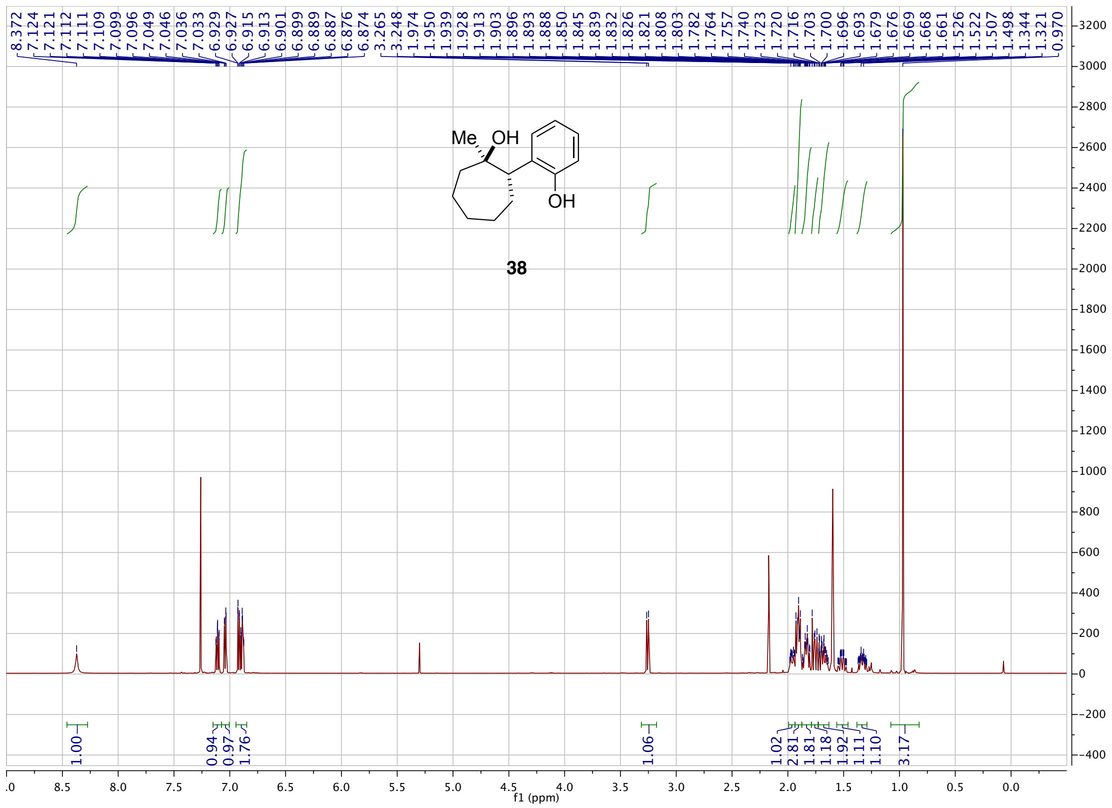

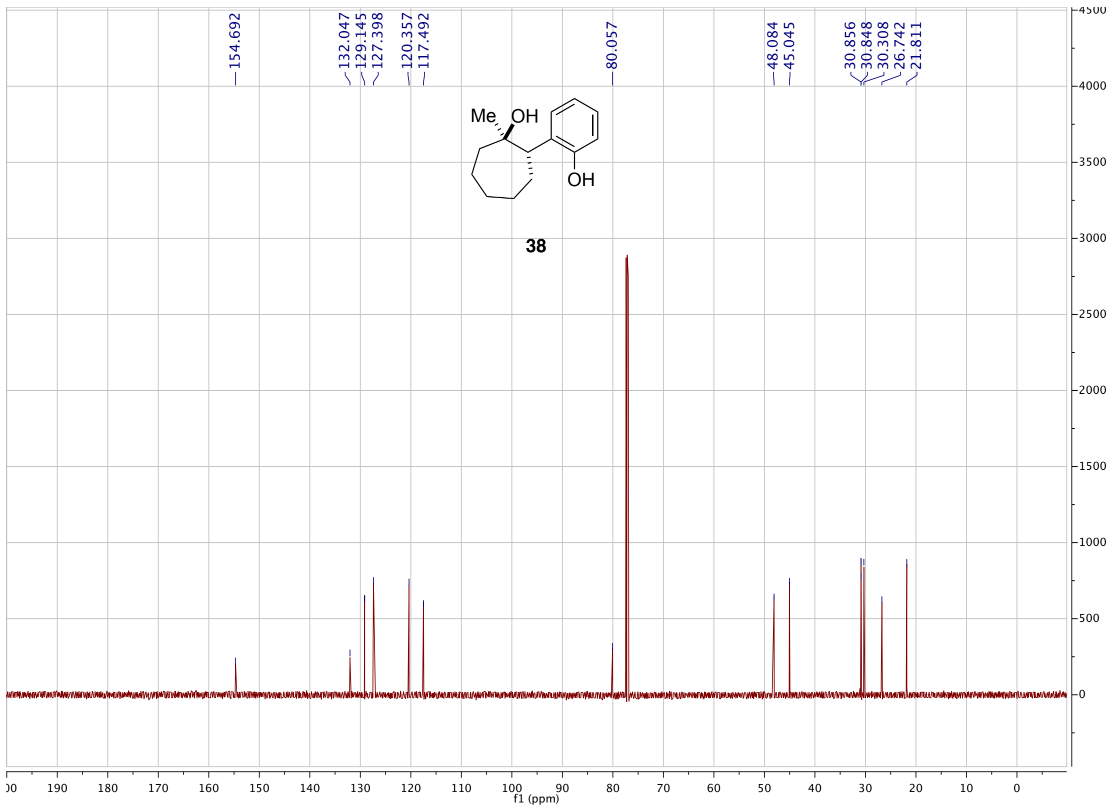

Figure S33. ^{1}H and ^{13}C NMR Spectra of **38**

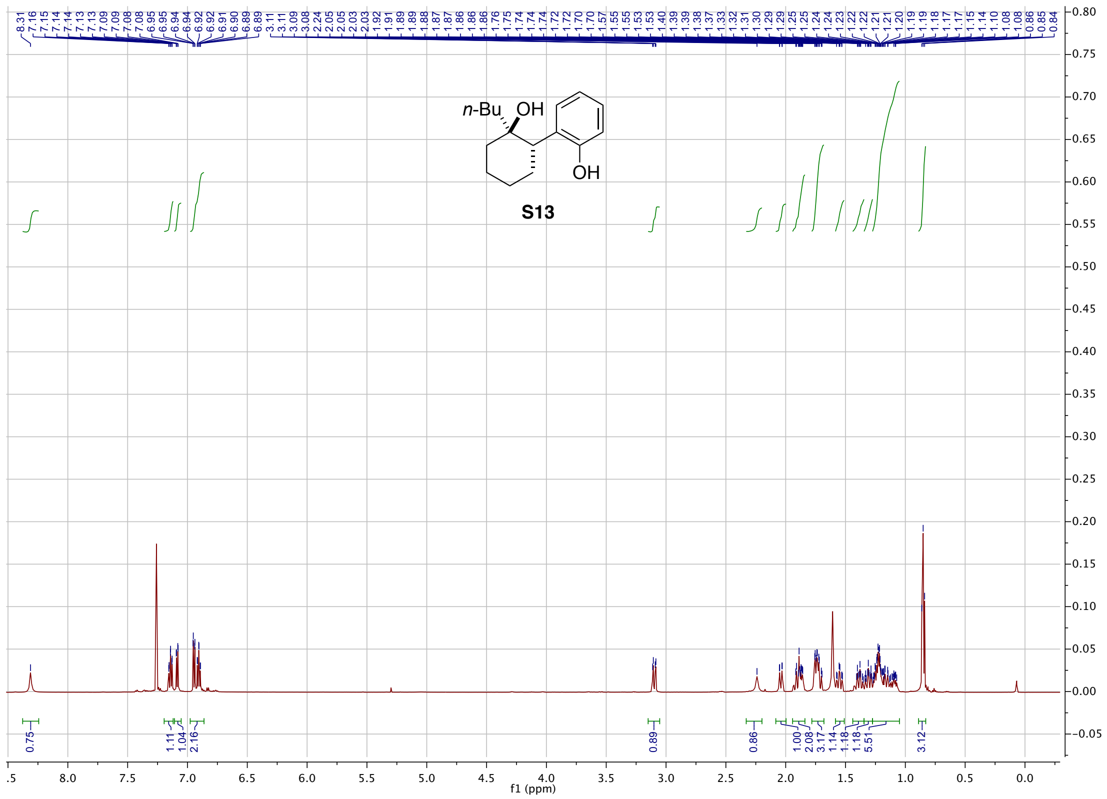

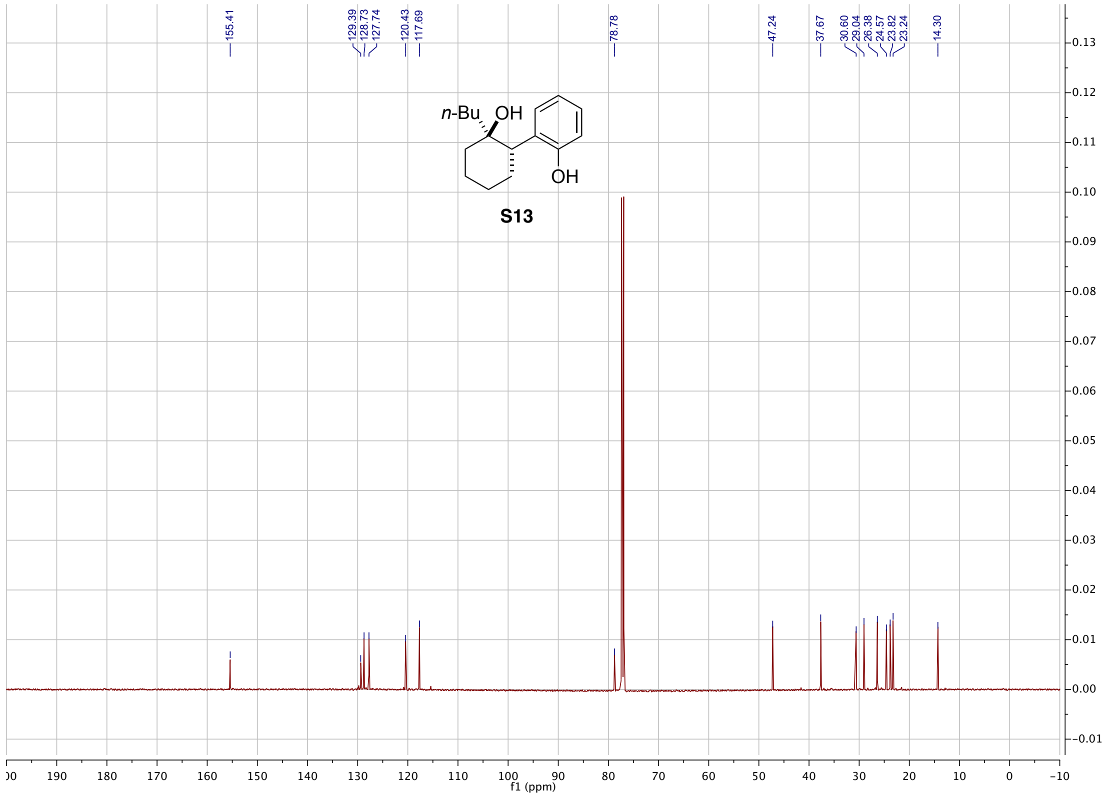

Figure S34. ^{1}H and ^{13}C NMR Spectra of **S13**

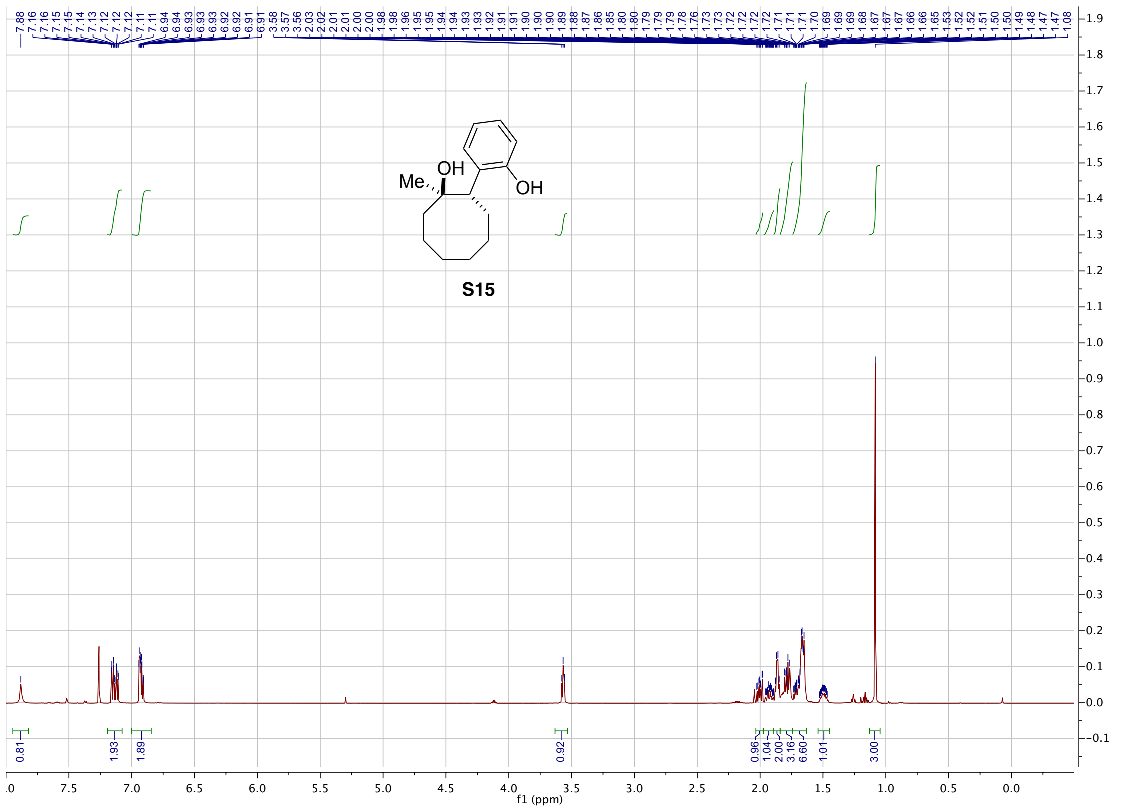

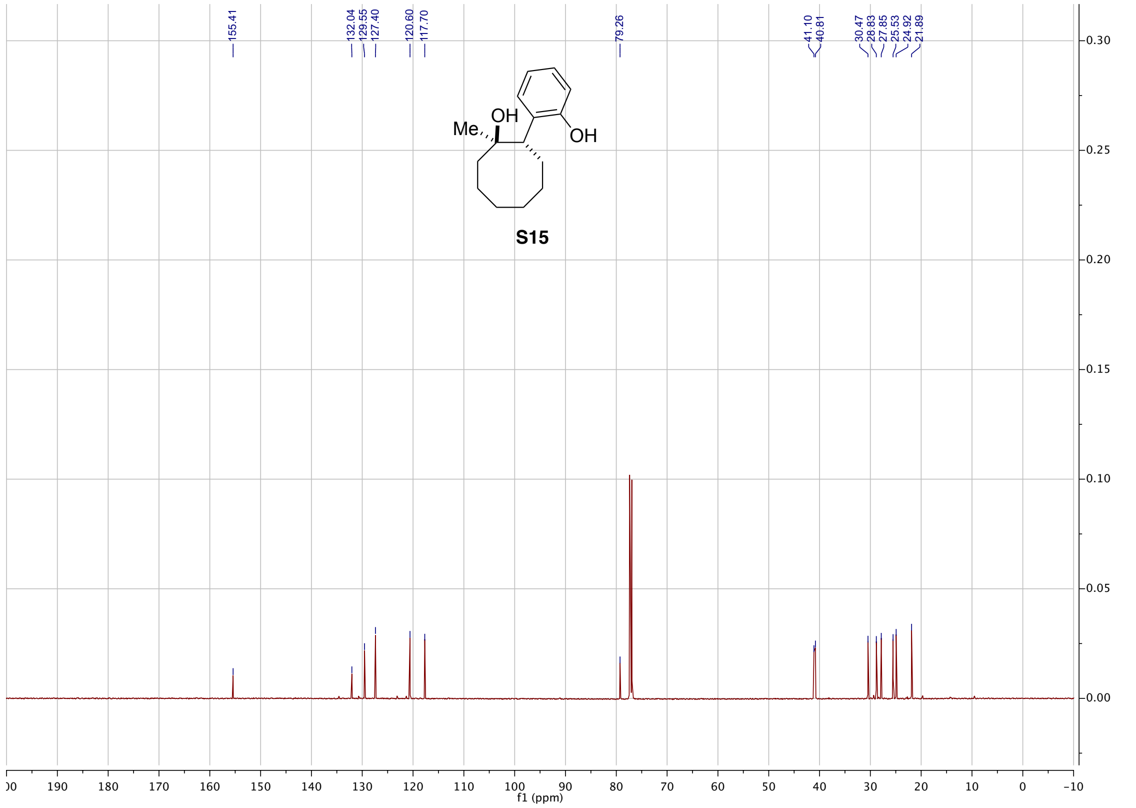

Figure S35. ^{1}H and ^{13}C NMR Spectra of **S15**

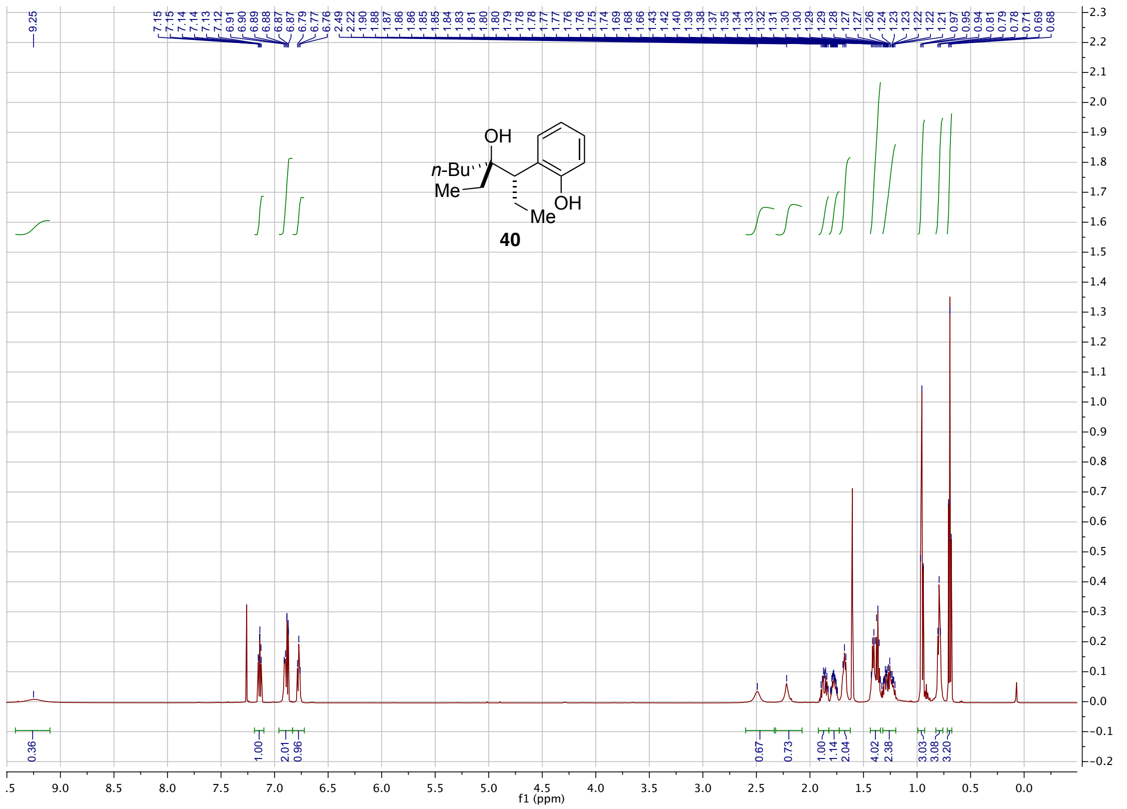

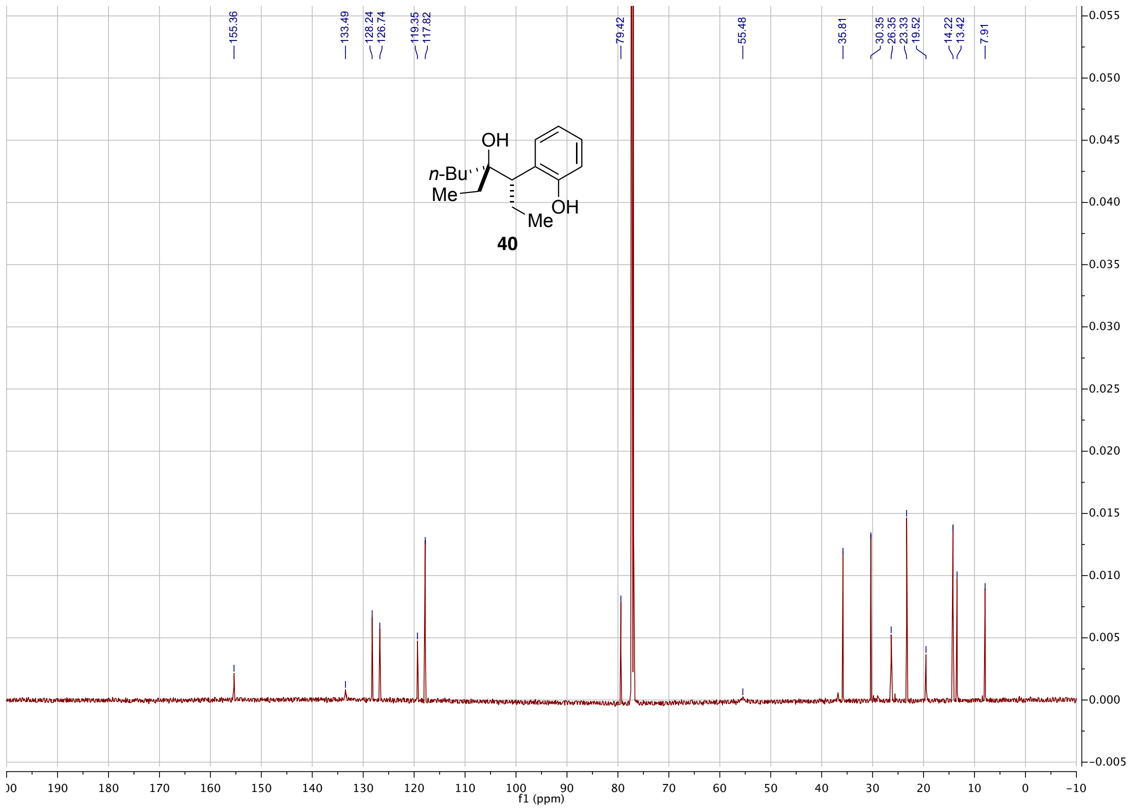

Figure S36. ^{1}H and ^{13}C NMR Spectra of **40**

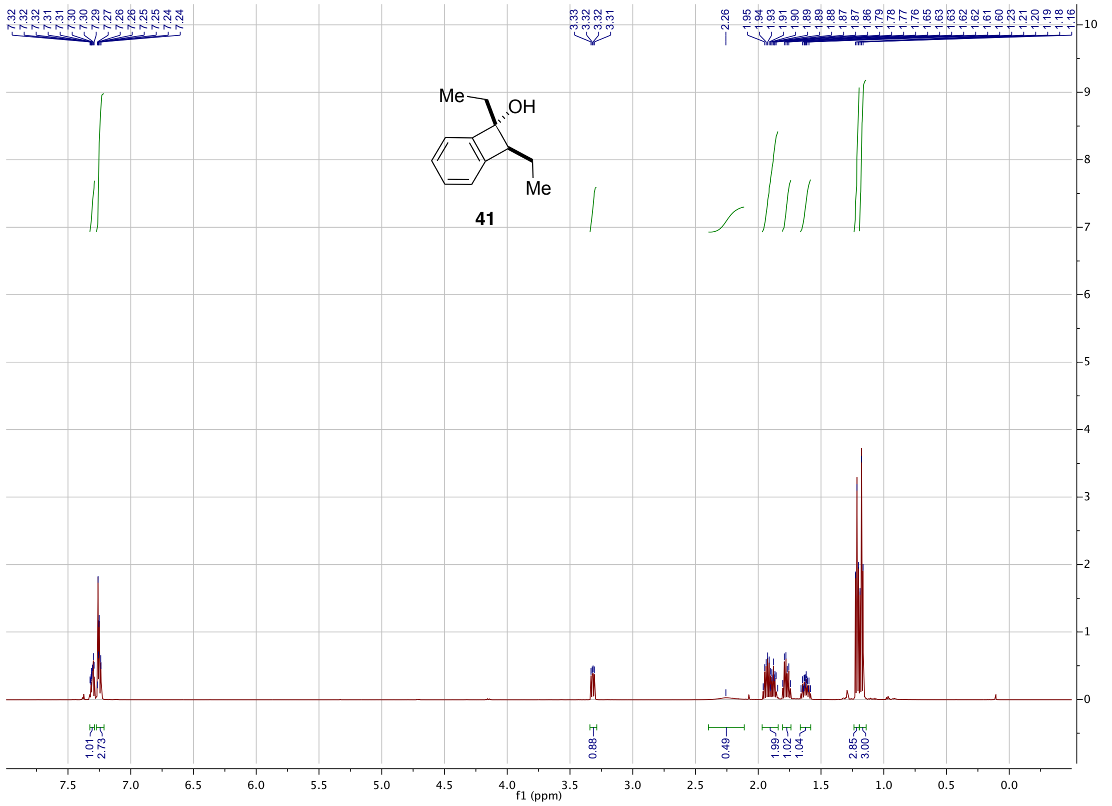

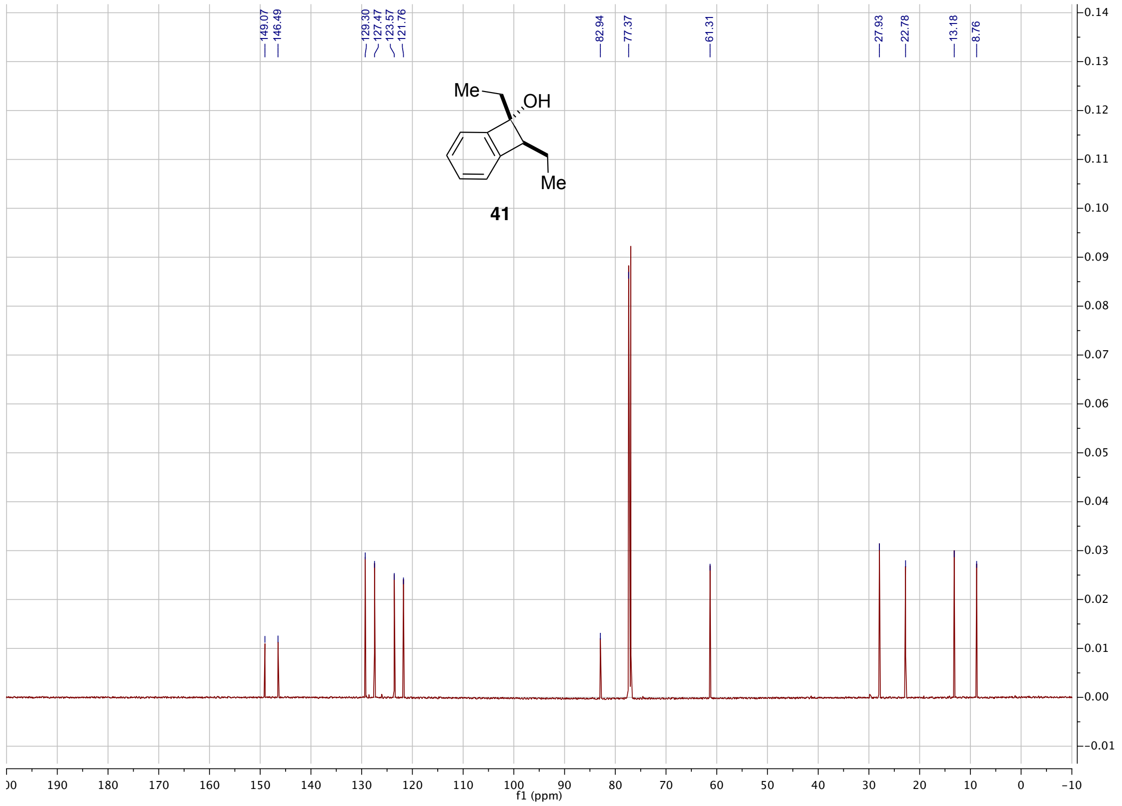

Figure S37. ^{1}H and ^{13}C NMR Spectra of **41**

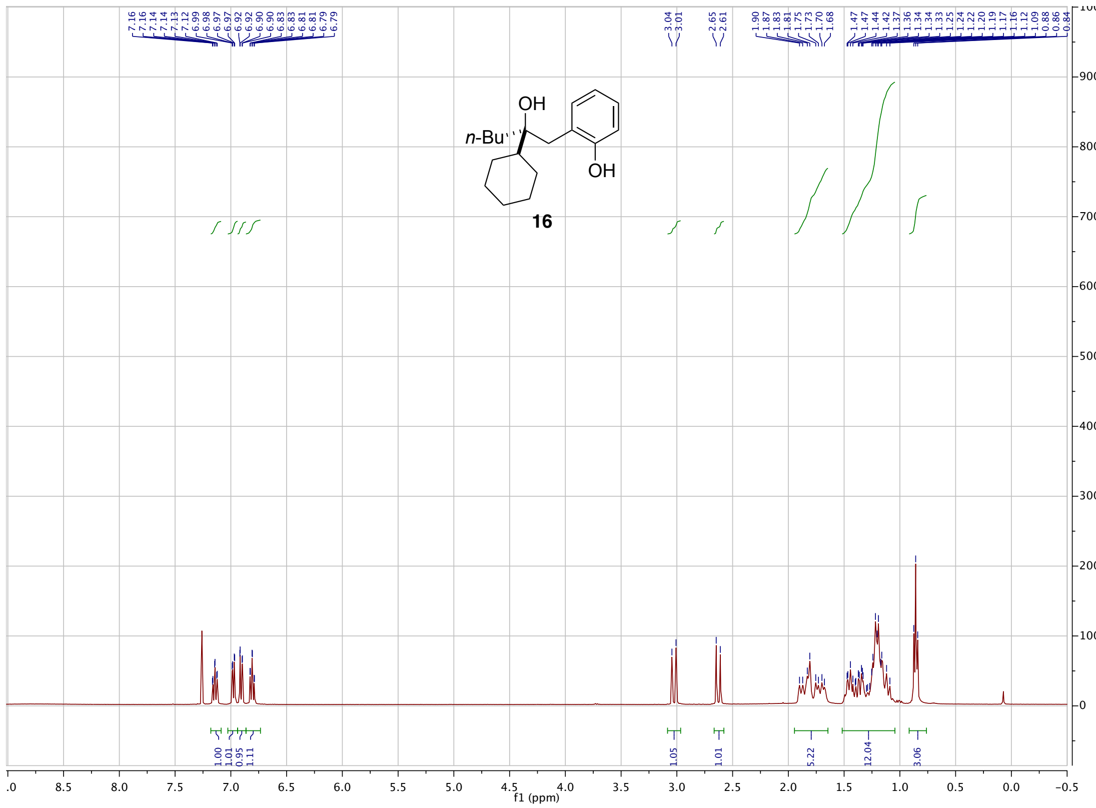

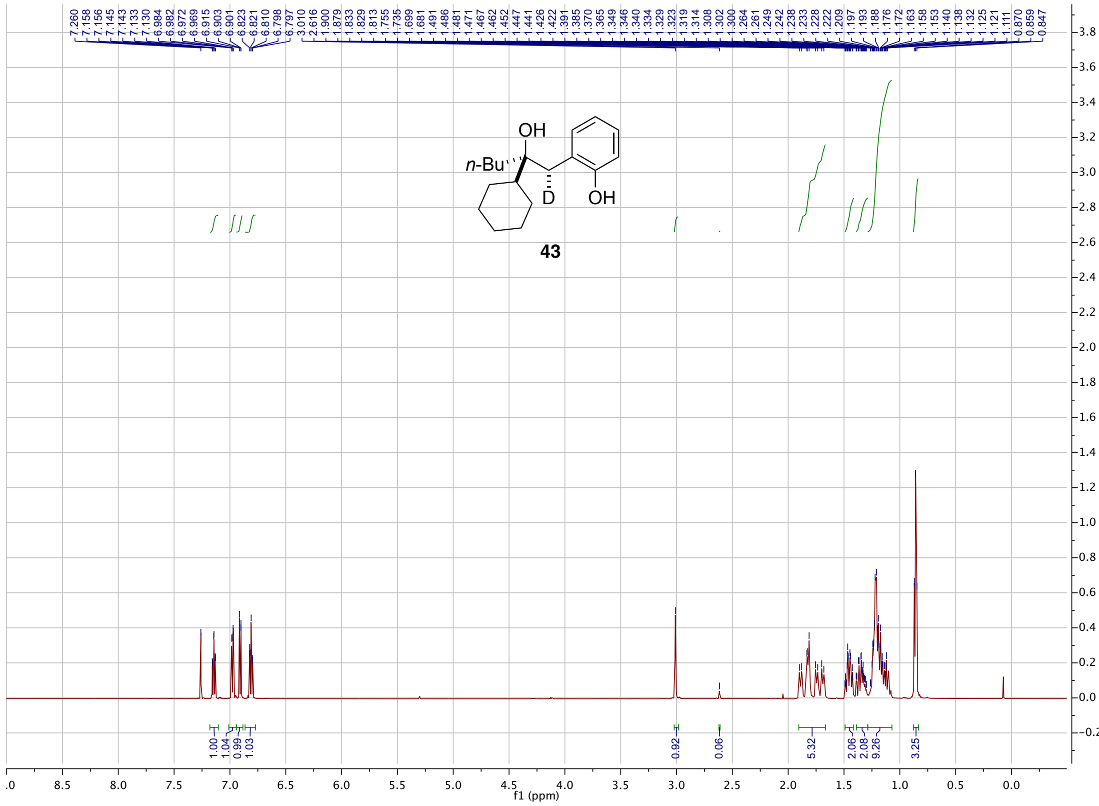

Figure S38. ^{1}H NMR Spectra of **16** and **43** (dr 15:1)

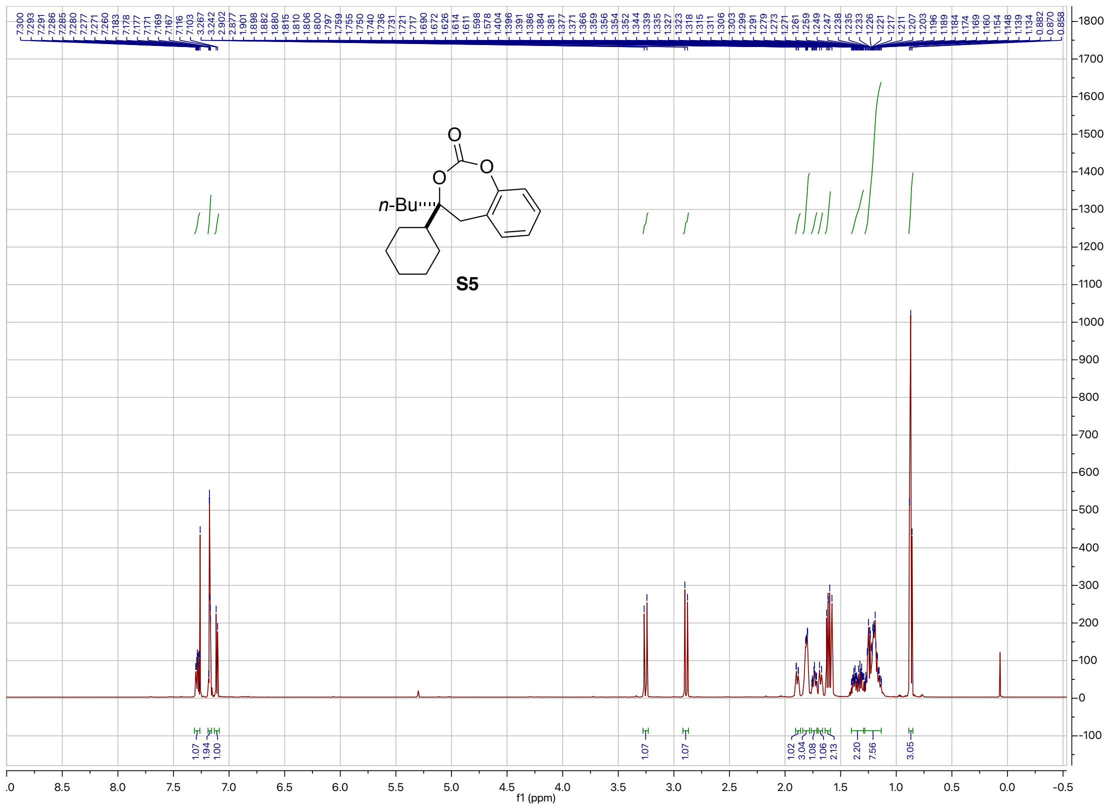

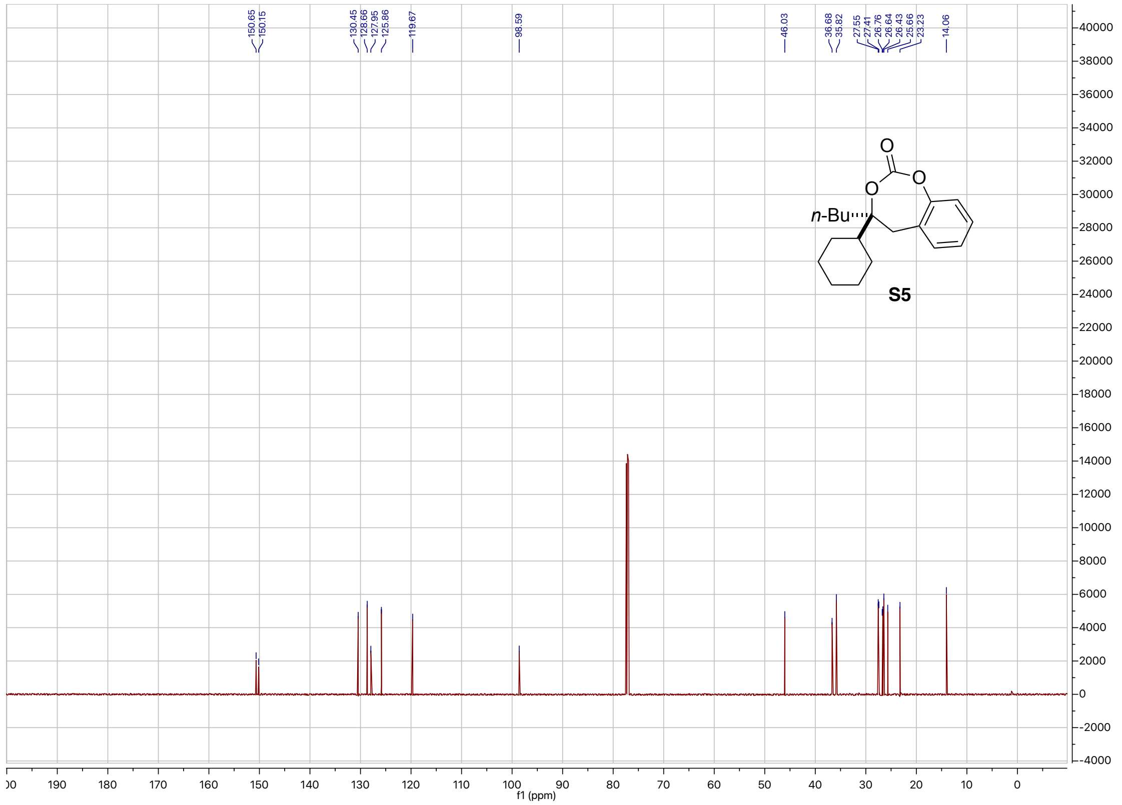

Figure S39. ^{1}H and ^{13}C NMR Spectra of **S5**

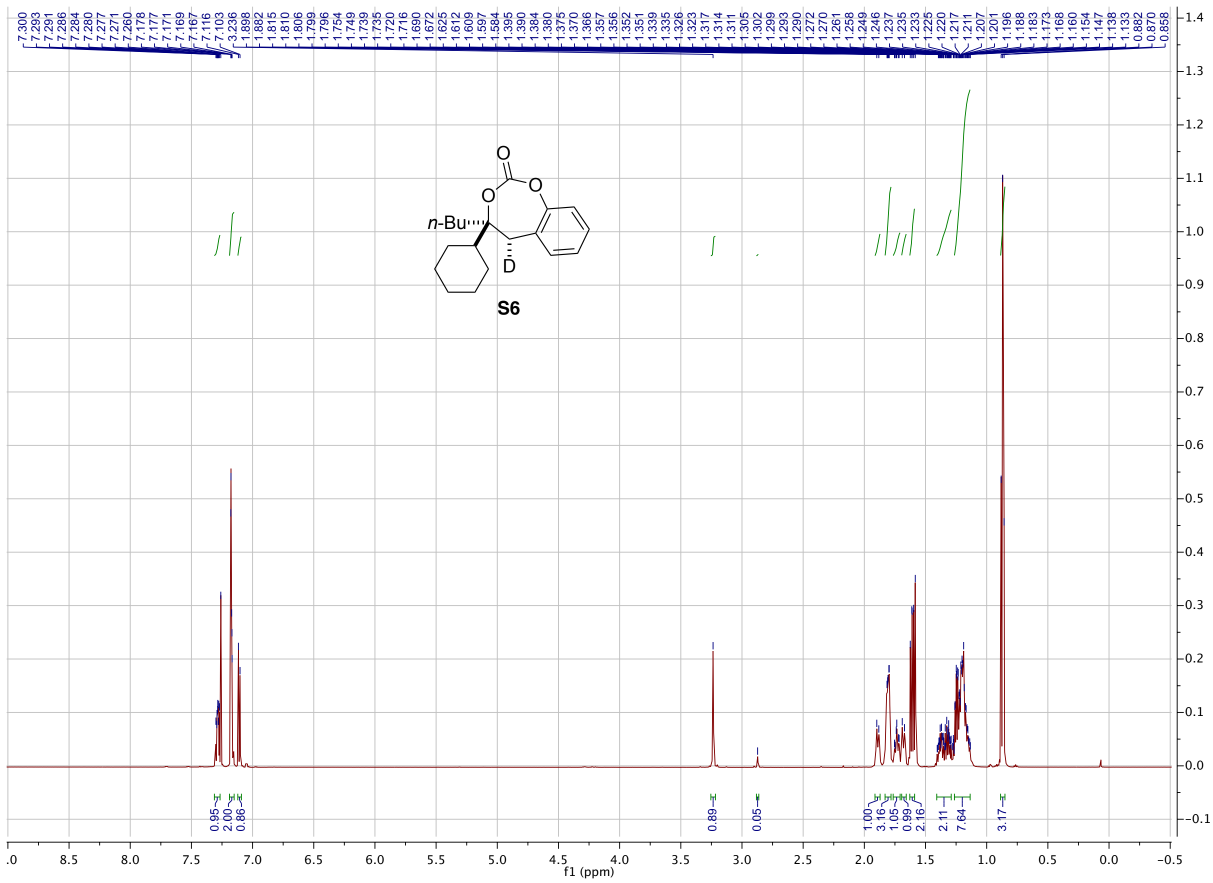

Figure S40. ^{1}H NMR Spectra of **S6** (dr 20:1)

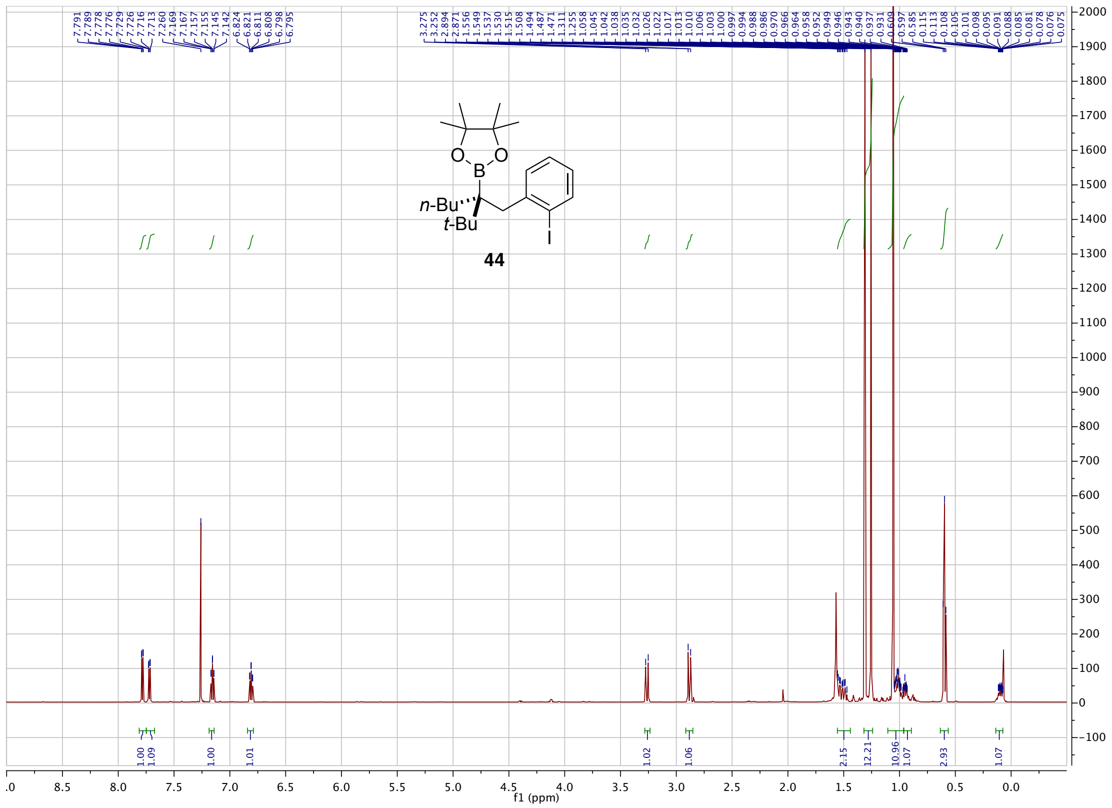

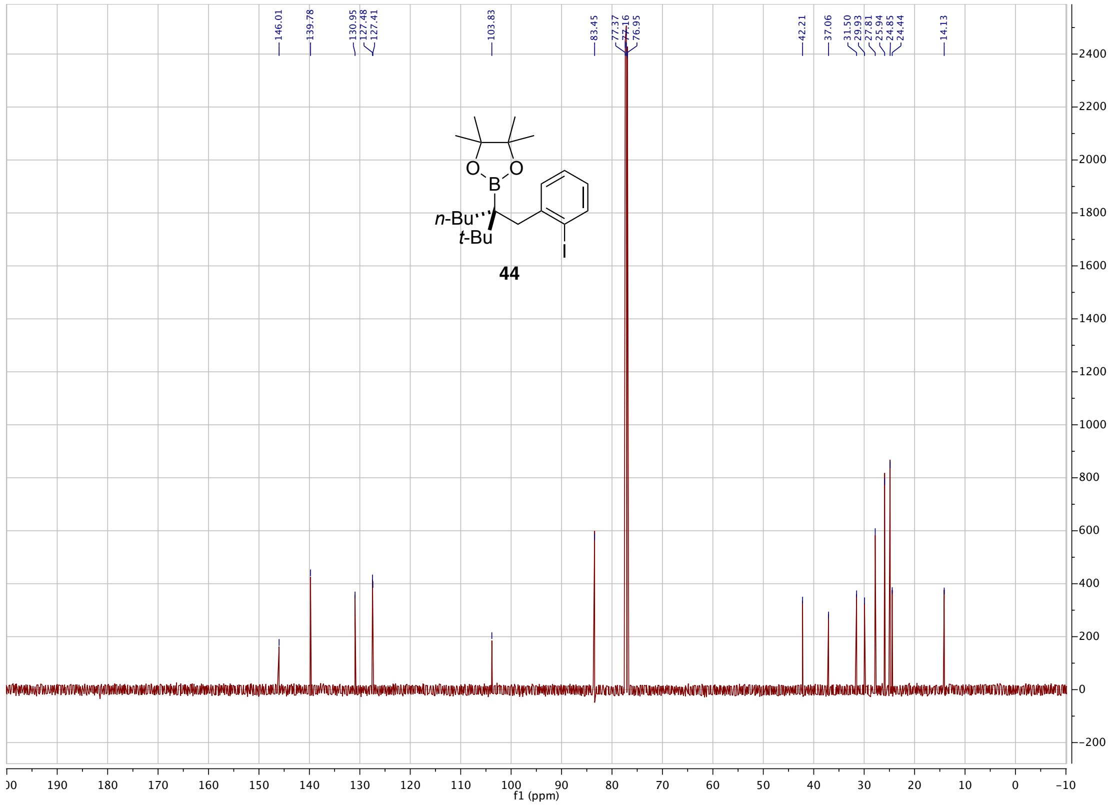

Figure S41. ^{1}H and ^{13}C NMR Spectra of **44**

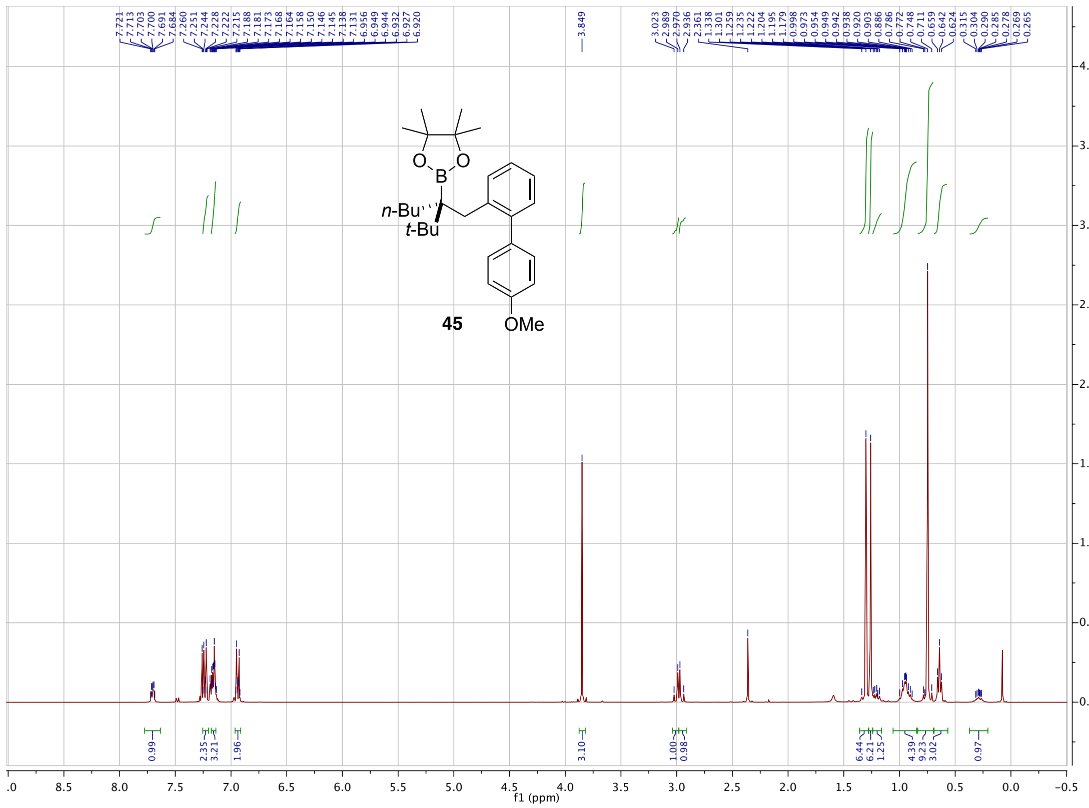

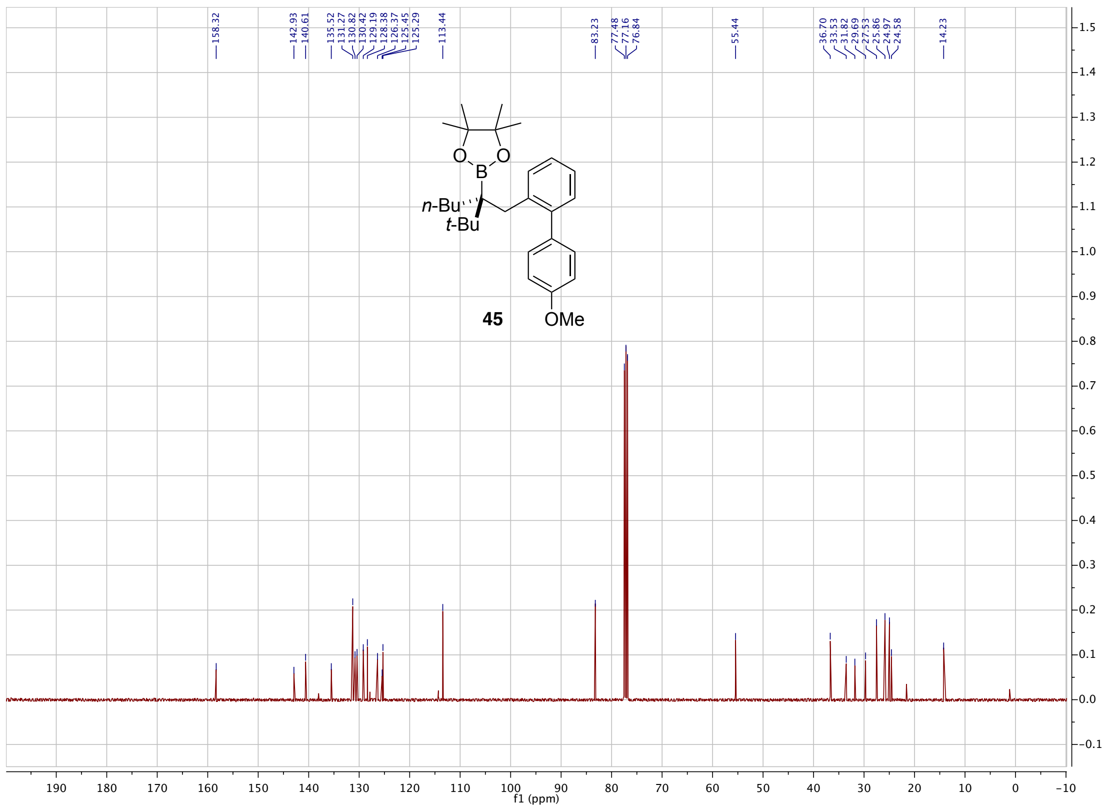

Figure S42. ^{1}H and ^{13}C NMR Spectra of **45**

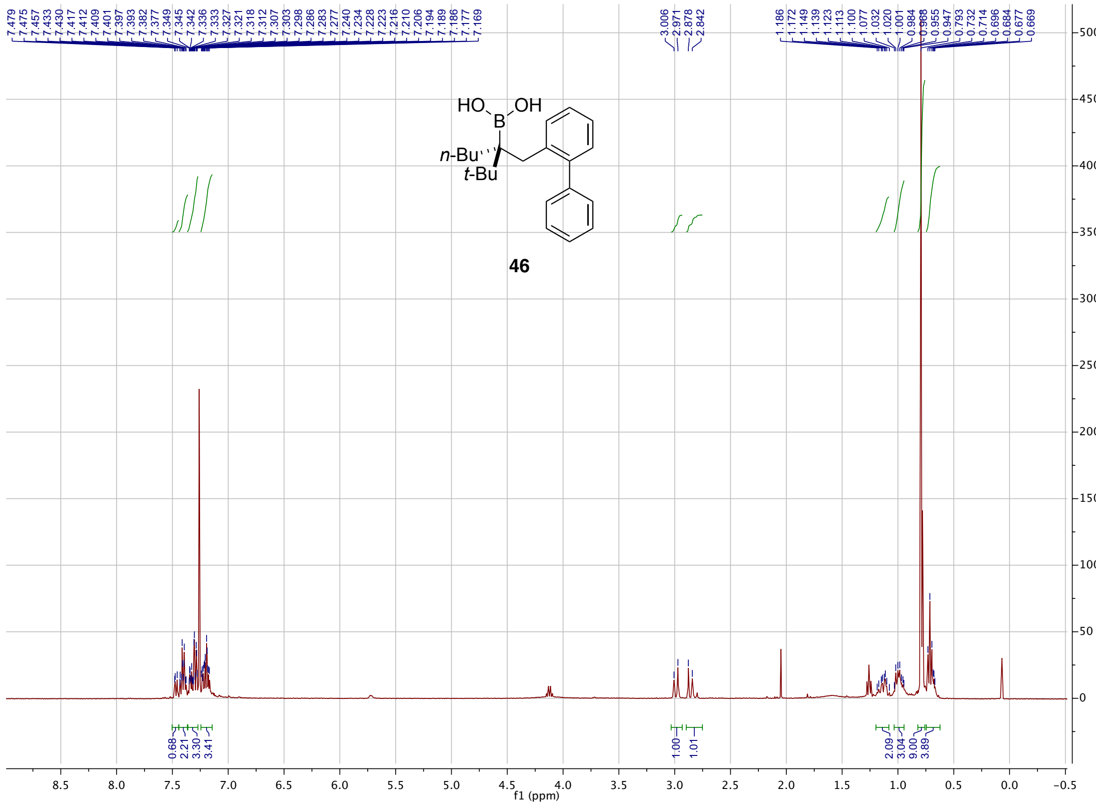

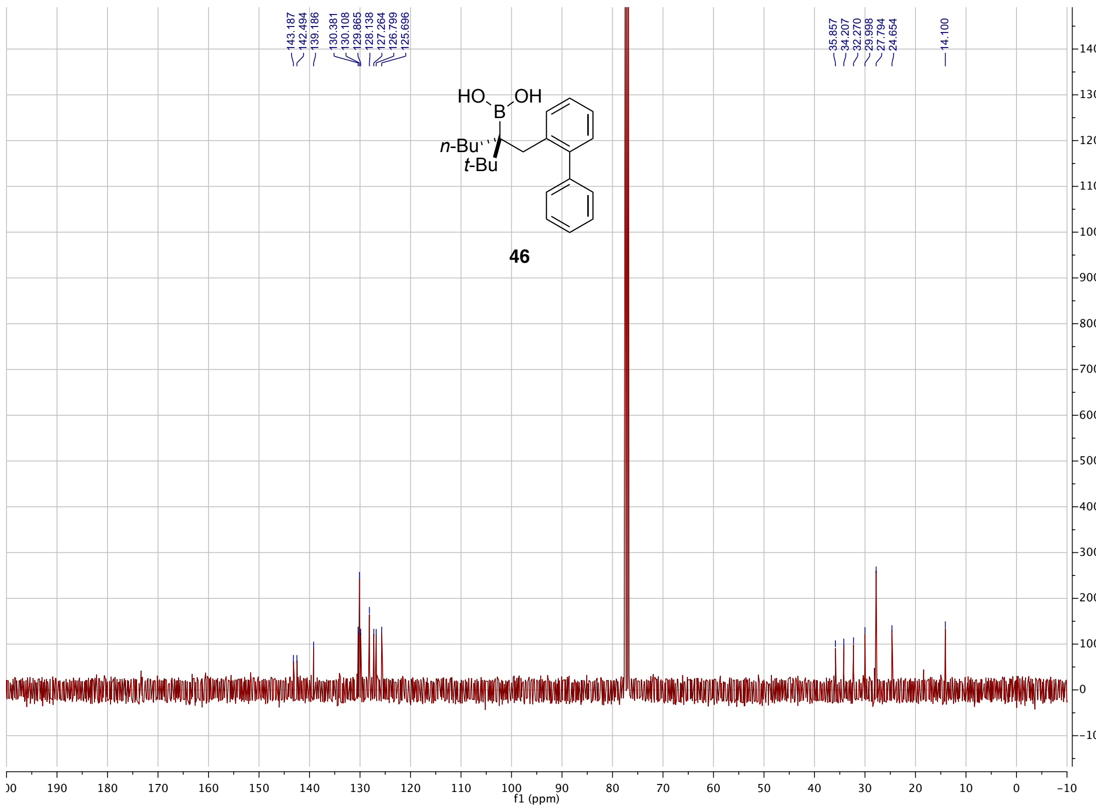

Figure S43. ^{1}H and ^{13}C NMR Spectra of **46**

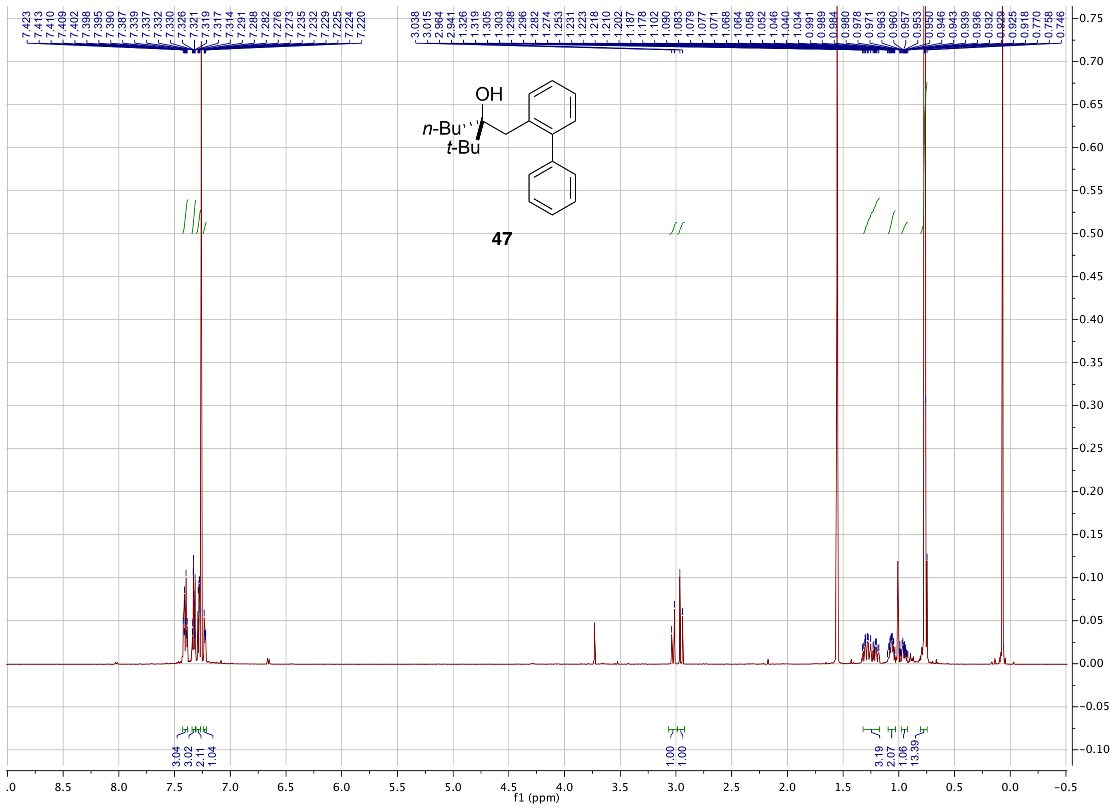

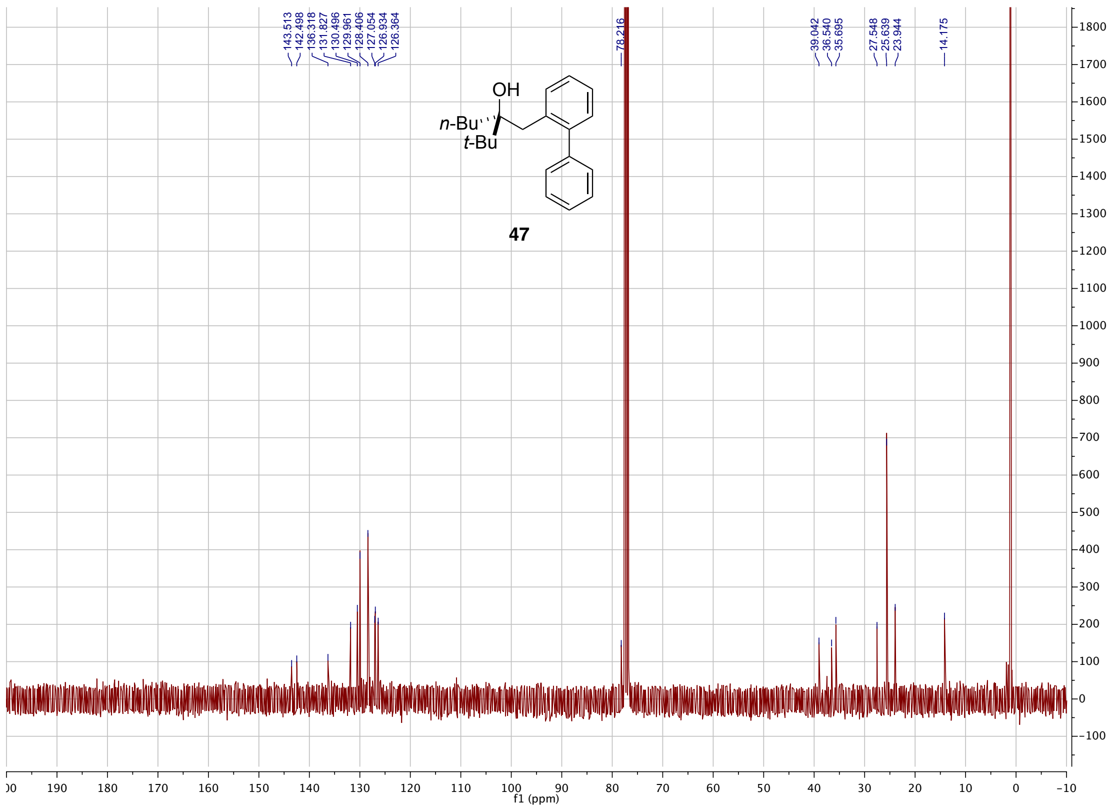

Figure S44. ^{1}H and ^{13}C NMR Spectra of **47**
